# Photo‐Mediated Silacyclization by Wavelength‐Dependent Selective C─F or C─H Functionalization

**DOI:** 10.1002/anie.202512420

**Published:** 2025-09-01

**Authors:** Gan Wang, Ye Yuan, Chu Wang, Bingjie Ren, Hwee Ting Ang, Rong Zhou, Jie Wu

**Affiliations:** ^1^ Department of Chemistry National University of Singapore 3 Science Drive 3 Singapore 117543 Singapore; ^2^ College of Chemistry and Chemical Engineering Taiyuan University of Technology Taiyuan 030024 P.R. China; ^3^ State Key Laboratory of Elemento‐Organic Chemistry Nankai University Tianjin 300071 P.R. China

**Keywords:** Cyclization, Heterocycles, Hydrogen evolution, Photocatalysis, Silicon

## Abstract

Silacycles have gained significant attention within the synthetic community due to their pivotal roles in medicinal chemistry and materials science. Despite recent advancements, the defluorosilylation of aryl fluorides with hydrosilanes and the selective silylation of arenes without external oxidants remains challenging. Herein, we present a wavelength‐dependent photo‐mediated cascade silacyclization of allylbenzene derivatives with dihydrosilanes to efficiently construct six‐membered benzosilacycles. By employing a synergistic system of an organophotocatalyst and a thiol‐based hydrogen atom transfer catalyst, our approach exploits specific light‐emitting diode (LED) wavelengths (456 and 335 nm) to achieve selective C–F and C–H functionalization. This wavelength modulation enables the first successful defluorosilacyclization of *ortho*‐fluoroallylbenzenes and facilitates an acceptorless dehydrosilacyclization, demonstrating precise site‐selective functionalization. Mechanistic investigations reveal a hydrogen atom transfer (HAT)‐facilitated intermolecular hydrosilylation followed by a wavelength‐dependent, chemoselective intramolecular silacyclization cascade. Additionally, an unprecedented light‐assisted hydrogen evolution process involving silane and thiol is uncovered within the cascade C–H silacyclization. This photo‐mediated strategy offers a sustainable and versatile platform for the synthesis of valuable silacycle compounds, exhibiting broad functional group tolerance and precise wavelength‐dependent chemoselectivity.

## Graphical abstract

A wavelength‐dependent, transition‐metal‐free cascade silacyclization of allylbenzene derivatives with dihydrosilanes is developed to enable the efficient construction of six‐membered benzosilacycles. Selective C–F functionalization is achieved under 456 nm LED irradiation, while irradiation at 335 nm promotes acceptorless dehydrosilacyclization via C–H functionalization.

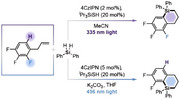

## Introduction

**Figure 1:**
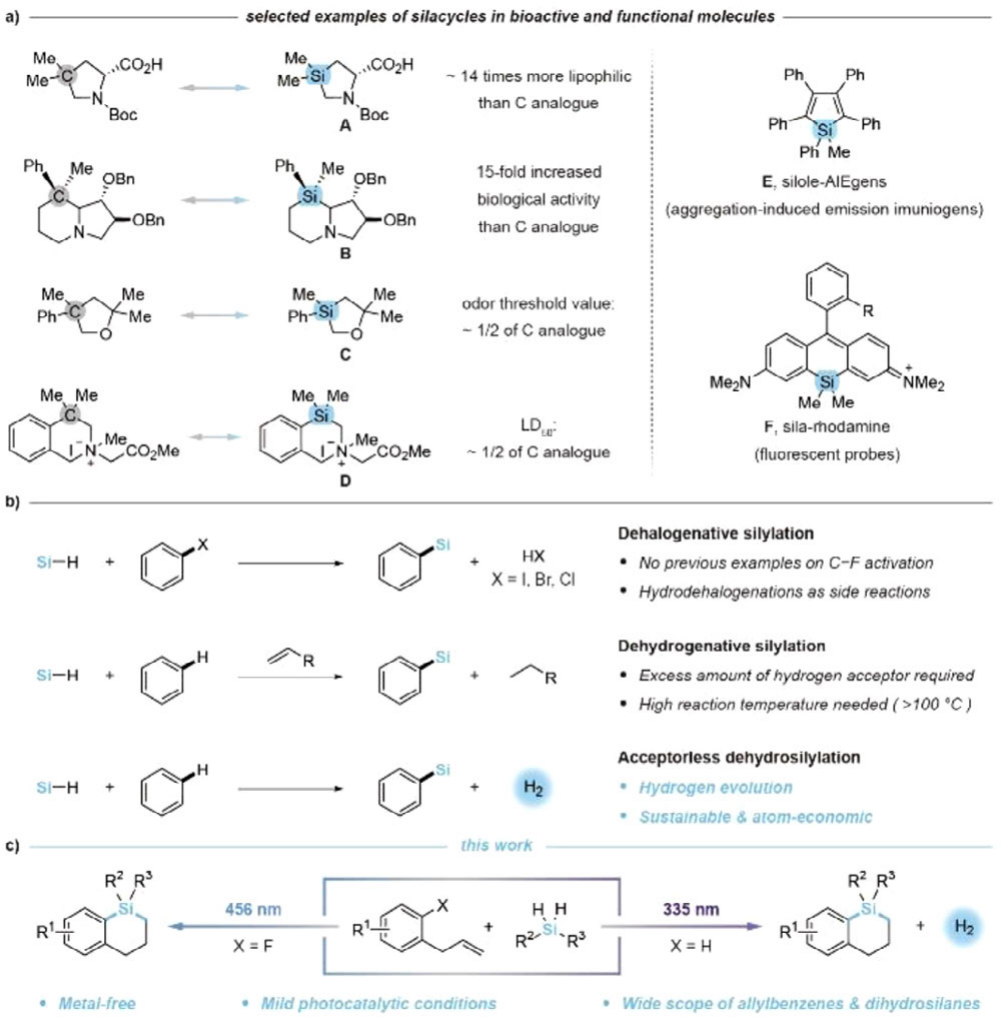
a) Representative bioactive and functional silacycles. b) Reported synthetic strategies for aryl C–Si bond formation. c) This work: Wavelength‐dependent selective C─F or C─H silylation.

Organosilicon compounds are pivotal across pharmaceutical, medicinal, and materials sciences. Among them, sila‐analogues, particularly silacycles, have garnered significant interest due to their enhanced lipophilicity, increased biological activity, and reduced toxicity compared to their carbon‐based counterparts. Representative examples, including sila‐proline **A**, sila‐lentiginosine **B**, sila‐tetrahydrofuran **C**, and sila‐tetrahydroisoquinoline **D**, underscore the promising potential of silacycles in medicinal chemistry (Figure 1a).^[^ ^1^, ^2^, ^3^, ^4^, ^5^, ^6^, ^7^, ^8^, ^9^ ^]^ Additionally, silacycles like silole‐AIEgens **E** and sila‐rhodamine fluorescent probes **F** demonstrate superior functional properties, highlighting their utility in materials science.^[^ ^10^, ^11^ ^]^ Given their extensive applicability, there is a growing focus on developing efficient synthetic methodologies for silacycles.

Traditional approaches for aryl C─Si bond formation rely on highly reactive organometallic reagents and silicon electrophiles, such as halosilanes.^[^ ^12^, ^13^, ^14^ ^]^ Although significant progress has been made using transition‐metal‐catalyzed aryl silylation strategies, these methods often require expensive catalysts and elevated temperatures, limiting their environmental and practical appeal. Recent advances in photo‐mediated silylation strategies present promising alternatives; however, these approaches often require excess oxidants, complicating their application and remaining predominantly confined to heteroarenes.^[^ ^15^, ^16^, ^17^, ^18^, ^19^, ^20^ ^]^ In this context, the direct functionalization of aryl C–X (X = halogen atom) and C–H bonds have gained increasing attention due to their abundance and potential to streamline synthetic processes, offering a greener and more atom‐economical approach to aryl C─Si bond formation. While aryl halides, particularly aryl iodides and bromides, have been extensively investigated in these reactions, the defluorinative functionalization of aryl fluorides with hydrosilanes to form C─Si bonds remains unreported (Figure 1b, top).^[^ ^21^, ^22^, ^23^, ^24^, ^25^, ^26^ ^]^ This challenge arises from the high bond dissociation energy (BDE) of the C─F bond in aryl fluorides (e.g., Ph─F: 127.2 kcal mol^−1^) and the strong Si─F bond formation (BDE: ∼160 kcal mol^−1^) with hydrosilanes.^[^ ^21^, ^22^, ^27^ ^]^ Additionally, the reducing nature of hydrosilanes often leads to competitive reduction of aryl halides, further complicating dehalogenative cross‐couplings. On the other hand, the dehydrosilylation of arene C─H bonds with hydrosilanes has made significant progress over the past decade, particularly with noble transition metal catalysts such as Ru, Rh, Ir, and Pt in the presence of excess hydrogen acceptors (Figure 1b, middle).^[^ ^28^, ^29^, ^30^ ^]^ Notably, this method has evolved into the acceptorless dehydrosilylation strategy, deemed the most sustainable silylation strategy due to its elimination of hydrogen acceptors and concurrent hydrogen evolution (Figure 1b, bottom). However, these reactions typically rely on precious transition metal catalysts and high reaction temperatures. Although alternative catalytic systems based on Fe, Sc, and *^t^* BuOK have occasionally enabled the acceptorless dehydrosilylation of specific arenes or heteroarenes,^[^ ^31^, ^32^, ^33^, ^34^ ^]^ their application scope remains restricted. These limitations highlight the need for innovative strategies that offer broader substrate scope and milder reaction conditions.

Recently, our research group has contributed to the field of C–F functionalization by developing the defluoroborylation of polyfluoroarenes through photoredox and hydrogen atom transfer (HAT)‐induced B─H activation of N‐heterocyclic carbene (NHC)‐boranes.^[^ ^35^ ^]^ Additionally, we reported an efficient and versatile neutral eosin Y‐based HAT photocatalysis strategy for the functionalization of multi‐hydrosilanes.^[^ ^36^ ^]^ Building on these foundations, we now introduce a wavelength‐dependent photo‐mediated cascade silacyclization of allylbenzenes with dihydrosilanes to access six‐membered benzosilacycles (Figure 1c). This method employs a synergistic catalytic system that comprises the organophotocatalyst 1,2,3,5‐tetrakis(carbazol‐9‐yl)‐4,6‐dicyanobenzene (4CzIPN) and the thiol HAT reagent *^i^* Pr_3_SiSH, enabling selective C–F or C–H silacyclization by switching the wavelength of the light source. Specifically, irradiation with a 456 nm LED facilitates defluorosilacyclization, while 335 nm light promotes acceptorless dehydrosilacyclization. This innovative approach not only advances the synthesis of silacycles but also represents a rare example of chemoselective functionalization achieved through wavelength modulation.

## Results and Discussion

### Reaction Optimization and Substrate Scope of Defluorosilacyclization

Using allylpentafluorobenzene (**1a**) and methylphenylsilane (**2a**) under 40 W blue light (456 nm) irradiation, with 4CzIPN as the photocatalyst and *^i^* Pr_3_SiSH as the HAT reagent, the desired silacyclization product (**3a**) was obtained in 86% yield in tetrahydrofuran (THF) at 40 °C in the presence of K_2_CO_3_ (Table S1). Alternative photocatalysts were explored; eosin Y and [Ir(dF(CF_3_)ppy)_2_ (dtbbpy)]PF_6_ led to lower yields, while no reaction occurred with [Ir(ppy)_2_(dtbbpy)]PF_6_ or *fac*‐Ir(ppy)_3_. Among various HAT reagents evaluated, only trimethoxysilylpropanethiol led to the formation of **3a**, albeit at a comparatively low yield. Other sulfur‐centered HAT reagents, including 2,4,6‐trimethylbenzenethiol, 3‐mercaptopropanoic acid, ethyl 2‐mercaptopropanoate, 3‐mercaptobutan‐2‐one, 1,2‐dibenzyldisulfane, phenylmethanethiol, and triphenylmethanethiol failed to produce the desired product. Solvent screening revealed that THF is the optimal medium, outperforming alternatives such as 1,4‐dioxane and CH_2_Cl_2_. Given the critical role of the base in the defluorination step, various base additives were tested; K_2_CO_3_ proved to be superior to organic bases like 1,8‐diazabicyclo[5.4.0]undec‐7‐ene (DBU) and other inorganic bases such as K_2_HPO_4_. Reducing the photocatalyst loading from 5 to 2 mol% resulted in diminished yield, and extending the reaction time to 24 h also led to a slight decline in efficiency. Control experiments confirmed the essential roles of light, the photocatalyst, and the HAT reagent as the omission of any of these components completely suppressed product formation.

With the optimized conditions established, we proceeded to investigate the scope of dihydrosilanes **2** (Table 1). When R^2^ is a phenyl ring, the reaction proceeded efficiently, affording product **3b** in a 90% yield. Methyl‐substituted aryl groups at various positions were also well tolerated, delivering the corresponding products (**3c**–**3e**). However, aryl groups with halo‐substituents (e.g., Cl, F) exhibited relatively lower reactivity (**3f**–**3h**). Aryl groups with para‐substituted bulky *tert*‐butyl or electron‐donating methoxy groups yielded products **3i** and **3j** in good yields. A biphenyl group as R^2^ resulted in a moderate yield (**3k**). Notably, heterocycles, such as a thienyl group, also underwent silacyclization, giving product (**3l**) in moderate yield. The reaction also demonstrated broad tolerance for various alkyl groups as R^2^, including linear chains (ethyl, *n*‐hexyl), branched chains (*i*‐butyl), and cyclic groups (cyclopentyl, cyclohexyl), furnishing the corresponding benzosilacycles in moderate to high yields (**3m**–**3q**). Additionally, dialkyl‐substituted silanes, such as diethylsilane, were viable substrates for this transformation, yielding **3r** in moderate yield. Trihydrosilanes, however, proved unsuccessful, leading to a complex mixture. Beyond silacyclization, this methodology was successfully extended to germane substrates, enabling the synthesis of benzogermacycles. Under the standard conditions, diphenylgermane underwent cascade germacyclization with allylpentafluorobenzene (**1a**), affording product **3s** in moderate yield.

**Table 1:** Substrate scope of defluorosilacyclization.^a)^

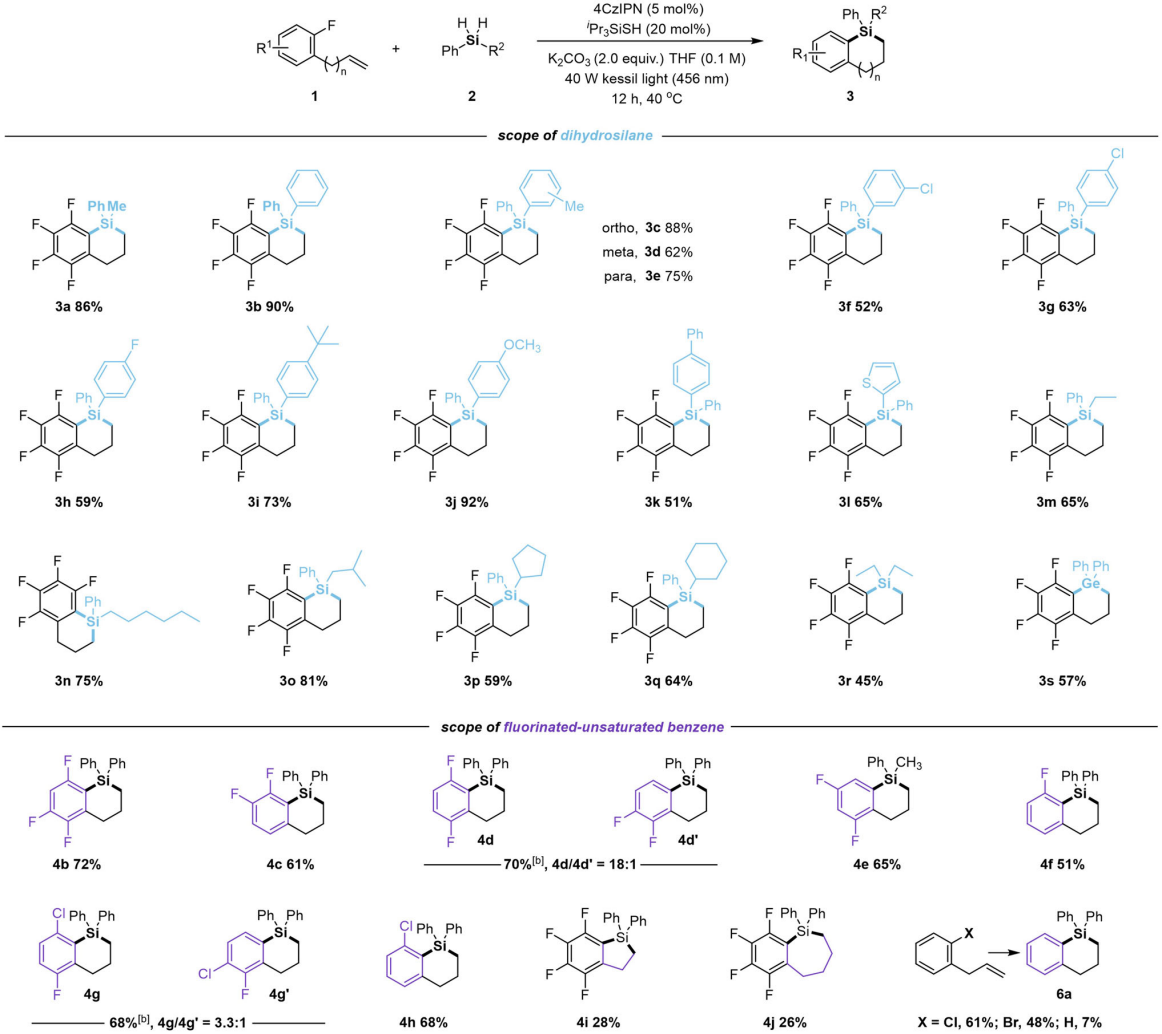

^a)^ Reaction conditions: **1** (0.10 mmol), **2** (0.15 mmol), 4CzIPN (5 mol%), *^i^* Pr_3_SiSH (20 mol%), K_2_CO_3_ (2 equiv.) in THF (0.1 M) at 40 °C under 40 W 456 nm LED irradiation for 12 h.

^b)^ Overall isolated yields. Regioselectivity is analyzed by GC or ^1^H NMR analysis of the crude reaction mixture.

^c)^ Isolated yields.

The scope of fluorinated allylbenzenes **1** was subsequently investigated with representative dihydrosilanes under standard conditions (Table 1). The silacyclization of tetrafluorinated allylbenzene **1b** proceeded efficiently, affording product **4b** in good yield. Trifluorinated allylbenzenes at various positions successfully underwent cyclization to furnish the corresponding silacycles in moderate to good yields (**4c**–**4e**). Difluorinated substrates such as 1,2‐ and 1,3‐difluorinated allylbenzenes also demonstrated good compatibility (**4f** and **4g**). A monofluorinated benzene derivative with a chlorine substituent at the 3‐position reacted efficiently to furnish product **4h**. For substrates with regioselectivity issues (**4d** and **4g**), the reactions preferentially occur at the more electron‐deficient position, consistent with the nucleophilic character of silyl radicals. Notably, this methodology could be extended to the synthesis of both 5‐ and 7‐membered benzosilacycles using vinylpentafluorobenzene and homoallylpentafluorobenzene as substrates, respectively, yielding **4i** and **4j**. Moreover, other halo‐substituents such as chlorine and bromine at the 2‐position also underwent a dehalosilylation process, leading to the formation of benzosilacycles (**6a**). Interestingly, treating nonhalogenated allylbenzene with diphenylsilane under the standard reaction conditions produced silacycle **6a** in 7% yield, an unexpected dehydrogenative cyclization that warrants further investigation.^[^ ^37^ ^]^

### Reaction Optimization and Substrate Scope of Acceptorless Dehydrosilacyclization

After systematically optimizing the reaction parameters for the dehydrosilacyclization of allylbenzene (**5a**) and diphenylsilane (**2b**), we found that changing the light wavelength to 335 nm resulted in the desired product **6a** in 73% isolated yield in MeCN using a catalytic amount of 4CzIPN and *^i^* Pr_3_SiSH (Table S2, entry 1). The reaction was accompanied by hydrogen evolution, as confirmed by GC analysis. Replacing either 4CzIPN or *^i^* Pr_3_SiSH with other photocatalysts or thiol‐based HAT reagents resulted in significantly lower yields (entries 2 and 3). Solvent screening identified MeCN as the optimal medium, while other solvents such as *^t^* BuCN, CH_2_Cl_2_, THF, and 1,4‐dioxane provided comparable or lower yields (entry 4). The addition of either basic (e.g., DBU, K_2_CO_3_) or acidic (e.g., AcOH, PhCO_2_H) additives proved detrimental to the reaction (entries 5–8). Increasing the photocatalyst loading to 5 mol% (entry 9) and altering the irradiation wavelength to 456 or 390 nm (entries 10 and 11) resulted in decreased product yields. Furthermore, shortening the reaction time to 24 h led to a reduced yield (entry 12). Control experiments confirmed that the presence of the photocatalyst, thiol, and light are all essential for the success of this acceptorless dehydrosilacyclization (entry 13).

The generality of this acceptorless dehydrosilacyclization was explored under the optimal conditions (Table 2). Employing diphenylsilane (**2b**) as a model substrate, a wide range of allylbenzenes was investigated. Monosubstituted allylbenzenes, bearing either electron‐donating or electron‐withdrawing groups on the aryl ring, reacted smoothly with diphenylsilane to produce the corresponding benzosilacycles (**6b**–**6k**) in good yields. Notably, the position of the substituent on the aryl ring had a minimal impact on the reaction efficiency, as demonstrated by the comparable yields obtained for 2‐, 3‐, and 4‐methyl‐substituted allylbenzenes (**6b**–**6d**). Among them, the 2‐methyl‐substituted allylbenzene showed a slight preference for forming **6b** over **6b′**. A variety of disubstituted allylbenzenes (**6l**–**6p**), with different substituents, including halogens (**6f**, **6g**, and **6o**), trifluoromethyl (**6h** and **6p**), cyano (**6i**), ester (**6j** and **6k**), and methoxy (**6n**), was well‐tolerated. For compound **6b** and 3, 4,‐disubstituted allybenzens (**6n**‐**6p**), product formation would typically favored the less sterically hindered positions (**6n** and **6p**), consistent with thermodynamic stability (with **6p** yielding only one regioisomer). Suprisingly, substrates **6b** and **6o** exhibited unusual regioselectivity, favoring formation at the more sterically congested sites. To rationalize this counterintuitive regioselectivity, we calculated the Fukui indices^[^ ^38^ ^]^ (*f* ^−^) for **6b** and **6o** (Figure S15). The results suggest that the observed regioselectivity arises from the enhanced nucleophilic character at the sterically hindered positions, as reflected by their relatively higher *f* ^−^ values. In addition to allylbenzenes, sterically hindered allylnaphthalenes were also viable substrates (**6q** and **6r**). The α‐position of naphthalene exhibited higher reactivity toward electrophiles due to its greater electron density compared to the β‐position, resulting in favorable regioselectivity for **6r**. Furthermore, diallylbiphenyl underwent smooth cyclization, affording the bisbenzosilacycle (**6s**) in moderate yield. Other dihydrosilanes, including substituted diarylsilanes, arylalkylsilanes, and dialkylsilanes, were also successfully applied to acceptorless dehydrosilacyclization with allylbenzene, yielding the corresponding benzosilacycles (**7a**‐**7d**) in moderate yields. The yield of **7e** was relatively low, likely due to insufficient absorption at 335 nm irradiation.

**Table 2:** Scope of acceptorless dehydrosilacyclization.^a)^

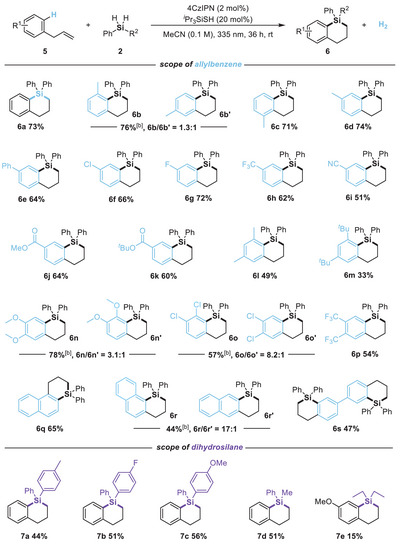

^a)^ Reaction conditions: **5** (0.10 mmol), **2b** (0.15 mmol), 4CzIPN (2 mol%), *^i^* Pr_3_SiSH (20 mol%), in MeCN (0.1 M) under 335 nm light irradiation at rt for 36 h.

^b)^ Overall isolated yields. Regioselectivity is analyzed by GC or ^1^H NMR analysis of crude reaction mixture.

^c)^ Isolated yields.

### Wavelength‐Dependent Chemoselective Silacyclization

**Scheme 1:**
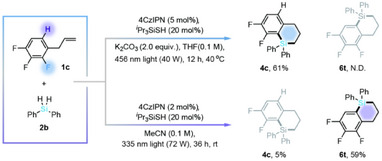
Wavelength‐dependent chemoselective silacyclization.

Recognizing the distinct wavelength requirements for defluoro‐ and dehydrosilacyclization, we achieved chemoselective cyclization by employing the appropriate irradiation wavelength. As illustrated in Scheme 1, the treatment of 2,3,4‐trifluoroallylbenzene (**1c**) with diphenylsilane (**2b**) under 456 nm light irradiation exclusively yielded the defluorosilacyclization product **4c**. In contrast, irradiation at 335 nm predominantly facilitated dehydrosilacyclization, affording mainly product **6t**.

### Mechanistic Studies

**Figure 2:**
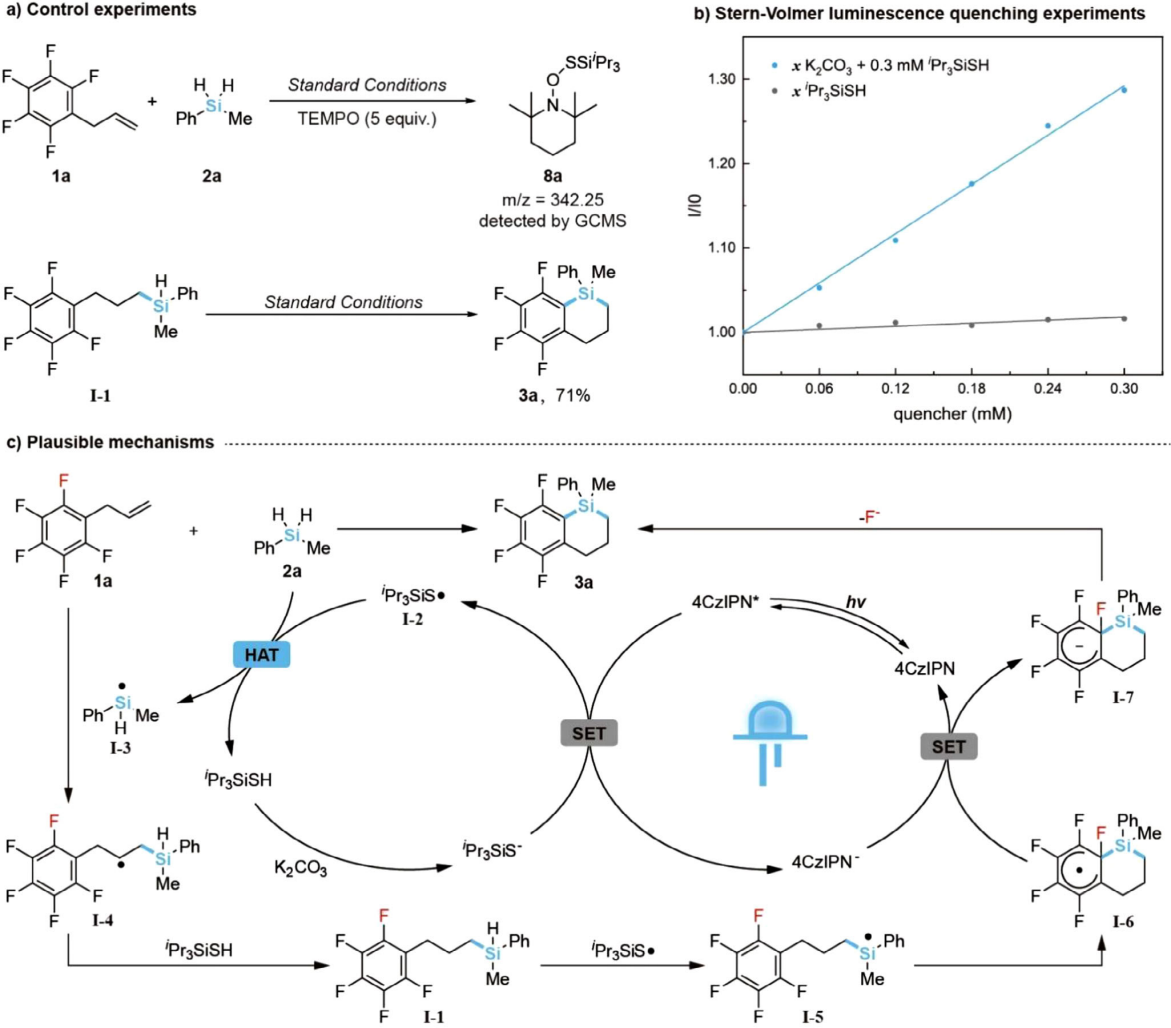
Proposed mechanisms of defluorosilacyclization with supporting evidence. a) Control experiments. b) Stern–Volmer luminescence quenching experiments. c) Plausible mechanisms.

To gain deeper insights into the two silacyclization reactions, a series of control experiments and spectroscopic studies were conducted. The mechanism of the defluorosilacyclization was investigated first. The addition of radical scavengers such as 2,2,6,6‐tetramethylpiperidoxyl (TEMPO) completely suppressed the standard defluorosilacyclization, with intermediate **8a** detected by GC‐MS, indicating the involvement of radical species and the presence of a thiyl radical (Figure 2a, top). Furthermore, subjecting the hydrosilylated **I‐1** to the standard conditions led to its smooth conversion to benzosilacycle **3a** in 71% yield, implying that **I‐1** serves as a key intermediate in the reaction pathway (Figure 2a, bottom). These findings strongly support a mechanism involving a hydrosilylation/cyclization cascade between **1a** and **2a**. Stern–Volmer quenching studies revealed that the excited‐state catalyst 4CzIPN* was weakly quenched by thiols, such as *^i^* Pr_3_SiSH, but not by allylbenzenes (e.g., **1a**) or silanes (e.g., **2a**). Notably, the quenching rate increased significantly with K_2_CO_3_, likely enhancing the single‐electron transfer (SET) process through thiol deprotonation (Figure 2b).^[^ ^39^, ^40^ ^]^

Based on these findings, a plausible mechanism for the defluorosilacyclization is proposed in Figure 2c. Initially, the excited‐state catalyst 4CzIPN* undergoes reductive quenching by the deprotonated thiol, generating thiyl radical **I‐2**. This radical then engages in a polarity‐matched HAT with the dihydrosilane to form silyl radical **I‐3** while regenerating the thiol. The silyl radical **I‐3** subsequently adds to the allylfluorobenzene, forming radical intermediate **I‐4**, and a subsequent HAT between **I‐4** and the thiol yields hydrosilylated intermediate **I‐1**. Another HAT by the thiol radical on intermediate **I‐1** generates a new silyl radical **I‐5**, which undergoes regioselective addition to the phenyl ring to form radical intermediate **I‐6**. Finally, the reduced photocatalyst 4CzIPN^−^ facilitates a single‐electron reduction of **I‐6**, furnishing silacycle product **3a** via intermediate **I‐7** after defluorination, thereby completing the catalytic cycle and regenerating the photocatalyst.

**Figure 3:**
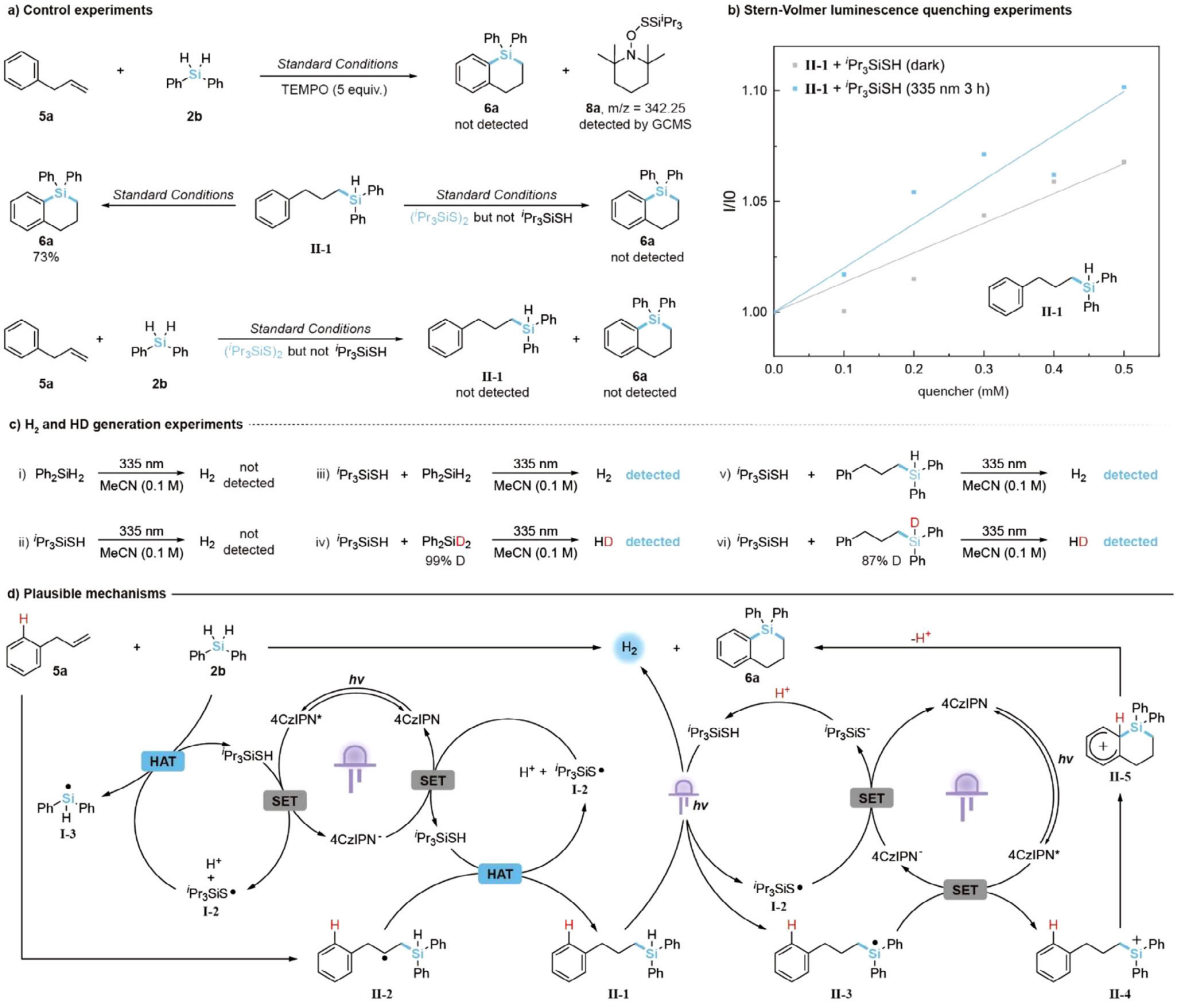
Proposed mechanisms for acceptorless dehydrosilacyclization with supporting evidence. a) Control experiments. b) Stern–Volmer luminescence quenching experiments. c) H_2_ and HD generation experiments. d) Plausible mechanisms.

We then conducted the mechanistic investigation of the acceptorless dehydrosilacyclization, focusing on its unique acceptorless hydrogen evolution process. Similar to defluorosilacyclization, radical inhibition experiments indicated the involvement of radical species and confirmed the presence of a thiyl radical (Figure 3a, top). Control experiments further established hydrosilylated **II‐1** as an intermediate in the reaction. Stern–Volmer quenching studies revealed that a mixture of intermediate **II‐1** and *^i^* Pr_3_SiSH effectively quenched the excited photocatalyst 4CzIPN*. Notably, irradiation of this mixture at 335 nm for 3 h improved the quenching efficiency, suggesting an enhanced SET process after 335 nm irradiation (Figure 3b). To identify the species involved in the hydrogen evolution oxidative coupling, we conducted additional control experiments. Disulfide compounds such as (*^i^* Pr_3_SiS)_2_ were unable to catalyze either intramolecular or intermolecular silacyclization (Figure 3a, middle and bottom). Neither the silane nor the thiol alone, when exposed to 335 nm light in CH_3_CN, produced hydrogen gas (Figure 3c, i and ii). In contrast, a mixture of Ph_2_SiH_2_ and *^i^* Pr_3_SiSH in CH_3_CN under identical conditions resulted in the generation of hydrogen gas (Figure 3c, iii). This catalyst‐free reaction was validated by subjecting Ph_2_SiD_2_ to the same conditions, leading to the detection of HD (Figure 3b, iv). Additionally, irradiation of either hydrosilylated intermediate **II‐1** or its deuterated counterpart with *^i^* Pr_3_SiSH at 335 nm led to the formation of H_2_ or HD, respectively (Figure 3c, v and vi). These results collectively indicate a light‐induced hydrogen evolution process between *i*Pr_3_SiSH and silanes (for light‐induced hydrogen evolution between hydrosilanes and other H‐donors, see Table S10). To explore the potential formation of a silyl–sulfide intermediate such as Ph(CH_2_)_3_Ph_2_Si–SSi*^i^* Pr_3_ during the hydrogen evolution process and serves as an active species for the subsequent transformations, we conducted both experimental analyses and density functional theory (DFT) calculations. However, experimental analyses, including crude nuclear magnetic resonance (NMR), high‐resolution mass spectrometry (HRMS), UV–vis spectroscopy, and in situ cyclic voltammetry (CV), failed to detect this species. Computational results also did not support the involvement of this species in the SET process with the excited catalyst 4CzIPN*, either through reductive or oxidative quenching,^[^ ^14^ ^]^ further ruling out its role in the reaction.

A plausible mechanism for the acceptorless dehydrosilacyclization is illustrated in Figure 3d. The reaction begins with the generation of thiyl radical **I‐2** via SET between the excited photocatalyst (4CzIPN*) and *^i^* Pr_3_SiSH, followed by deprotonation.^[^ ^38^, ^39^ ^]^ Thiyl radical **I‐2** then abstracts a hydrogen atom from silane **2b**, producing silyl radical **I‐3** and regenerating the thiol (*^i^* Pr_3_SiSH). Subsequently, the addition of silyl radical **I‐3** to allylbenzene **5a** forms the carbon‐centered radical **II‐2**, which undergoes HAT with *^i^* Pr_3_SiSH to form hydrosilylation intermediate **II‐1** and thiyl radical **I‐2**. The reduced photocatalyst (4CzIPN^−^) then facilitates a single‐electron reduction of **I‐2**, regenerating both the photocatalyst and *^i^* Pr_3_SiSH upon protonation. Under 335 nm light irradiation, intermediate **II‐1** likely undergoes a hydrogen evolution process with *^i^* Pr_3_SiSH, forming both the thiyl radical **I‐2** and the silyl radical **II‐3**. Reductive quenching of the excited‐state photocatalyst 4CzIPN* [*E*_1/2_(4CzIPN*/4CzIPN^‐^) = 1.35 V versus SCE (saturated calomel electrode)] by the silyl radical **II‐3** [*E*(**II‐3^+^**/**II‐3**)* *= −0.35 V versus SCE] generates silyl cation **II‐4**.^[^ ^41^ ^]^ This cation undergoes intramolecular cyclization to produce the aryl cation **II‐5**, which is then deprotonated to yield silacycle **6a**. Meanwhile, the thiyl radical **I‐2** is reduced by 4CzIPN^−^ via SET, completing the catalytic cycle with the regeneration of both the photocatalyst and *^i^* Pr_3_SiSH upon protonation.

## Conclusion

In summary, we have developed photo‐mediated silacyclizations of allylbenzenes with dihydrosilanes, enabling the efficient synthesis of six‐membered benzosilacycles from readily available starting materials, featuring an unprecedented wavelength‐dependent selective C─F or C─H functionalization. Additionally, this protocol allows for the facile synthesis of five‐ and seven‐membered benzosilacycles as well as benzogermacycles. Mechanistic investigations reveal that the reaction proceeds via a HAT‐facilitated intermolecular hydrosilylation, followed by an intramolecular cyclization. The merits of this method—including its metal‐free nature, redox‐ and atom‐economy, mild conditions, use of readily available starting materials, broad substrate scope, and wavelength‐controlled chemoselectivity—render it a highly attractive and practical approach for the synthesis of silacycles.

## Conflict of Interests

The authors declare no conflict of interest.

## Supporting information



Supporting Information

## Data Availability

The data that support the findings of this study are available in the Supporting Information of this article.
