# Supplementary material for: Photo‐Mediated Silacyclization by Wavelength‐Dependent Selective C─F or C─H Functionalization
Source: Angew Chem Int Ed Engl. 2025 Sep 1;64(43):e202512420. doi: 10.1002/anie.202512420 (PMC12535383; doi:10.1002/anie.202512420)

Supporting Information

©Wiley-VCH 2025

69451 Weinheim, Germany

Photo-mediated Silacyclization by Wavelength-Dependent Selective C−F or C−H Functionalization

Gan Wang,[‡] Ye Yuan,[‡] Chu Wang, Bingjie Ren, Hwee Ting Ang, Rong Zhou*, and Jie Wu*

Abstract: Silacycles have gained significant attention within the synthetic community due to their pivotal roles in medicinal chemistry and materials science. Despite recent advancements, the defluosilylation of aryl fluorides with hydrosilanes and the selective silylation of arenes without external oxidants remain challenging. Herein, we present a wavelength-dependent photo-mediated cascade silacyclization of allylbenzene derivatives with dihydrosilanes to efficiently construct six-membered benzosilacycles. By employing a synergistic system of an organophotocatalyst and a thiol-based hydrogen atom transfer catalyst, our approach exploits specific LED wavelengths (456 nm and 335 nm) to achieve selective C–F and C–H functionalization. This wavelength modulation enables the first successful defluorosilacyclization of *ortho*-fluoroallylbenzenes and facilitates an acceptorless dehydrosilacyclization, demonstrating precise site-selective functionalization. Mechanistic investigations reveal a HAT-facilitated intermolecular hydrosilylation followed by a wavelength-dependent, chemoselective intramolecular silacyclization cascade. Additionally, an unprecedented light-assisted hydrogen evolution process involving silane and thiol is uncovered within the cascade C–H silacyclization. This photomediated strategy offers a sustainable and versatile platform for the synthesis of valuable silacycle compounds, exhibiting broad functional group tolerance and precise wavelength-dependent chemoselectivity.

SUPPORTING INFORMATION

Table of Contents

I. General Methods 3

II. Synthesis of Starting Materials 4

III. General Procedures 6

IV. Detailed Optimization of Reaction Conditions 7

V. Mechanism Studies for Defluorosilacyclization 12

VI. Mechanism Studies for Dehydrogenative Silacyclization 15

VII. Computational Studies 24

VIII. Characteristic Data 37

IX. References 66

X. ^{1}H, ^{19}F and ^{13}C NMR Spectra for New Compounds 67

SUPPORTING INFORMATION

I. General Methods

All chemicals were obtained from commercial suppliers (Sigma Aldrich, TCI, BLD or Oakwood) and used without further purification unless otherwise noted. Solvents were freshly distilled before using. 50W 335 nm LED lights were purchased from Inwares Pte Ltd (Singapore). 40W 456 nm LED lights and 370 nm LED lights were purchased from Kessil. Silica gel column chromatography was carried out using silica Gel 60 (230–400 mesh). Analytical thin layer chromatography (TLC) was done using silica Gel (silica gel 60 GF254). TLC plates were analyzed by an exposure to ultraviolet (UV) light. ^{1}H NMR, ^{19}F NMR and ^{13}C NMR spectra were recorded on Bruker AV 400 MHz instrument at 400 MHz (^{1}H NMR), 101 MHz (^{13}C NMR), as well as 376 MHz (^{19}F NMR), or Bruker AV 600 MHz instrument at 600 MHz (^{1}H NMR), 151 MHz (^{13}C NMR), as well as 565 MHz (^{19}F NMR). Chemical shifts were calibrated using residual undeuterated solvent as an internal reference ($CDCl_3$: 7.26 ppm ^{1}H NMR, 77.0 ppm ^{13}C NMR). Multiplicity was indicated as follows: s (singlet), d (doublet), t (triplet), q (quartet), m (multiplet), dd (doublet of doublets), td (triplet of doublets), dt (doublet of triplets), bs (broad singlet), hept (heptet). High-resolution mass spectrometric data (HRMS) were obtained using Finnigan/MAT 95XL-T spectrometer. Gas Chromatography-Mass Spectrometry (GC-MS) was performed on Agilent 7820A with FID detection. Emission spectra were recorded in 1 cm path quartz cuvettes using an Edinburgh FS5 spectrofluorometer. Absorption spectra were recorded in 1 cm path quartz cuvettes using a Shimadzu UV-3600 Plus spectrofluorometer.

II. Synthesis of Starting Materials

II-1. *Preparation of fluorinated unsaturated benzene and allylbenzene*

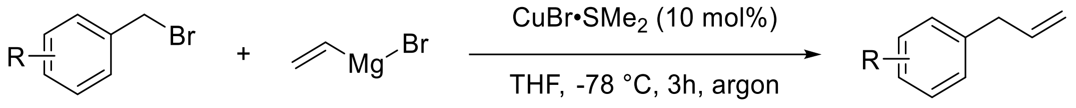

According to the previous reference,[1] to a suspension of CuBr·SMe_2 (41 mg, 0.20 mmol) in THF (1 mL), vinylmagnesiumbromide solution (6.03 mL, 4.0 mmol, 0.665 M in THF) was added at -78 °C and stirred for 30 min. After the desired transmetalation, a solution of benzyl bromide (2.00 mmol) in THF (1 mL) was added at -78 °C and stirred for 1.5 hr. The reaction solution was hydrolyzed with H_2O at -78 °C, then warmed to room temperature and extracted with pentane (3 x 10 mL). After treatment with sat. NaCl solution and extraction, the combined organic phase was dried over $MgSO_4$ and concentrated. A mixture of the allylbenzenes were obtained. The crude material was purified by flash chromatography to afford the products. Fluorinated-unsaturated benzenes for synthesis of products **4b**, **4g**, **4j**, and allylbenzene for synthesis of product **6s**, were prepared by this method and the analytical data of this alkene are in accordance with the reported data.

II-2. Preparation of dihydrosilanes

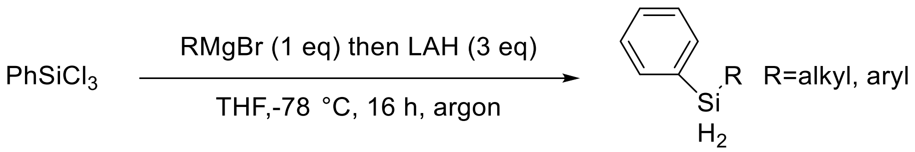

According to the previous reference,[2] In a 100 mL two-necked round-bottom flask, phenyltrichlorosilane (1.60 mL, 10 mmol) in THF (10 mL) was cooled to -78 °C under nitrogen atmosphere. A solution of Grignard reagent (10 mL, 1.0 M in THF) was added dropwise slowly over 60 min. Then the reaction was allowed to warm to room temperature and stirred for 12 h. The reaction was cooled down to –78 °C and lithium aluminium hydride (2.5 M in THF, 12 mL, 30 mmol) was added dropwise. After warming to room temperature, the solution was stirred for an additional 3 h. The reaction mixture was quenched with saturated NH_4Cl solution and extracted with Et_2O (25 mL × 3). The organic layer was washed with water (20 mL), brine (20 mL), dried over Na_2SO_4, filtered and concentrated. The crude product was purified by silica

column chromatography with hexanes to afford organosilianes. Dihydrosilane for synthesis of products **3c-3q**, **7a-7c**, were synthesized by this method and the analytical data of these dihydrosilane are in accordance with the reported data.[2]

II-3. Preparation of deuterated silane intermediates

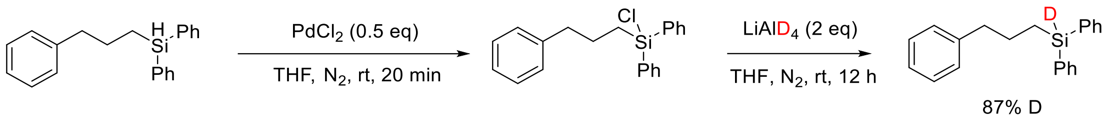

In a 100 mL two-nenecked round-bottom flask equipped with a stir bar, intermediate **II-1** (1 mmol) and $PdCl_2$ (0.5 equiv) were dissolved in THF (10 mL) under a nitrogen atmosphere at room temperature. After stirring for 20 min, the reaction mixture was filtered through a short layer of silica gel to remove residual $PdCl_2$. The filtrate was transferred to another 100 mL two-necked round-bottom flask, and $LiAlD_4$ (2 equiv) was added portionwise under N_2 at room temperature. The reaction was stirred for 12 h, then quenched with saturated aqueous NH_4Cl solution. The mixture was extracted with EtOAc (3 × 25 mL), and the combined organic layers were washed with H_2O (20 mL) and brine (20 mL), dried over anhydrous Na_2SO_4, filtered, and concentrated. The crude product was purified by flash column chromatography to afford the deuterated silane intermediate as a colorless oil. Deuterium incorporation (87%) was determined by ^{1}H NMR spectroscopy.

III. General Procedures

III-1. General procedure for defluorosilacyclization

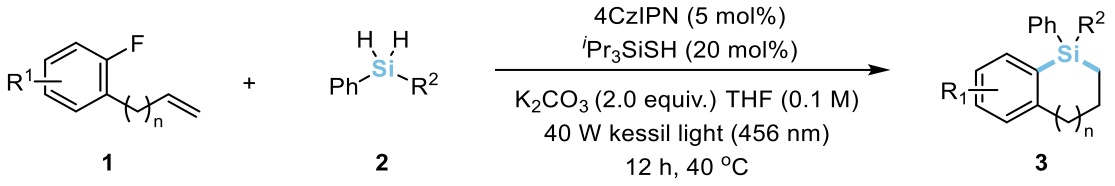

To a flame-dried 10 mL reaction vial with a magnetic stir bar was added 4CzIPN (0.01 mmol, 5.0 mol%), triisopropylsilanethiol ${}^{i}Pr_3SiSH$ (0.04 mmol, 20.0 mol%.), potassium carbonate K_2CO_3 (0.40 mol, 2.0 equiv.), fluorinated-unsaturated benzene **1** (0.2 mmol, 1.0 equiv.), dihydrosilane **2** (0.30 mmol, 1.5 equiv.) and THF (2 mL) in the glovebox. The reaction mixture was irradiated with a 40 W blue LED for 12 h at 40 ℃. After stirred for 12 hours, the reaction mixture was concentrated in vacuo and then quenched with water, extracted with ethyl acetate. The combined organic layers were dried with $MgSO_4$, filtered and concentrated in vacuo. The crude material was purified by flash chromatography to afford the products.

III-2. General procedure for dehydrogenative silacyclization

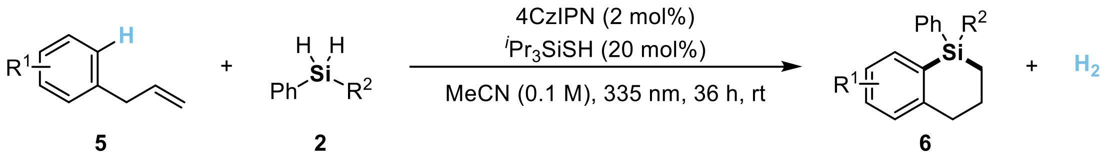

To a flame-dried 10 mL reaction vial with a magnetic stir bar was added 4CzIPN (0.004 mmol, 2.0 mol%), triisopropylsilanethiol ${}^{i}Pr_3SiSH$ (0.04 mmol, 20.0 mol%.), allylbenzene **5** (0.2 mmol, 1.0 equiv.), dihydrosilane **2** (0.30 mmol, 1.5 equiv.) and CH_3CN (2 mL) in the glovebox. The reaction mixture was irradiated with 335 nm light for 36 h at room temperature. After stirred for 36 hours, the reaction mixture was concentrated in vacuo and then quenched with water, extracted with ethyl acetate. The combined organic layers were dried with $MgSO_4$, filtered and concentrated in vacuo. The crude material was purified by flash chromatography to afford the products.

SUPPORTING INFORMATION

IV. Detailed Optimization of Reaction Conditions

Table S1. Optimization of defluorosilacyclization[a]

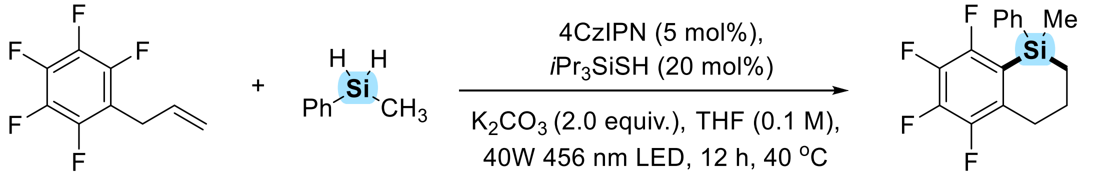

1a **2a** **3a**

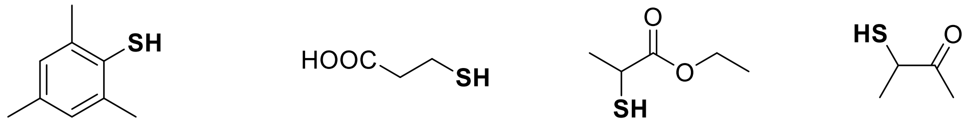

SH1 **SH2** **SH3** **SH4**

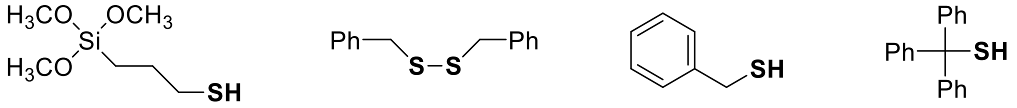

SH5 **SH6** **SH7** **SH8**

Entery	Deviation	Yield of 3a[b]
1	**None**	86
2	eosin Y instead of **4CzIPN**	56
3	$[Ir(dF(CF_3)ppy)_2(dtbbpy)]PF_6$ instead of **4CzIPN**	5
4	$[Ir(ppy)_2(dtbbpy)]PF_6$ instead of **4CzIPN**	0
5	*fac*-$Ir(ppy)_3$ instead of **4CzIPN**	0
6	other RSH instead of **$^iPr_3SiSH$**	0-22
7	other solvent instead of **THF**	0-15
8	K_2HPO_4 instead of **K_2CO_3**	63
9	DBU instead of **K_2CO_3**	31
10	**2 mol%** of 4CzIPN	71
11	**24 h** reaction time	81
12	427 nm light instead of **456 nm**	79
13	no photocatalyst or no light or no RSH	0

[a]Reaction conditions: **1a** (0.10 mmol), **2a** (0.15 mmol), 4CzIPN (5 mol%), $^iPr_3SiSH$ (20 mol%), K_2CO_3 (2 equiv.) in THF (0.1 M) at 40 °C under 40 W 456 nm LED (456 nm) for 12 h. [b]Isolated yields

Table S2. Optimization of dehydrogenative silacyclization [a]

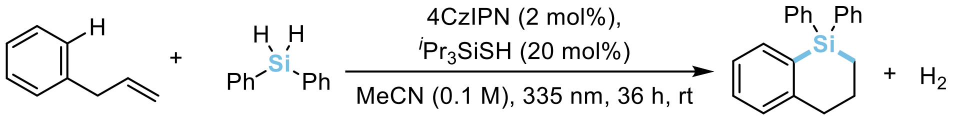

5a **2b** **6a**

Entry	Deviation	Yield of 6a[b]
1	none	73
2	other PC instead of 4CzIPN	0-19
3	other RSH instead of $^{i}Pr_3SiSH$	0-12
4	other solvent instead of MeCN	22-68
5	DBU (1.5 equiv.) as an additive	19
6	K_2CO_3 (1.5 equiv.) as an additive	24
7	AcOH (20 mol%) as an additive	17
8	PhCOOH (20 mol%) as an additive	16
9	5 mol% of 4CzIPN	39
10	456 nm light instead of 335 nm	37
11	390 nm light instead of 335 nm	61
12	24 h reaction time	64
13	no photocatalyst or no light or no RSH	0

[a]Reaction conditions: 5a (0.10 mmol), 2b (0.15 mmol), 4CzIPN (5 mol%), $^{i}Pr_3SiSH$ (20 mol%), in MeCN (0.1 M) under 335 nm light irradiation at rt for 36 h. [b]Isolated yields

Table S3. Screening of photocatalysts for dehydrogenative silacyclization

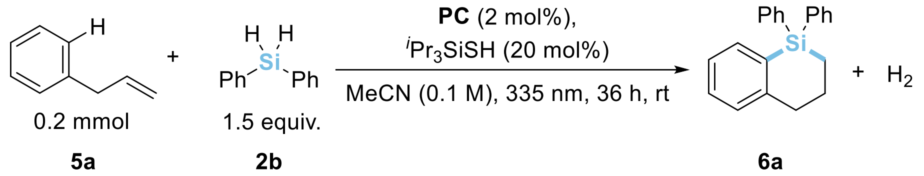

Entery	Deviation	Yield of **6a**[a]
1	$[Ru(bpy)_3](PF_6)_2$	N.D
2	*fac*-$Ir(ppy)_3$	N.D
3	$[Ir(ppy)_2(dtbbpy)]PF_6$	N.D
4	$[IrdF(CF_3)ppy_2(dtbbpy)]PF_6$	19
5	eosin Y	N.D
6	$[Ru(bpz)_3](PF_6)_2$	N.D

[a]: isolated yields

Table S4. Screening of solvents for dehydrogenative silacyclization

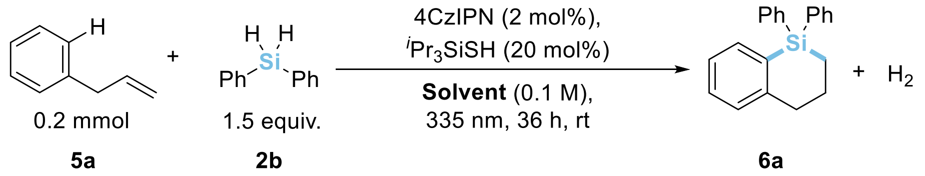

Entery	Deviation	Yield of **6a**[a]
1	acetone	N.D
2	^{t}BuCN	68
3	DMSO	14
4	DMF	15
5	toluene	6
6	DCM	22
7	1,4-dioxane	29
8	methanol	N.D

[a]: isolated yields

Table S5. Screening of HAT reagents for dehydrogenative silacyclization

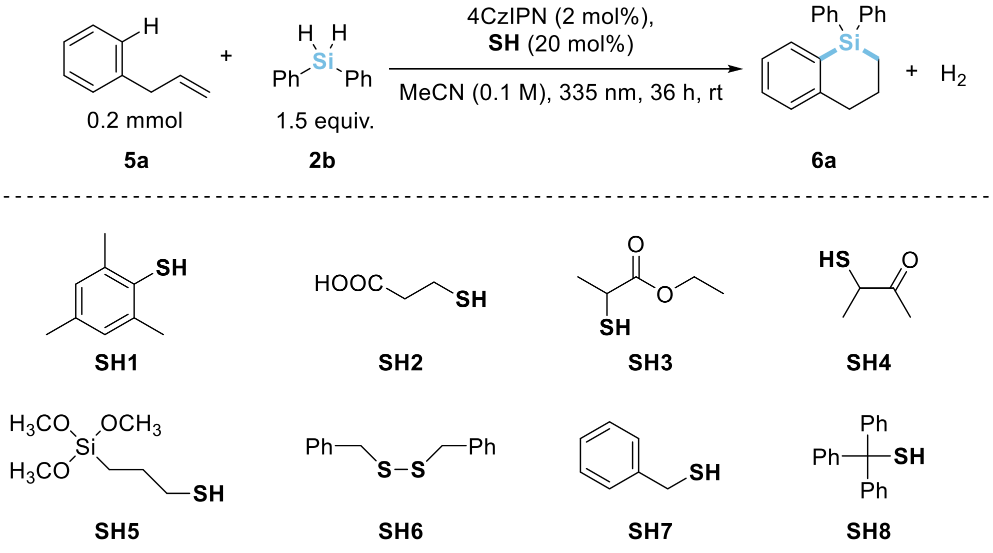

Entery	Deviation	Yield of **6a**[a]
1	**SH1**	N.D
2	**SH2**	N.D
3	**SH3**	N.D
4	**SH4**	N.D
5	**SH5**	N.D
6	**SH6**	12
7	**SH7**	N.D
8	**SH8**	N.D

[a]: isolated yields

Table S6. Screening of additives for dehydrogenative silacyclization

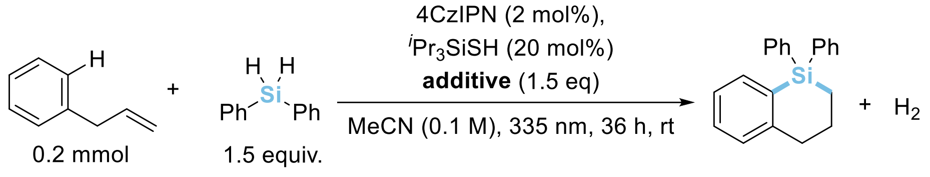

Entery	Deviation	Yield of **6a**[a]
1	K_2HPO_4	33
2	KH_2PO_4	15
3	DBU	19
4	K_2CO_3	24
5	NaOH	16
6	DABCO	nd
7	Na_2CO_3	21
8	$^{t}BuOK$	nd
9	DMAP	4
10	Cs_2CO_3	24
11	AcOH (20 mol%)	17
12	$PhCO_2H$ (20 mol%)	16

[a]: isolated yields

Table S7. Screening of concentration for dehydrogenative silacyclization

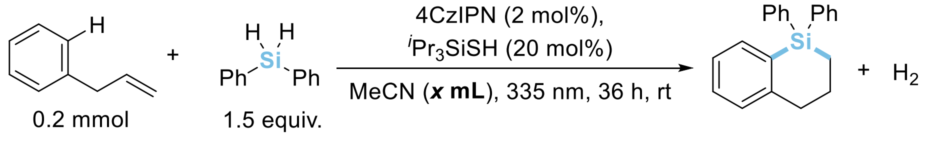

Entery	Deviation	Yield of **6a**[a]
1	0.5 mL	62
2	2 mL	64
3	4 mL	57
4	8 mL	44

[a]: isolated yields

Table S8. Screening of light sources for dehydrogenative silacyclization

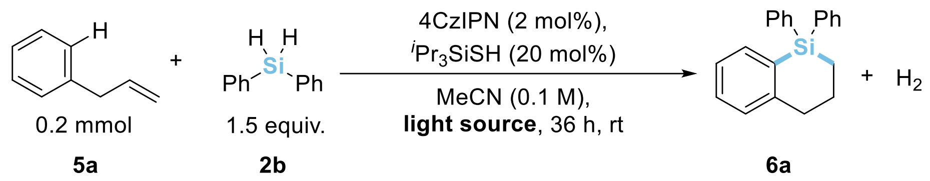

Entery	Deviation	Yield of **6a**[a]
1	456 nm	37
2	427 nm	43
3	390 nm	61
4	white light	10

[a]: isolated yields

V. Mechanism Studies for Defluorosilacyclization

V-1. Radical scavenger

The reaction was inhibited by the radical scavenger, 2,2,6,6-tetramethyl-1-piperidinyloxy (TEMPO), thus suggesting a radical process. Intermediate **8a** was detected by GC-MS.

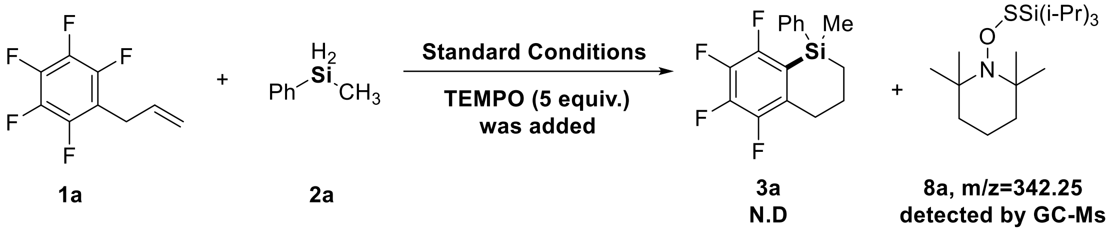

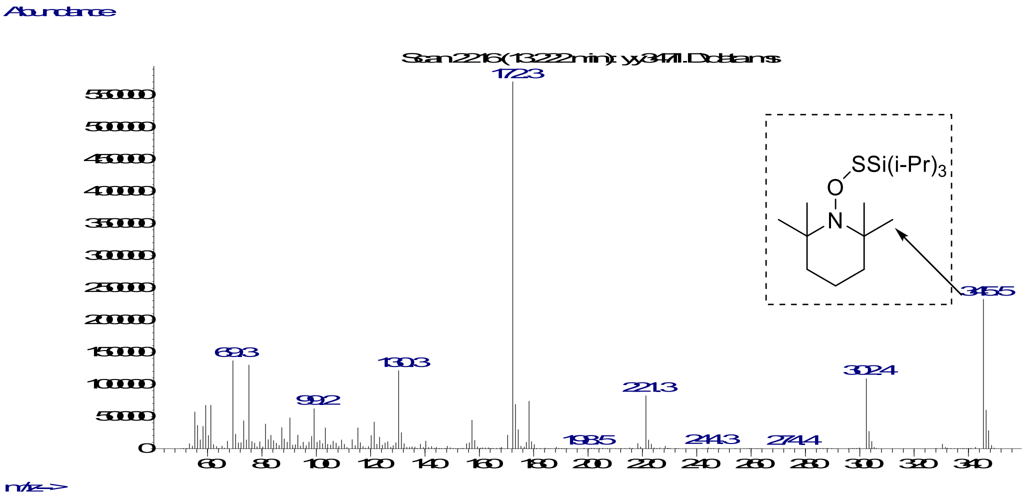

Figure S1. Detection of **8a** by GC-MS in radical scavenger experiments

V-2. Intermediate trapping experiments

According to the general procedure, allypentafluorobenzene **1a** (83.2 mg, 0.40 mmol, 1.0 equiv.), methylphenylsilane **2a** (146.6 mg, 1.2 mmol, 3.0 equiv.), $^{i}Pr_3$SiSH (15.2 mg, 0.08 mmol, 20 mol%), 4CzIPN (3.15 mg, 0.004 mmol, 1 mol%), K_2HPO_4 (55.2 mg, 0.40 mmol, 1.0 equiv.) and THF (4 mL) were used. After irradiated under the 456 nm light 12 h, the resulting mixture was concentrated under vacuum and the residue was separated by flash chromatography (PE) to give the intermediate **I-1** as a slightly yellow oil (96.8 mg, 74%).

Intermediate **I-1** (66.0 mg, 0.2 mmol, 1.0 equiv.), $^{i}Pr_3$SiSH (7.6 mg, 0.04 mmol, 20 mol%), 4CzIPN (7.9 mg, 0.01 mmol, 5 mol%), K_2CO_3 (55.2 mg, 0.40 mmol, 2.0 equiv.)

and THF (2 mL) were used for the second step silacyclization. After irradiated under the 456 nm light 12 h, the result mixture was concentrated under vacuum and the product was isolated by flash chromatography as a colorless oil **3a** (44.0 mg, 71%). The formation of the intermediate **I-1** was generated under the reaction condition shown in Fig. S2, confirming its generation during the reaction. Consistently, the treatment of **I-1** with the standard condition without methylphenylsilane **2a** led to the formation of silacyclization products in high yield. These control experiments collectively support that defluorosilacyclization involves a radical addition process and an intramolecular cyclization reaction.

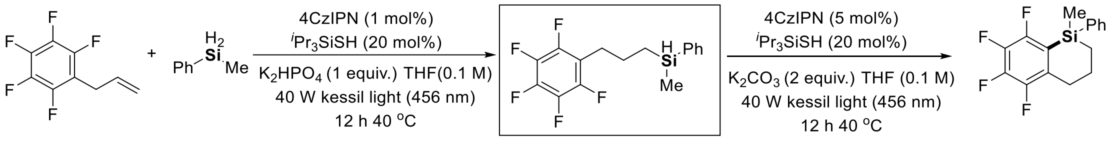

Figure S2. Evidence of **I-1** being the key intermediate for defluorosilacyclization

V-3. Stern-Volmer fluorescence quenching experiments

Emission intensities were recorded using a Perkin Elmer LS50 Luminescence spectrometer. All 4CzIPN solutions were excited at 440 nm and the emission intensity at 556 nm was observed. In a typical experiment, a 3.0×10^{-5} M solution of 4CzIPN in THF was added to the appropriate amount of quencher in a screw top 1.0 cm quartz cuvette. After degassing with a stream of nitrogen for 10 minutes, the emission spectrum of the sample was collected. The result revealing that with base K_2CO_3, the HAT reagent $^{i}Pr_3$SiSH exhibit the obvious enhanced efficiency in quenching 4CzIPN* fluorescence. This observation strongly suggests the HAT reagent $^{i}Pr_3$SiSH take part in a reductive quenching pathway in the deactivation of the excited photocatalyst.

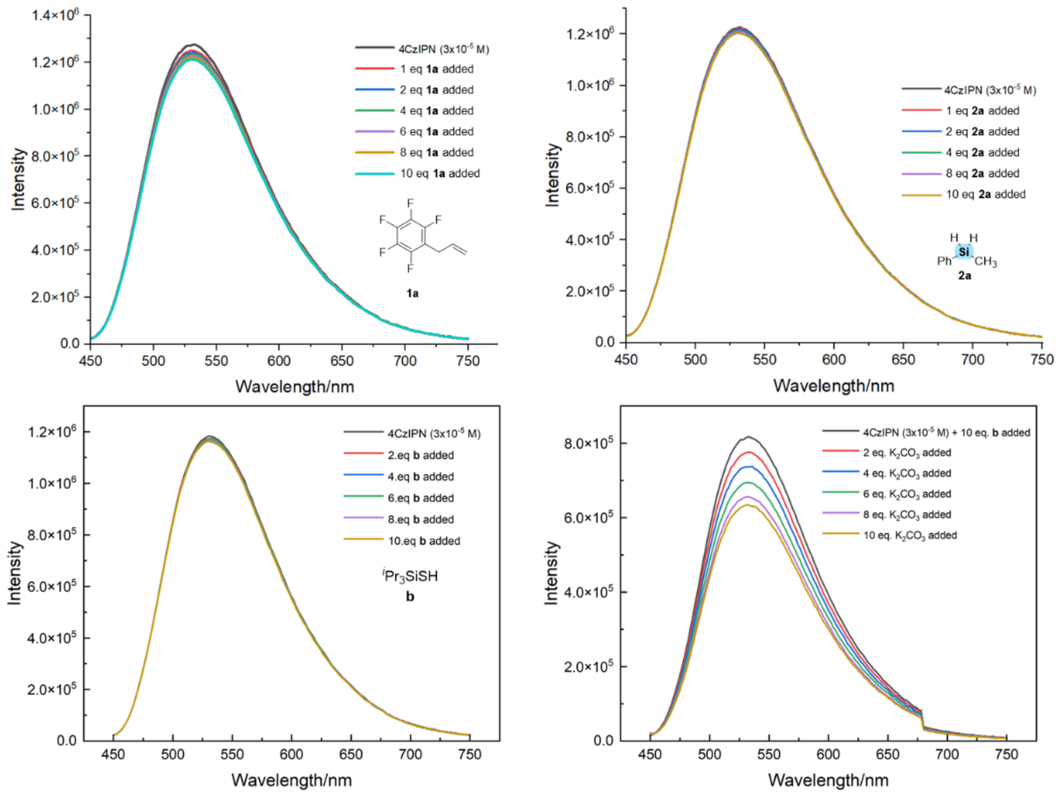

Figure S3. Stern-Volmer fluorescence quenching experiments for defluorosilacyclization

V-4. Time-monitoring experiments

Time-course experiments were conducted under standard conditions. In situ NMR studies confirmed the formation of **I-1** and its subsequent conversion to product **3a**. Critically, no silylation of aromatic ring (including three regioisomers) were detected by GC-MS or NMR. These observations strongly support our conclusion that the cascade reaction initiates with hydrosilylation of the terminal alkene rather than the aromatic ring.

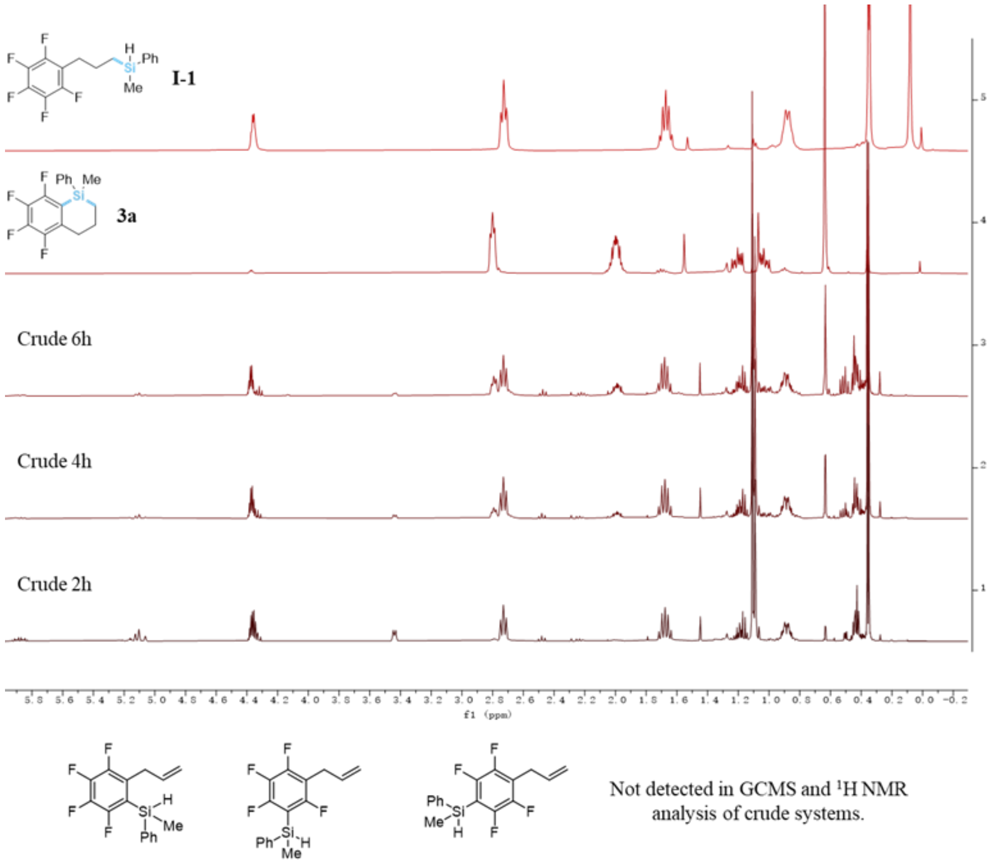

Figure S4. Time-monitoring experiments for defluorosilacyclization

VI. Mechanism Studies for Dehydrogenative Silacyclization

VI-1. Detection of H_2 gas

A 10 mL microwave tube equipped with a penetrable septum and a magnetic stir bar was charged with 4CzIPN (0.004 mmol, 2.0 mol%), triisopropylsilanethiol $^{i}Pr_3SiSH$ (0.04 mmol, 20.0 mol%.), allylbenzene **5** (0.2 mmol, 1.0 equiv.), dihydrosilane **2b** (0.30 mmol, 1.5 equiv.) and CH_3CN (2 mL) and bubbled with argon for 15 min. Then, the reaction tube was exposed to a 335 nm light for 36 h at room temperature. The upper atmosphere (8.0 mL) was analyzed by GC to confirm the generation of H_2 gas.

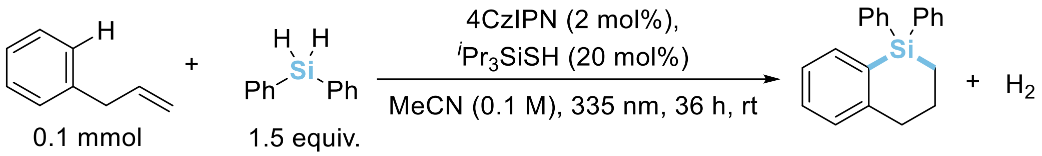

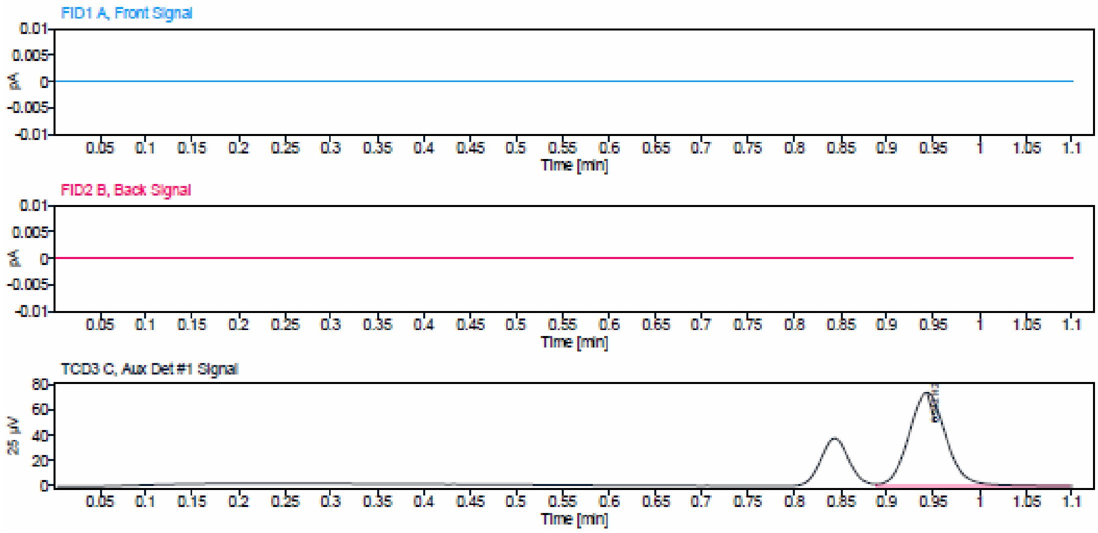

Figure S5. Detection of H_2 by GC in dehydrogenative silacyclization.

VI-2. Radical scavenger

Radical inhibition experiments were performed to investigate the mechanism of the dehydrogenative silacyclization reaction. The target product **9-a** also could not be generated when 2,2,6,6-Tetramethylpiperidinooxy (TEMPO) was added to the reaction system, which suggested that the reaction involved a radical process. Notably,

intermediate **8a** was also detected by ESI-MS, which suggested that thiyl radicals might be generated.

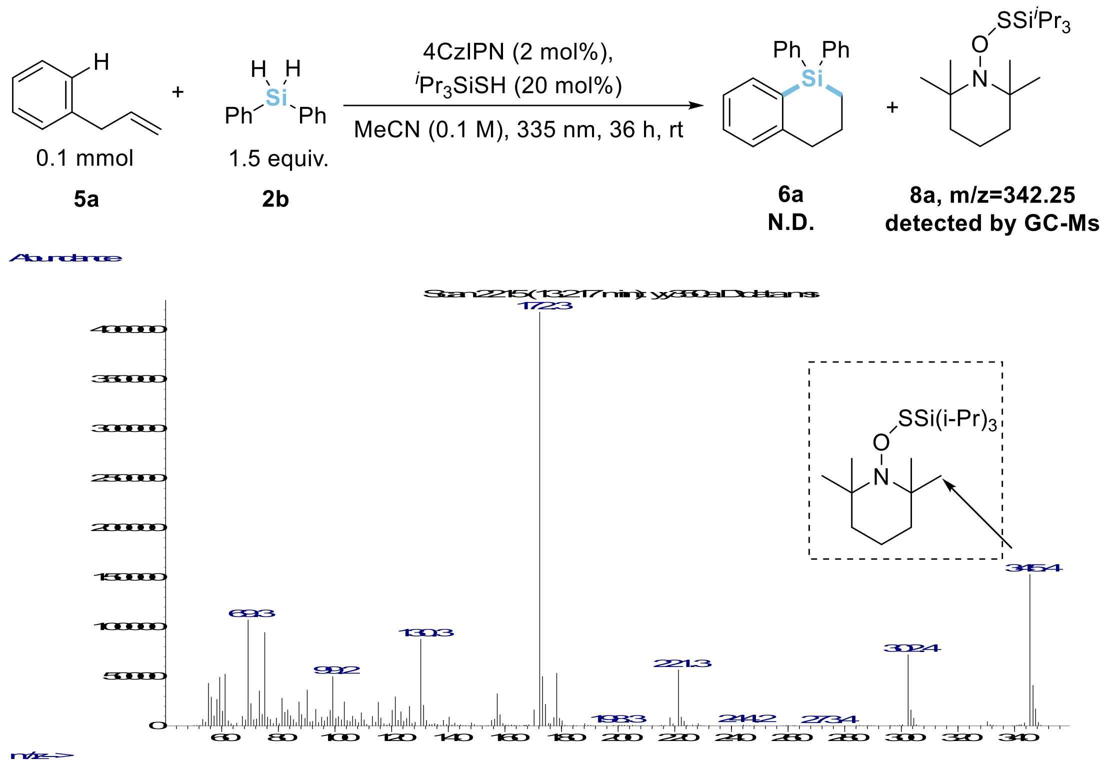

Figure S6. Detection of **8a** by GC-MS in radical scavenger experiments

VI-3. Intermediate trapping experiments

According to the general procedure, allylbenzene **5a** (47.2 mg, 0.40 mmol, 1.0 equiv.), diphenylsilane **2b** (120.4 mg, 0.60 mmol, 1.5 equiv.), $^{i}Pr_3SiSH$ (15.2 mg, 0.08 mmol, 20 mol%), 4CzIPN (15.78 mg, 0.02 mmol, 5 mol%), K_2CO_3 (110.4 mg, 0.80 mmol, 2.0 equiv.) and THF (4 mL) were used. After irradiated under the 456 nm light 12 h, the resulting mixture was concentrated under vacuum and the residue was separated by flash chromatography to give the intermediate **II-1** as a colorless oil (102.7 mg, 85%).

Intermediate **II-1** (60.4 mg, 0.2 mmol, 1.0 equiv.), $^{i}Pr_3SiSH$ (7.6 mg, 0.04 mmol, 20 mol%), 4CzIPN (3.2 mg, 0.004 mmol), 2 mol% and MeCN (2 mL) were used. After irradiated under the 335 nm light 36 h, the result mixture was concentrated under vacuum and the product was isolated by flash chromatography as a colorless oil **6a** (43.8 mg, 73%).

The treatment of **II-1** with the standard condition without diphenylsilane **2b** led to the formation of dehydrogenative silacyclization products **6a** in high yield. These control experiments collectively support that the dehydogenative silacyclization involves a radical addition process and an intramolecular cyclization reaction.

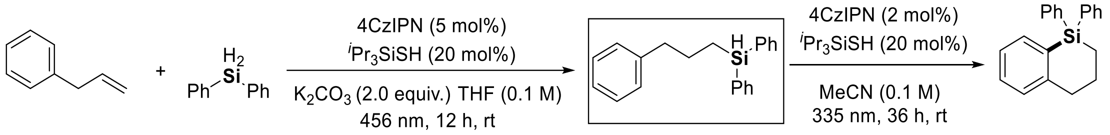

Figure S7. Evidence of **II-1** being the key intermediate for deydrogenatvie silacyclization

VI-4. Control experiments regarding the second step dehydrogenative process

Since the dehydrogenative process is only associated with the second step, control experiments were conducted using intermediate **II-1**. When 2,2,6,6-Tetramethylpiperidinooxy (TEMPO) was added to the reaction system, no product **6a** was detected, indicating a radical-mediated pathway. Notably, in absence of photocatalyst, trace amounts of **6a** were detected when shining light on the mixture of **II-1**and $^i Pr_3$SiSH. This observation prompted investigation into the interaction between $^i Pr_3$SiSH and silane species under 335 nm light.

Table S9. Control experiments regarding the second step dehydrogenative process

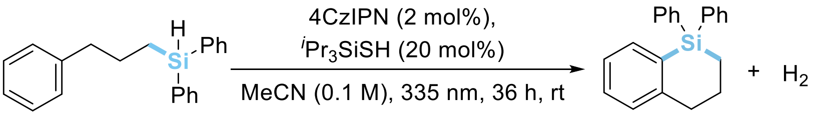

Entery	Deviation	Yield of **6a**
1	-	73%[a]
2	no 4CzIPN	< 5%[b]
3	no ^{i}Pr$_3$SiSH	N.D
4	no light	N.D
5	no 4CzIPN and no light	N.D
6	TEMPO (5 eq) added	N.D

[a]: isolated yield, [b]: NMR yield

VI-5. H_2 and HD generation experiments

A 10 mL microwave tube equipped with a penetrable septum and a magnetic stir bar was charged with triisopropylsilanethiol $^{i}Pr_3$SiSH (0.1 mmol), dihydrosilane **2b** (0.1 mmol) and CH_3CN (1 mL) and bubbled with argon for 15 min. Then, the reaction tube was exposed to a 335 nm light for 12 h at room temperature. The upper atmosphere (9.0 mL) was analyzed by GC to confirm the generation of H_2 gas. The generation of H_2 is not observed when $^{i}Pr_3$SiSH or **2b** are individually exposed to 335 nm irradiation. However, when a mixture of $^{i}Pr_3$SiSH and **2b** is irradiated at 335 nm, a distinct signal for H_2 evolution is detected.

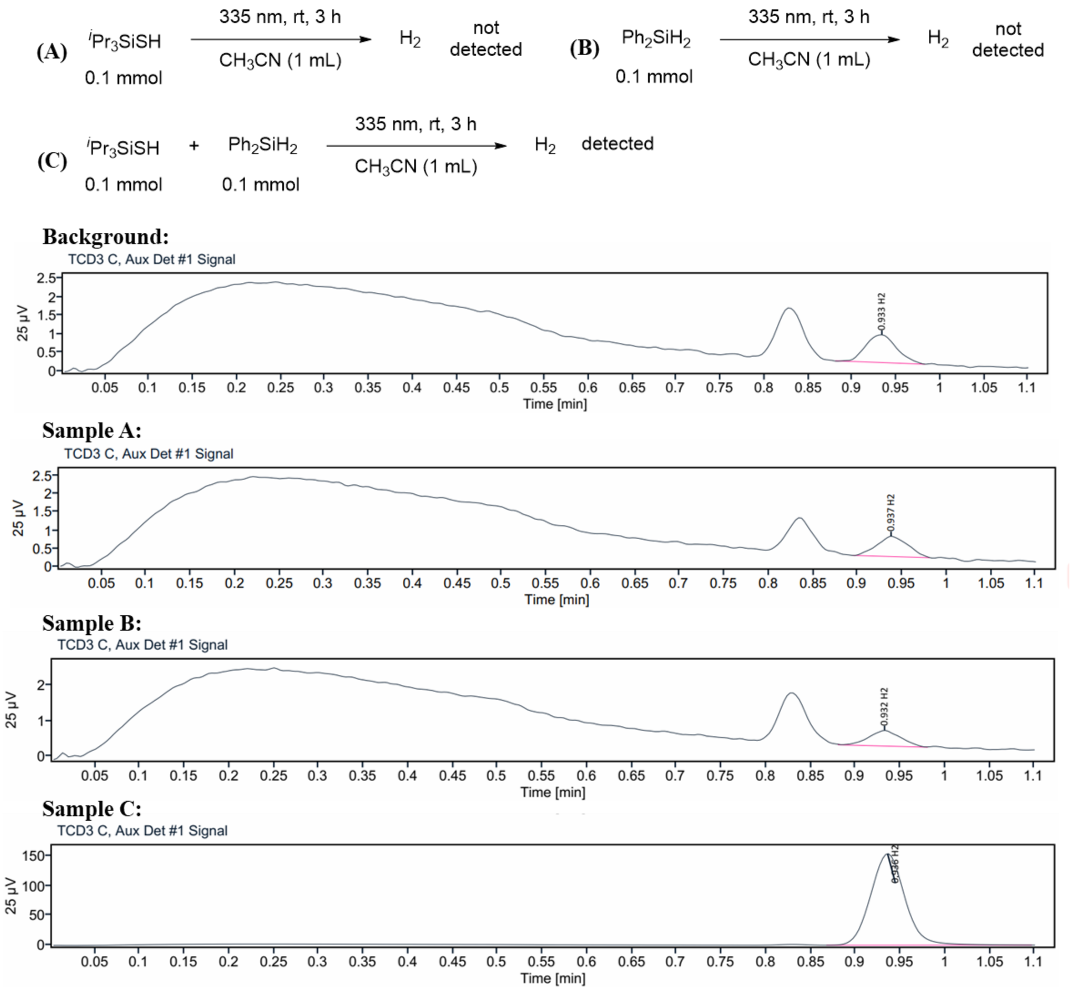

Figure S8. H_2 detection experiments by GC

A 10 mL microwave tube equipped with a penetrable septum and a magnetic stir bar was charged with triisopropylsilanethiol $^{i}Pr_3$SiSH (0.1 mmol), silane species (0.1 mmol) and CH_3CN (1 mL) and bubbled with argon for 15 min. Then, the reaction tube was exposed to a 335 nm light for 5 h at room temperature. The upper atmosphere (9.0 mL) was analyzed by differential electrochemical mass spectroscopy (DEMS). H_2 (*m/z* =2,

black), HD (m/z =3, red), D_2 (m/z =4, blue) were collected using a Hiden HPR-40 DEMS Mass Spectrometer with a tube connected to the upper atmosphere in microwave tube. The results show that when using deuterated silane, the generation of H_2 will change to generation of HD. Notably, the signal of H_2 severely dropped and no D_2 signal is detected in all groups. These results indicate that the dehydrogenative process is happening via a "cross-coupling" fashion (thiol and silane each contributes one H atom).

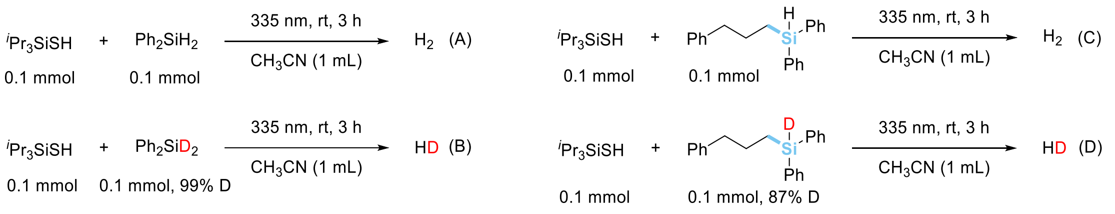

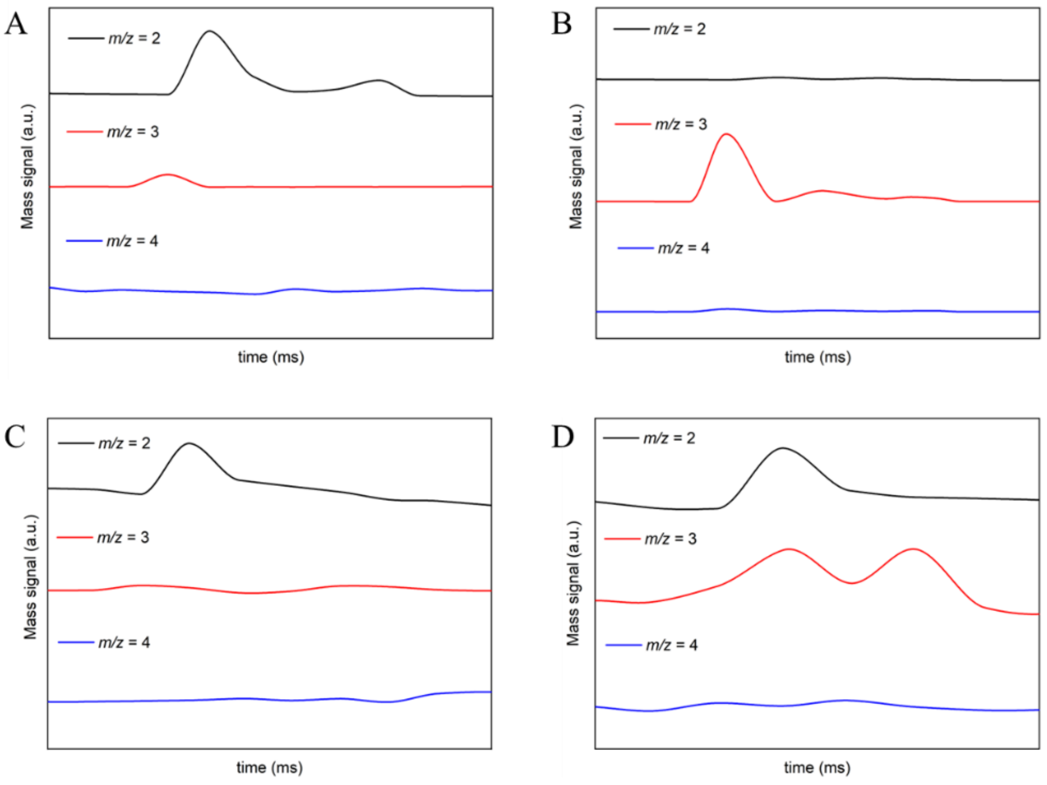

Figure S9. H_2 and HD detection experiments by DEMS

VI-6. Stern-Volmer fluorescence quenching experiments

Emission intensities were recorded using a Perkin Elmer LS50 Luminescence spectrometer. All 4CzIPN solutions were excited at 390 nm and the emission intensity at 565 nm was observed. In a typical experiment, a 5.0×10^{-5} M solution of 4CzIPN in

MeCN was added the appropriate amount of quencher solution inside a screw top 1.0 cm quartz cuvette with nitrogen protection. The emission spectrum of the sample was collected. The result revealing that the mixed solution of ${}^{i}Pr_3$SiSH and intermediate **II-2** in MeCN can better quench 4CzIPN* after exposure under 335 nm light for 3 h. These results reveal that beside generation of H_2, ${}^{i}Pr_3$SiSH and II-1 under 335 nm light is likely to also generate radical species (**I-2** and **II-3**) in the solution so that can better quench 4CzIPN*.

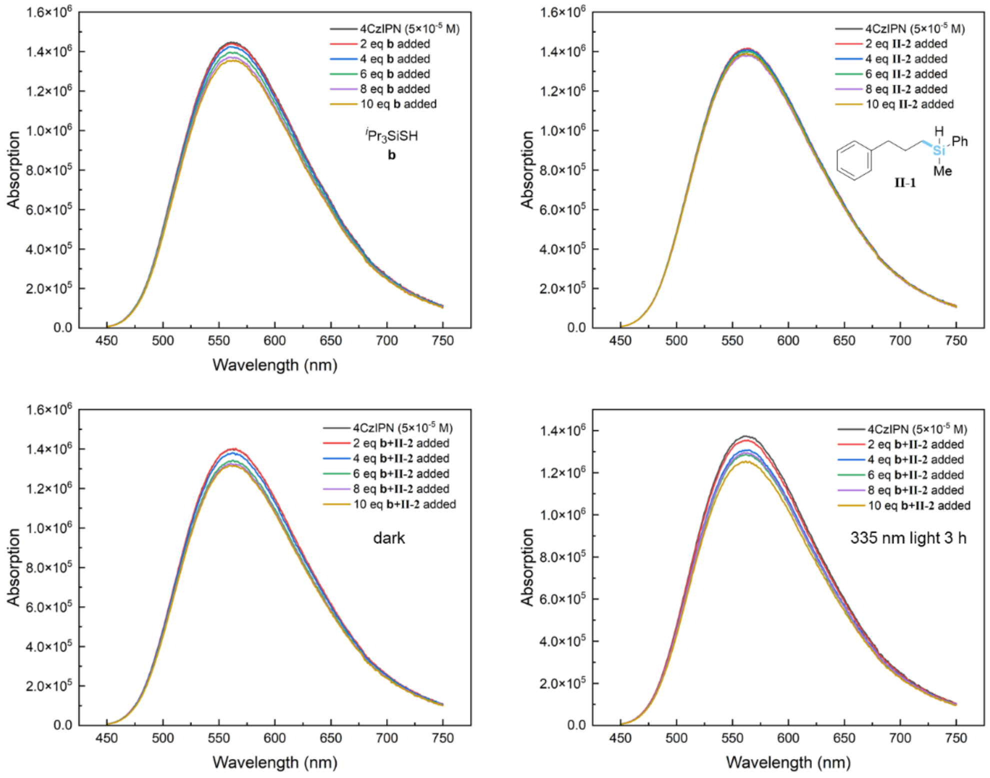

Figure S10. Stern-Volmer fluorescence quenching experiments for dehydrogenative silacyclization

VI-7. Attempt to capture possibly $Ph(CH_2)_3Ph_2Si–SSi^{i}Pr_3$ intermediate

Based on the results of H_2 and HD detection experiments, we hypothesized that, in addition to HD, another cross-coupling product, such as $Ph(CH_2)_3Ph_2Si–SSi^{i}Pr_3$, might form as an intermediate. To probe this, we compared the crude NMR (Table S8, entry 2) with the standard reference, but no new peaks corresponding to the proposed intermediate were observed.

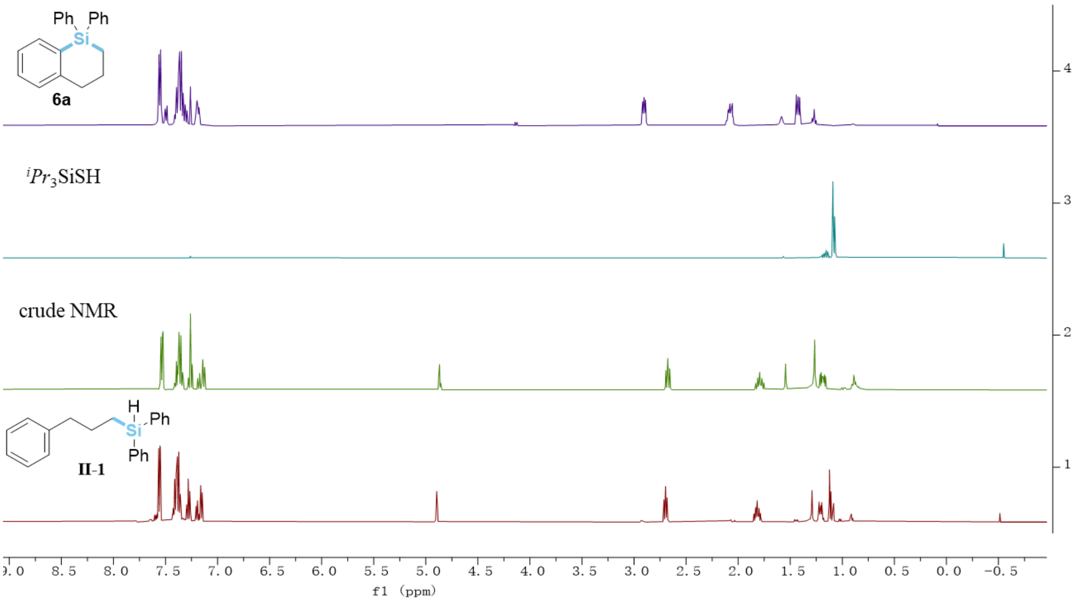

Figure S11. NMR study to identify Ph(CH$_2$)$_3$Ph$_2$Si–SSi^{i}Pr$_3$

Subsequently, we attempted to detect the formation of Ph(CH$_2$)$_3$Ph$_2$Si–SSi^{i}Pr$_3$ and other potential homo-coupling products by HRMS (high-resolution mass spectrometry). However, none of the structures shown below were observed in the mass spectra.

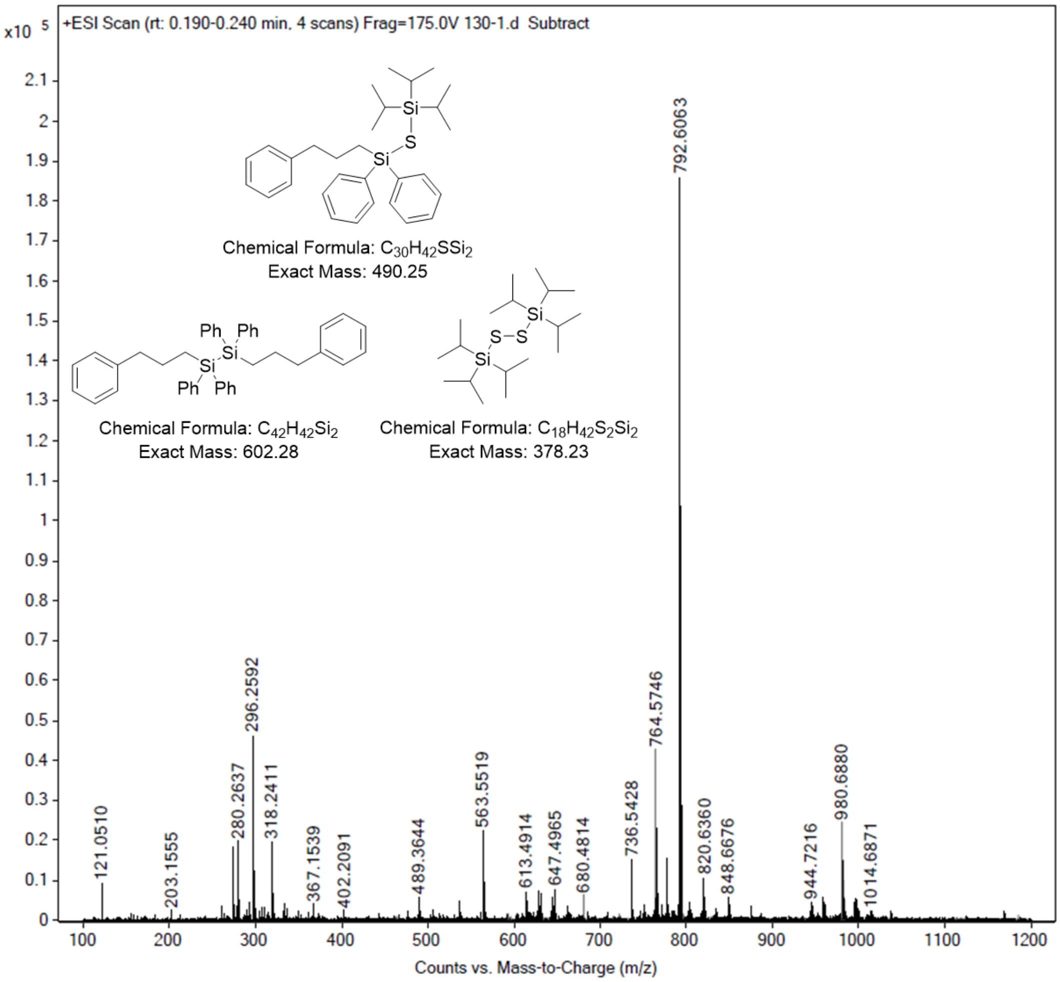

Figure S12. HRMS study to identify $Ph(CH_2)_3Ph_2Si–SSi^iPr_3$

Next, we compared the UV-vis spectrum of $^iPr_3SiSH$, **II-2**, and mixture before and after 335 nm irradiation. However, no new absorption band was detected.

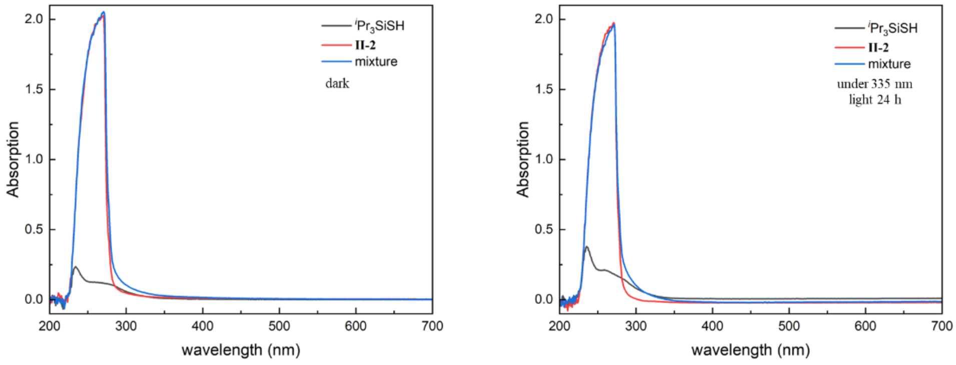

Figure S13. UV-vis study to identify $Ph(CH_2)_3Ph_2Si–SSi^iPr_3$, substances dissolved in MeCN (10^{-3} M)

Cyclic voltammetry was conducted on a VersaSTAT 3 Potentiostat Galvanostat from Princeton Applied Research using a 3-electrode cell configuration. A glassy carbon working electrode was employed alongside a platinum wire counter electrode and a Ag/AgCl reference electrode. 0.1 M s $^{i}Pr_3$SiSH, **II-2**, and mixture were freshly prepared in MeCN along with 0.1 M of tetrabutylammonium hexafluorophosphate as supporting electrolyte and were degassed by bubbling argon prior to measurements. The solutions were examined at a scan rate of 100 mV s^{-1}. The mixture solution is tested after 335 nm irradiation for 4 h, the light is on throughout the measurement. No new reductive peaks were observed.

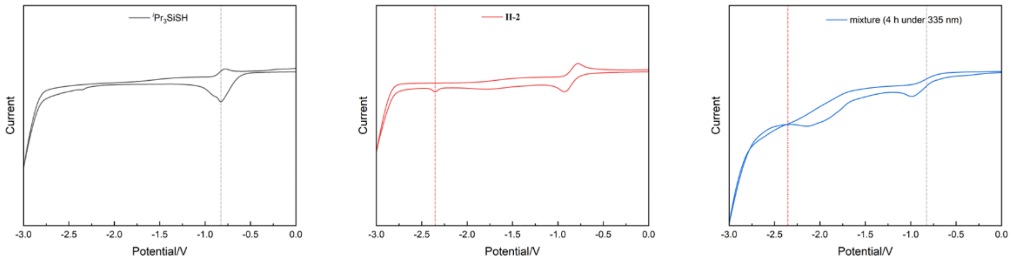

Figure S14. *In situ* CV study to identify Ph$(CH_2)_3Ph_2$Si–SSi$^{i}Pr_3$

VI-8. Photoreactions of hydrosilanes with other H-donors

A 10 mL microwave tube equipped with a penetrable septum and a magnetic stir bar was charged with H-donors **1-6** (0.1 mmol), hydrosilane (0.1 mmol) and CH_3CN (1 mL) and bubbled with argon for 15 min. Then, the reaction tube was exposed to a 335 nm light for 6 h at room temperature. The upper atmosphere (9.0 mL) was analyzed by GC to test the generation of H_2 gas. The experimental results demonstrate that multiple classes of hydrogen donors, including thiols, alcohols, carboxylic acids, and amines, can effectively participate in hydrogen (H_2) generation with hydrosilane compounds under 335 nm irradiation.

SUPPORTING INFORMATION

Table S10. Photoreactions between H-donors and hydrosilanes

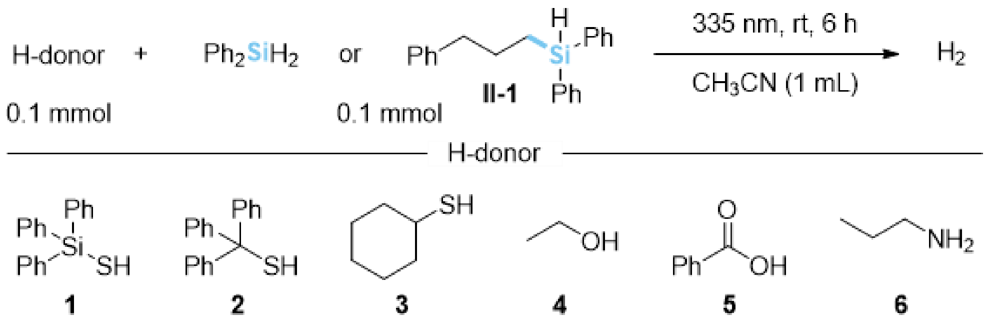

H-donor	H_2 (detected by GC) with Ph_2SiH_2	H_2 (detected by GC) with II-1
1	484 ppm	trace
2	310 ppm	163 ppm
3	269 ppm	trace
4	6027 ppm	trace
5	1288 ppm	trace
6	43044 ppm	5829 ppm

SUPPORTING INFORMATION

VII. Computational Studies

All calculations were performed with the Gaussian 16 program package.[3] The geometries were optimized at the B3LYP level of density functional theory (DFT) with the 6-31+G(d,p) basis sets. Solvation effects were incorporated using the polarizable continuum model (PCM) with acetonitrile. Harmonic vibration frequency calculations at the same level were performed to verify all stationary points as local minima with no imaginary frequency and to derive the thermochemistry correction terms at 298 K and 1 atm.

The ground-state reduction potential of any species (S) can be calculated by the Gibbs free energy change between itself and its reduction state (S^-) in solution according to[4,5]

$$\Delta G^{\circ}(\text{soln, red}) = -nFE^{\circ}_{\text{abs}}(\text{S/S}^-) \quad \text{(eq. 1)}$$

$$\Delta G^{\circ}(\text{soln, red}) = G^{\circ}(\text{soln, S}^-) - G^{\circ}(\text{soln, S}) \quad \text{(eq. 2)}$$

where $E^{\circ}_{\text{abs}}(\text{S/S}^-)$: the absolute reduction potential of S, $\Delta G^{\circ}(\text{soln, red})$: the reduction free energy change in solution, $G^{\circ}(\text{soln, S}^-)$: the free energy of S^- in solution, $G^{\circ}(\text{soln, S})$: the free energy of S in solution, F : the Farady constant (23.06 kcal·mol^{-1}·V^{-1}) and n : the number of electrons transferred in the reduction reaction (here $n = 1$).

The relative reduction potential versus the saturated calomel electrode (SCE) in acetonitrile solution is corrected by[6]

$$E^{\circ}_{\text{SCE}}(\text{S/S}^-) = E^{\circ}_{\text{abs}}(\text{S/S}^-) - 4.429\text{ V} \quad \text{(eq. 3)}$$

where $E^{\circ}_{\text{SCE}}(\text{S/S}^-)$: the relative reduction potential of S versus SCE.

To identify the reactive sites, Mulliken charges were adopted to calculate the Fukui indices for electrophilic attack according to[7]

$$f_{\text{A}}^{-} = q_{\text{A}}^{\text{N}} - q_{\text{A}}^{\text{N-1}} \quad \text{(eq. 4)}$$

where f_A^-: Fukui index for electrophilic attack at atom A, q_{A}^{N}: Mulliken charge of atom A in the neutral state and $q_{\text{A}}^{\text{N-1}}$: Mulliken charge of atom A in the cationic state.

Table S11. Gibbs free energies and calculated redox potentials for **II-3**, **I-2**, and **III-1**.

	$G^{\circ}(\text{soln})$/H	$\Delta G^{\circ}(\text{soln, red})$/eV	$E^{\circ}_{\text{abs}}(\text{S/S}^-)$/V	$E^{\circ}_{\text{SCE}}(\text{S/S}^-)$/V
**II-3**	−1102.123647	−4.08	4.08	−0.35
**II-4**	−1101.973588			
**I-2⁻**	−1043.285529	−4.77	4.77	0.34
**I-2**	−1043.110194			

**III-1**	−2145.328227	−6.36	6.36	1.93
**III-1**$^{\bullet+}$	−2145.094284			
**III-1**$^{\bullet-}$	−2145.387084	−1.60	1.60	−2.83
**III-1**	−2145.328227			

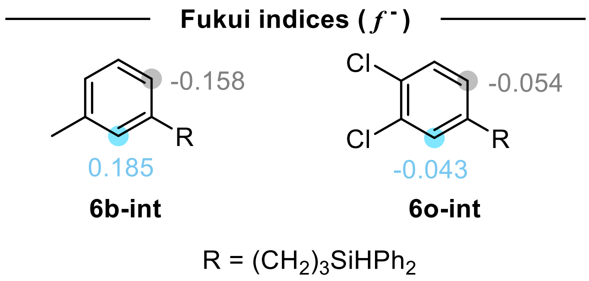

Figure S15. Fukui indices for intermediate of substrate **6b** and **6o**

Cartesian coordinates

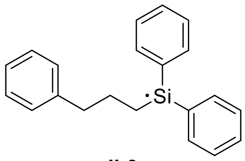

II-3

6	5.995090000	-1.301224000	1.024236000
6	4.730652000	-1.636798000	0.530016000
6	4.072967000	-0.811752000	-0.396808000
6	4.721408000	0.360586000	-0.817953000
6	5.985817000	0.701533000	-0.327336000
6	6.627841000	-0.128949000	0.597262000
1	6.486911000	-1.956680000	1.737806000
1	4.249320000	-2.553237000	0.863849000
1	4.232966000	1.009888000	-1.541014000
1	6.470325000	1.611616000	-0.670380000
1	7.611518000	0.131925000	0.977042000
6	2.688274000	-1.158501000	-0.901347000
6	1.559254000	-0.575072000	-0.023186000
1	2.573978000	-2.248946000	-0.943769000
1	2.566414000	-0.786527000	-1.926377000
6	0.156515000	-0.940831000	-0.542306000
1	1.680027000	-0.941654000	1.004083000
1	1.676650000	0.514835000	0.024633000
1	0.044040000	-0.611229000	-1.587044000
1	0.045367000	-2.032725000	-0.561768000
14	-1.306888000	-0.240428000	0.454161000

6	-2.890592000	-1.213503000	0.145762000
6	-2.871199000	-2.625667000	0.204704000
6	-4.131835000	-0.591149000	-0.110318000
6	-4.029827000	-3.379027000	0.004455000
1	-1.940011000	-3.147893000	0.411799000
6	-5.293987000	-1.342265000	-0.308342000
1	-4.192752000	0.491774000	-0.167637000
6	-5.248081000	-2.739070000	-0.252013000
1	-3.983463000	-4.463614000	0.051089000
1	-6.234235000	-0.836427000	-0.510736000
1	-6.150979000	-3.323095000	-0.405549000
6	-1.534773000	1.617791000	0.232165000
6	-2.434276000	2.340013000	1.047076000
6	-0.780175000	2.349272000	-0.709976000
6	-2.588394000	3.722046000	0.914392000
1	-3.019282000	1.818536000	1.801118000
6	-0.928336000	3.733388000	-0.843420000
1	-0.072796000	1.837977000	-1.357215000
6	-1.834459000	4.424241000	-0.032805000
1	-3.291327000	4.251162000	1.551926000
1	-0.337977000	4.270404000	-1.580843000
1	-1.949566000	5.499525000	-0.134822000

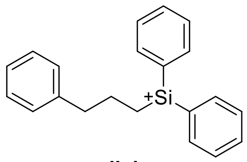

II-4

6	5.597443000	-1.537999000	1.222950000
6	4.429981000	-1.734978000	0.479061000
6	3.963318000	-0.750017000	-0.406009000
6	4.699493000	0.439194000	-0.528347000
6	5.867558000	0.641156000	0.213215000
6	6.320486000	-0.347671000	1.092784000
1	5.944068000	-2.314831000	1.898778000
1	3.877796000	-2.666277000	0.581843000
1	4.358463000	1.210816000	-1.214608000
1	6.425127000	1.566724000	0.100141000
1	7.229721000	-0.194387000	1.666828000
6	2.682008000	-0.952862000	-1.186803000
6	1.438175000	-0.447691000	-0.424333000
1	2.550484000	-2.017678000	-1.412839000

1	2.745204000	-0.426746000	-2.146995000
6	0.144543000	-0.679577000	-1.247006000
1	1.368826000	-0.967992000	0.537290000
1	1.560605000	0.617253000	-0.199992000
1	0.227204000	-0.171701000	-2.219814000
1	0.018427000	-1.743571000	-1.474439000
14	-1.427546000	-0.059556000	-0.472357000
6	-2.784910000	-1.222697000	-0.098684000
6	-2.507118000	-2.606482000	0.021507000
6	-4.123592000	-0.782263000	0.043001000
6	-3.529273000	-3.510979000	0.298623000
1	-1.493557000	-2.978223000	-0.090575000
6	-5.142534000	-1.694787000	0.304456000
1	-4.373708000	0.266842000	-0.072711000
6	-4.845256000	-3.056013000	0.438948000
1	-3.303097000	-4.567441000	0.399502000
1	-6.166079000	-1.347738000	0.401505000
1	-5.641493000	-3.764097000	0.647207000
6	-1.588598000	1.722493000	-0.102990000
6	-2.481910000	2.205421000	0.883453000
6	-0.772470000	2.650208000	-0.795141000
6	-2.558252000	3.568499000	1.159769000
1	-3.100887000	1.517865000	1.449758000
6	-0.864509000	4.013198000	-0.523572000
1	-0.072807000	2.311776000	-1.553145000
6	-1.755649000	4.471643000	0.453423000
1	-3.239916000	3.926649000	1.924331000
1	-0.241596000	4.715910000	-1.067279000
1	-1.820743000	5.533861000	0.668372000

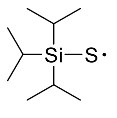

I-2

16	-0.168309000	2.450544000	-0.267660000
14	0.048759000	0.297284000	0.054453000
6	-1.263033000	-0.222902000	1.363217000
6	-1.487091000	-1.748247000	1.442492000
6	-2.619030000	0.500606000	1.226775000
1	-0.813432000	0.101475000	2.314328000
1	-0.558338000	-2.313163000	1.565097000
1	-2.131778000	-1.987424000	2.298228000

1	-1.990571000	-2.123631000	0.544956000
1	-2.509034000	1.589123000	1.258185000
1	-3.124164000	0.240616000	0.289770000
1	-3.286570000	0.209275000	2.048104000
6	1.832793000	0.200076000	0.796031000
6	2.931997000	0.481205000	-0.247753000
6	2.114045000	-1.112796000	1.556510000
1	1.859040000	1.011291000	1.536914000
1	2.791439000	1.444907000	-0.748112000
1	3.917083000	0.497945000	0.236015000
1	2.964594000	-0.293799000	-1.021806000
1	1.407951000	-1.277444000	2.375981000
1	2.080711000	-1.988618000	0.899671000
1	3.119712000	-1.074216000	1.995493000
6	-0.087124000	-0.550400000	-1.661006000
6	-1.476713000	-0.377714000	-2.307596000
6	0.350295000	-2.029912000	-1.699449000
1	0.629369000	0.018263000	-2.272658000
1	-1.788509000	0.672097000	-2.341028000
1	-1.467420000	-0.752743000	-3.339198000
1	-2.246641000	-0.939082000	-1.766128000
1	1.369525000	-2.174328000	-1.328049000
1	-0.313801000	-2.669182000	-1.108706000
1	0.324317000	-2.401537000	-2.732176000

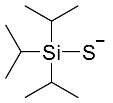

I-2⁻

16	-0.340681000	-2.503350000	-0.014308000
14	0.022923000	-0.408068000	-0.092022000
6	-1.215811000	0.473013000	-1.307879000
6	-1.327337000	2.004051000	-1.143160000
6	-2.630868000	-0.136319000	-1.331398000
1	-0.769770000	0.282572000	-2.298553000
1	-0.356492000	2.507606000	-1.135312000
1	-1.916922000	2.439808000	-1.962730000
1	-1.839690000	2.263426000	-0.209303000
1	-2.603871000	-1.215986000	-1.502072000
1	-3.153279000	0.031900000	-0.381295000
1	-3.240387000	0.323782000	-2.122901000
6	1.807785000	-0.145364000	-0.817128000

6	2.914107000	-0.594018000	0.158474000
6	2.144226000	1.240360000	-1.403943000
1	1.818304000	-0.855247000	-1.659554000
1	2.728731000	-1.601825000	0.544750000
1	3.896876000	-0.600360000	-0.334591000
1	2.991758000	0.082383000	1.018842000
1	1.447559000	1.533441000	-2.196099000
1	2.129885000	2.028089000	-0.641752000
1	3.152514000	1.237936000	-1.843237000
6	-0.068587000	0.320493000	1.705630000
6	-1.477239000	0.220394000	2.324117000
6	0.497954000	1.740478000	1.911213000
1	0.578442000	-0.366717000	2.273332000
1	-1.883405000	-0.793271000	2.245143000
1	-1.460181000	0.493302000	3.389296000
1	-2.181807000	0.900608000	1.829750000
1	1.533352000	1.829979000	1.568361000
1	-0.091000000	2.497023000	1.382026000
1	0.483977000	2.009585000	2.977564000

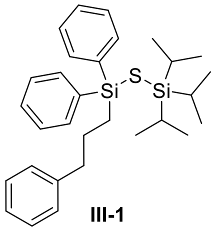

III-1

6	6.163297838	-2.959904194	-0.115947013
6	5.043394247	-2.421750131	-0.757886688
6	4.945087005	-1.043663500	-1.009223163
6	6.005379652	-0.218026633	-0.601016601
6	7.127774070	-0.750162901	0.041252118
6	7.210588192	-2.124845594	0.287179335
1	6.219352579	-4.029890474	0.064833320
1	4.236618922	-3.079837487	-1.072444303
1	5.952832775	0.851350222	-0.792577019
1	7.937981586	-0.092858203	0.344939028
1	8.082990546	-2.540848297	0.782935134
6	3.714722464	-0.458984801	-1.669752170
6	2.633450691	-0.021079025	-0.656619255
1	3.279349767	-1.197032947	-2.354920447
1	4.000650452	0.408632242	-2.277655362
6	1.387125089	0.568222053	-1.339013209

SUPPORTING INFORMATION

1	2.349647916	-0.884700099	-0.042684468
1	3.069971798	0.715178279	0.029779314
1	1.678090760	1.420519100	-1.970805107
1	0.958867942	-0.173339056	-2.026140125
14	0.015328849	1.182952885	-0.181549473
6	-1.383770068	1.913357493	-1.228638397
6	-1.658367784	1.440198582	-2.526911869
6	-2.175998137	2.970230580	-0.737577151
6	-2.684569880	1.992477752	-3.300331417
1	-1.069071353	0.631044854	-2.949783383
6	-3.204852384	3.524829446	-1.504291150
1	-1.988744692	3.369423402	0.255930145
6	-3.462630296	3.035597369	-2.789173511
1	-2.874562097	1.609143335	-4.299048862
1	-3.801499891	4.338562701	-1.101335502
1	-4.260142787	3.466251250	-3.387989290
6	0.703799285	2.541515965	0.951023620
6	0.380358201	2.655734683	2.315749239
6	1.558926833	3.518839017	0.400252455
6	0.886587373	3.698887316	3.099278443
1	-0.272420767	1.919536865	2.775695128
6	2.067692689	4.563716360	1.176891546
1	1.836287446	3.472940881	-0.650196752
6	1.731860449	4.656287331	2.531711078
1	0.620452313	3.761913417	4.150910713
1	2.725330218	5.301806590	0.726040494
1	2.126774916	5.466326739	3.138404179
16	-0.628086414	-0.401303188	1.176808539
14	-2.341311363	-1.634213991	0.496298034
6	-1.931405522	-2.519126397	-1.161439554
6	-2.887278120	-3.692705753	-1.478907411
6	-0.473430062	-3.001537315	-1.299479975
1	-2.096617692	-1.743467852	-1.924997350
1	-3.944068053	-3.420910460	-1.412066927
1	-2.706138822	-4.057433758	-2.498332408
1	-2.716219548	-4.536191967	-0.801019433
1	0.249345109	-2.202960109	-1.118738697
1	-0.251744055	-3.811048053	-0.595415990
1	-0.299610211	-3.392441237	-2.310645653
6	-3.835449729	-0.442431319	0.304803245
6	-4.288529003	0.154573766	1.653118997
6	-5.043381002	-0.998867530	-0.478289882

1	-3.427424960	0.383410806	-0.292204050
1	-3.454150072	0.596129718	2.207579117
1	-5.033545994	0.943900682	1.488682802
1	-4.754593615	-0.600829997	2.296150218
1	-4.773042290	-1.300777322	-1.494826084
1	-5.502147011	-1.861272645	0.017928967
1	-5.818850175	-0.226305522	-0.564577238
6	-2.537404687	-2.794480730	2.016551719
6	-1.349287402	-3.752096496	2.240605366
6	-3.866912338	-3.580155716	2.044596012
1	-2.558320105	-2.096396877	2.866848068
1	-0.389702187	-3.226147757	2.261249983
1	-1.461129749	-4.278782320	3.197457100
1	-1.296624725	-4.515715914	1.456314797
1	-4.742580281	-2.928208906	1.975241005
1	-3.928476491	-4.308511339	1.229355300
1	-3.950645472	-4.139554124	2.985624643

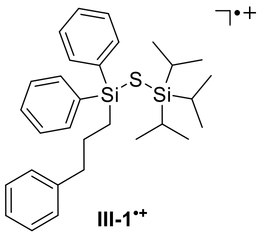

III-1•+

6	6.396120217	-2.661473166	-0.362637128
6	5.348020486	-2.011012291	-1.021253823
6	5.202872268	-0.616334642	-0.947016642
6	6.139661184	0.110983435	-0.195257866
6	7.189524768	-0.534248947	0.465463865
6	7.320917045	-1.924607960	0.384747193
1	6.492599760	-3.741123418	-0.436845724
1	4.637151165	-2.591849443	-1.604499752
1	6.048762556	1.192923780	-0.131638830
1	7.905838884	0.048315388	1.038176779
1	8.137530622	-2.427601360	0.894571266
6	4.046408647	0.082795304	-1.629860519
6	2.796742418	0.184435578	-0.726787793
1	3.777444674	-0.454215890	-2.547605472
1	4.351598348	1.092769022	-1.929247368
6	1.633866136	0.902791251	-1.433655554
1	2.486969179	-0.824752371	-0.433476156
1	3.071032232	0.703969760	0.198315594

1	1.960193273	1.882712114	-1.815130686
1	1.323846010	0.332447988	-2.317898217
14	0.068349838	1.293700274	-0.445184774
6	-1.340342814	1.828028318	-1.555298677
6	-1.579003331	1.164847308	-2.777209104
6	-2.147408320	2.936899838	-1.231232043
6	-2.595201966	1.586893189	-3.637175179
1	-0.970457951	0.313547822	-3.069912969
6	-3.162661294	3.361533825	-2.092646513
1	-1.984318564	3.480806307	-0.305932259
6	-3.389774168	2.685845779	-3.295173712
1	-2.762924885	1.061749070	-4.572702213
1	-3.773038601	4.218704219	-1.824565075
1	-4.178726132	3.015830366	-3.964485531
6	0.375121165	2.441051067	1.016038273
6	-0.599296484	2.642822711	2.022477435
6	1.598328064	3.133676891	1.127768826
6	-0.367613883	3.518764588	3.084520478
1	-1.552207620	2.127042695	1.974126388
6	1.826660220	4.015249637	2.184761116
1	2.374769595	3.005758366	0.380475681
6	0.845510307	4.206187414	3.168048646
1	-1.131469828	3.661167752	3.842544382
1	2.766944722	4.554772540	2.242905213
1	1.030558421	4.886739906	3.993480566
16	-0.324242740	-0.607068441	0.704844569
14	-2.313918820	-1.680236701	0.544262600
6	-2.193615421	-2.563451860	-1.177573269
6	-3.246011923	-3.689162589	-1.265254214
6	-0.809504970	-3.099725196	-1.585853625
1	-2.476780770	-1.764399738	-1.876702951
1	-4.253731233	-3.373147487	-0.984083804
1	-3.294477172	-4.044514478	-2.302749603
1	-2.969239088	-4.545332869	-0.642612594
1	-0.059190799	-2.308742863	-1.666329489
1	-0.434239971	-3.846240023	-0.879400961
1	-0.885917260	-3.586170911	-2.565786161
6	-3.692633431	-0.350311591	0.586340013
6	-3.946728481	0.189148531	2.009481249
6	-5.015987734	-0.796928861	-0.072079242
1	-3.295406179	0.470572268	-0.023789755
1	-3.031651543	0.521384276	2.509545785

1	-4.629136030	1.046066277	1.963474293
1	-4.414748318	-0.565041263	2.650271383
1	-4.885736332	-1.084304943	-1.119292878
1	-5.483437738	-1.633309844	0.458219729
1	-5.727021571	0.037881027	-0.049991071
6	-2.242009998	-2.798103086	2.094605876
6	-1.057756475	-3.784715701	2.116945379
6	-3.574158651	-3.541973392	2.350549353
1	-2.102235247	-2.089361126	2.923462049
1	-0.092529676	-3.288172337	1.976440067
1	-1.023615943	-4.299783344	3.084520632
1	-1.158631698	-4.553513420	1.343620374
1	-4.436704417	-2.870332923	2.386326037
1	-3.770543699	-4.301270983	1.587635487
1	-3.521533106	-4.056807144	3.317268254

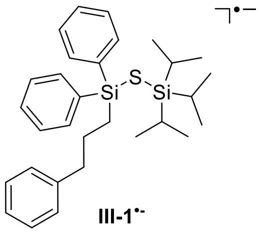

III-1•−

6	6.142403000	-2.767264000	-0.139268000
6	5.050220000	-2.195538000	-0.799606000
6	4.954771000	-0.805166000	-0.974214000
6	5.990979000	-0.002447000	-0.469299000
6	7.085728000	-0.567947000	0.192103000
6	7.165374000	-1.954428000	0.360575000
1	6.196005000	-3.845925000	-0.019071000
1	4.262429000	-2.836564000	-1.188745000
1	5.941105000	1.076259000	-0.599658000
1	7.877040000	0.073033000	0.571336000
1	8.016236000	-2.396306000	0.871236000
6	3.752023000	-0.186160000	-1.653609000
6	2.614280000	0.171275000	-0.670092000
1	3.360458000	-0.878294000	-2.409931000
1	4.058928000	0.723362000	-2.185691000
6	1.396384000	0.796841000	-1.370517000
1	2.307034000	-0.734195000	-0.133784000
1	3.011058000	0.855235000	0.090677000
1	1.714786000	1.681096000	-1.943127000
1	1.014696000	0.089788000	-2.119138000

14	-0.073358000	1.331051000	-0.277118000
6	-1.497720000	1.852647000	-1.312529000
6	-1.603384000	1.497587000	-2.707439000
6	-2.592514000	2.643334000	-0.799902000
6	-2.660223000	1.917274000	-3.502950000
1	-0.826995000	0.889858000	-3.167385000
6	-3.643649000	3.062213000	-1.601912000
1	-2.593356000	2.940304000	0.246484000
6	-3.706520000	2.716944000	-2.975708000
1	-2.682070000	1.623758000	-4.551695000
1	-4.436141000	3.666627000	-1.162203000
1	-4.536541000	3.037919000	-3.598209000
6	0.462263000	2.695728000	0.906486000
6	-0.346486000	3.108697000	1.998510000
6	1.659779000	3.424922000	0.698862000
6	0.009146000	4.188926000	2.811302000
1	-1.258708000	2.563653000	2.227071000
6	2.025358000	4.496584000	1.514346000
1	2.318494000	3.157759000	-0.123597000
6	1.197166000	4.892931000	2.579587000
1	-0.639368000	4.476384000	3.636005000
1	2.954702000	5.027122000	1.321068000
1	1.476812000	5.728973000	3.214519000
16	-0.475497000	-0.414283000	1.177461000
14	-2.098376000	-1.753924000	0.572027000
6	-1.774493000	-2.500290000	-1.177607000
6	-2.652886000	-3.728376000	-1.507067000
6	-0.300042000	-2.841451000	-1.470705000
1	-2.066014000	-1.690520000	-1.864854000
1	-3.718442000	-3.557527000	-1.331690000
1	-2.534362000	-4.006458000	-2.563133000
1	-2.353745000	-4.598091000	-0.911261000
1	0.363976000	-1.993307000	-1.290813000
1	0.049748000	-3.671234000	-0.845830000
1	-0.180914000	-3.150584000	-2.518257000
6	-3.712083000	-0.704215000	0.561022000
6	-4.132732000	-0.258881000	1.976588000
6	-4.917016000	-1.295567000	-0.199114000
1	-3.408758000	0.201265000	0.017606000
1	-3.304908000	0.208002000	2.520601000
1	-4.949847000	0.473310000	1.922186000
1	-4.494133000	-1.101104000	2.578598000

1	-4.685185000	-1.491780000	-1.250712000
1	-5.274137000	-2.231780000	0.245447000
1	-5.757889000	-0.588545000	-0.179470000
6	-2.122131000	-3.057098000	1.993800000
6	-0.854617000	-3.932542000	2.065104000
6	-3.382888000	-3.947862000	2.037697000
1	-2.137008000	-2.434352000	2.901138000
1	0.060543000	-3.332005000	2.073283000
1	-0.861083000	-4.545743000	2.976662000
1	-0.795295000	-4.621456000	1.214278000
1	-4.308321000	-3.365024000	2.066451000
1	-3.439729000	-4.617583000	1.173062000
1	-3.367829000	-4.580601000	2.935770000

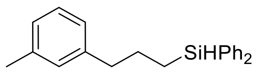

6b-Int

6	-4.805873000	-1.817205000	-0.723480000
6	-3.749755000	-1.777013000	0.192516000
6	-3.339130000	-0.557355000	0.750363000
6	-4.014355000	0.612532000	0.365693000
6	-5.073457000	0.594117000	-0.552954000
6	-5.463685000	-0.641787000	-1.093878000
1	-6.288453000	-0.683103000	-1.801407000
6	-2.176501000	-0.495548000	1.718466000
6	-0.814568000	-0.282254000	1.020451000
1	-2.132149000	-1.424773000	2.300299000
1	-2.335975000	0.320189000	2.434842000
6	0.358566000	-0.216463000	2.014233000
1	-0.656255000	-1.095582000	0.300831000
1	-0.860768000	0.644002000	0.434022000
1	0.195443000	0.606295000	2.725071000
1	0.384046000	-1.131555000	2.623642000
14	2.087672000	0.040993000	1.274102000
1	3.044911000	0.157331000	2.413363000
6	2.594555000	-1.423878000	0.183548000
6	2.080564000	-2.715245000	0.415578000
6	3.534817000	-1.275367000	-0.855324000
6	2.488360000	-3.811567000	-0.351513000
1	1.351248000	-2.877238000	1.205308000
6	3.947902000	-2.366805000	-1.626161000
1	3.951812000	-0.294896000	-1.071044000

6	3.424382000	-3.639243000	-1.375921000
1	2.074538000	-4.796068000	-0.151135000
1	4.674555000	-2.223454000	-2.421230000
1	3.742014000	-4.488412000	-1.974447000
6	2.218189000	1.666176000	0.315105000
6	2.855306000	2.781290000	0.894229000
6	1.661259000	1.827950000	-0.969923000
6	2.930946000	4.007491000	0.224383000
1	3.303061000	2.694173000	1.881512000
6	1.733016000	3.050065000	-1.645177000
1	1.168717000	0.990680000	-1.458568000
6	2.368628000	4.144209000	-1.048214000
1	3.430060000	4.851088000	0.693303000
1	1.296251000	3.147670000	-2.635299000
1	2.427216000	5.093995000	-1.572340000
6	-5.769863000	1.872241000	-0.963611000
1	-5.548595000	2.688014000	-0.269347000
1	-5.449117000	2.192062000	-1.962887000
1	-6.856362000	1.740583000	-1.001076000
1	-5.121375000	-2.768589000	-1.143566000
1	-3.248163000	-2.697456000	0.481952000
1	-3.709882000	1.563082000	0.800319000

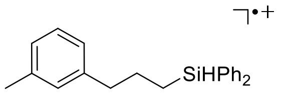

6b-Int•+

6	-4.617193000	-1.861935000	-0.789433000
6	-3.591516000	-1.751065000	0.134310000
6	-3.294438000	-0.481513000	0.769673000
6	-4.039375000	0.653203000	0.408663000
6	-5.067874000	0.565287000	-0.519462000
6	-5.350271000	-0.722376000	-1.123510000
1	-6.150684000	-0.786392000	-1.854476000
6	-2.185160000	-0.407017000	1.751869000
6	-0.799785000	-0.252226000	1.013610000
1	-2.143906000	-1.323660000	2.349842000
1	-2.318331000	0.447445000	2.420151000
6	0.350199000	-0.170706000	2.021069000
1	-0.662094000	-1.100364000	0.335134000
1	-0.844094000	0.652362000	0.398990000
1	0.194472000	0.672380000	2.706829000

1	0.378334000	-1.072899000	2.646429000
14	2.088512000	0.066389000	1.262073000
1	3.021369000	0.190173000	2.418021000
6	2.571735000	-1.421137000	0.198516000
6	2.101674000	-2.714613000	0.502501000
6	3.453951000	-1.287092000	-0.892142000
6	2.495659000	-3.827227000	-0.247200000
1	1.420394000	-2.866137000	1.336064000
6	3.853003000	-2.395970000	-1.645085000
1	3.836728000	-0.306319000	-1.161660000
6	3.373332000	-3.669664000	-1.324636000
1	2.117858000	-4.813249000	0.008539000
1	4.534844000	-2.264807000	-2.480728000
1	3.680282000	-4.532106000	-1.909432000
6	2.209043000	1.679139000	0.289414000
6	2.825369000	2.806595000	0.868478000
6	1.666301000	1.822174000	-1.004055000
6	2.895507000	4.027014000	0.188495000
1	3.260428000	2.733439000	1.862381000
6	1.732539000	3.039084000	-1.688421000
1	1.190935000	0.974838000	-1.492374000
6	2.347891000	4.145506000	-1.092341000
1	3.378519000	4.880525000	0.655951000
1	1.307996000	3.123357000	-2.684910000
1	2.401809000	5.091356000	-1.623828000
6	-5.898077000	1.740722000	-0.911368000
1	-5.583966000	2.651321000	-0.399961000
1	-5.840249000	1.899210000	-1.996202000
1	-6.955768000	1.550291000	-0.687359000
1	-4.844162000	-2.814736000	-1.253437000
1	-2.996985000	-2.618260000	0.403920000
1	-3.817980000	1.605701000	0.879427000

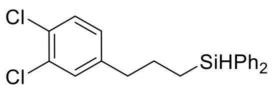

6o-Int

6	-3.986239000	-1.903003000	-0.065837000
6	-2.852789000	-1.828178000	0.742025000
6	-2.405169000	-0.600212000	1.252323000
6	-3.130942000	0.553731000	0.930426000
6	-4.267756000	0.487835000	0.121788000
6	-4.700667000	-0.744708000	-0.381464000

6	-1.152239000	-0.511069000	2.095250000
6	0.124973000	-0.290903000	1.253178000
1	-1.039425000	-1.432768000	2.678513000
1	-1.249668000	0.311333000	2.813916000
6	1.393581000	-0.193050000	2.117946000
1	0.218669000	-1.113213000	0.532682000
1	0.003322000	0.625432000	0.662319000
1	1.291218000	0.636959000	2.831458000
1	1.499939000	-1.099106000	2.731763000
14	3.029667000	0.085328000	1.194505000
1	4.094582000	0.258500000	2.225469000
6	3.460526000	-1.396290000	0.094670000
6	2.978870000	-2.687645000	0.387389000
6	4.314866000	-1.257254000	-1.017298000
6	3.334992000	-3.793132000	-0.392363000
1	2.316241000	-2.842735000	1.235116000
6	4.676283000	-2.357823000	-1.800758000
1	4.704030000	-0.277003000	-1.280913000
6	4.185814000	-3.630153000	-1.489859000
1	2.947577000	-4.777529000	-0.144363000
1	5.336868000	-2.221872000	-2.652708000
1	4.463293000	-4.486452000	-2.098094000
6	3.011202000	1.684380000	0.184720000
6	3.653107000	2.839423000	0.673121000
6	2.335309000	1.785602000	-1.048317000
6	3.619003000	4.046502000	-0.033786000
1	4.191359000	2.799162000	1.617299000
6	2.297131000	2.988311000	-1.760151000
1	1.835226000	0.915610000	-1.467214000
6	2.939340000	4.123139000	-1.253087000
1	4.124288000	4.922106000	0.364507000
1	1.769736000	3.039196000	-2.708763000
1	2.912451000	5.058041000	-1.805756000
1	-4.325468000	-2.857966000	-0.452207000
1	-2.313862000	-2.740744000	0.980186000
1	-2.816398000	1.519626000	1.312925000
17	-6.125049000	-0.875271000	-1.397193000
17	-5.131097000	1.973849000	-0.235672000

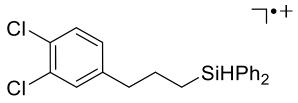

6o-Int•+

6	-3.881883000	-1.879348000	-0.041845000
6	-2.781418000	-1.768205000	0.774225000
6	-2.377346000	-0.497007000	1.303271000
6	-3.111572000	0.657257000	0.950320000
6	-4.217908000	0.564601000	0.130473000
6	-4.617840000	-0.722473000	-0.382024000
6	-1.174746000	-0.397122000	2.163157000
6	0.119279000	-0.238669000	1.262130000
1	-1.055648000	-1.302115000	2.765442000
1	-1.237832000	0.469624000	2.825429000
6	1.370663000	-0.131306000	2.130413000
1	0.180213000	-1.098756000	0.588552000
1	-0.007169000	0.655447000	0.644328000
1	1.303372000	0.734446000	2.801252000
1	1.479295000	-1.015314000	2.772134000
14	3.016936000	0.067611000	1.162698000
1	4.069198000	0.192977000	2.210102000
6	3.345606000	-1.449145000	0.084714000
6	2.944820000	-2.734789000	0.502659000
6	4.041653000	-1.346054000	-1.136821000
6	3.229261000	-3.869137000	-0.262599000
1	2.405593000	-2.862682000	1.438011000
6	4.330146000	-2.477534000	-1.905942000
1	4.366112000	-0.372951000	-1.495035000
6	3.922956000	-3.742407000	-1.471055000
1	2.909750000	-4.848484000	0.082283000
1	4.869771000	-2.370987000	-2.842767000
1	4.143598000	-4.622464000	-2.068288000
6	3.042786000	1.662661000	0.159936000
6	3.746312000	2.784758000	0.645367000
6	2.349174000	1.801063000	-1.060766000
6	3.758488000	3.993877000	-0.056239000
1	4.295399000	2.714356000	1.581125000
6	2.356974000	3.007092000	-1.765516000
1	1.799983000	0.959023000	-1.474827000
6	3.062443000	4.107406000	-1.263761000
1	4.310580000	4.842924000	0.336541000
1	1.816624000	3.089205000	-2.704282000

1	3.069495000	5.045426000	-1.811383000
1	-4.197457000	-2.838991000	-0.433343000
1	-2.210688000	-2.653675000	1.033604000
1	-2.813981000	1.624992000	1.338215000
17	-5.971014000	-0.876006000	-1.404404000
17	-5.116068000	1.979436000	-0.267682000

VIII. Characteristic Data

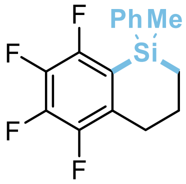

5,6,7,8-tetrafluoro-1-methyl-1-phenyl-1,2,3,4-tetrahydrobenzo[*b*]siline **(3a)**

Following the general procedure for defluorosilacyclization, the product **3a** was isolated by flash chromatography (PE) as colorless oil (53.3 mg, 86%). ^{1}H NMR (400 MHz, $CDCl_3$) δ 7.53 (d, J = 7.4 Hz, 2H), 7.41 – 7.35 (m, 3H), 2.80 (t, J = 6.0 Hz, 2H), 2.00 (p, J = 6.2 Hz, 2H), 1.20 – 1.03 (m, 2H), 0.63 (s, 3H). ^{13}C NMR (101 MHz, $CDCl_3$) δ 151.7 – 149.3 (m), 146.5 – 143.9 (m), 142.6 – 139.8 (m), 139.4 – 136.6 (m), 135.9, 133.9, 130.5 – 130.2 (m), 129.7, 127.9, 116.7 – 116.4 (m), 25.7, 20.8, 11.8zf, 3.4. ^{19}F NMR (376 MHz, $CDCl_3$) δ -126.06 – -126.19 (m, 1F), -142.05 – -142.15 (m, 1F), -154.61 – -154.73 (m, 1F), -159.67 – -159.80 (m, 1F). HRMS (ESI): calculated for $C_{16}H_{14}F_4SiNa^+$ $[M+Na]^+$ 333.0699, found 333.0704.

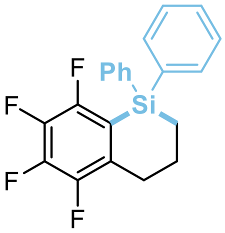

5,6,7,8-tetrafluoro-1,1-diphenyl-1,2,3,4-tetrahydrobenzo[*b*]siline (**3b**)

Following the general procedure for defluorosilacyclization, the product **3b** was isolated by flash chromatography (PE) as colorless oil (70.0 mg, 90%). ^{1}H NMR (400 MHz, $CDCl_3$) δ 7.59 (d, J = 7.2 Hz, 4H), 7.47 – 7.38 (m, 6H), 2.89 (t, J = 6.2 Hz, 2H), 2.07 (p, J = 6.4 Hz, 2H), 1.41 (t, J = 6.6 Hz, 2H). ^{19}F NMR (376 MHz, $CDCl_3$) δ -122.67 – -122.79 (m, 1F), -141.41 – -141.51 (m, 1F), -153.66 – -153.99 (m, 1F), -159.10 – -159.17 (m, 1F). ^{13}C NMR (101 MHz, $CDCl_3$) δ 151.7 – 149.2 (m), 146.7 – 144.1 (m), 142.9 – 140.1 (m), 139.6 – 136.7 (m), 135.1, 133.6, 130.9 – 130.6 (m),

130.0, 128.1, 115.3 – 115.0 (m), 25.8, 20.7, 11.2, 3.4. HRMS (ESI): calculated for $C_{21}H_{16}F_4SiNa^+$ $[M+Na]^+$ 395.0855, found 395.0855.

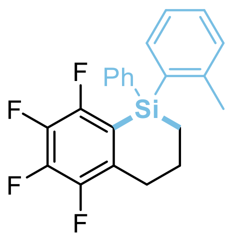

5,6,7,8-tetrafluoro-1-phenyl-1-(o-tolyl)-1,2,3,4-tetrahydrobenzo[*b*]siline (**3c**)

Following the general procedure for defluorosilacyclization, the product **3c** was isolated by flash chromatography (PE) as colorless oil (67.9 mg, 88%). ^{1}H NMR (400 MHz, $CDCl_3$) δ 7.63 (d, *J* = 7.0 Hz, 2H), 7.44 – 7.31 (m, 5H), 7.24 – 7.16 (m, 2H), 2.99 – 2.76 (m, 2H), 2.25 (s, 3H), 2.08 (p, *J* = 6.2 Hz, 2H), 1.64 – 1.35 (m, 2H). ^{13}C NMR (101 MHz, $CDCl_3$) δ 151.7 – 149.2 (m), 146.7 – 144.1 (m), 144.0, 142.9 – 140.0 (m), 139.6 – 136.7 (m), 136.6, 134.9, 134.3, 131.7, 130.8 – 130.6 (m), 130.3, 130.2, 139.9, 128.1, 125.2, 115.4 – 115.1 (m), 25.6, 23.3, 21.1, 11.0. ^{19}F NMR (376 MHz, $CDCl_3$) δ -122.59 – -122.71 (m, 1F), -141.51 – -141.61 (m, 1F), -153.78 – -153.90 (m, 1F), -158.96 – -159.09 (m, 1F). HRMS (ESI): calculated for $C_{22}H_{18}F_4SiH^+$ $[M+H]^+$ 387.1192, found 387.1178.

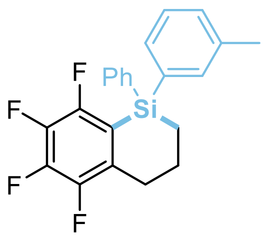

5,6,7,8-tetrafluoro-1-phenyl-1-(m-tolyl)-1,2,3,4-tetrahydrobenzo[*b*]siline (**3d**)

Following the general procedure for defluorosilacyclization, the product **3d** was isolated by flash chromatography (PE) as colorless oil (47.9 mg, 62%). ^{1}H NMR (400 MHz, $CDCl_3$) δ 7.58 (d, *J* = 7.0 Hz, 2H), 7.57 – 7.37 (m, 5H), 7.31 – 7.27 (m, 2H), 2.88 (t, *J* = 6.0 Hz, 2H), 2.36 (s, 3H), 2.06 (p, *J* = 6.2 Hz, 2H), 1.39 (t, *J* = 6.4 Hz,

2H). ^{13}C NMR (101 MHz, $CDCl_3$) δ 151.7 – 149.2 (m), 146.7 – 144.1 (m), 142.9 – 140.0 (m), 139.6 – 136.7 (m), 137.5, 135.6, 135.1, 133.7, 133.3, 132.2, 130.9, 130.8 – 130.6 (m), 130.0, 128.0, 127.9, 115.4 – 115.1 (m), 25.8, 21.5, 20.7, 11.3. ^{19}F NMR (376 MHz, $CDCl_3$) δ -122.63 – -122.75 (m, 1F), -140.43 – -141.59 (m, 1F), -153.78 – -153.92 (m, 1F), -158.40 – -159.21 (m, 1F). HRMS (ESI): calculated for $C_{22}H_{18}F_4SiNa^+$ $[M+Na]^+$ 409.1012, found 409.1001.

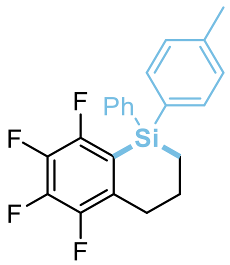

5,6,7,8-tetrafluoro-1-phenyl-1-(p-tolyl)-1,2,3,4-tetrahydrobenzo[*b*]siline (**3e**)

Following the general procedure for defluorosilacyclization, the product **3e** was isolated by flash chromatography (PE) as colorless oil (57.9 mg, 75%). ^{1}H NMR (400 MHz, $CDCl_3$) δ 7.59 (d, J = 7.1 Hz, 2H), 7.50 (d, J = 7.7 Hz, 2H), 7.45 – 7.38 (m, 3H), 7.24 (d, J = 7.7 Hz, 2H), 2.89 (t, J = 6.0 Hz, 2H), 2.40 (s, 3H), 2.07 (p, J = 6.4 Hz, 2H), 1.40 (t, J = 6.6 Hz, 2H). ^{13}C NMR (101 MHz, $CDCl_3$) δ 151.7 – 149.2 (m), 146.7 – 144.1 (m), 142.9 – 140.2 (m), 140.1, 139.5 – 136.7 (m), 135.2, 135.1, 133.8, 130.9 – 130.6 (m), 129.9, 129.8, 128.9, 128.0, 115.6 – 115.2 (m), 25.8, 21.5, 20.7, 11.2. ^{19}F NMR (376 MHz, $CDCl_3$) δ -122.75 – -122.87 (m, 1F), -141.50 – -141.60 (m, 1F), -153.84 – -153.96 (m, 1F), -159.11 – -159.24 (m, 1F). HRMS (ESI): calculated for $C_{22}H_{18}F_4SiH^+$ $[M+H]^+$ 387.1192, found 387.1182.

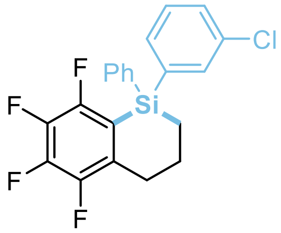

SUPPORTING INFORMATION

1-(3-chlorophenyl)-5,6,7,8-tetrafluoro-1-phenyl-1,2,3,4-tetrahydrobenzo[*b*]siline (**3f**)

Following the general procedure for defluorosilacyclization, the product **3f** was isolated by flash chromatography (PE) as colorless oil (42.2 mg, 52%). ^{1}H NMR (400 MHz, $CDCl_3$) δ 7.55 (d, J = 7.2 Hz, 2H), 7.50 (s, 1H), 7.46 – 7.38 (m, 5H), 7.50 (s, 1H), 7.33 – 7.29 (s, 1H), 2, 87 (t, J = 6.2 Hz, 2H), 2.05 (p, J = 6.4 Hz, 2H), 1.40 – 1.35 (m, 2H). ^{13}C NMR (101 MHz, $CDCl_3$) δ 151.7 – 149.2 (m), 146.7 – 144.2 (m), 143.1 – 140.3 (m), 139.7 – 136.8 (m), 136.5, 135.0, 134.6, 134.5, 133.1, 132.6, 130.9 – 130.7 (m), 130.3, 130.2, 129.5, 128.2, 114.5 – 114.2 (m), 25.7, 20.6, 11.0. ^{19}F NMR (376 MHz, $CDCl_3$) δ -122.63 – -122.82 (m, 1F), -141.04 – -141.55 (m, 1F), -153.02 – -153.14 (m, 1F), -158.68 – -158.81 (m, 1F). HRMS (ESI): calculated for $C_{21}H_{15}ClF_4SiH^+$ $[M+H]^+$ 407.0646, found 407.0653.

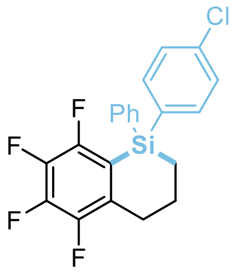

1-(4-chlorophenyl)-5,6,7,8-tetrafluoro-1-phenyl-1,2,3,4-tetrahydrobenzo[*b*]siline (**3g**)

Following the general procedure for defluorosilacyclization, the product **3g** was isolated by flash chromatography (PE) as colorless oil (51.2 mg, 63%). ^{1}H NMR (400 MHz, $CDCl_3$) δ 7.56 (d, J = 7.2 Hz, 2H), 7.49 (d, J = 7.4 Hz, 2H), 7.45 – 7.35 (m, 5H), 2.87 (t, J = 6.0 Hz, 2H), 2.04 (p, J = 6.2 Hz, 2H), 1.40 – 1.35 (m, 2H). ^{13}C NMR (101 MHz, $CDCl_3$) δ 151.7 – 149.2 (m), 146.8 – 144.2 (m), 142.9 – 140.2 (m), 139.5 – 136.6 (m), 136.5, 136.4, 135.0, 132.9, 132.0, 130.9 – 130.2 (m), 130.2, 128.4, 128.2, 114.8 – 114.4 (m), 25.7, 20.6, 11.1. ^{19}F NMR (376 MHz, $CDCl_3$) δ -122.81 – -122.93 (m, 1F), -140.93 – -141.20 (m, 1F), -153.18 – -153.30 (m, 1F), -158.80 – -158.93 (m, 1F). HRMS (ESI): calculated for $C_{21}H_{15}ClF_4SiNa^+$ $[M+Na]^+$ 429.0465, found 429.0479.

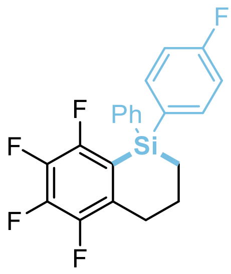

5,6,7,8-tetrafluoro-1-(4-fluorophenyl)-1-phenyl-1,2,3,4-tetrahydrobenzo[*b*]siline (**3h**)

Following the general procedure for defluorosilacyclization, the product **3h** was isolated by flash chromatography (PE) as colorless oil (46.0 mg, 59%). ^{1}H NMR (400 MHz, $CDCl_3$) δ 7.57 –7.54 (m, 4H), 7.47 –7.37 (m, 3H), 7.09 (t, *J* = 8.9 Hz, 2H), 2.88 (t, *J* = 5.7 Hz, 2H), 2.05 (p, *J* = 6.0 Hz, 2H), 1.44 – 1.32 (m, 2H). ^{13}C NMR (101 MHz, $CDCl_3$) δ 164.3 (d, *J* = 251.5 Hz), 151.7 – 149.2 (m), 146.8 – 144.2 (m), 143.0 – 140.1 (m), 139.6 – 136.7 (m), 137.2, 135.0, 133.3, 130.9 – 130.6 (m), 130.1, 129.1 (d, *J* = 4.4 Hz), 128.1, 115.3 (d, *J* = 20.2 Hz), 115.1 – 114.7 (m), 25.8, 20.6, 11.3. ^{19}F NMR (376 MHz, $CDCl_3$) δ -108.16 – -110.21 (m, 1F), -122.90 – -123.03 (m, 1F), -141.19 – -141.29 (m, 1F), -153.25 – -153.50 (m, 1F), -158.72 – -159.02 (m, 1F). HRMS (ESI): calculated for $C_{21}H_{15}F_5SiH^+$ $[M+H]^+$ 391.0941, found 391.0950.

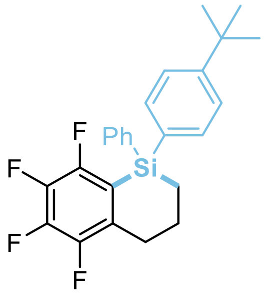

1-(4-(tert-butyl)phenyl)-5,6,7,8-tetrafluoro-1-phenyl-1,2,3,4-tetrahydrobenzo[*b*]siline (**3i**)

Following the general procedure for defluorosilacyclization, the product **3i** was isolated by flash chromatography (PE) as colorless oil (62.5 mg, 73%). ^{1}H NMR (400 MHz, $CDCl_3$) δ 7.59 (d, *J* = 7.0 Hz, 2H), 7.54 (d, *J* = 7.4 Hz, 2H), 7.46 – 7.37 (m, 5H), 2.88 (t, *J* = 6.2 Hz, 2H), 2.07 (p, *J* = 6.4 Hz, 2H), 1.42 – 1.37 (m, 2H), 1.35 (s, 9H). ^{13}C NMR (101 MHz, $CDCl_3$) δ 153.1, 151.7 – 149.2 (m), 146.7 – 144.1 (m), 142.9 – 140.1 (m), 139.6 – 136.7 (m), 135.1, 135.0, 133.9, 130.9 – 130.6 (m), 129.9,

129.7, 128.0, 125.1, 115.6 – 115.3 (m), 34.8, 31.2, 25.8, 20.8, 11.3. ^{19}F NMR (376 MHz, $CDCl_3$) δ -122.63 – -122.74 (m, 1F), -141.56 – -141.66 (m, 1F), -153.93 – -154.05 (m, 1F), -159.17 – -159.30 (m, 1F). HRMS (ESI): calculated for $C_{25}H_{24}F_4SiNa^+$ $[M+Na]^+$ 451.1481, found 451.1470.

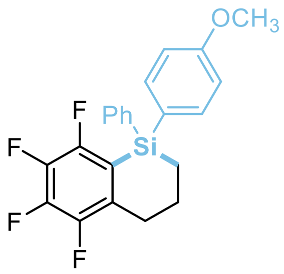

5,6,7,8-tetrafluoro-1-(4-methoxyphenyl)-1-phenyl-1,2,3,4-tetrahydrobenzo[*b*]siline (**3j**)

Following the general procedure for defluorosilacyclization, the product **3j** was isolated by flash chromatography (PE) as colorless oil (74.0 mg, 92%). ^{1}H NMR (400 MHz, $CDCl_3$) δ 7.61 – 7.59 (m, 2H), 7.55 (d, J = 7.9 Hz, 2H), 7.46 – 7.41 (m, 3H), 6.97 (d, J = 8.7 Hz, 2H), 3.85 (s, 3H), 2.89 (t, J = 6.6 Hz, 2H), 2.07 (p, J = 6.2 Hz, 2H), 1.40 (t, J= 6.1 Hz, 2H). ^{13}C NMR (101 MHz, $CDCl_3$) δ 161.3, 151.8 – 149.3 (m), 146.8 – 144.2 (m), 142.9 – 140.2 (m), 139.7 – 136.9 (m), 136.8, 135.1, 134.1, 130.9 – 130.7 (m), 130.0, 128.1, 124.1, 115.8 – 115.4 (m), 114.0, 55.1, 25.9, 20.8, 11.4. ^{19}F NMR (376 MHz, $CDCl_3$) δ -123.03 (ddd, 1F), -141.54 (ddd, 1F), -153.94 (td, 1F), -159.19 (ddd, 1F). HRMS (ESI): calculated for $C_{22}H_{18}F_4OSiNa^+$ $[M+Na]^+$ 425.0961, found 425.0953.

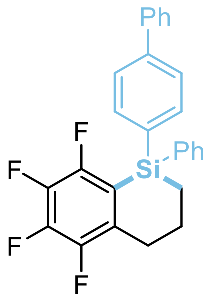

1-([1,1'-biphenyl]-4-yl)-5,6,7,8-tetrafluoro-1-phenyl-1,2,3,4-tetrahydrobenzo[*b*]siline (**3k**)

Following the general procedure for defluorosilacyclization, the product **3k** was isolated by flash chromatography (PE) as colorless oil (45.7 mg, 51%). ^{1}H NMR (400 MHz, $CDCl_3$) δ 7.67 – 7.61 (m, 8H), 7.48 – 7.35 (m, 6H), 2.90 (t, *J* = 6.4 Hz, 2H), 2.09 (p, *J* = 6.4 Hz, 2H), 1.45 (t, *J*= 6.4 Hz, 2H). ^{13}C NMR (101 MHz, $CDCl_3$) δ 151.7 – 149.2 (m), 146.8 – 144.1 (m), 142.9 – 140.2 (m), 142.8, 140.8, 140.5 – 136.9 (m), 135.6, 135.1, 133.5, 132.2, 130.9 – 130.6 (m), 130.0, 128.8, 128.0, 127,6, 127.2, 126.8, 115.3 – 114.9 (m), 25.8, 20.7, 11.3. ^{19}F NMR (376 MHz, $CDCl_3$) δ -122.63 – -122.75 (m, 1F), -144.33 – -144.43 (m, 1F), -153.56 – -153.69 (m, 1F), -158.96 – -159.32 (m, 1F). HRMS (ESI): calculated for $C_{27}H_{20}F_4SiNa^+$ $[M+Na]^+$ 471.1168, found 471.1165.

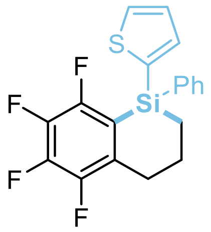

2-(5,6,7,8-tetrafluoro-1-phenyl-1,2,3,4-tetrahydrobenzo[*b*]silin-1-yl)thiophene (**3l**)

Following the general procedure for defluorosilacyclization, the product **3l** was isolated by flash chromatography (PE) as colorless oil (49.1 mg, 65%). ^{1}H NMR (400 MHz, $CDCl_3$) δ 7.72 (d, *J* = 5.4 Hz, 2H), 7.57 (d, *J* = 6.2 Hz, 2H), 7.46 – 7.35 (m, 4H), 7.26 – 7.24 (m, 1H), 2.86 (t, *J* = 6.6 Hz, 2H), 2.08 (p, *J* = 6.2 Hz, 2H), 1.47 – 1.37 (m, 2H). ^{13}C NMR (101 MHz, $CDCl_3$) δ 151.7 – 149.2 (m), 146.8 – 144.1 (m), 142.9 – 140.2 (m), 142.8, 140.8, 140.5 – 136.9 (m), 137.6, 134.7, 133.6, 132.4, 132.2, 130.9 – 130.6 (m), 130.2, 128.6, 128.1, 115.1 – 114.6 (m), 25.7, 20.7, 12.2. ^{19}F NMR (376 MHz, $CDCl_3$) δ -122.71 – -122.83 (m, 1F), -141.36 – -141.48 (m, 1F), -153.35 – -153.47 (m, 1F), -158.88 – -159.02 (m, 1F). HRMS (ESI): calculated for $C_{19}H_{14}F_4SSiNa^+$ $[M+Na]^+$ 401.0419, found 401.0419.

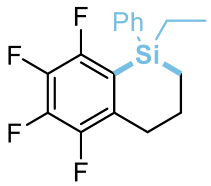

1-ethyl-5,6,7,8-tetrafluoro-1-phenyl-1,2,3,4-tetrahydrobenzo[*b*]siline (**3m**)

Following the general procedure for defluorosilacyclization, the product **3m** was isolated by flash chromatography (PE) as colorless oil (42.1 mg, 65%). ^{1}H NMR (400 MHz, $CDCl_3$) δ 7.54 (d, *J* = 6.3 Hz, 2H), 7.42 – 7.34 (m, 3H), 2.86 – 2.70 (m, 2H), 1.99 (p, *J* = 6.2 Hz, 2H), 1.23 – 1.08 (m, 4H), 1.03 (t, *J* = 7.6 Hz, 3H). ^{13}C NMR (101 MHz, $CDCl_3$) δ 151.7 – 149.2 (m), 146.6 – 144.0 (m), 142.6 – 139.7 (m), 139.4 – 136.5 (m), 135.2, 134.1, 130.8 – 130.5 (m), 129.6, 128.0, 115.9 – 115.5 (m), 25.7, 21.0, 9.3, 7.3, 5.2. ^{19}F NMR (376 MHz, $CDCl_3$) δ -125.57 – -125.69 (m, 1F), -141.91 – -142.01 (m, 1F), -154.50 – -154.62 (m, 1F), -159.64 – -159.76 (m, 1F). HRMS (ESI): calculated for $C_{17}H_{16}F_4SiH^+$ $[M+H]^+$ 325.1036, found 325.1044.

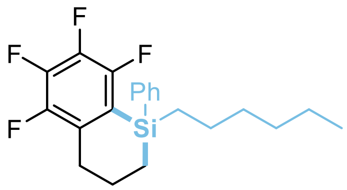

5,6,7,8-tetrafluoro-1-hexyl-1-phenyl-1,2,3,4-tetrahydrobenzo[*b*]siline (**3n**)

Following the general procedure for defluorosilacyclization, the product **3n** was isolated by flash chromatography (PE) as colorless oil (57.0 mg, 75%). ^{1}H NMR (400 MHz, $CDCl_3$) δ 7.53 (d, *J* = 6.7 Hz, 2H), 7.42 – 7.34 (m, 3H), 2.85 – 2.70 (m, 2H), 2.06 – 1.92 (m, 2H), 1.44 – 1.06 (m, 12H), 0.87 (d, *J* = 6.2 Hz, 3H). ^{13}C NMR (101 MHz, $CDCl_3$) δ 151.6 – 149.2 (m), 146.5 – 143.9 (m), 142.6 – 139.7 (m), 139.4 – 136.5 (m), 135.4, 134.1, 130.7 – 130.4 (m), 129.6, 128.0, 116.1 – 115.8 (m), 33.1, 31.4, 25.7, 23.7, 22.6, 21.0, 14.1, 13.3, 9.8. ^{19}F NMR (376 MHz, $CDCl_3$) δ -125.52 – -125.63 (m, 1F), -141.91 – -142.00 (m, 1F), -154.55 – -154.67 (m, 1F), -159.61 – -159.73 (m, 1F). HRMS (ESI): calculated for $C_{21}H_{24}F_4SiNa^+$ $[M+Na]^+$ 403.1481,

found 401.1483.

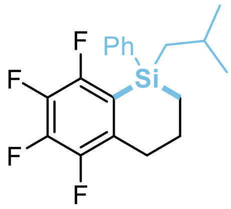

5,6,7,8-tetrafluoro-1-isobutyl-1-phenyl-1,2,3,4-tetrahydrobenzo[*b*]siline (**3o**)

Following the general procedure for defluorosilacyclization, the product **3o** was isolated by flash chromatography (PE) as colorless oil (57.0 mg, 81%). ^{1}H NMR (400 MHz, $CDCl_3$) δ 7.54 (d, *J* = 6.4 Hz, 2H), 7.41 – 7.33 (m, 3H), 2.92 – 2.66 (m, 2H), 2.12 – 2.03 (m, 1H), 1.98 – 1.84 (m, 1H), 1.30 – 1.11 (m, 4H), 0.96 (d, *J* = 6.6 Hz, 3H), 0.89 (d, *J* = 6.6 Hz, 3H). ^{13}C NMR (101 MHz, $CDCl_3$) δ 151.7 – 149.2 (m), 146.6 – 144.0 (m), 142.6 – 139.8 (m), 139.3 – 136.6 (m), 135.5, 134.3, 130.8 – 130.5 (m), 129.7, 128.1, 116.2 – 115.8 (m), 33.2, 31.5, 23.8, 21.1, 13.3, 9.8. ^{19}F NMR (376 MHz, $CDCl_3$) δ -125.16 – -125.29 (m, 1F), -141.79 – -141.89 (m, 1F), -154.57 – -154.69 (m, 1F), -159.57 – -159.70 (m, 1F). HRMS (ESI): calculated for $C_{19}H_{20}F_4SiNa^+$ $[M+Na]^+$ 375.1168, found 375.1168.

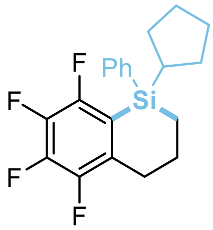

1-cyclopentyl-5,6,7,8-tetrafluoro-1-phenyl-1,2,3,4-tetrahydrobenzo[*b*]siline (**3p**)

Following the general procedure for defluorosilacyclization, the product **3p** was isolated by flash chromatography (PE) as colorless oil (43.0 mg, 59%). ^{1}H NMR (400 MHz, $CDCl_3$) δ 7.56 (d, *J* = 6.8 Hz, 2H), 7.41 – 7.33 (m, 3H), 2.91 – 2.63 (m, 2H), 2.10 – 2.01 (m, 2H), 1.97 – 1.86 (m, 2H), 1.77 – 1.54 (m, 6H), 1.46 – 1.31 (m, 2H),

1.24 – 1.00 (m, 2H). ^{13}C NMR (101 MHz, $CDCl_3$) δ 151.5 – 149.0 (m), 146.5 – 143.9 (m), 142.5 – 139.6 (m), 139.4 – 136.5 (m), 135.5, 134.3, 130.7 – 130.4 (m), 129.5, 128.0, 116.4 – 116.0 (m), 28.8, 28.2, 27.3, 26.9, 25.7, 23.3, 21.1, 7.9. ^{19}F NMR (376 MHz, $CDCl_3$) δ -123.47 – -125.06 (m, 1F), -141.28 – -142.01 (m, 1F), -154.53 – -154.81 (m, 1F), -159.50 – -159.75 (m, 1F). HRMS (ESI): calculated for $C_{20}H_{20}F_4SiH^+$ $[M+H]^+$ 365.1349, found 365.1357.

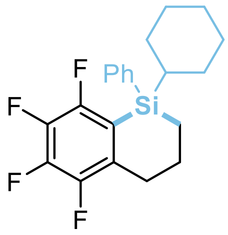

1-cyclohexyl-5,6,7,8-tetrafluoro-1-phenyl-1,2,3,4-tetrahydrobenzo[*b*]siline (**3q**)

Following the general procedure for defluorosilacyclization, the product **3q** was isolated by flash chromatography (PE) as colorless oil (48.4 mg, 64%). ^{1}H NMR (400 MHz, $CDCl_3$) δ 7.57 (d, J = 6.8 Hz, 2H), 7.41 – 7.34 (m, 3H), 2.85 – 2.62 (m, 2H), 2.09 – 2.00 (m, 1H), 1.96 – 1.85 (m, 1H), 1.80 – 1.65 (m, 4H), 1.56 – 1.44 (m, 2H), 1.33 – 1.11 (m, 7H). ^{13}C NMR (101 MHz, $CDCl_3$) δ 151.4 – 148.9 (m), 146.6 – 144.0 (m), 142.5 – 139.7 (m), 139.3 – 136.5 (m), 135.0, 134.4, 130.8 – 130.6 (m), 129.6, 128.0, 115.6 – 115.3 (m), 28.0, 27.9, 27.3, 26.8, 25.7, 23.8, 21.2, 7.9. ^{19}F NMR (376 MHz, $CDCl_3$) δ -124.73 – -124.85 (m, 1F), -141.74 – -141.84 (m, 1F), -154.57 – -154.69 (m, 1F), -159.56 – -159.68 (m, 1F). HRMS (ESI): calculated for $C_{21}H_{22}F_4SiH^+$ $[M+H]^+$ 379.1505, found 379.1509.

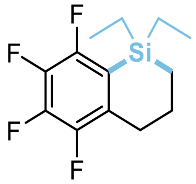

1,1-diethyl-5,6,7,8-tetrafluoro-1,2,3,4-tetrahydrobenzo[*b*]siline (**3r**)

Following the general procedure for defluorosilacyclization, the product **3r** was isolated by flash chromatography (PE) as colorless oil (24.8 mg, 45%). ^{1}H NMR (400 MHz, $CDCl_3$) δ 2.73 (d, *J* = 7 .4 Hz, 2H), 1.88 (p, *J* = 6.0 Hz, 2H), 1.03 – 0.88 (m, 5H), 0.68 – 0.56 (m, 2H). ^{13}C NMR (101 MHz, $CDCl_3$) δ 151.49 – 149.40 (m), 145.21 (ddd, *J* = 245.6, 9.5, 3.5 Hz), 142.10 – 139.64 (m), 139.12 – 136.62 (m), 25.80, 21.32, 8.10, 7.52, 5.40 (d, *J* = 1.6 Hz). ^{19}F NMR (376 MHz, $CDCl_3$) δ -127.58 (ddd, *J* = 25.4, 14.9, 4.0 Hz), -142.41 – -142.50 (m), -155.48 (td, *J* = 19.9, 3.8 Hz), -160.26 – -160.42 (m). HRMS (EI): calculated for $C_{13}H_{16}F_4Si$ [M] 276.0957, found 276.0953.

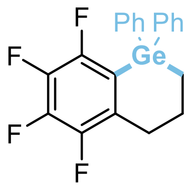

5,6,7,8-tetrafluoro-1,1-diphenyl-1,2,3,4-tetrahydrobenzo[*b*]germine (**3s**)

Following the general procedure for defluorosilacyclization, the product **3s** was synthesized using dihydrogermane and was isolated by flash chromatography (PE) as colorless oil (47.7 mg, 57%). ^{1}H NMR (400 MHz, $CDCl_3$) 1H NMR δ 7.57 – 7.49 (m, 4H), 7.44 – 7.34 (m, 6H), 2.81 (q, J = 9.5, 7.6 Hz, 2H), 2.09 (tdd, J = 7.6, 6.0, 3.7 Hz, 2H), 1.59 (dd, J = 7.9, 5.2 Hz, 2H). ^{13}C NMR (101 MHz, $CDCl_3$) δ 136.08, 135.77, 134.33, 134.32, 134.26, 134.24, 129.47, 129.36, 128.44, 116.84, 25.50, 22.15, 12.69. HRMS (ESI): calculated for $C_{21}H_{16}F_4GeH^+$ $[M+H]^+$ 419.0478, found 419.0479.

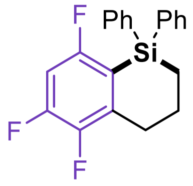

5,6,8-trifluoro-1,1-diphenyl-1,2,3,4-tetrahydrobenzo[*b*]siline (**4b**)

Following the general procedure for defluorosilacyclization, the product **4b** was isolated by flash chromatography (PE) as colorless oil (60.0 mg, 72%). ^{1}H NMR (400 MHz, $CDCl_3$) δ 7.58 (d, *J* = 7.0 Hz, 4H), 7.45 – 7.37 (m, 6H), 6.75 – 6.70 (m, 1H), 2.92 (t, *J* = 5.6 Hz, 2H), 2.06 (p, *J* = 6.5 Hz, 2H), 1.38 (t, *J* = 6.6 Hz, 2H). ^{13}C NMR (101 MHz, $CDCl_3$) δ 163.4 – 160.9 (m), 153.0 – 150.2 (m), 146.6 – 144.0 (m), 138.4 – 138.1 (m), 135.2, 134.3, 129.2, 128.0, 115.1 – 114.8 (m), 102.9 – 102.4 (m), 26.5, 20.9, 11.3. ^{19}F NMR (376 MHz, $CDCl_3$) δ -97.36 – -97.45 (m, 1F), -131.48 – -131.67 (m, 1F), -146.65 – -146.77 (m, 1F). HRMS (ESI): calculated for $C_{21}H_{17}F_3SiH^+$ $[M+H]^+$ 355.1130, found 355.1122.

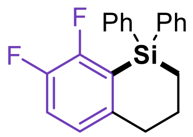

5,6,8-trifluoro-1,1-diphenyl-1,2,3,4-tetrahydrobenzo[*b*]siline (**4c**)

Following the general procedure for defluorosilacyclization, the product **4c** was isolated by flash chromatography (PE) as colorless oil (41.0 mg, 61%). ^{1}H NMR (400 MHz, $CDCl_3$) δ 7.60 (d, *J* = 6.8 Hz, 4H), 7.44 – 7.35 (m, 6H), 7.14 – 7.07 (m, 1H), 6.96 – 6.93 (m, 1H), 2.87 (t, *J* = 5.5 Hz, 2H), 2.05 (p, *J* = 5.6 Hz, 2H), 1.39 (t, *J* = 6.7 Hz, 2H). ^{19}F NMR (376 MHz, $CDCl_3$) δ -120.91 – -121.00 (m, 1F), -141.61 – -141.72 (m, 1F). ^{13}C NMR (101 MHz, $CDCl_3$) δ 155.4 – 152.9 (m), 149.7 – 147.0 (m), 145.4 – 145.3 (m), 135.3, 134.7, 129.8, 128.0, 125.6 – 125.5 (m), 121.2 – 20.9 (m), 118.8 – 118.6 (m), 34.7, 22.3, 11.8. HRMS (ESI): calculated for $C_{21}H_{18}F_2SiNa^+$ $[M+Na]^+$ 359.1044, found 359.1042.

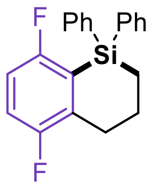

SUPPORTING INFORMATION

5,8-difluoro-1,1-diphenyl-1,2,3,4-tetrahydrobenzo[*b*]siline (**4d**)

Following the general procedure for defluorosilacyclization, the product **4d** was isolated by flash chromatography (PE) as colorless oil (47.0 mg, 70%). ^{1}H NMR (400 MHz, $CDCl_3$) δ 7.62 (d, *J* = 5.8 Hz, 4H), 7.45 – 7.36 (m, 6H), 7.07 – 7.01 (m, 1H), 6.84 – 6.79 (m, 1H), 2.90 (t, *J* = 5.8 Hz, 2H), 2.06 (p, *J* = 5.9 Hz, 2H), 1.39 (t, *J* = 6.4 Hz, 2H). ^{13}C NMR (101 MHz, $CDCl_3$) δ 163.0 (d, *J* = 237.8 Hz), 156.8 (d, *J* = 240.8 Hz), 136.7 (q, *J* = 4.5 Hz), 135.3, 134.6, 129.8, 128.0, 120.61 (q, *J* = 34.7 Hz), 117.8 (q, *J* = 16.6 Hz), 113.2 (q, *J* = 20.9 Hz), 26.5, 21.0, 11.5. ^{19}F NMR (376 MHz, $CDCl_3$) δ -101.06 – -101.15 (m, 1F), -122.62 – -122.71 (m, 1F). HRMS (ESI): calculated for $C_{21}H_{18}F_2SiNa^+$ $[M+Na]^+$ 359.1044, found 359.1034.

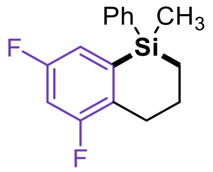

5,7-difluoro-1-methyl-1-phenyl-1,2,3,4-tetrahydrobenzo[*b*]siline (**4e**)

Following the general procedure for defluorosilacyclization, the product **4e** was isolated by flash chromatography (PE) as colorless oil (35.6 mg, 65%). ^{1}H NMR (400 MHz, $CDCl_3$) δ 7.51 – 7.46 (m, 2H), 7.41 – 7.32 (m, 3H), 6.89 (dd, *J* = 7.6, 2.4 Hz, 1H), 6.75 (ddd, *J* = 10.1, 8.9, 2.6 Hz, 1H), 2.82 – 2.74 (m, 2H), 2.04 – 1.95 (m, 2H), 1.22 – 1.06 (m, 2H), 0.53 (s, 3H). ^{13}C NMR (101 MHz, $CDCl_3$) δ 161.54 (dd, *J* = 70.1, 10.3 Hz), 159.06 (dd, *J* = 70.9, 10.2 Hz), 138.05 (dd, *J* = 4.5, 2.0 Hz), 137.09, 134.24, 130.69 (dd, *J* = 12.7, 3.4 Hz), 129.48, 127.97, 116.21 (dd, *J* = 17.5, 3.8 Hz), 104.06 (dd, *J* = 26.9, 25.0 Hz), 25.71, 21.26, 11.66, -2.99. ^{19}F NMR (376 MHz, $CDCl_3$) δ -113.44 (d, 1F), -114.53 (d, 1F). HRMS (ESI): calculated for $C_{16}H_{16}F_2SiH^+$ $[M+H]^+$ 275.1068, found 275.1059.

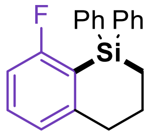

8-fluoro-1,1-diphenyl-1,2,3,4-tetrahydrobenzo[*b*]siline (**4f**)

Following the general procedure for defluorosilacyclization, the product **4f** was isolated by flash chromatography (PE) as colorless oil (32.4 mg, 51%). ^{1}H NMR (400 MHz, $CDCl_3$) δ 7.61 (d, *J* = 5.4 Hz, 4H), 7.42 – 7.29 (m, 7H), 7.01 (d, *J* = 7.6 Hz, 1H), 6.84 (t, *J* = 8.0 Hz, 1H), 2.92 (t, *J* = 6.2 Hz, 2H), 2.06 (p, *J* = 5.4 Hz, 2H), 1.39 (t, *J* = 5.9 Hz, 2H). ^{13}C NMR (101 MHz, $CDCl_3$) δ 167.6 (d, *J* = 242.4 Hz), 151.5 (d, *J* = 10.1 Hz), 135.3, 131.5 (d, *J* = 9.1 Hz), 129.6, 127.9, 125.2 (d, *J* = 2.8 Hz), 118.2 (d, *J* = 31.3 Hz), 112.4 (d, *J* = 25.7 Hz), 25.2, 22.2, 12.3. ^{19}F NMR (376 MHz, $CDCl_3$) δ -94.85 (s, 1H). HRMS (ESI): calculated for $C_{21}H_{19}FSiNa^+$ $[M+Na]^+$ 341.1138, found 341.1130.

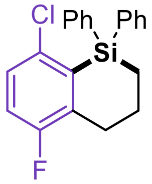

8-chloro-5-fluoro-1,1-diphenyl-1,2,3,4-tetrahydrobenzo[*b*]siline (**4g**)

Following the general procedure for defluorosilacyclization, the product **4g** was isolated by flash chromatography (PE) as colorless oil (47.9 mg, 68%). ^{1}H NMR (400 MHz, $CDCl_3$) δ 7.66 (d, *J* = 6.4 Hz, 4H), 7.46 – 7.36 (m, 6H), 7.18 – 7.14 (m, 1H), 7.05 (t, *J* =6.9 Hz, 1H), 2.94 (t, *J* = 5.8 Hz, 2H), 1.99 (p, *J* = 5.2 Hz, 2H), 1.33 (t, *J* = 5.5 Hz, 2H). ^{13}C NMR (101 MHz, $CDCl_3$) δ 159.2 (d, *J* = 246.4 Hz), 138.2 (d, *J* = 14.1 Hz), 137.0 (d, *J* = 3.0 Hz), 135.2, 133.8 (d, *J* = 9.1 Hz), 135.5, 134.5, 133.2, 129.5, 128.0 (d, *J* = 8.1 Hz), 127.8, 117.8 (d, *J* = 25.3 Hz), 26.8, 20.6, 13.9. ^{19}F NMR (376 MHz, $CDCl_3$) δ -118.57 (s, 1H). HRMS (ESI): calculated for $C_{21}H_{18}ClFSiNa^+$ $[M+Na]^+$ 375.0748, found 375.0745.

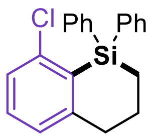

8-chloro-1,1-diphenyl-1,2,3,4-tetrahydrobenzo[*b*]siline (**4h**)

Following the general procedure for defluorosilacyclization, the product **4h** was isolated by flash chromatography (PE) as colorless oil (45.4 mg, 68%). ^{1}H NMR (400 MHz, $CDCl_3$) δ 7.54 (d, *J* = 7.2 Hz, 4H), 7.42 – 7.33 (m, 6H), 7.25 (d, *J* = 6.1 Hz, 1H), 7.19 – 7.14 (m, 1H), 7.04 (t, *J* =7.0 Hz, 1H), 2.91 (t, *J* = 6.2 Hz, 2H), 2.07 (p, *J* = 5.7 Hz, 2H), 1.41 (t, *J* = 5.2 Hz, 2H). ^{13}C NMR (101 MHz, $CDCl_3$) 161.5, 159.1, 135.7, 135.6, 134.4, 131.8, 129.6, 128.0, 126.9, 116.1, 26.1, 21.3, 11.0. HRMS (ESI): calculated for $C_{21}H_{18}ClSiNa^+$ $[M+Na]^+$ 357.0842, found 357.0849.

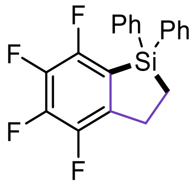

4,5,6,7-tetrafluoro-1,1-diphenyl-2,3-dihydro-1H-benzo[*b*]silole (**4i**)

Following the general procedure for defluorosilacyclization, the product **4i** was isolated by flash chromatography (PE) as colorless oil (20.1 mg, 28%). ^{1}H NMR (400 MHz, $CDCl_3$) δ 7.64 – 7.57 (m, 4H), 7.51 – 7.42 (m, 2H), 7.45 – 7.39 (m, 2H), 7.42 – 7.36 (m, 2H), 3.24 (ddd, *J* = 9.0, 5.8, 1.7 Hz, 2H), 1.66 – 1.57 (m, 2H). ^{13}C NMR (101 MHz, $CDCl_3$) δ 148.77 (dd, *J* = 242.6, 10.2 Hz), 145.62 – 143.40 (m), 142.15 (dddd, *J* = 254.9, 16.7, 12.7, 4.4 Hz), 140.09 – 137.63 (m), 135.04, 132.82, 130.52, 128.40, 119.75 (dt, *J* = 33.4, 2.4 Hz), 26.46 (d, *J* = 2.4 Hz), 9.59. ^{19}F NMR (376 MHz, $CDCl_3$) δ -125.01 – -125.19 (m, 1F), -141.29 – -141.44 (m, 1F), -152.44 (td, *J* = 19.4, 4.6 Hz, 1F), -157.42 (ddd, *J* = 24.9, 18.7, 2.3 Hz, 1F). HRMS (EI): calculated for $C_{20}H_{14}F_4Si$ [M] 358.0795, found 358.0796.

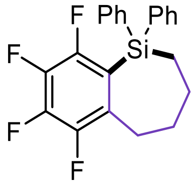

6,7,8,9-tetrafluoro-1,1-diphenyl-2,3,4,5-tetrahydro-1H-benzo[*b*]silepine (**4j**)

Following the general procedure for defluorosilacyclization, the product **4j** was isolated by flash chromatography (PE) as colorless oil (20.1 mg, 26%). ^{1}H NMR (400 MHz, $CDCl_3$) δ 7.50 – 7.44 (m, 4H), 7.44 – 7.41 (m, 2H), 7.39 – 7.34 (m, 4H), 2.81 – 2.75 (m, 2H), 1.94 – 1.85 (m, 2H), 1.77 (p, J = 6.3 Hz, 2H), 1.38 – 1.33 (m, 2H). ^{19}F NMR (376 MHz, $CDCl_3$) δ -121.74 (ddd, J = 24.4, 14.7, 5.6 Hz), -143.60 (ddd, J = 20.7, 14.7, 2.5 Hz), -152.84 (td, J = 20.1, 5.5 Hz), -158.30 (ddd, J = 24.4, 19.3, 2.5 Hz). ^{13}C NMR (101 MHz, $CDCl_3$) δ 151.99 – 149.78 (m), 146.44 – 144.23 (m), 142.83 – 140.33 (m), 139.67 – 137.17 (m), 135.08 (d, *J* = 2.2 Hz), 134.72, 129.86, 129.60 (ddd, *J* = 13.1, 9.3, 5.2 Hz), 128.08, 118.42 (dt, *J* = 24.6, 3.5 Hz), 27.41, 24.36, 23.36, 20.73, 11.68. HRMS (EI): calculated for $C_{22}H_{18}F_4Si$ [M] 386.1108, found 386.1115.

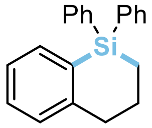

1,1-diphenyl-1,2,3,4-tetrahydrobenzo[*b*]siline (**6a**)

Following the general procedure for dehydrogenative silacyclization, the product **6a** was isolated by flash chromatography (PE) as colorless oil (44.1 mg, 73%). ^{1}H NMR (400 MHz, $CDCl_3$) δ 7.55 (dd, *J* = 7.7, 6.2 Hz, 4H), 7.49 (d, *J* = 6.4 Hz, 1H), 7.42 – 7.29 (m, 7H), 7.19 (q, *J* = 3.5 Hz, 2H), 2.90 (t, *J* = 5.6 Hz, 2H), 2.08 (p, *J* = 5.9 Hz, 2H), 1.42 (t, *J* = 5.8 Hz, 2H). ^{13}C NMR (101 MHz, $CDCl_3$) δ 13C NMR (126 MHz, Chloroform-d) δ 149.5, 136.3, 136.2, 135.4, 131.1, 129.4, 129.3, 128.9, 127.8, 125.5,

35.3, 22.5, 11.6. HRMS (EI): calculated for $C_{21}H_{20}Si$ [M] 300.1334, found 300.1337.

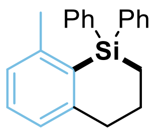

8-methyl-1,1-diphenyl-1,2,3,4-tetrahydrobenzo[*b*]siline (**6b**)

Following the general procedure for dehydrogenative silacyclization, the product **6b** was isolated by flash chromatography (PE) as colorless oil (25.7 mg, 41%). ^{1}H NMR (400 MHz, $CDCl_3$) δ 7.52 (dd, *J* = 7.4, 6.0 Hz, 4H), 7.34 – 7.25 (m, 6H), 7.19 – 7.15 (m, 1H), 6.98 (d, *J* = 7.6 Hz, 2H), 6.92 (t, *J* = 7.6 Hz, 2H), 2.85 (t, *J* = 5.6 Hz, 2H), 1.96 (s, 3H), 1.89 (p, *J* = 6.7 Hz, 2H), 1.42 (t, *J* = 5.6 Hz, 2H). ^{13}C NMR (101 MHz, $CDCl_3$) δ 150.3, 136.3, 135.4, 129.7, 129.2, 128.9, 127.9, 127.7, 127.6, 126.8, 36.4, 24.0, 22.1, 15.2. HRMS (EI): calculated for $C_{22}H_{22}Si$ [M] 314.1491, found 314.1488.

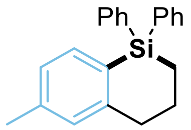

6-methyl-1,1-diphenyl-1,2,3,4-tetrahydrobenzo[*b*]siline (**6b′**)

Following the general procedure for dehydrogenative silacyclization, the product **6b′** was isolated by flash chromatography (PE) as colorless oil (19.5 mg, 31%). ^{1}H NMR (400 MHz, $CDCl_3$) δ 7.57 – 7.52 (m, 4H), 7.41 – 7.31 (m, 7H), 7.04 – 7.00 (m, 2H), 2.89 – 2.83 (m, 2H), 2.33 (s, 3H), 2.06 (dtd, *J* = 9.0, 6.3, 4.2 Hz, 2H), 1.43 – 1.37 (m, 2H). ^{13}C NMR (101 MHz, $CDCl_3$) δ 149.69, 139.39, 136.65, 136.40, 135.49, 129.85, 129.39, 127.94, 126.56, 35.43, 22.70, 21.65, 11.77. HRMS (EI): calculated for $C_{22}H_{22}Si$ [M] 314.1491, found 314.1488.

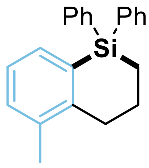

5-methyl-1,1-diphenyl-1,2,3,4-tetrahydrobenzo[*b*]siline (**6c**)

Following the general procedure for dehydrogenative silacyclization, the product **6c** was isolated by flash chromatography (PE) as colorless oil (44.6 mg, 71%). ^{1}H NMR (400 MHz, $CDCl_3$) δ 7.55 (dd, *J* = 7.4, 6.0 Hz, 4H), 7.40 – 7.31 (m, 7H), 7.20 (d, *J* = 7.4 Hz, 1H), 7.10 (t, *J* = 7.4 Hz, 1H), 2.82 (t, *J* = 5.4 Hz, 2H), 2.30 (s, 3H), 2.09 (p, *J* = 6.5 Hz, 2H), 1.38 (t, *J* = 5.4 Hz, 2H). ^{13}C NMR (101 MHz, $CDCl_3$) δ 147.5, 136.3, 135.8, 135.5, 134.2, 131.3, 130.8, 129.3, 127.8, 125.1, 31.2, 21.8, 20.5, 10.9. HRMS (EI): calculated for $C_{22}H_{22}Si$ [M] 314.1491, found 314.1492.

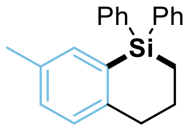

7-methyl-1,1-diphenyl-1,2,3,4-tetrahydrobenzo[*b*]siline (**6d**)

Following the general procedure for dehydrogenative silacyclization, the product **6d** was isolated by flash chromatography (PE) as colorless oil (46.5 mg, 74%). ^{1}H NMR (400 MHz, $CDCl_3$) δ 7.55 (dd, *J* = 7.6, 6.2 Hz, 4H), 7.41 – 7.32 (m, 6H), 7.24 (d, *J* = 4.3 Hz, 1H), 7.12 (d, *J* = 6.1 Hz, 2H), 2.85 (t, *J* = 5.5 Hz, 2H), 2.05 (p, *J* = 6.1Hz, 2H), 1.40 (t, *J* = 5.3 Hz, 2H). ^{13}C NMR (101 MHz, $CDCl_3$) δ 146.5, 136.5, 136.4, 135.4, 134.6, 130.7, 130.3, 129.3, 128.8, 127.8, 34.9, 22.6, 21.1, 11.7. HRMS (EI): calculated for $C_{22}H_{22}Si$ [M] 314.1491, found 314.1485.

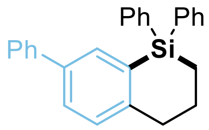

1,1,7-triphenyl-1,2,3,4-tetrahydrobenzo[*b*]siline (**6e**)

Following the general procedure for dehydrogenative silacyclization, the product **6e** was isolated by flash chromatography (PE) as colorless oil (48.1 mg, 64%). ^{1}H NMR (400 MHz, $CDCl_3$) δ 7.72 (d, *J* = 2 Hz, 1H), 7.59 (dd, *J* = 6.1, 1.7 Hz, 4H), 7.56 – 7.50 (m, 3H), 7.41 – 7.28 (m, 10H), 2.95 (t, *J* = 6.1 Hz, 2H), 2.11 (p, *J* = 6.3 Hz, 2H), 1.45 (t, *J* = 5.7 Hz, 2H). ^{13}C NMR (101 MHz, $CDCl_3$) δ 148.7, 141.2, 138.2, 136.1, 135.4, 134.7, 131.6, 129.4, 129.3, 128.7, 128.2, 127.9, 127.0, 126.9, 35.0, 22.5, 11.6. HRMS (EI): calculated for $C_{27}H_{24}Si$ [M] 376.1647, found 376.1644.

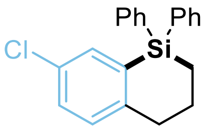

7-chloro-1,1-diphenyl-1,2,3,4-tetrahydrobenzo[*b*]siline (**6f**)

Following the general procedure for dehydrogenative silacyclization, the product **6f** was isolated by flash chromatography (PE) as colorless oil (44.1 mg, 66%). ^{1}H NMR (400 MHz, $CDCl_3$) δ 7.53 (dd, *J* = 7.2, 6.2 Hz, 4H), 7.43 – 7.34 (m, 7H), 7.28 (s, 1H), 7.13 – 7.06 (m, 2H), 2.85 (t, *J* = 5.8 Hz, 2H), 2.27 (s, 3H), 2.05 (p, *J* = 6.6 Hz, 2H), 1.39 (t, *J* = 5.6 Hz, 2H). ^{13}C NMR (101 MHz, $CDCl_3$) δ 147.6, 135.8, 135.4, 135.3, 133.8, 131.5, 130.4, 129.6, 129.4, 128.0, 34.7, 22.4, 11.1. HRMS (EI): calculated for $C_{21}H_{19}ClSi$ [M] 334.0945, found 334.0936.

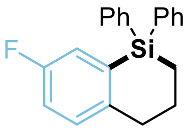

7-fluoro-1,1-diphenyl-1,2,3,4-tetrahydrobenzo[*b*]siline (**6g**)

Following the general procedure for dehydrogenative silacyclization, the product **6g** was isolated by flash chromatography (PE) as colorless oil (45.8 mg, 72%). ^{1}H NMR

(400 MHz, $CDCl_3$) δ 7.53 (dd, *J* = 7.4, 6.1 Hz, 4H), 7.43 – 7.34 (m, 6H), 7.17 – 7.12 (m, 2H), 7.00 – 6.95 (m, 1H), 2.86 (t, *J* = 5.6 Hz, 2H), 2.06 (p, *J* = 6.3 Hz, 2H), 1.41 (t, *J* = 5.6 Hz, 2H). ^{13}C NMR (101 MHz, $CDCl_3$) δ 160.8 (d, *J* = 197.8 Hz), 144.8, 135.5, 135.3, 133.7, 130.5 (d, *J* = 5.1 Hz), 129.6, 128.0, 121.6 (d, *J* = 14.6 Hz), 116.3 (d, *J* = 16.8 Hz), 34.5, 22.6, 11.0. ^{19}F NMR (376 MHz, $CDCl_3$) δ -117.17(s, 1F). HRMS (EI): calculated for $C_{21}H_{19}FSi$ [M] 318.1240, found 318.1234.

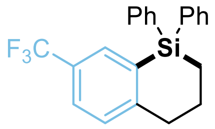

1,1-diphenyl-7-(trifluoromethyl)-1,2,3,4-tetrahydrobenzo[*b*]siline (**6h**)

Following the general procedure for dehydrogenative silacyclization, the product **6h** was isolated by flash chromatography (PE) as colorless oil (45.6 mg, 62%). ^1H NMR (400 MHz, $CDCl_3$) δ 7.71 (s, 1H), 7.55 – 7.51 (m, 5H), 7.44 – 7.35 (m, 6H), 7.29 (d, *J* = 8.1 Hz, 1H), 2.94 (t, *J* = 5.7 Hz, 2H), 2.09 (p, *J* = 6.2 Hz, 2H), 1.45 (t, *J* = 5.5 Hz, 2H). ^{19}F NMR (376 MHz, $CDCl_3$) δ -62.24 (s, 1F). ^{13}C NMR (101 MHz, $CDCl_3$) δ 153.4, 135.5, 135.3, 135.2, 132.6, 132.4 (d, *J* = 2.9 Hz), 129.7, 129.2, 128.1, 126.0 (d, *J* = 2.4 Hz), 35.3, 22.2, 11.2. HRMS (EI): calculated for $C_{22}H_{19}F_3Si$ [M] 368.1208, found 368.1207.

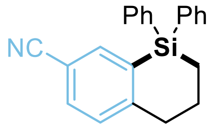

1,1-diphenyl-1,2,3,4-tetrahydrobenzo[*b*]siline-7-carbonitrile (**6i**)

Following the general procedure for dehydrogenative silacyclization, the product **6i** was isolated by flash chromatography (PE) as colorless oil (33.2 mg, 51%). ^1H NMR (400 MHz, $CDCl_3$) δ 7.71 ((d, *J* = 1.8 Hz, 1H), 7.53 (dd, *J* = 6.4, 1.8 Hz, 1H), 7.48

(dd, J = 6.1, 1.7 Hz, 4H), 7.43 – 7.34 (m, 6H), 7.24 (s, 1H), 2.92 (t, J = 5.9 Hz, 2H), 2.07 (p, J = 6.4 Hz, 2H), 1.43 (t, J = 5.8 Hz, 2H). ^{13}C NMR (101 MHz, $CDCl_3$) δ 154.5, 139.8, 135.3, 134.6, 133.8, 132.4, 129.9, 129.5, 128.2, 119.4, 109.6, 35.6, 22.0, 10.8. HRMS (EI): calculated for $C_{22}H_{19}F_3Si$ [M] 325.1287, found 368.1282.

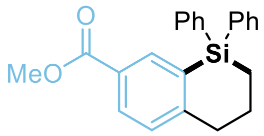

Methyl 1,1-diphenyl-1,2,3,4-tetrahydrobenzo[*b*]siline-7-carboxylate (**6j**)

Following the general procedure for dehydrogenative silacyclization, the product **6j** was isolated by flash chromatography (PE) as colorless oil (45.8 mg, 64%). ^{1}H NMR (400 MHz, $CDCl_3$) δ 8.14 (d, J = 1.8 Hz, 1H), 7.88 (dd, J = 6.2, 1.8 Hz, 1H), 7.54 (dd, J = 6.2, 1.8 Hz, 4H), 7.55 – 7.53 (m, 6H), 7.25 (s, 1H), 3.84 (s, 3H), 2.94 (t, J = 6.3 Hz, 2H), 2.08 (p, J = 6.6 Hz, 2H), 1.43 (t, J = 6.0 Hz, 2H). ^{13}C NMR (101 MHz, $CDCl_3$) δ 142.2, 135.4, 135.2, 134.4, 129.6, 128.9, 128.6, 128.3, 128.1, 127.9, 125.8, 39.3, 26.4, 11.9. HRMS (EI): calculated for $C_{23}H_{22}O_2Si$ [M] 400.1859, found 400.1866.

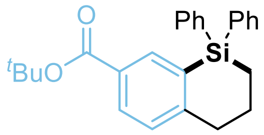

tert-Butyl 1,1-diphenyl-1,2,3,4-tetrahydrobenzo[*b*]siline-7-carboxylate (**6k**)

Following the general procedure for dehydrogenative silacyclization, the product **6k** was isolated by flash chromatography (PE) as colorless oil (48.0 mg, 60%). ^{1}H NMR (400 MHz, $CDCl_3$) δ 8.17 (d, J = 1.8 Hz, 1H), 7.96 (dd, J = 6.2, 1.8 Hz, 1H), 7.54 (dd, J = 6.1, 1.8 Hz, 4H), 7.42 – 7.33 (m, 6H), 7.21 (d, J = 8.0 Hz, 1H), 2.93 (t, J = 6.6 Hz, 2H), 2.08 (p, J = 6.4 Hz, 2H), 1.54 (s, 9H), 1.44 (t, J = 6.4 Hz, 2H). ^{13}C NMR (101

MHz, $CDCl_3$) δ 165.9, 154.2, 137.3, 135.6, 135.4, 135.1, 131.5, 130.2, 129.5, 128.8, 128.0, 80.7, 35.5, 28.2, 22.2 11.3. HRMS (EI): calculated for $C_{26}H_{28}O_2Si$ [M] 400.1859, found 400.1866.

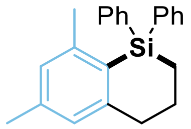

6,8-dimethyl-1,1-diphenyl-1,2,3,4-tetrahydrobenzo[*b*]siline (**6l**)

Following the general procedure for dehydrogenative silacyclization, the product **6l** was isolated by flash chromatography (PE) as colorless oil (32.1 mg, 49%). ^{1}H NMR (400 MHz, $CDCl_3$) δ 7.59 (dd, J = 6.1, 1.7 Hz, 4H), 7.41 – 7.32 (m, 6H), 6.90 (s, 1H), 6.84 (s, 1H), 2.89 (t, J = 6.4 Hz, 2H), 2.31 (s, 3H), 2.00 (s, 3H), 1.95 (p, J = 6.2 Hz, 2H), 1.28 (t, J = 6.6 Hz, 2H). ^{13}C NMR (101 MHz, $CDCl_3$) δ 142.1, 137.7, 135.8, 135.4, 134.2, 129.9, 128.1, 127.9, 127.4, 126.4, 39.3, 29.7, 24.9, 22.3, 14.9. HRMS (ESI): calculated for $C_{23}H_{24}Si$ [M] 328.1647, found 328.1644.

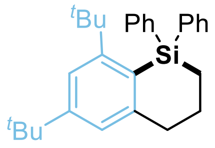

6,8-di-tert-butyl-1,1-diphenyl-1,2,3,4-tetrahydrobenzo[*b*]siline (**6m**)

Following the general procedure for dehydrogenative silacyclization, the product **6m** was isolated by flash chromatography (PE) as colorless oil (27.2 mg, 33%). ^{1}H NMR (400 MHz, $CDCl_3$) δ 7.61 – 7.55 (m, 4H), 7.44 – 7.33 (m, 6H), 6.97 (d, J = 1.8 Hz, 2H), 2.68 – 2.60 (m, 2H), 1.80 (t, J = 8.1 Hz, 2H), 1.30 (s, 18H), 1.24 – 1.20 (m, 2H). ^{13}C NMR (101 MHz, $CDCl_3$) δ 150.47, 141.38, 136.32, 135.67, 134.95, 129.09, 127.84, 127.76, 122.69, 119.70, 40.40, 34.75, 31.53, 25.96, 12.60. HRMS (ESI): calculated for $C_{29}H_{36}SiH^+$ $[M+H]^+$ 413.2586, found 413.2664.

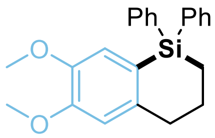

6,7-dimethoxy-1,1-diphenyl-1,2,3,4-tetrahydrobenzo[*b*]siline (**6n**)

Following the general procedure for dehydrogenative silacyclization, the product **6n** was isolated by flash chromatography (PE) as colorless oil (42.5 mg, 59%). ^{1}H NMR (400 MHz, $CDCl_3$) δ 7.56 (dd, *J* = 7.8, 1.7 Hz, 4H), 7.42 – 7.31 (m, 6H), 6.92 (s, 1H), 6.72 (s, 1H), 3.89 (s, 3H), 3.76 (s, 3H), 2.88 – 2.82 (m, 2H), 2.08 – 2.01 (m, 3H), 1.38 – 1.34 (m, 2H). ^{13}C NMR (101 MHz, $CDCl_3$) δ 149.93, 146.84, 143.04, 136.41, 135.22, 129.18, 127.73, 121.33, 117.73, 112.10, 55.95, 55.49, 34.80, 22.48, 11.44. HRMS (EI): calculated for $C_{23}H_{24}O_2Si$ [M] 360.1546, found 360.1544.

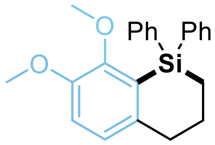

7,8-dimethoxy-1,1-diphenyl-1,2,3,4-tetrahydrobenzo[*b*]siline (**6n′**)

Following the general procedure for dehydrogenative silacyclization, the product **6n′** was isolated by flash chromatography (PE) as colorless oil (13.7 mg, 19%). ^{1}H NMR (400 MHz, $CDCl_3$) ^{1}H NMR (400 MHz, Chloroform-*d*) δ 7.65 – 7.60 (m, 4H), 7.37 – 7.31 (m, 6H), 6.91 (s, 2H), 3.81 (s, 3H), 3.06 (s, 3H), 2.87 – 2.80 (m, 2H), 2.02 – 1.98 (m, 2H), 1.33 – 1.31 (m, 2H). ^{13}C NMR (101 MHz, $CDCl_3$) δ 153.81, 149.89, 142.07, 136.71, 135.60, 129.13, 127.68, 125.42, 124.85, 114.81, 59.46, 55.70, 34.81, 22.80, 13.44. HRMS (EI): calculated for $C_{23}H_{24}O_2Si$ [M] 360.1546, found 360.1544.

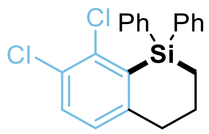

7,8-dichloro-1,1-diphenyl-1,2,3,4-tetrahydrobenzo[*b*]siline (**6o**)

Following the general procedure for dehydrogenative silacyclization, the product **6o** was isolated by flash chromatography (PE) as colorless oil (41.9 mg, 57%). ^{1}H NMR (400 MHz, $CDCl_3$) δ 7.64 – 7.60 (m, 4H), 7.43 – 7.39 (m, 3H), 7.39 – 7.35 (m, 4H), 7.09 (dd, *J* = 8.2, 0.9 Hz, 1H), 2.94 – 2.89 (m, 2H), 2.00 – 1.95 (m, 2H), 1.32 – 1.29 (m, 2H). ^{13}C NMR (101 MHz, $CDCl_3$) δ 150.51, 139.80, 135.61, 134.85, 133.34, 131.60, 130.62, 129.58, 129.30, 127.94, 35.84, 21.83, 14.71. HRMS (EI): calculated for $C_{21}H_{18}Cl_2Si$ [M] 368.0555, found 368.0544.

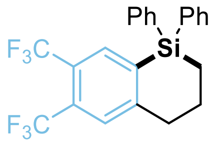

1,1-diphenyl-6,7-bis(trifluoromethyl)-1,2,3,4-tetrahydrobenzo[*b*]siline (**6p**)

Following the general procedure for dehydrogenative silacyclization, the product **6p** was isolated by flash chromatography (PE) as colorless oil (47.1 mg, 54%). ^{1}H NMR (400 MHz, $CDCl_3$) δ 7.79 (s, 1H), 7.66 (s, 1H), 7.48 (dd, *J* = 5.4, 1.2 Hz, 4H), 7.43 – 7.33 (m, 6H), 3.03 (t, *J* = 6.1 Hz, 2H), 1.98 (p, *J* = 5.9 Hz, 2H), 1.36 (t, *J* = 6.2 Hz, 2H). ^{19}F NMR (376 MHz, $CDCl_3$) δ -56.67 (s, 1F), -63.37 (s, 1F). ^{13}C NMR (101 MHz, $CDCl_3$) δ 157.4 – 156.6 (m), 146.5– 145.9 (m), 137.9 – 137.7 (m), 135.3, 134.9, 129.6, 129.4, 127.7, 123.2 – 122.7 (m), 121.5 – 121.1 (m), 36.9, 21.2, 14.6. HRMS (ESI): calculated for $C_{23}H_{18}F_6Si$ [M] 436.1082, found 436.1081.

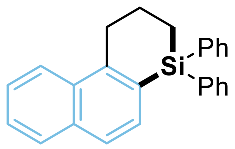

4,4-diphenyl-1,2,3,4-tetrahydronaphtho[2,1-*b*]siline (**6q**)

Following the general procedure for dehydrogenative silacyclization, the product **6q** was isolated by flash chromatography (PE) as colorless oil (45.5 mg, 65%). ^{1}H NMR

(400 MHz, $CDCl_3$) δ 8.17 (d, *J* = 7.9, 1H), 7.83 (dd, *J* = 6.6, 1.8 Hz, 1H), 7.68 (d, *J* = 8.1, 1H), 7.61 – 7.50 (m, 7H), 7.42 – 7.34 (m, 6H), 6.92 (s, 1H), 3.41 (t, *J* = 6.2 Hz, 2H), 2.25 (p, *J* = 6.4 Hz, 2H), 1.46 (t, *J* = 6.0 Hz, 2H). ^{13}C NMR (101 MHz, $CDCl_3$) δ 146.4, 135.9, 135.6, 135.2, 134.1, 132.0, 131.8, 129.4, 128.8, 128.3, 127.9, 126.2, 125.5, 123.5, 30.3, 21.8, 10.8. HRMS (EI): calculated for $C_{25}H_{22}Si$ [M] 350.1491, found 350.1487.

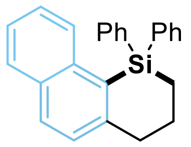

1,1-diphenyl-1,2,3,4-tetrahydronaphtho[1,2-*b*]siline (**6r**)

Following the general procedure for dehydrogenative silacyclization, the product **6r** was isolated by flash chromatography (PE) as colorless oil (30.8 mg, 44%). ^{1}H NMR (400 MHz, $CDCl_3$) δ 7.85 (d, *J* = 8.4 Hz, 1H), 7.82 (d, *J* = 8.5 Hz, 1H), 7.79 (d, *J* = 8.1 Hz, 1H), 7.66 – 7.60 (m, 4H), 7.40 – 7.30 (m, 8H), 7.14 (t, *J* = 7.7 Hz, 1H), 3.17 – 3.12 (m, 2H), 2.11 – 2.06 (m, 2H), 1.43 – 1.38 (m, 2H). ^{13}C NMR (101 MHz, $CDCl_3$) δ 150.12, 137.89, 136.61, 135.58, 132.14, 130.31, 129.54, 129.35, 128.96, 128.52, 128.02, 127.17, 125.59, 124.81, 36.84, 21.88, 14.79. HRMS (EI): calculated for $C_{25}H_{22}Si$ [M] 350.1491, found 350.1498.

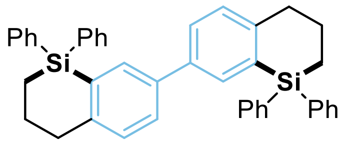

1,1,1',1'-tetraphenyl-1,1',2,2',3,3',4,4'-octahydro-7,7'-bibenzo[*b*]siline (**6s**)

Following the general procedure for dehydrogenative silacyclization, the product **6s** was isolated by flash chromatography (PE) as colorless oil (56.2 mg, 47%). ^{1}H NMR (400 MHz, $CDCl_3$) δ 7.64 – 7.53 (m, 10H), 7.43 – 7.28 (m, 14H), 7.21 – 7.14 (m, 2H),

2.91 (t, J = 6.2 Hz, 2H), 2.09 (p, J = 6.6 Hz, 2H), 1.44 (t, J = 6.2 Hz, 2H). ^{13}C NMR (101 MHz, $CDCl_3$) δ 148.3, 138.1, 135.3, 134.7, 131.6, 129.3, 129.2, 128.0, 127.9, 126.9, 34.9, 22.6, 11.3. HRMS (EI): calculated for $C_{42}H_{38}Si_2$ [M] 598.2512, found 598.2519.

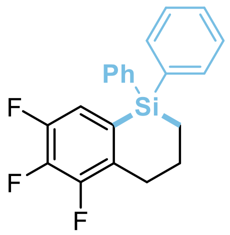

5,6,7-trifluoro-1,1-diphenyl-1,2,3,4-tetrahydrobenzo[*b*]siline (**6t**)

Following the general procedure for dehydrogenative silacyclization, the product **6t** was isolated by flash chromatography (PE) as colorless oil (41.8 mg, 59%). ^{1}H NMR (400 MHz, $CDCl_3$) δ 7.53 – 7.48 (m, 4H), 7.45 – 7.35 (m, 6H), 7.03 – 6.94 (m, 1H), 2.86 (t, J = 6.2 Hz, 2H), 2.06 (p, J = 6.4 Hz, 2H), 1.41 (t, J = 6.0 Hz, 2H). ^{13}C NMR (101 MHz, $CDCl_3$) δ 135.3, 135.2, 134.5, 129.9, 129.7, 128.1, 128.0, 118.3 – 118.1 (m), 117.4 – 117.2 (m), 104.7 – 104.3 (m), 25.9, 21.1, 10.6. ^{19}F NMR (376 MHz, $CDCl_3$) δ -113.05 – -114.24 (m, 1F), -137.87 – -138.38 (m, 1F), -150.09 (s, 1F). HRMS (EI): calculated for $C_{21}H_{17}F_3Si$ [M] 354.1052, found 354.1054.

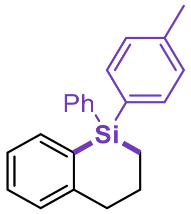

1-phenyl-1-(p-tolyl)-1,2,3,4-tetrahydrobenzo[*b*]siline (**7a**)

Following the general procedure for dehydrogenative silacyclization, the product **7a** was isolated by flash chromatography (PE) as colorless oil (27.6 mg, 44%). ^{1}H NMR (400 MHz, $CDCl_3$) δ 7.58 – 7.51 (m, 2H), 7.45 (dd, J = 15.3, 7.4 Hz, 3H), 7.38 – 7.26

(m, 4H), 7.17 (t, J = 6.0 Hz, 4H), 2.98 – 2.78 (m, 2H), 2.34 (s, 3H), 2.06 (p, J = 6.5 Hz, 2H), 1.42 – 1.36 (m, 2H). ^{13}C NMR (101 MHz, $CDCl_3$) δ 149.46, 139.30, 136.53, 136.21, 135.45, 135.38, 132.56, 131.37, 129.30, 129.28, 128.84, 128.72, 127.81, 125.43, 35.34, 22.53, 21.54, 11.63. HRMS (ESI): calculated for $C_{22}H_{22}SiH^+$ $[M+H]^+$ 315.1564, found 315.1569.

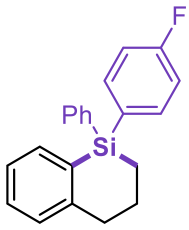

1-(4-fluorophenyl)-1-phenyl-1,2,3,4-tetrahydrobenzo[*b*]siline (**7b**)

Following the general procedure for dehydrogenative silacyclization, the product **7b** was isolated by flash chromatography (PE) as colorless oil (32.5 mg, 51%). ^{1}H NMR (400 MHz, $CDCl_3$) δ 7.56 – 7.44 (m, 5H), 7.41 – 7.29 (m, 4H), 7.21 – 7.16 (m, 2H), 7.04 (t, J = 8.9 Hz, 2H), 2.95 – 2.85 (m, 2H), 2.15 – 2.00 (m, 2H), 1.47 – 1.35 (m, 2H). ^{13}C NMR (101 MHz, $CDCl_3$) δ 163.95 (d, J = 248.7 Hz), 149.48, 137.36 (d, J = 7.5 Hz), 136.11, 136.00, 135.31, 131.80 (d, J = 3.7 Hz), 130.89, 129.50, 129.48, 128.96, 127.93, 125.53, 115.07 (d, J = 19.7 Hz, 1F), 35.26, 22.45, 11.60. HRMS (ESI): calculated for $C_{21}H_{19}SiFH^+$ $[M+H]^+$ 319.1313, found 315.1318.

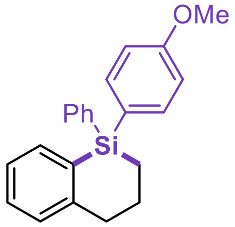

1-(4-methoxyphenyl)-1-phenyl-1,2,3,4-tetrahydrobenzo[*b*]siline (**7c**)

Following the general procedure for dehydrogenative silacyclization, the product **7c**

was isolated by flash chromatography (PE) as colorless oil (37.0 mg, 56%). ^{1}H NMR (400 MHz, $CDCl_3$) δ 7.59 – 7.55 (m, 2H), 7.52 – 7.48 (m, 3H), 7.43 – 7.34 (m, 3H), 7.34 – 7.30 (m, 1H), 7.23 – 7.18 (m, 2H), 6.95 – 6.91 (m, 2H), 3.83 (s, 3H), 2.95 – 2.88 (m, 2H), 2.10 (dtdd, *J* = 11.5, 5.7, 2.9, 1.3 Hz, 2H), 1.45 – 1.38 (m, 2H). ^{13}C NMR (101 MHz, $CDCl_3$) δ 160.83, 149.55, 137.01, 136.80, 136.29, 135.48, 131.66, 129.40, 129.39, 128.95, 127.93, 127.08, 125.54, 113.78, 55.14, 35.45, 22.65, 11.84. HRMS (EI): calculated for $C_{22}H_{22}OSi$ [M] 330.1434, found 330.1433.

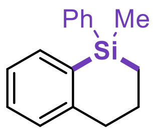

1-methyl-1-phenyl-1,2,3,4-tetrahydrobenzo[*b*]siline (**7d**)

Following the general procedure for dehydrogenative silacyclization, the product **7d** was isolated by flash chromatography (PE) as colorless oil (24.3 mg, 51%). ^{1}H NMR (400 MHz, $CDCl_3$) δ 7.53 (dt, *J* = 7.7, 1.8 Hz, 2H), 7.44 (dt, *J* = 7.2, 1.8 Hz, 1H), 7.39 – 7.32 (m, 3H), 7.29 (tt, *J* = 7.5, 1.5 Hz, 1H), 7.22 – 7.14 (m, 2H), 2.86 (dd, *J* = 7.2, 4.3 Hz, 2H), 2.02 (ddtd, *J* = 11.9, 10.0, 5.1, 1.8 Hz, 2H), 1.24 – 1.03 (m, 2H), 0.55 (d, *J* = 1.7 Hz, 3H). ^{13}C NMR (101 MHz, $CDCl_3$) δ 149.21, 138.79, 135.24, 134.46, 133.29, 129.21, 129.18, 128.81, 127.91, 125.59, 35.49, 22.62, 12.61, -2.48. HRMS (EI): calculated for $C_{16}H_{18}Si$ [M] 238.1172, found 238.1178.

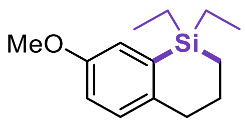

1,1-diethyl-7-methoxy-1,2,3,4-tetrahydrobenzo[*b*]siline (**7e**)

Following the general procedure for dehydrogenative silacyclization, the product **7e** was isolated by flash chromatography (PE) as colorless oil (7.0 mg, 15%). ^{1}H NMR (400 MHz, $CDCl_3$) δ 7.07 – 7.01 (m, 1H), 6.96 (d, *J* = 2.9 Hz, 1H), 6.80 (dd, *J* = 8.4,

2.9 Hz, 1H), 3.80 (s, 3H), 2.70 – 2.63 (m, 2H), 1.93 – 1.83 (m, 2H), 0.95 (t, *J* = 7.8 Hz, 6H), 0.89 – 0.82 (m, 2H), 0.73 (qd, *J* = 7.8, 0.8 Hz, 4H). ^{13}C NMR (101 MHz, $CDCl_3$) δ 157.09, 141.61, 135.12, 129.60, 119.50, 114.03, 55.32, 34.73, 23.31, 8.64, 7.65, 5.80. HRMS (EI): calculated for $C_{14}H_{22}OSi$ [M] 234.1434, found 234.1436.

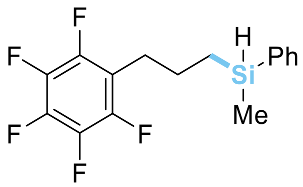

methyl(3-(perfluorophenyl)propyl)(phenyl)silane (**I-1**)

Following the procedure in Fig. S2, the intermediate of defluorosilacyclization **I-1** was isolated by flash chromatography (PE) as colorless oil (48.8 mg, 74%). ^{1}H NMR (400 MHz, $CDCl_3$) δ 7.51 (d, *J* = 6.2 Hz, 2H), 7.41 – 7.35 (m, 3H), 4.36 (m, 1H), 2.73 (t, *J* = 5.8 Hz, 2H), 1.67 (p, *J* = 6.0 Hz, 2H), 0.87 (t, *J* = 5.6 Hz, 2H), 0.35 (d, *J* = 3.6 Hz, 3H). ^{13}C NMR (101 MHz, $CDCl_3$) δ 146.4 – 146.1 (m), 144.1 – 143.7 (m), 138.6 – 138.2 (m), 135.8, 134.2, 129.4, 127.9, 115.2 – 114.8 (m), 25.5, 24.3, 13.2, 1.0. ^{19}F NMR (376 MHz, $CDCl_3$) δ -144.20 – -144.29 (m, 2F), -158.09 – -158.20 (m, 1F), -162.99 – -163.12 (m, 2F). HRMS (EI): calculated for $C_{16}H_{15}F_5Si$ [M] 330.0863, found 330.0866.

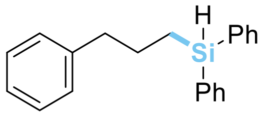

diphenyl(3-phenylpropyl)silane (**II-1**)

Following the procedure in Fig. S6, the intermediate of dehydrogenative silacyclization **II-1** was isolated by flash chromatography (PE) as colorless oil (51.3 mg, 85%). ^{1}H NMR (400 MHz, $CDCl_3$) δ 7.68 (d, *J* = 6.2 Hz, 4H), 7.53 – 7.47 (m, 6H), 7.39 (t, *J* = 5.8 Hz, 2H), 7.33 – 7.26 (m, 3H), 5.02 (t, *J* = 2.9 Hz, 2H), 2.81 (t, *J* =

5.4 Hz, 2H), 1.93 (p, *J* = 5.6 Hz, 2H), 1.32 (t, *J* = 5.86 Hz, 2H). ^{13}C NMR (101 MHz, $CDCl_3$) δ ^{13}C NMR (126 MHz, Chloroform-d) δ 142.2, 135,2, 134.4, 129.6, 129.3, 128.6, 128.3, 128.1, 125.8, 39.3, 26.4, 11.9. HRMS (EI): calculated for $C_{21}H_{20}Si$ [M] 302.1491, found 302.1484.

IX. References

[1] E. E. Swayze, P. P. Seth, R. H. Griffey, E. A. Jefferson, **2005**, Benzimidazoles and analogs preparation as antiviral agents. *U.S. Patent Application US20050124638 A1*.

[2] K. Wang, J. Zhou, Y. Jiang, M. Zhang, C. Wang, D. Xue, W. Tang, H. Sun, J. Xiao, C. Li, Selective Manganese-Catalyzed Oxidation of Hydrosilanes to Silanols under Neutral Reaction Conditions. *Angew. Chem. Int. Ed. Engl.* **2019**, *58*, 6380-6384.

[3] Gaussian 16, Revision A.03, M. J. Frisch, G. W. Trucks, H. B. Schlegel, G. E. Scuseria, M. A. Robb, J. R. Cheeseman, G. Scalmani, V. Barone, G. A. Petersson, H. Nakatsuji, X. Li, M. Caricato, A. V. Marenich, J. Bloino, B. G. Janesko, R. Gomperts, B. Mennucci, H. P. Hratchian, J. V. Ortiz, A. F. Izmaylov, J. L. Sonnenberg, D. Williams-Young, F. Ding, F. Lipparini, F. Egidi, J. Goings, B. Peng, A. Petrone, T. Henderson, D. Ranasinghe, V. G. Zakrzewski, J. Gao, N. Rega, G. Zheng, W. Liang, M. Hada, M. Ehara, K. Toyota, R. Fukuda, J. Hasegawa, M. Ishida, T. Nakajima, Y. Honda, O. Kitao, H. Nakai, T. Vreven, K. Throssell, J. A. Montgomery, Jr., J. E. Peralta, F. Ogliaro, M. J. Bearpark, J. J. Heyd, E. N. Brothers, K. N. Kudin, V. N. Staroverov, T. A. Keith, R. Kobayashi, J. Normand, K. Raghavachari, A. P. Rendell, J. C. Burant, S. S. Iyengar, J. Tomasi, M. Cossi, J. M. Millam, M. Klene, C. Adamo, R. Cammi, J. W. Ochterski, R. L. Martin, K. Morokuma, O. Farkas, J. B. Foresman, and D. J. Fox, Gaussian, Inc., Wallingford CT, 2016.

[4] Y. Fu, L. Liu, H.-Z. Yu, Y.-M. Wang, Q.-X. Guo, Quantum-Chemical Predictions of Absolute Standard Redox Potentials of Diverse Organic Molecules and Free Radicals in Acetonitrile. *J. Am. Chem. Soc.* **2005**, *127*, 7227–7234.

[5] T. B. Demissie, K. Ruud, J. H. Hansen, DFT as a Powerful Predictive Tool in Photoredox Catalysis: Redox Potentials and Mechanistic Analysis. *Organometallics* **2015**, *34*, 4218–4228.

[6] A. A. Isse, A. Gennaro, Absolute Potential of the Standard Hydrogen Electrode and the Problem of Interconversion of Potentials in Different Solvents. *J. Phys. Chem. B* **2010**, *114*, 7894–7899.

[7] W. Yang, W. J. Mortier, The use of global and local molecular parameters for the analysis of the gas-phase basicity and proton affinity of amines. *J. Am. Chem. Soc.* **1986**, 108, 5708–5711.

SUPPORTING INFORMATION

X. ^{1}H, ^{13}C and ^{19}F NMR Spectra for New Compounds

5,6,7,8-tetrafluoro-1-methyl-1-phenyl-1,2,3,4-tetrahydrobenzo[*b*]siline **(3a)**

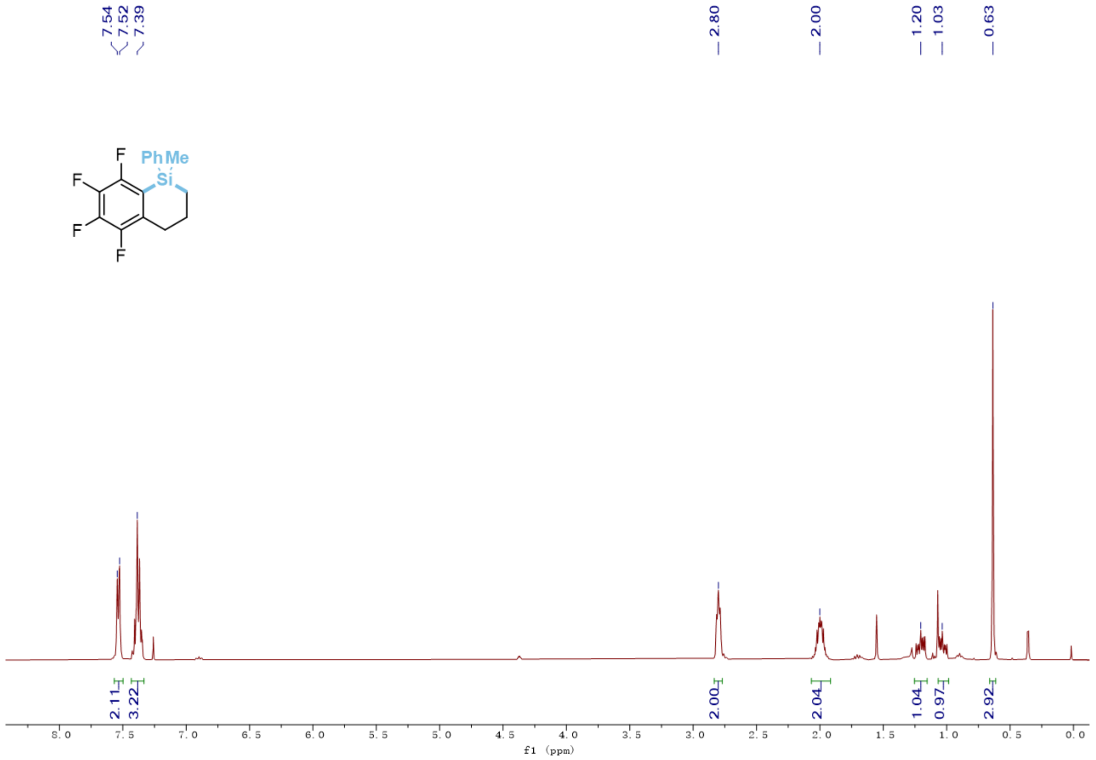

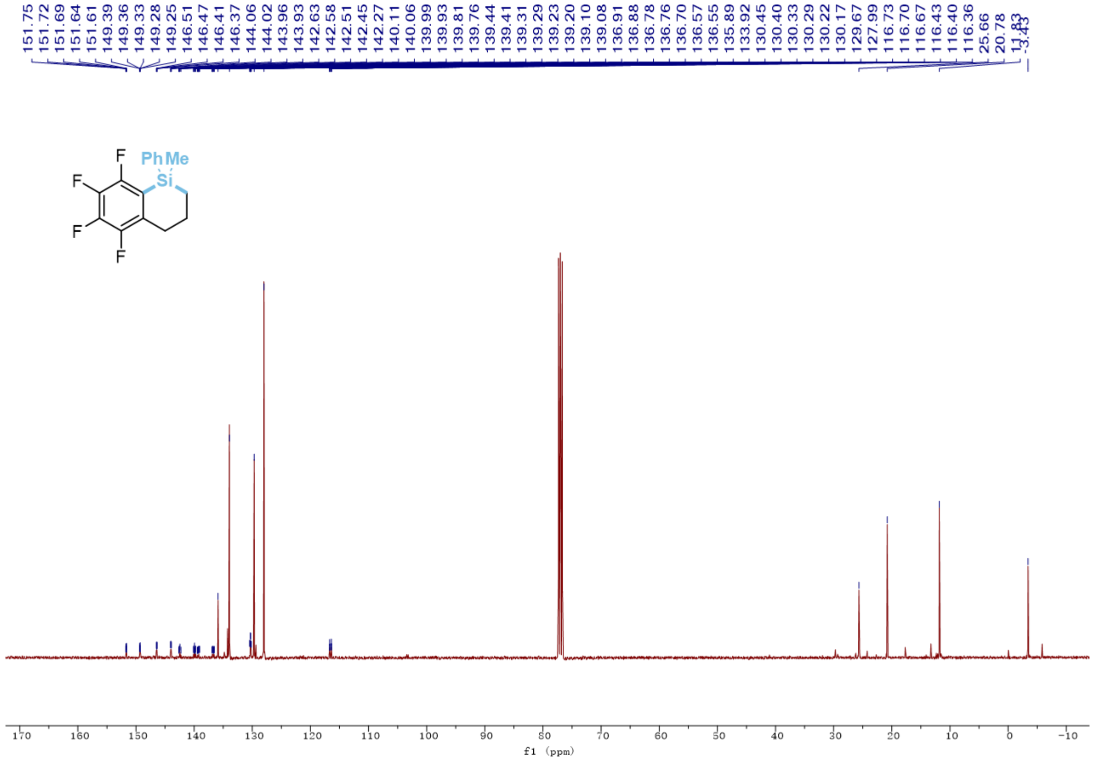

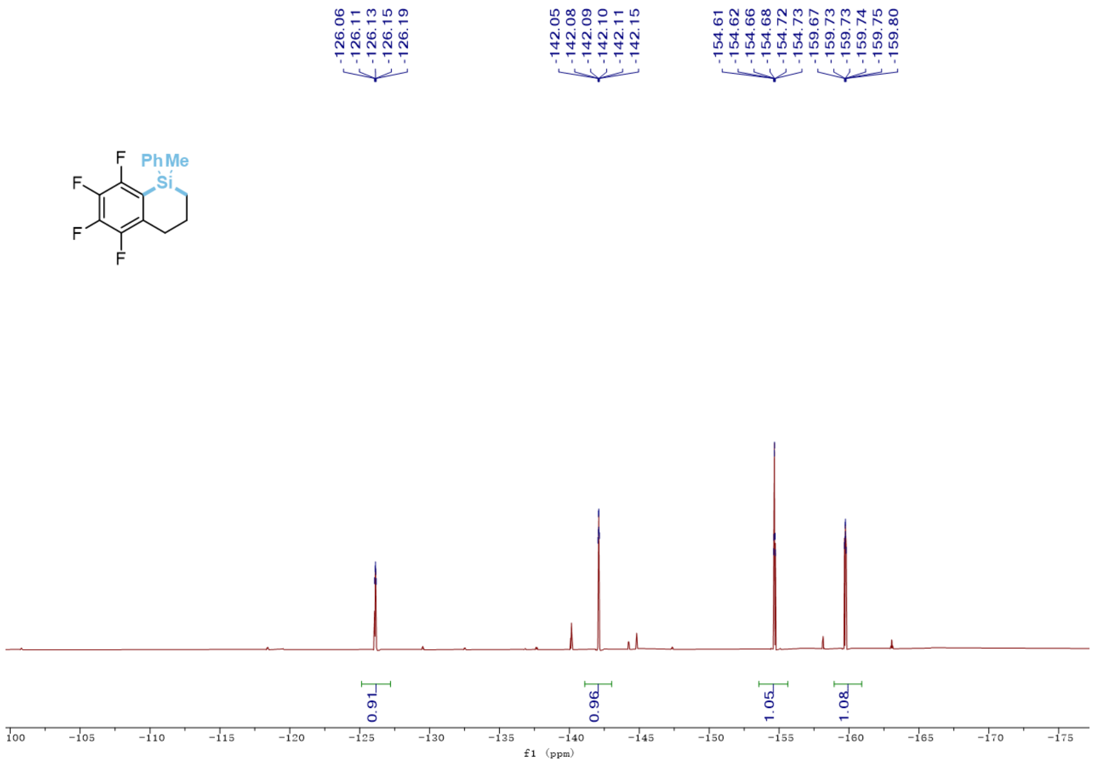

5,6,7,8-tetrafluoro-1,1-diphenyl-1,2,3,4-tetrahydrobenzo[*b*]siline (**3b**)

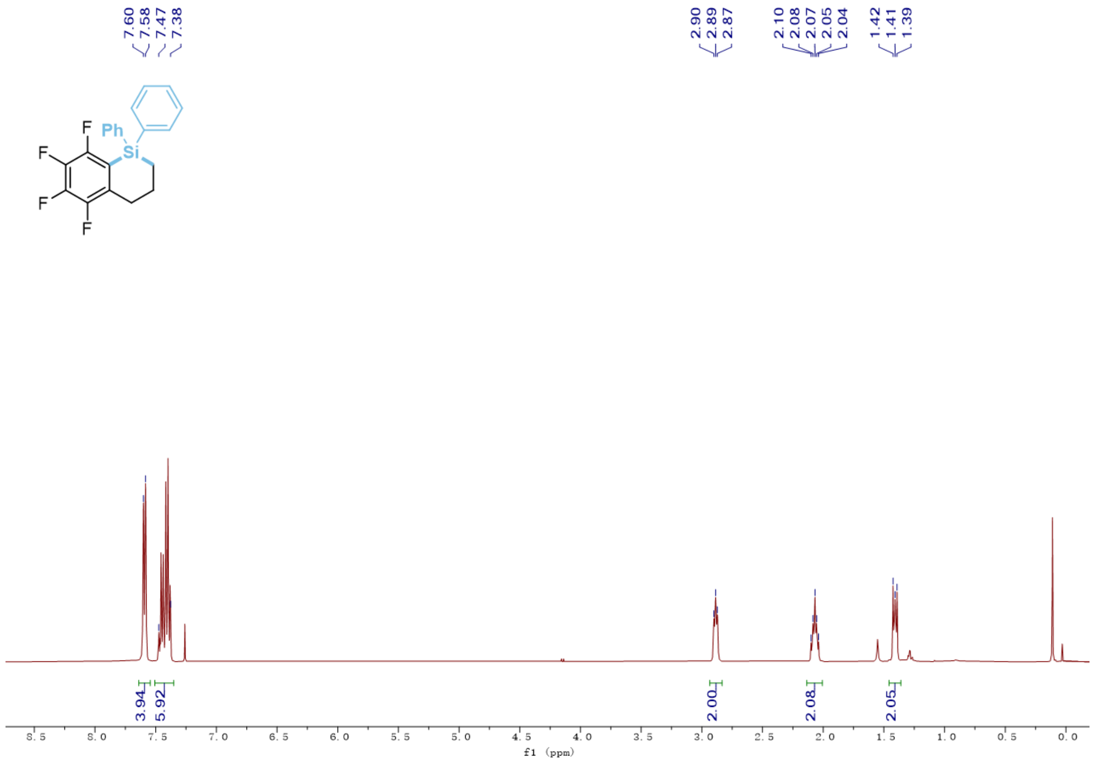

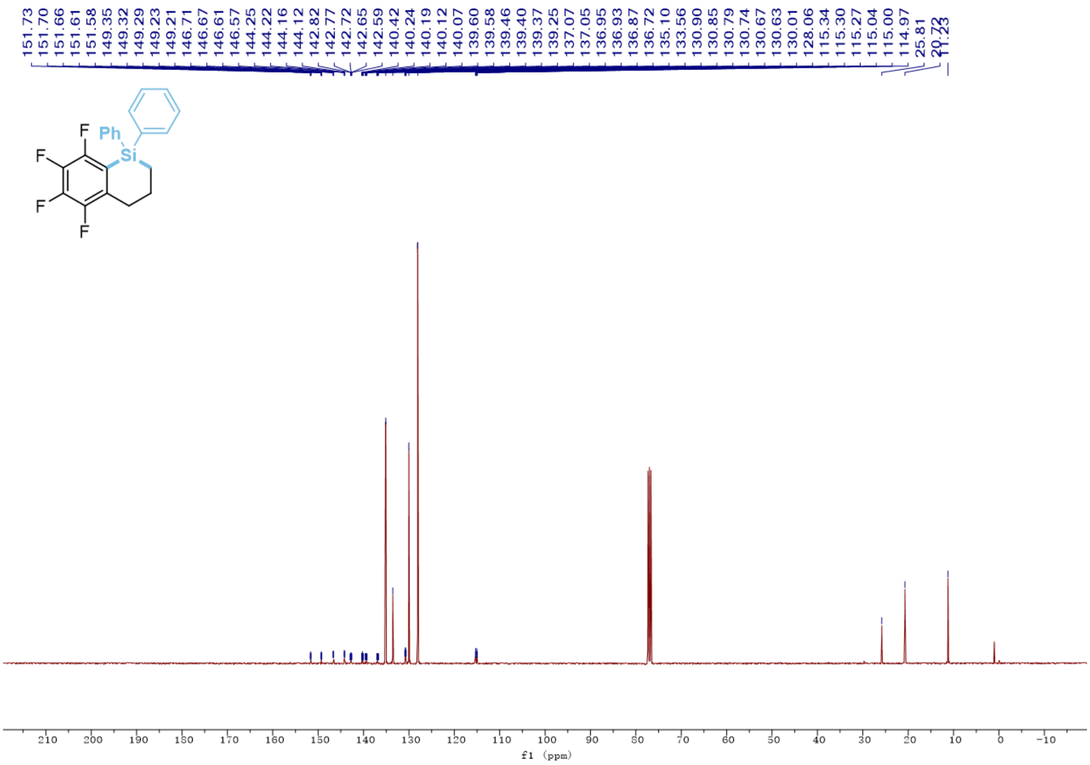

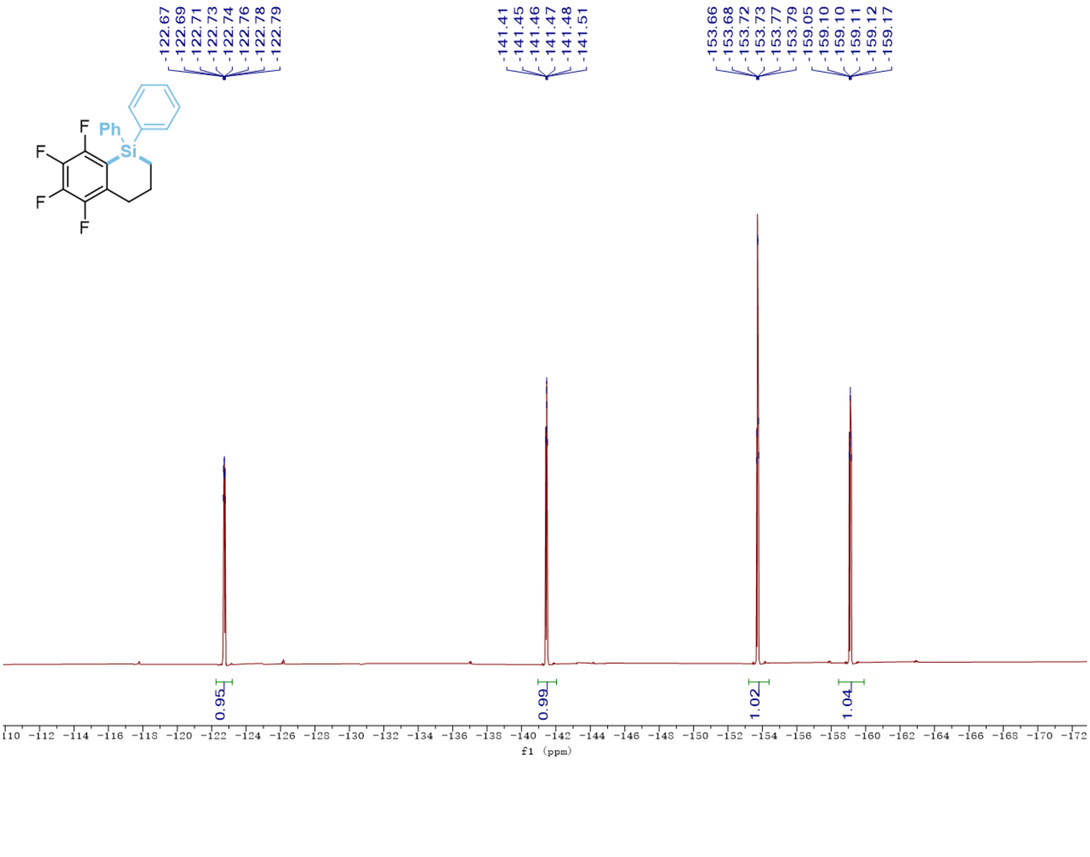

5,6,7,8-tetrafluoro-1-phenyl-1-(o-tolyl)-1,2,3,4-tetrahydrobenzo[*b*]siline (**3c**)

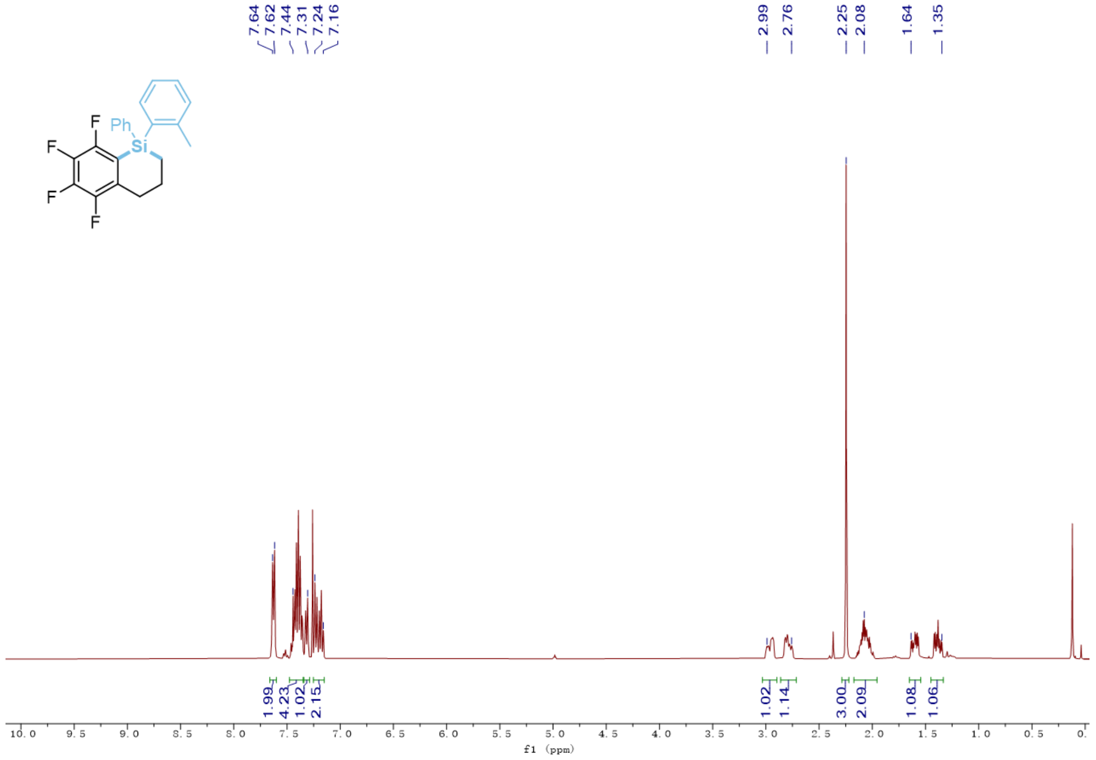

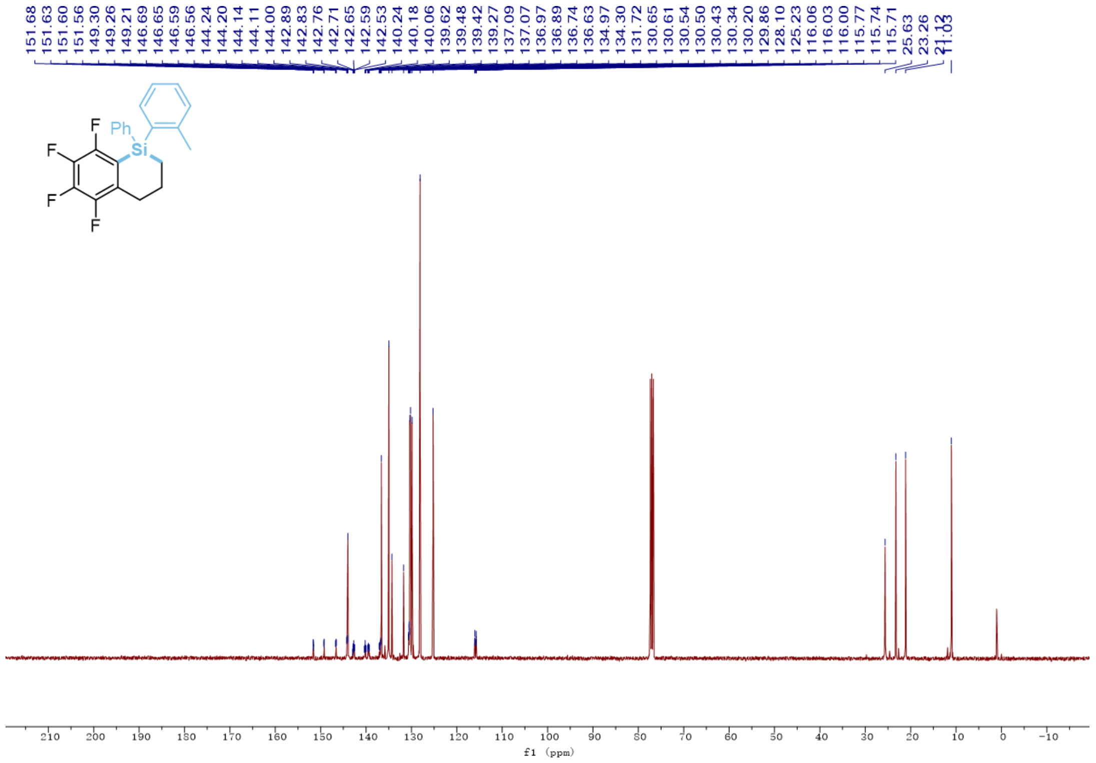

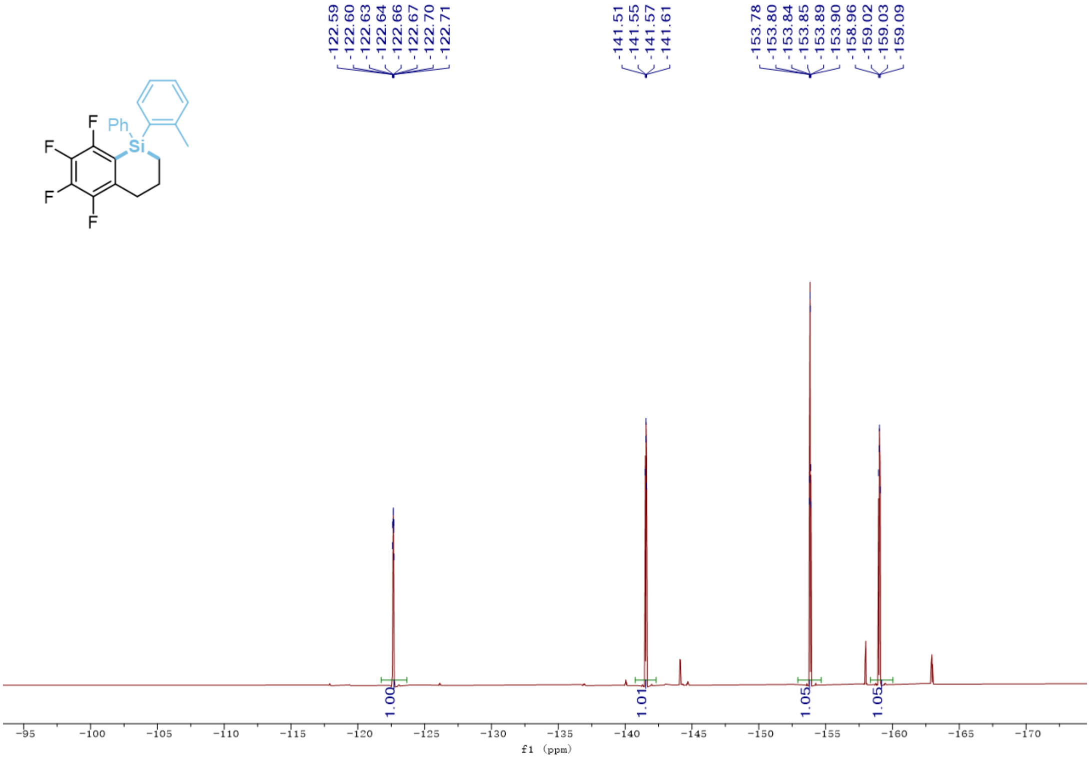

5,6,7,8-tetrafluoro-1-phenyl-1-(m-tolyl)-1,2,3,4-tetrahydrobenzo[*b*]siline (**3d**)

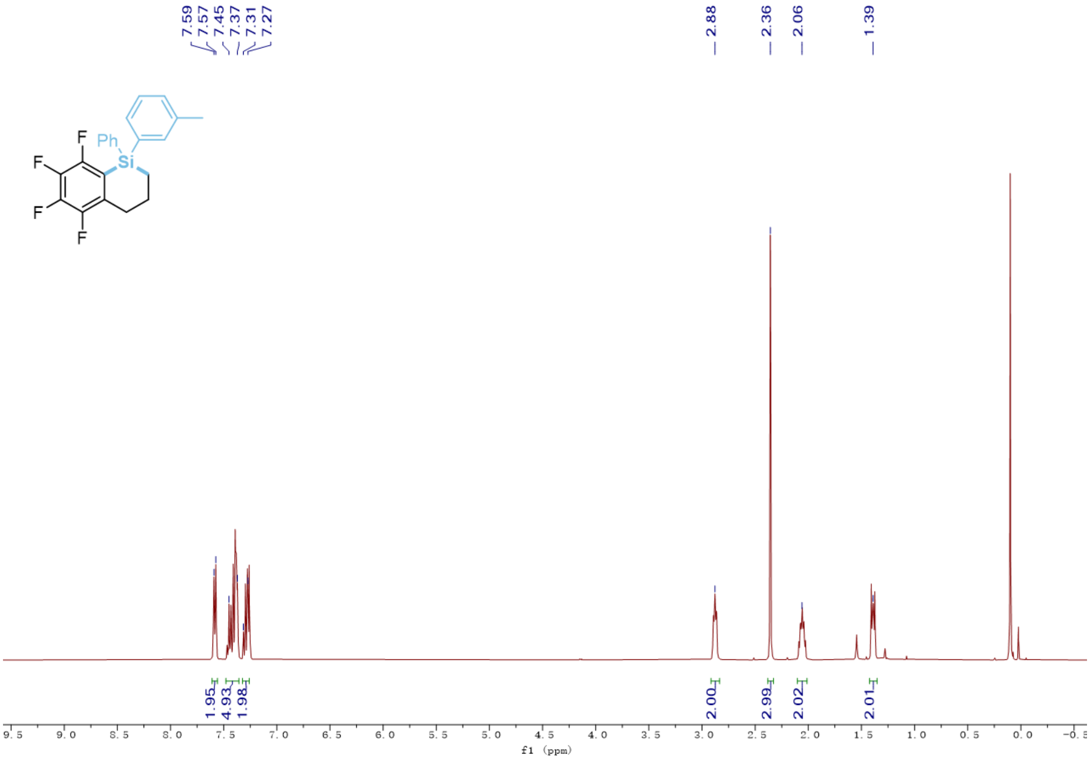

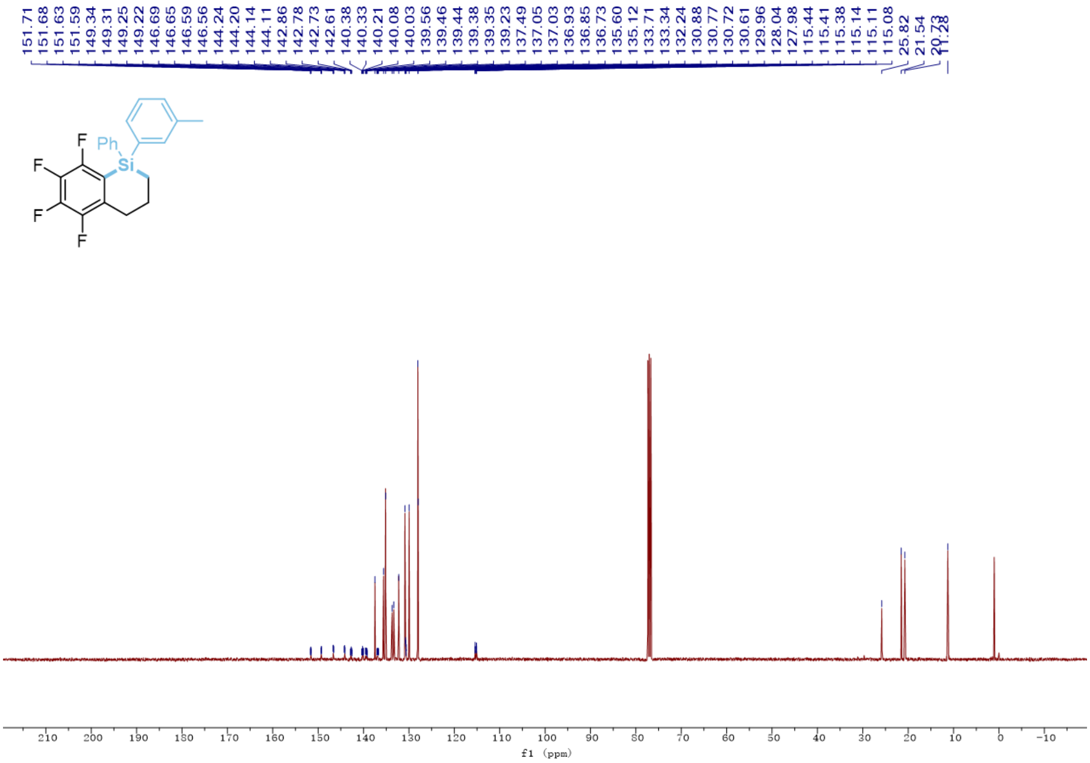
151.71
151.68
151.63
151.59
149.34
149.31
149.25
149.22
146.69
146.65
146.59
146.56
144.24
144.20
144.14
144.11
142.86
142.78
142.73
142.61
140.38
140.33
140.21
140.08
140.03
139.56
139.46
139.44
139.38
139.35
139.23
137.49
137.05
137.03
136.93
136.85
136.73
135.60
135.12
133.71
133.34
132.24
130.88
130.77
130.72
130.61
129.96
128.04
127.98
115.44
115.41
115.38
115.14
115.11
115.08
25.82
21.54
20.73
11.28
F
F
F
F
Ph
Si
f1 (ppm)

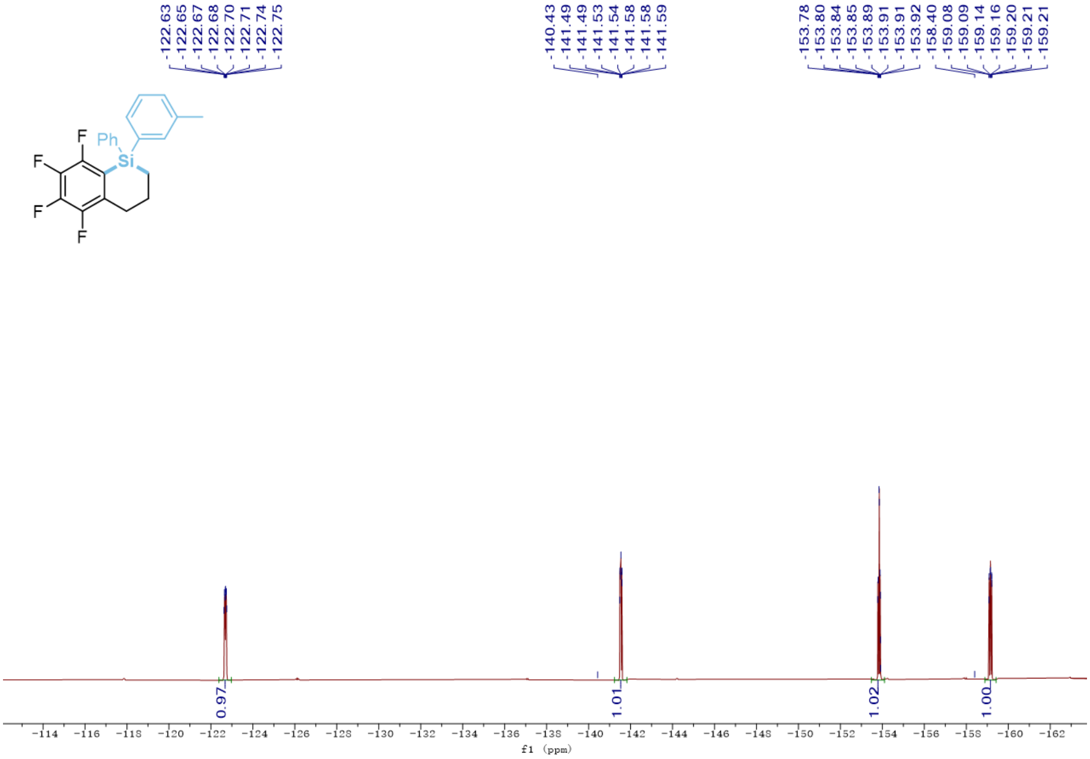
-122.63
-122.65
-122.67
-122.68
-122.70
-122.71
-122.74
-122.75
-140.43
-141.49
-141.49
-141.53
-141.54
-141.58
-141.58
-141.59
-153.78
-153.80
-153.84
-153.85
-153.89
-153.91
-153.91
-153.92
-158.40
-159.08
-159.09
-159.14
-159.16
-159.20
-159.21
-159.21
F
F
F
F
Ph
Si
0.97
1.01
1.02
1.00
f1 (ppm)

SUPPORTING INFORMATION

5,6,7,8-tetrafluoro-1-phenyl-1-(p-tolyl)-1,2,3,4-tetrahydrobenzo[*b*]siline (**3e**)

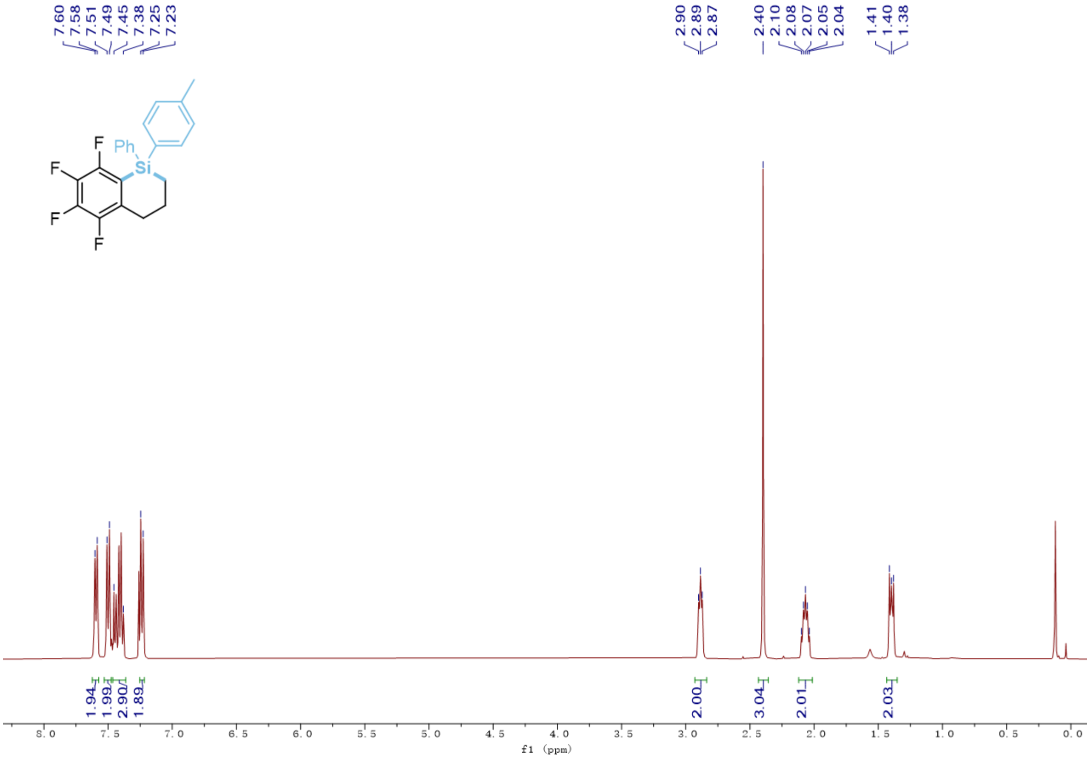

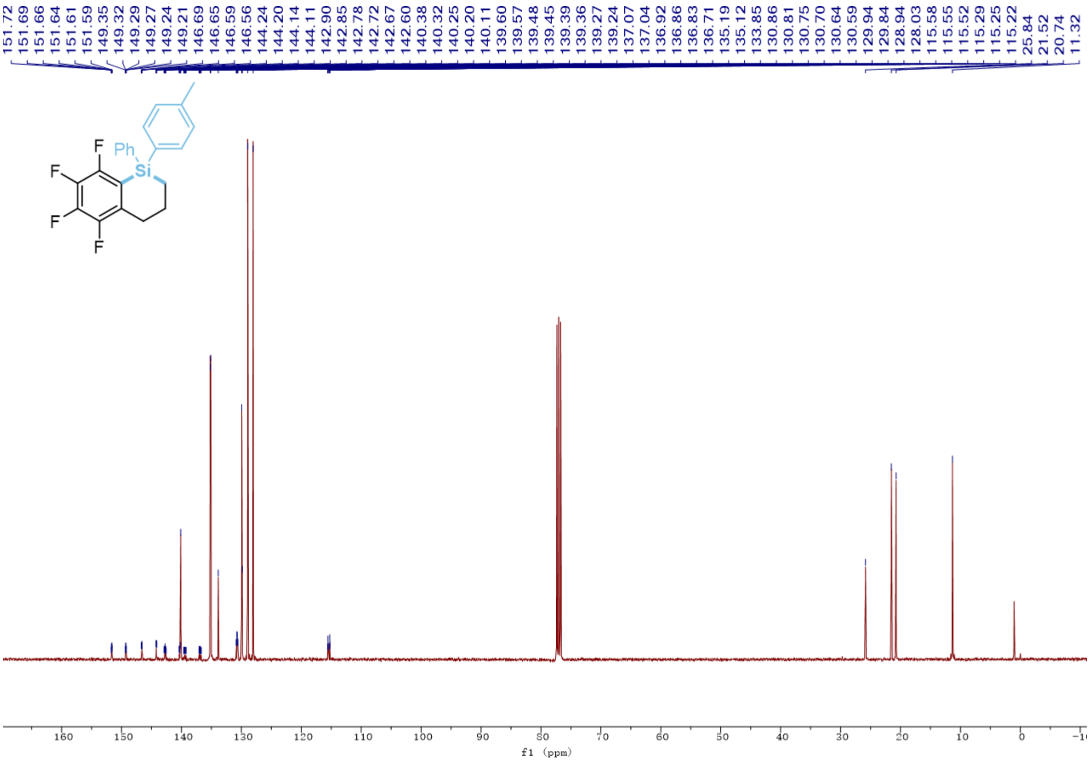

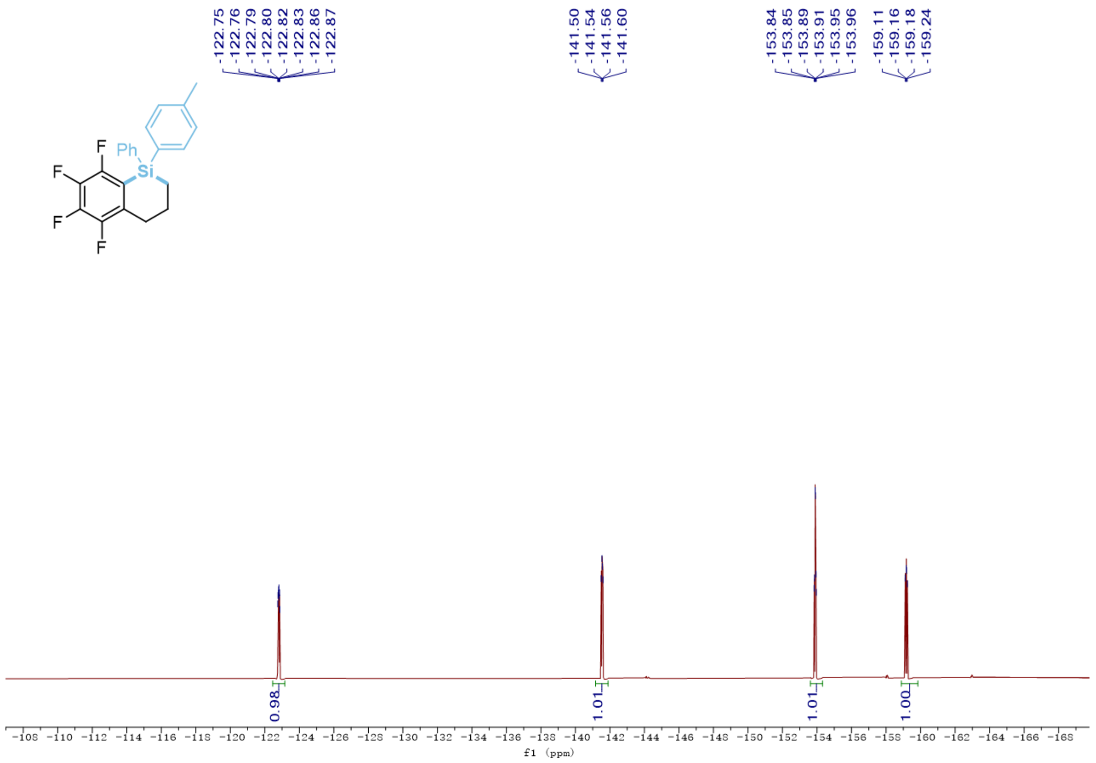

1-(3-chlorophenyl)-5,6,7,8-tetrafluoro-1-phenyl-1,2,3,4-tetrahydrobenzo[*b*]siline (**3f**)

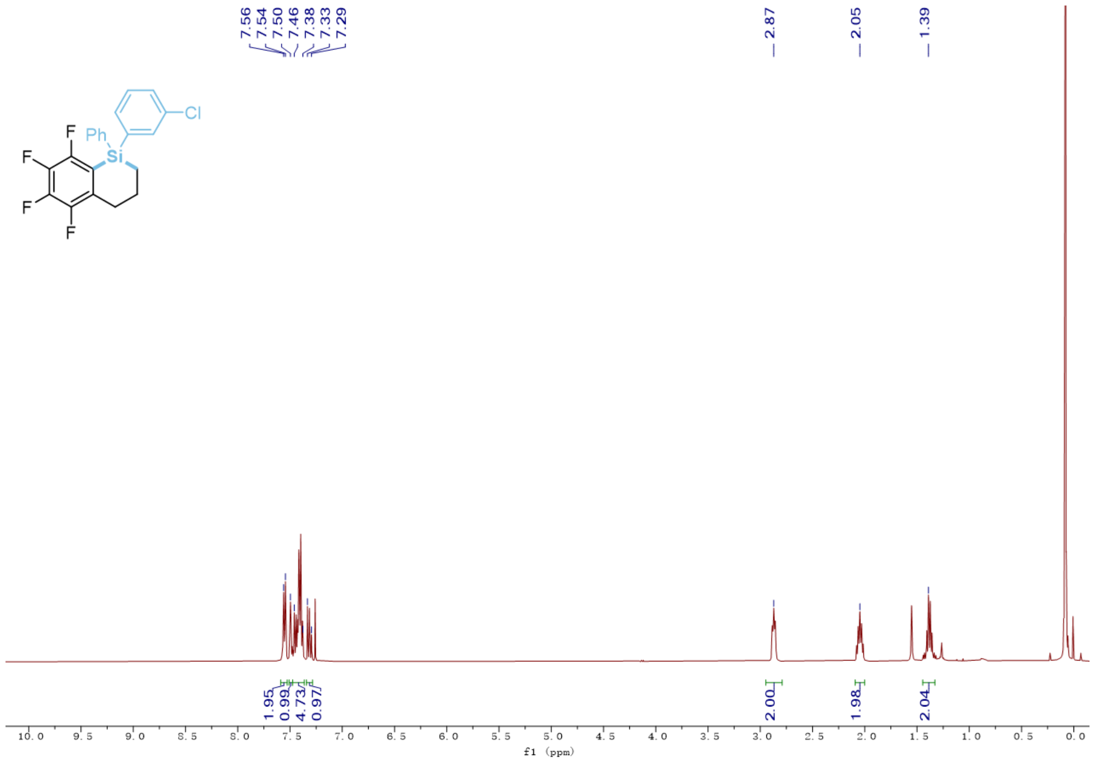

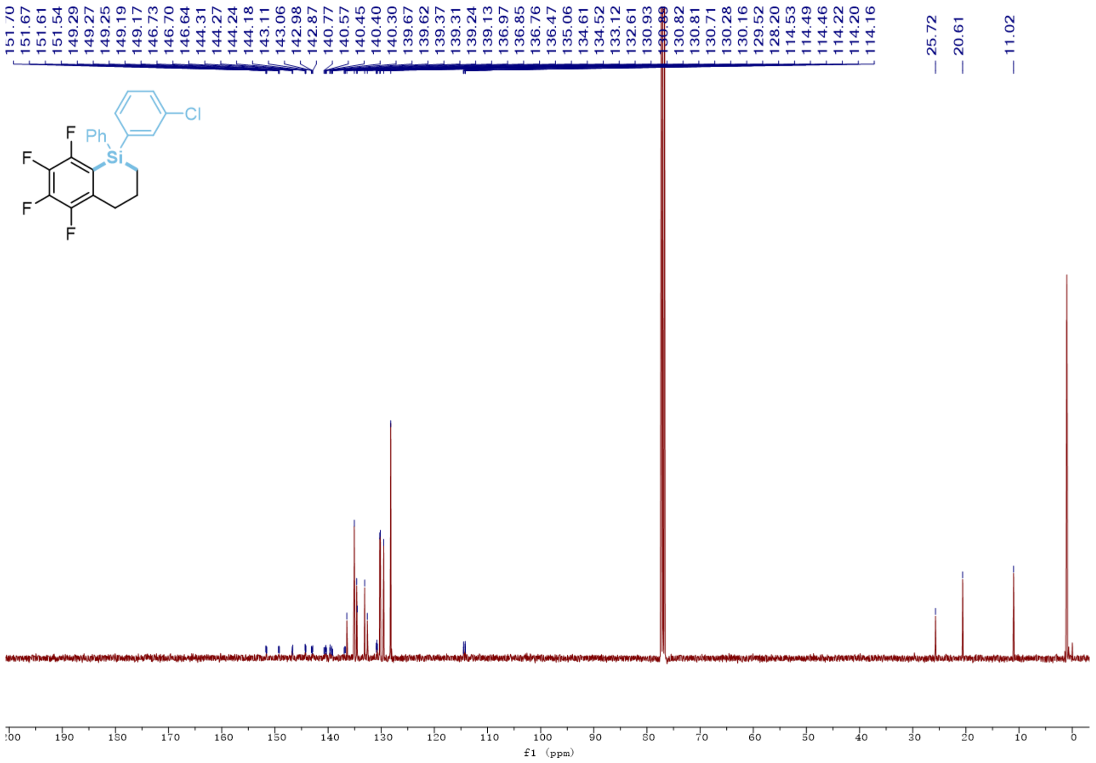
151.70
151.67
151.61
151.54
149.29
149.27
149.25
149.19
149.17
146.73
146.70
146.64
144.31
144.27
144.24
144.18
143.11
143.06
142.98
142.87
140.77
140.57
140.45
140.40
140.30
139.67
139.62
139.37
139.31
139.24
139.13
136.97
136.85
136.76
136.47
135.06
134.61
134.52
133.12
132.61
130.93
130.82
130.81
130.71
130.28
130.16
129.52
128.20
114.53
114.49
114.46
114.22
114.20
114.16
25.72
20.61
11.02
F
F
F
F
Ph
Si
Cl
f1 (ppm)

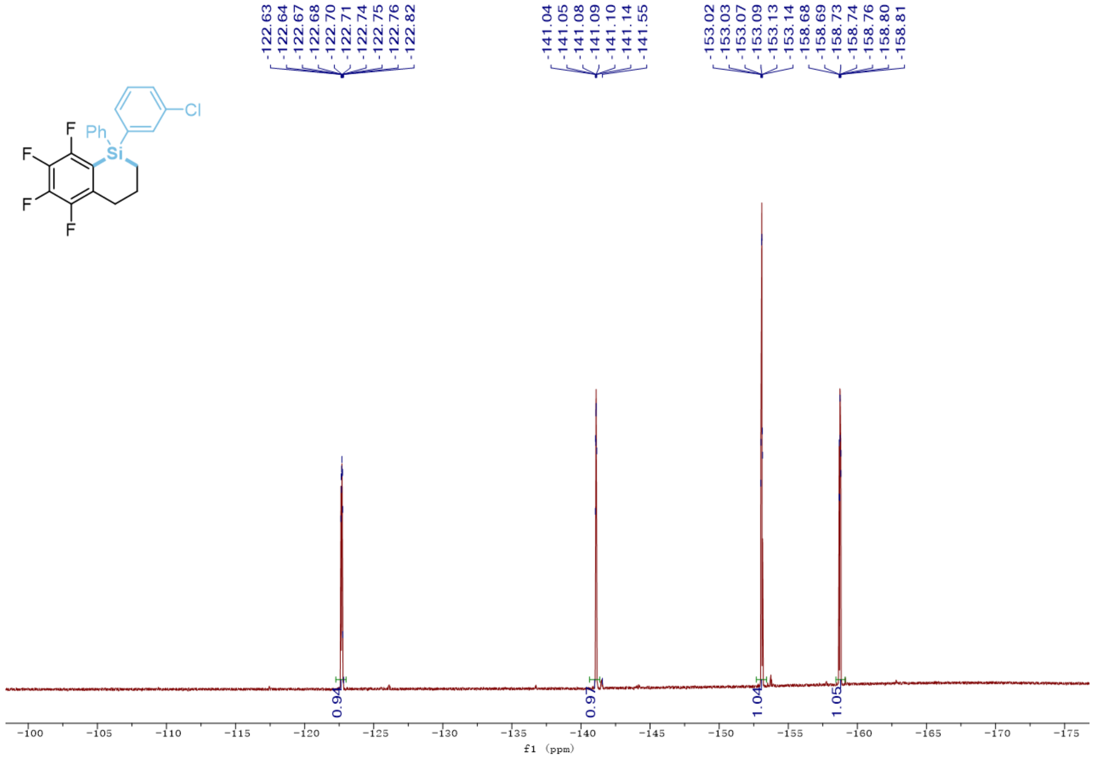
-122.63
-122.64
-122.67
-122.68
-122.70
-122.71
-122.74
-122.75
-122.76
-122.82
-141.04
-141.05
-141.08
-141.09
-141.10
-141.14
-141.55
-153.02
-153.03
-153.07
-153.09
-153.13
-153.14
-158.68
-158.69
-158.73
-158.74
-158.76
-158.80
-158.81
F
F
F
F
Ph
Si
Cl
0.94
0.97
1.04
1.05
f1 (ppm)

SUPPORTING INFORMATION

1-(4-chlorophenyl)-5,6,7,8-tetrafluoro-1-phenyl-1,2,3,4-tetrahydrobenzo[*b*]siline (**3g**)

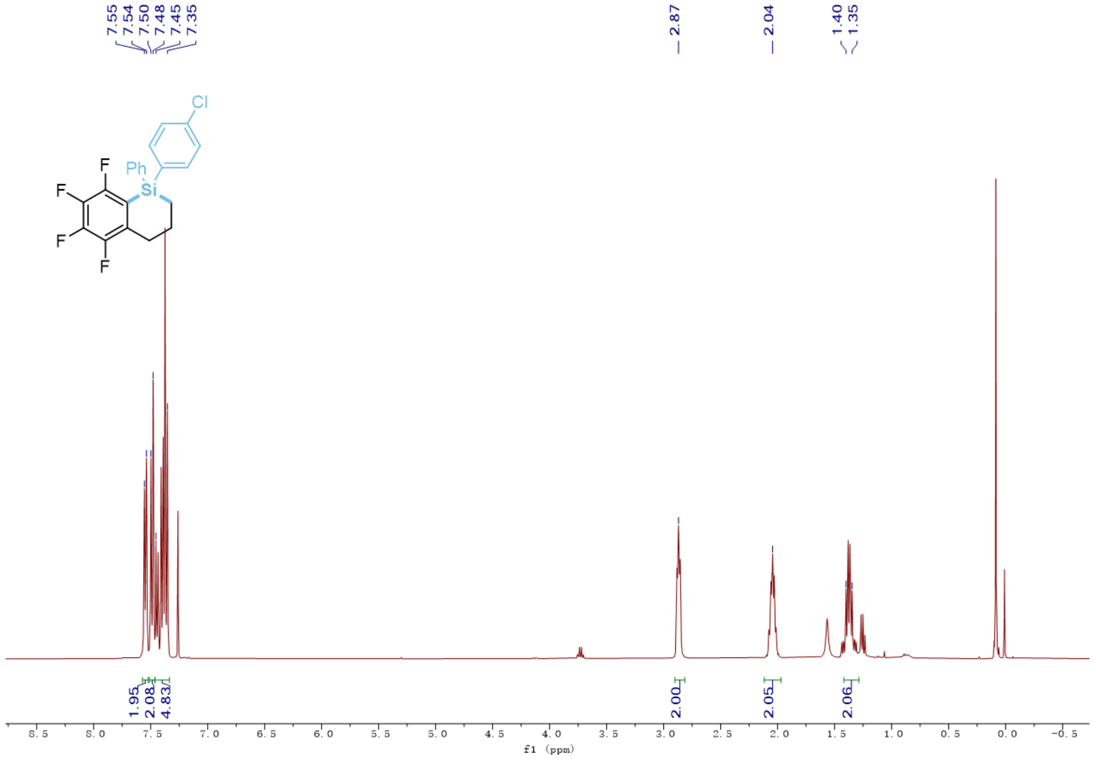

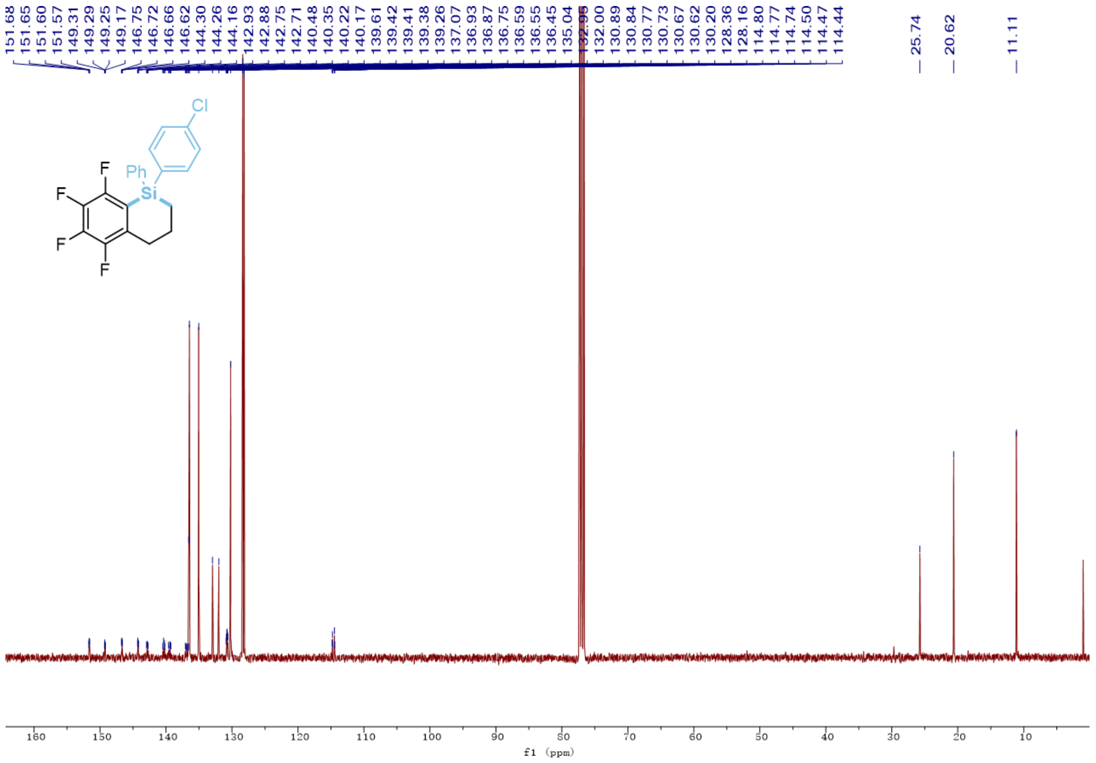

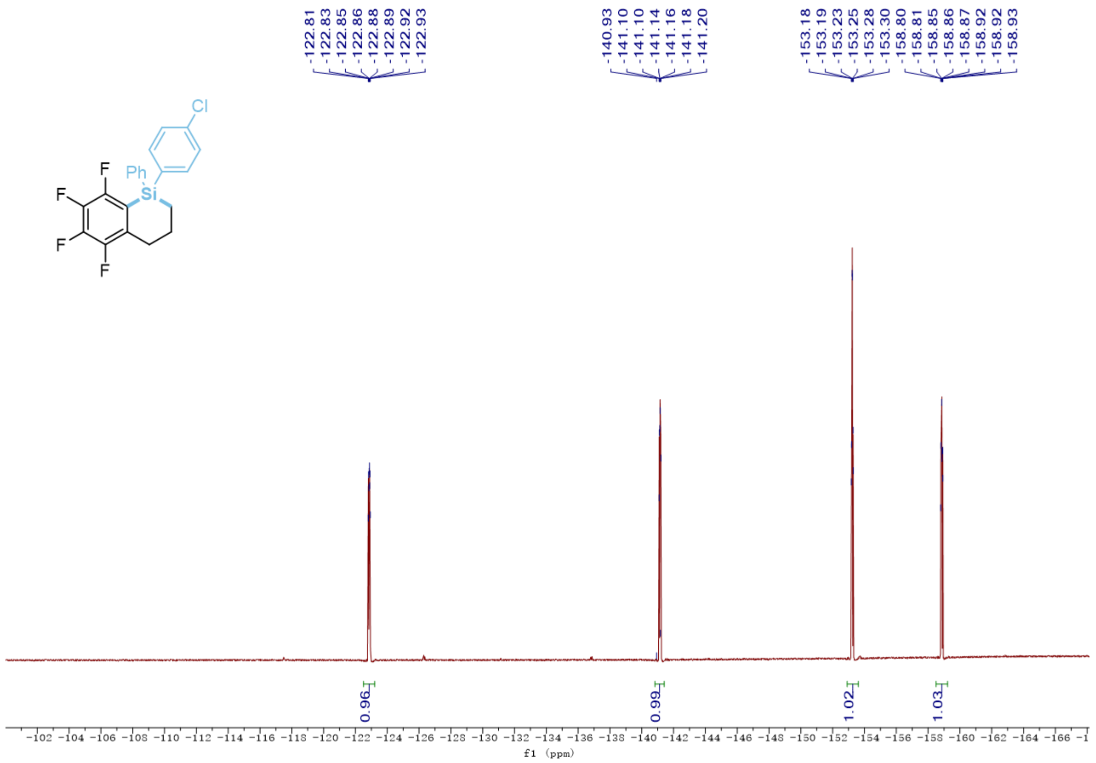

5,6,7,8-tetrafluoro-1-(4-fluorophenyl)-1-phenyl-1,2,3,4-tetrahydrobenzo[*b*]siline (**3h**)

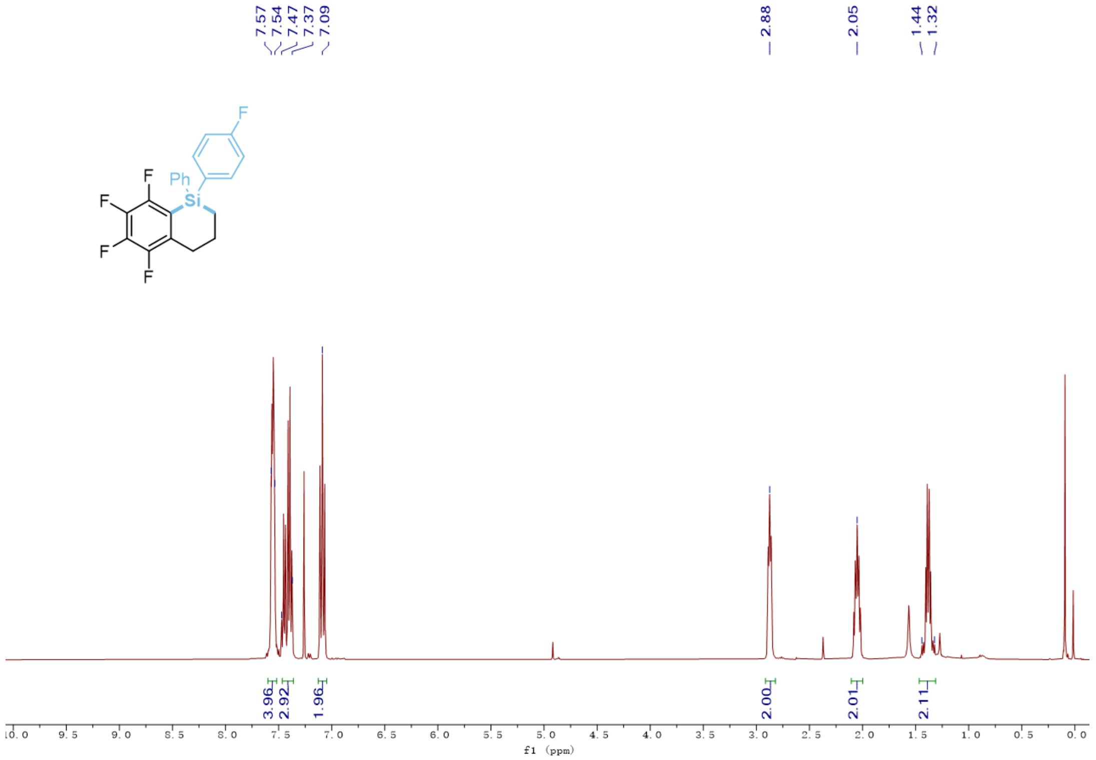

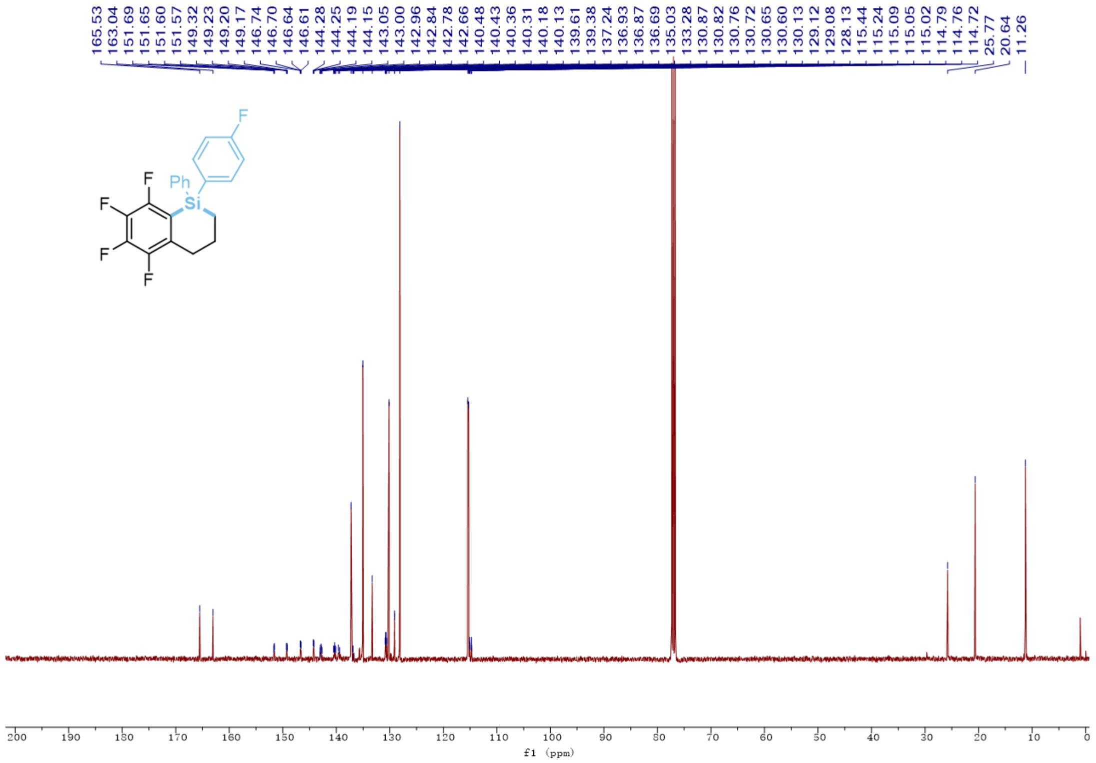
165.53
163.04
151.69
151.65
151.60
151.57
149.32
149.23
149.20
149.17
146.74
146.70
146.64
146.61
144.28
144.25
144.19
144.15
143.05
143.00
142.96
142.84
142.78
142.66
140.48
140.43
140.36
140.31
140.18
140.13
139.61
139.38
137.24
136.93
136.87
136.69
135.03
133.28
130.87
130.82
130.76
130.72
130.65
130.60
130.13
129.12
129.08
128.13
115.44
115.24
115.09
115.05
115.02
114.79
114.76
114.72
25.77
20.64
11.26
F
Ph
Si
F
F
F
F
f1 (ppm)

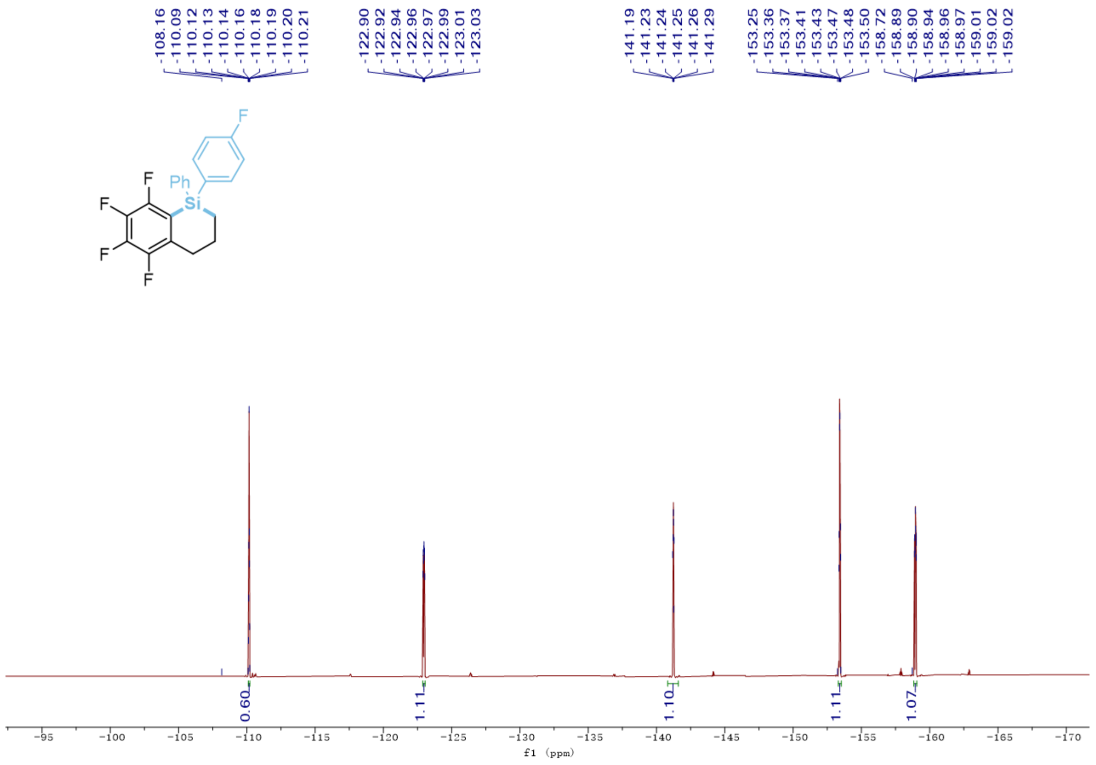
-108.16
-110.09
-110.12
-110.13
-110.14
-110.16
-110.18
-110.19
-110.20
-110.21
-122.90
-122.92
-122.94
-122.96
-122.97
-122.99
-123.01
-123.03
-141.19
-141.23
-141.24
-141.25
-141.26
-141.29
-153.25
-153.36
-153.37
-153.41
-153.43
-153.47
-153.48
-153.50
-158.72
-158.89
-158.90
-158.94
-158.96
-158.97
-159.01
-159.02
-159.02
F
Ph
Si
F
F
F
F
0.60
1.11
1.10
1.11
1.07
f1 (ppm)

SUPPORTING INFORMATION

1-(4-(tert-butyl)phenyl)-5,6,7,8-tetrafluoro-1-phenyl-1,2,3,4-tetrahydrobenzo[*b*]siline (**3i**)

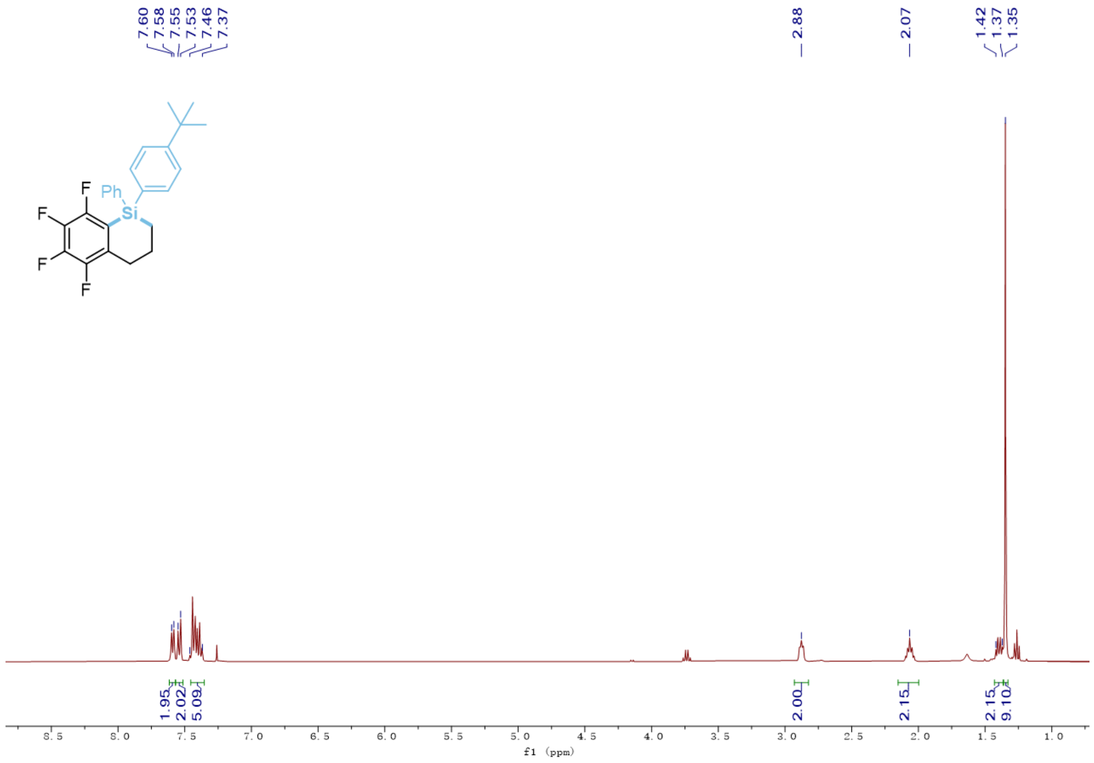

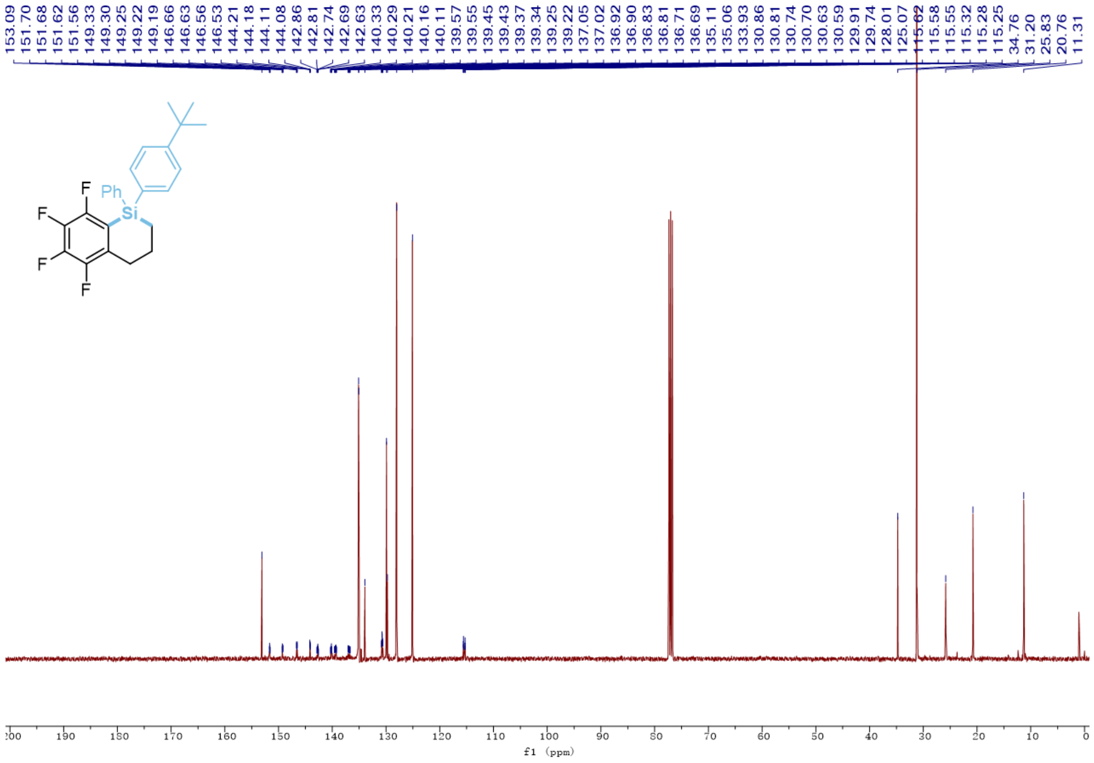

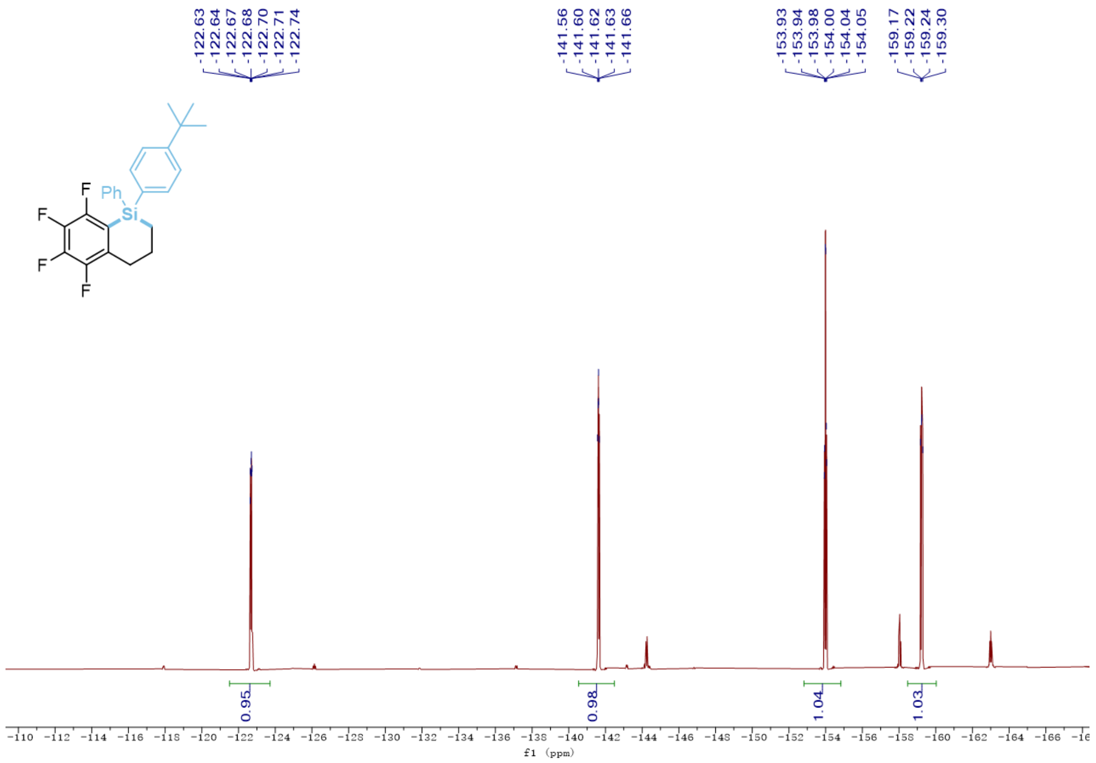

5,6,7,8-tetrafluoro-1-(4-methoxyphenyl)-1-phenyl-1,2,3,4-tetrahydrobenzo[*b*]siline (**3j**)

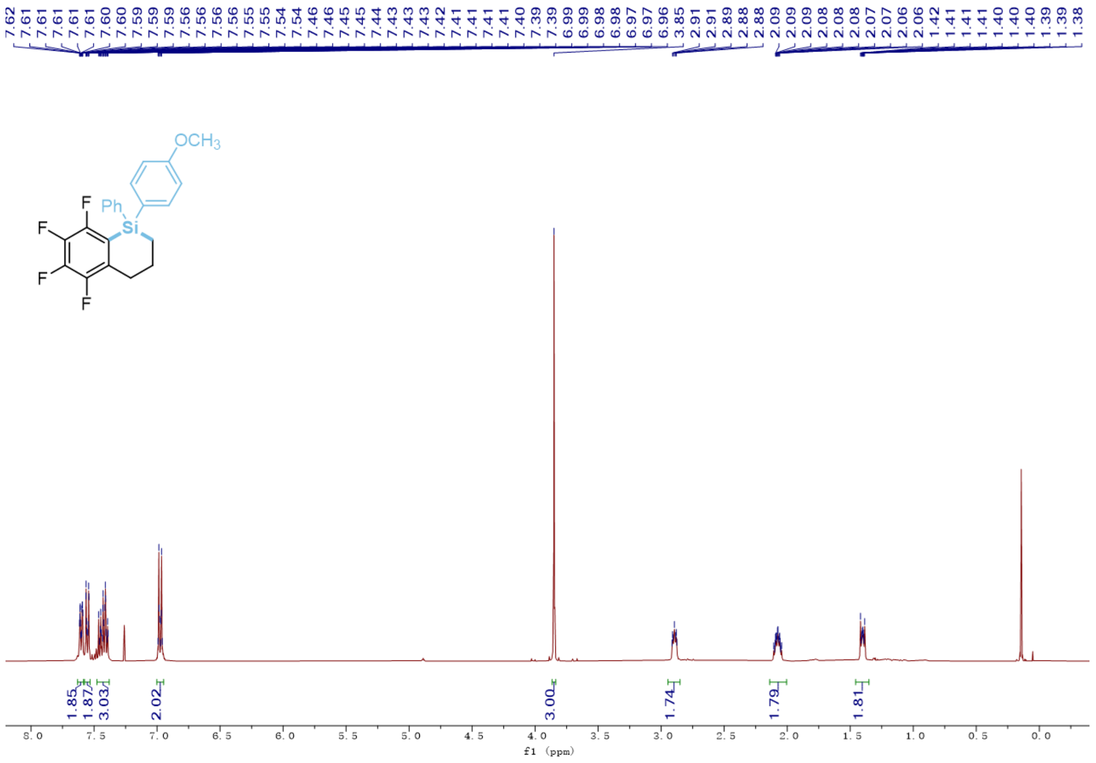

SUPPORTING INFORMATION

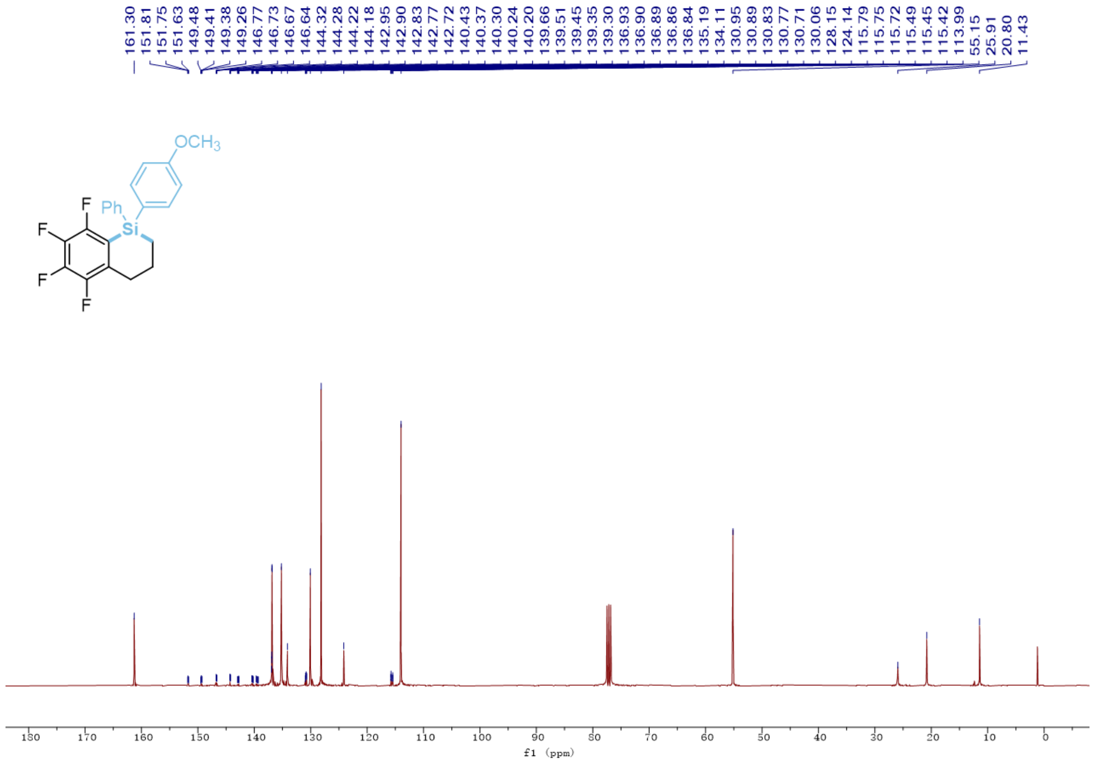

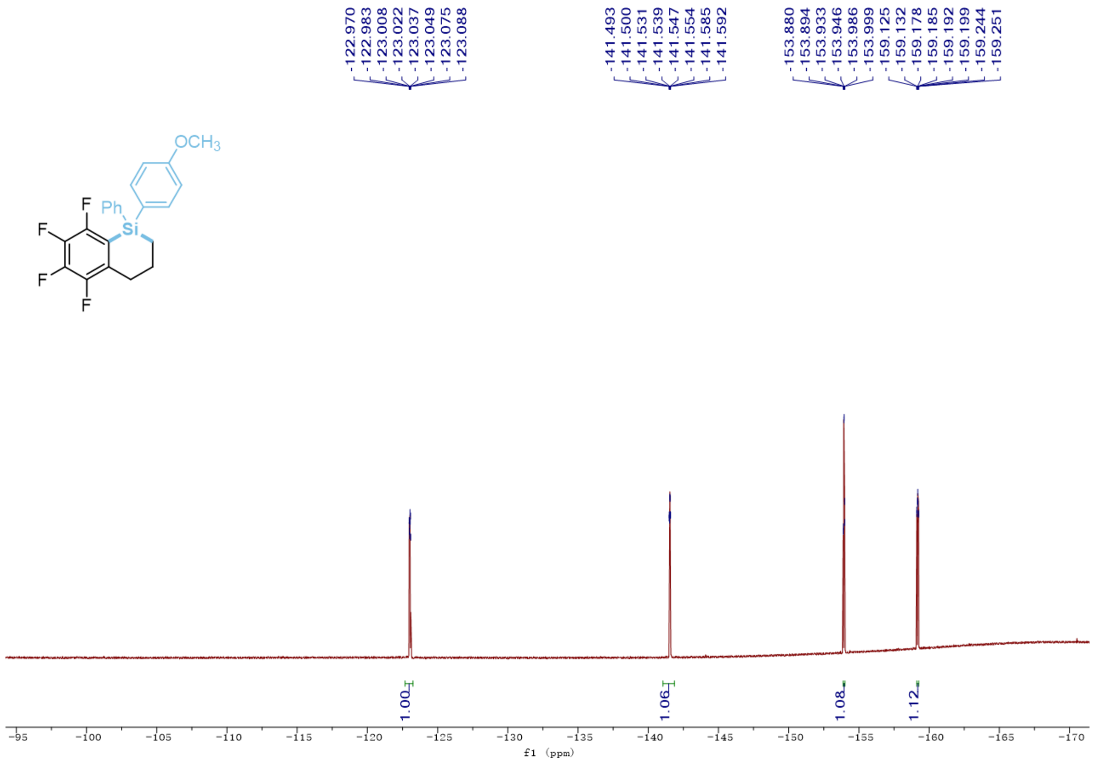

SUPPORTING INFORMATION

1-([1,1'-biphenyl]-4-yl)-5,6,7,8-tetrafluoro-1-phenyl-1,2,3,4-tetrahydrobenzo[*b*]siline (**3k**)

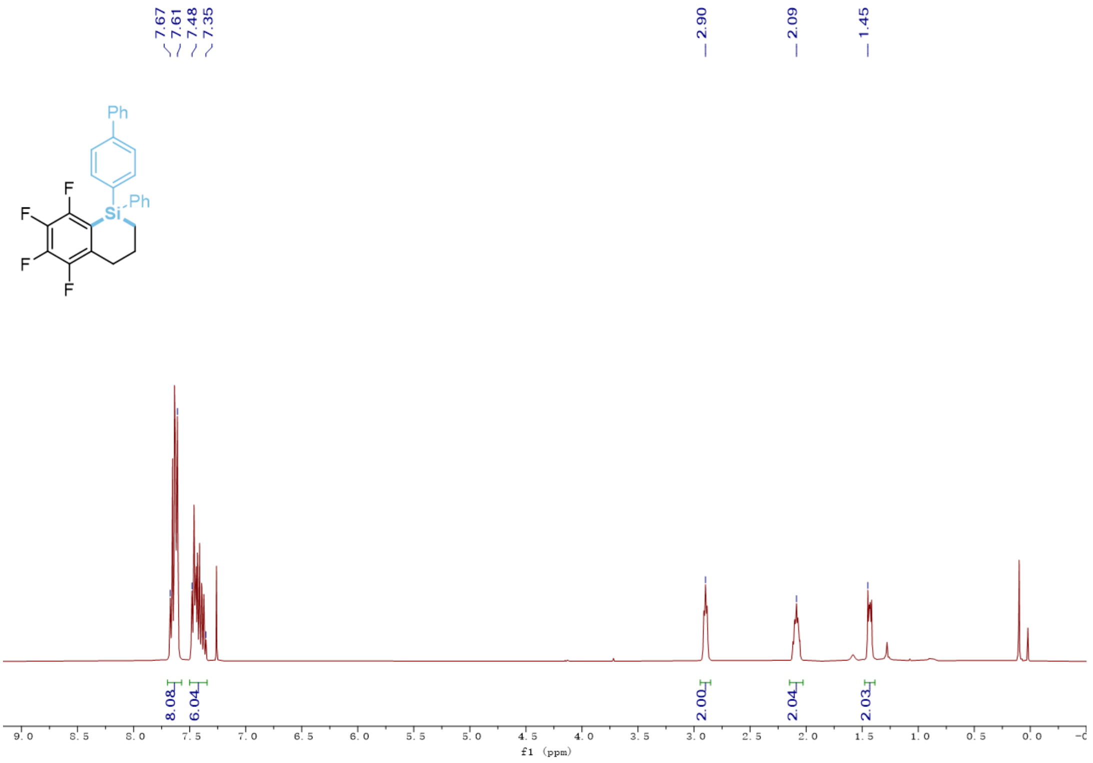

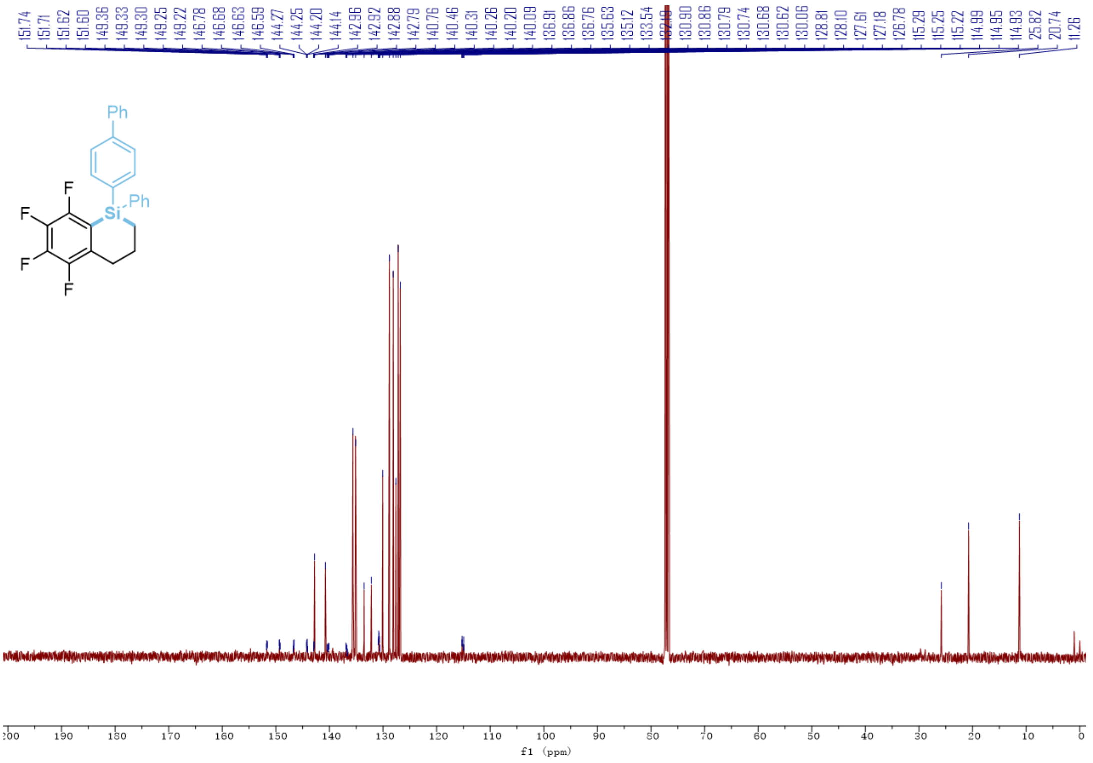

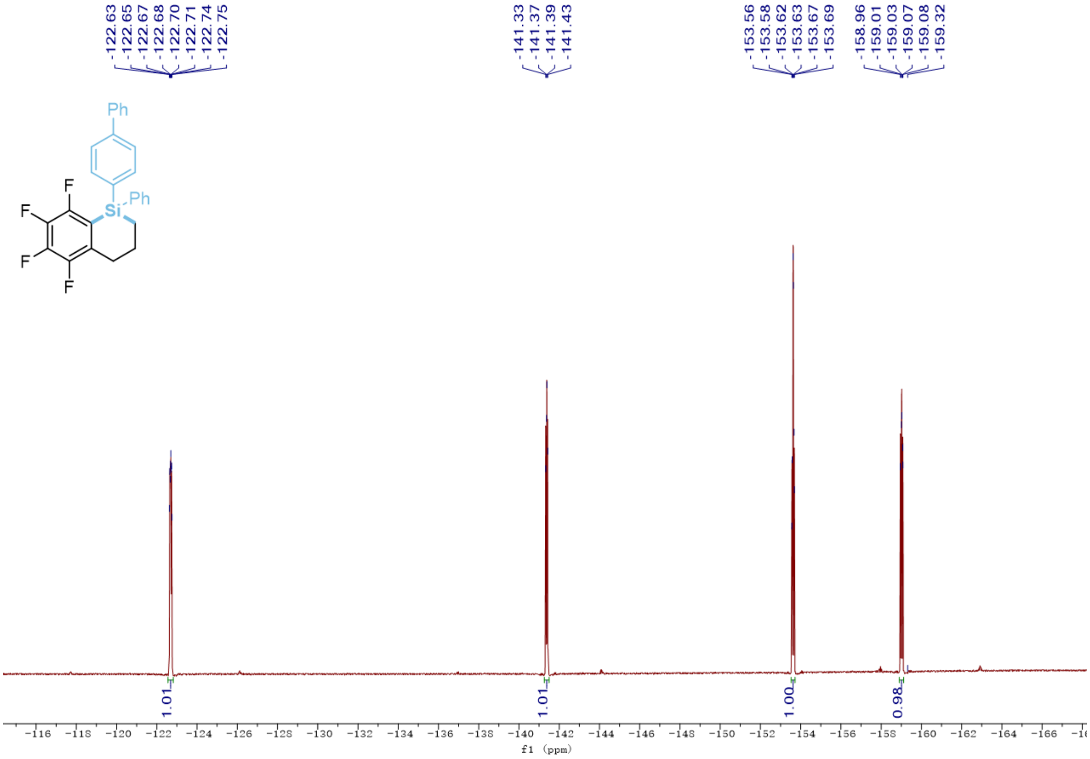

2-(5,6,7,8-tetrafluoro-1-phenyl-1,2,3,4-tetrahydrobenzo[*b*]silin-1-yl)thiophene (**3l**)

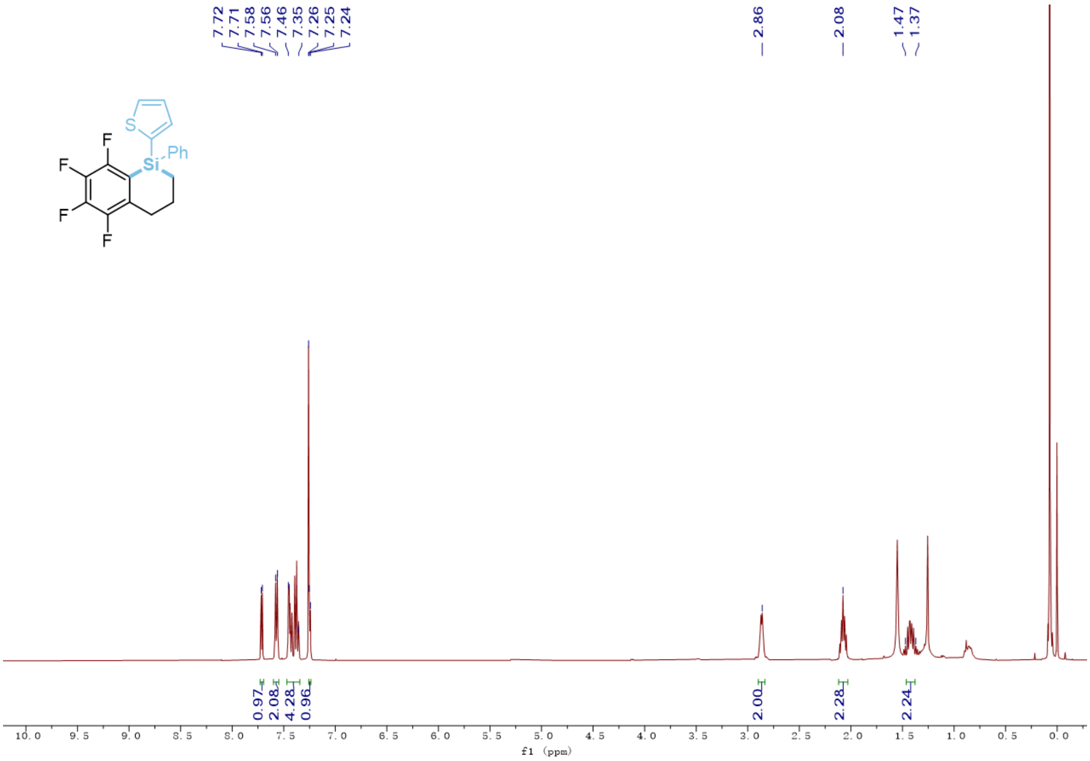

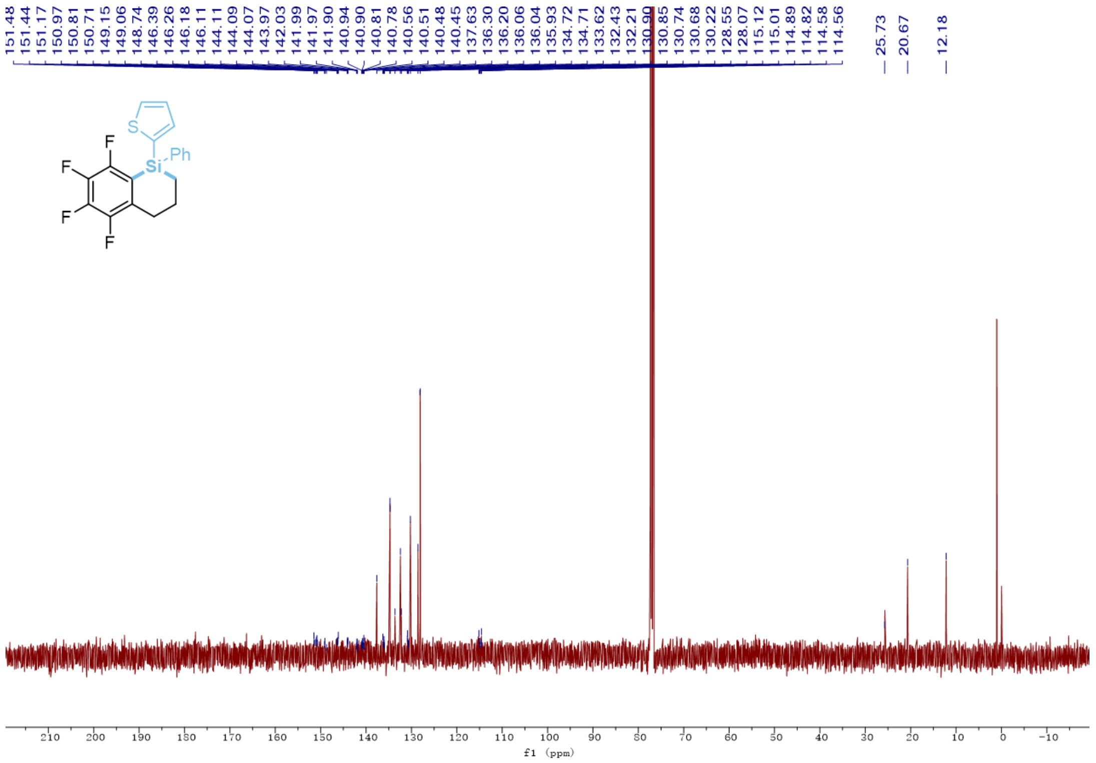
151.48
151.44
151.17
150.97
150.81
150.71
149.15
149.06
148.74
146.39
146.26
146.18
146.11
144.11
144.09
144.07
143.97
142.03
141.99
141.97
141.90
140.94
140.90
140.81
140.78
140.56
140.51
140.48
140.45
137.63
136.30
136.20
136.06
136.04
135.93
134.72
134.71
133.62
132.43
132.21
130.90
130.85
130.74
130.68
130.22
128.55
128.07
115.12
115.01
114.89
114.82
114.58
114.56
25.73
20.67
12.18
F
F
F
F
S
Si
Ph
f1 (ppm)

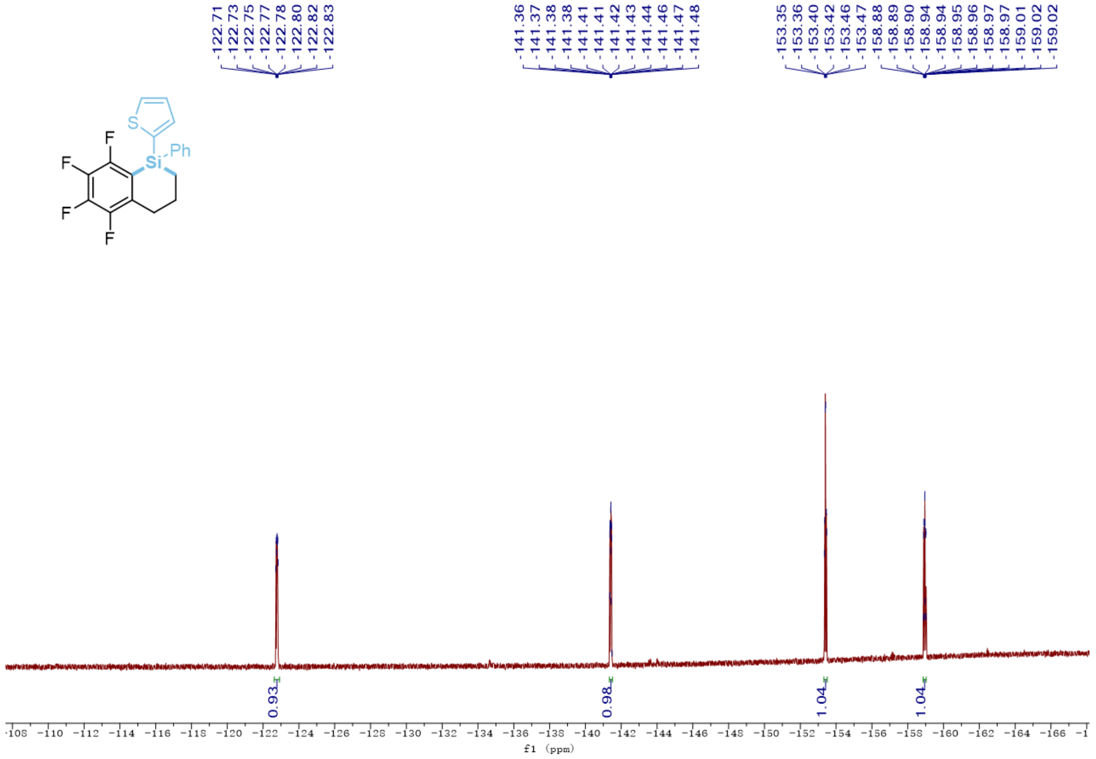
-122.71
-122.73
-122.75
-122.77
-122.78
-122.80
-122.82
-122.83
-141.36
-141.37
-141.38
-141.41
-141.41
-141.42
-141.43
-141.44
-141.46
-141.47
-141.48
-153.35
-153.36
-153.40
-153.42
-153.46
-153.47
-158.88
-158.89
-158.90
-158.94
-158.94
-158.95
-158.96
-158.97
-158.97
-159.01
-159.02
-159.02
F
F
F
F
S
Si
Ph
0.93
0.98
1.04
1.04
f1 (ppm)

SUPPORTING INFORMATION

1-ethyl-5,6,7,8-tetrafluoro-1-phenyl-1,2,3,4-tetrahydrobenzo[*b*]siline (**3m**)

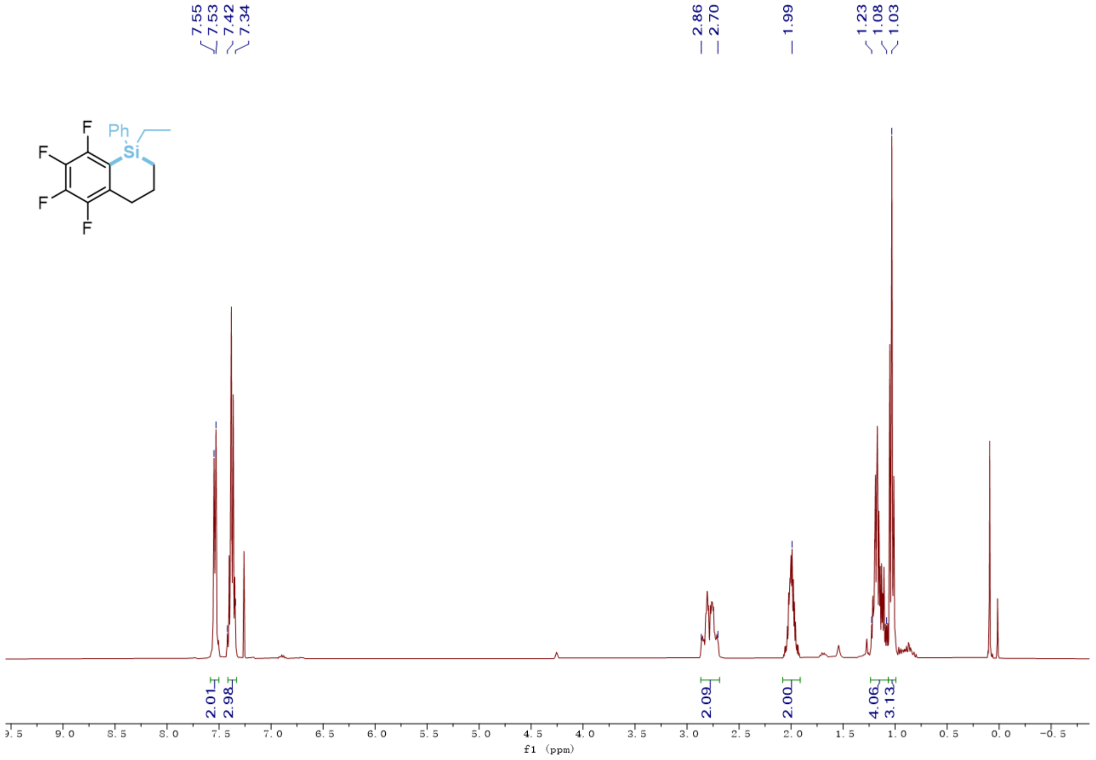

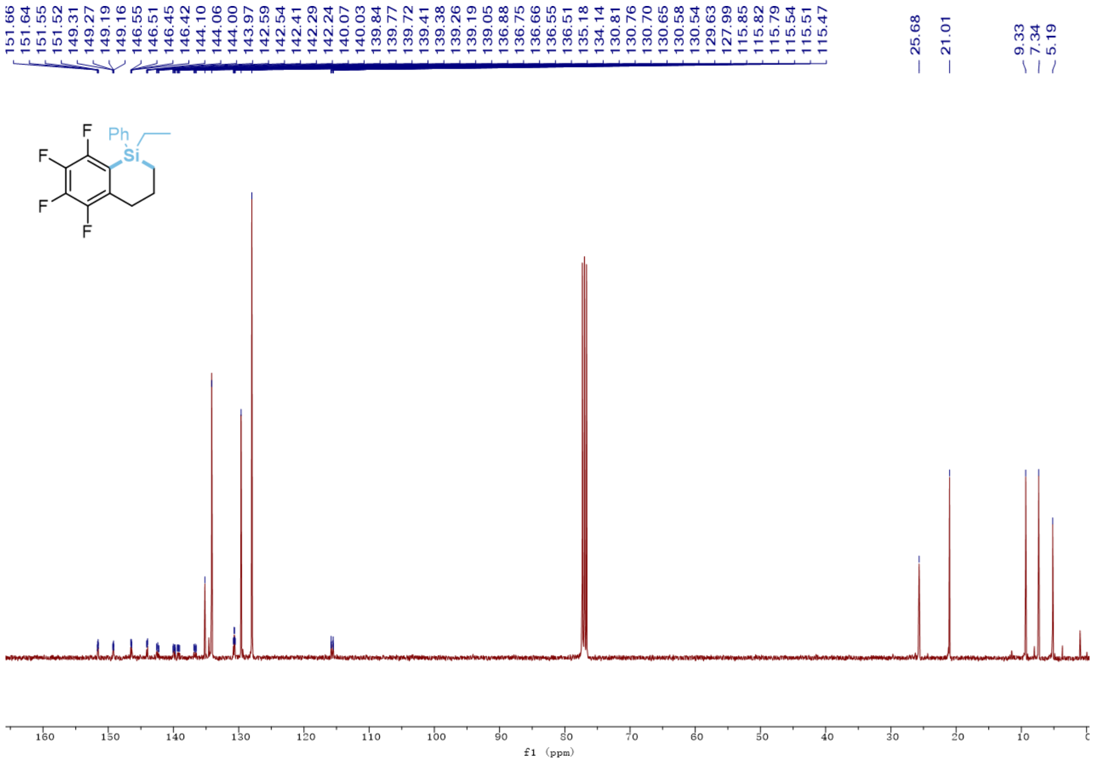

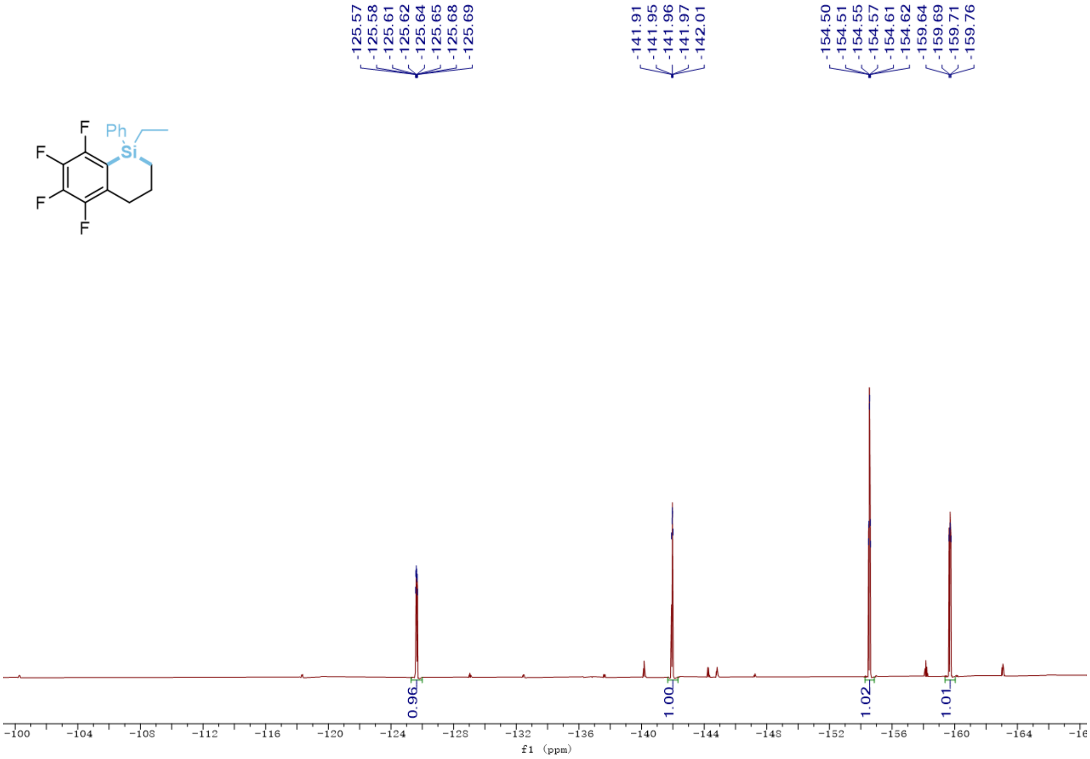

5,6,7,8-tetrafluoro-1-hexyl-1-phenyl-1,2,3,4-tetrahydrobenzo[*b*]siline (**3n**)

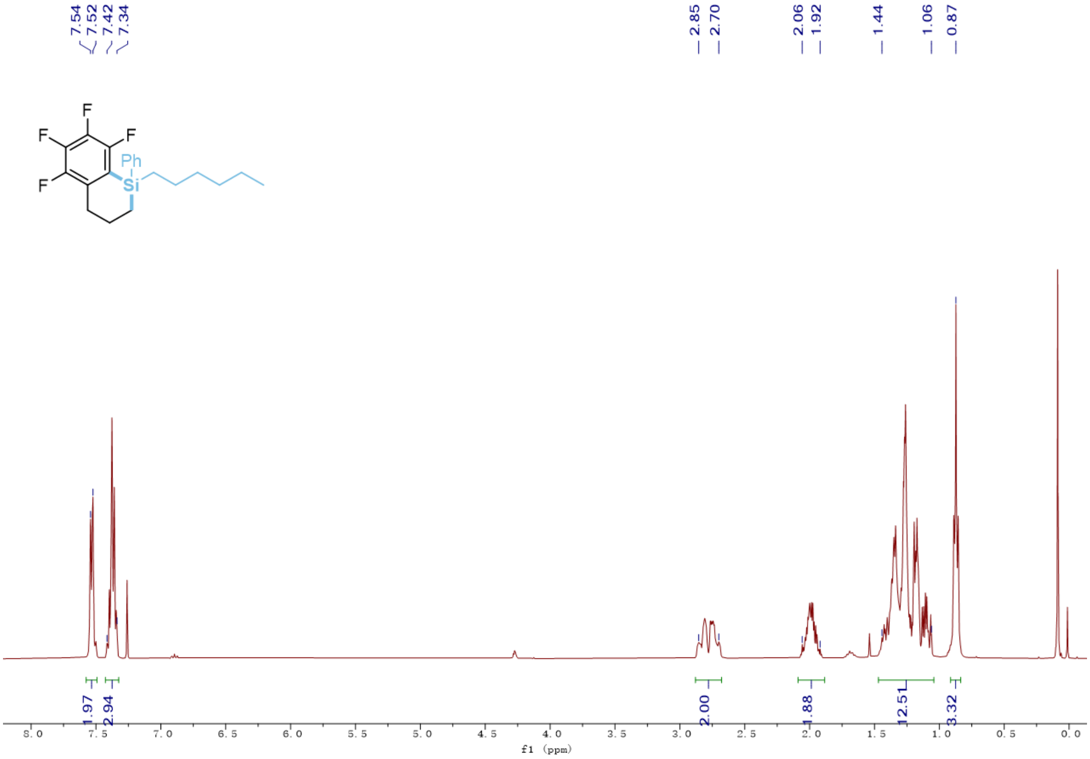

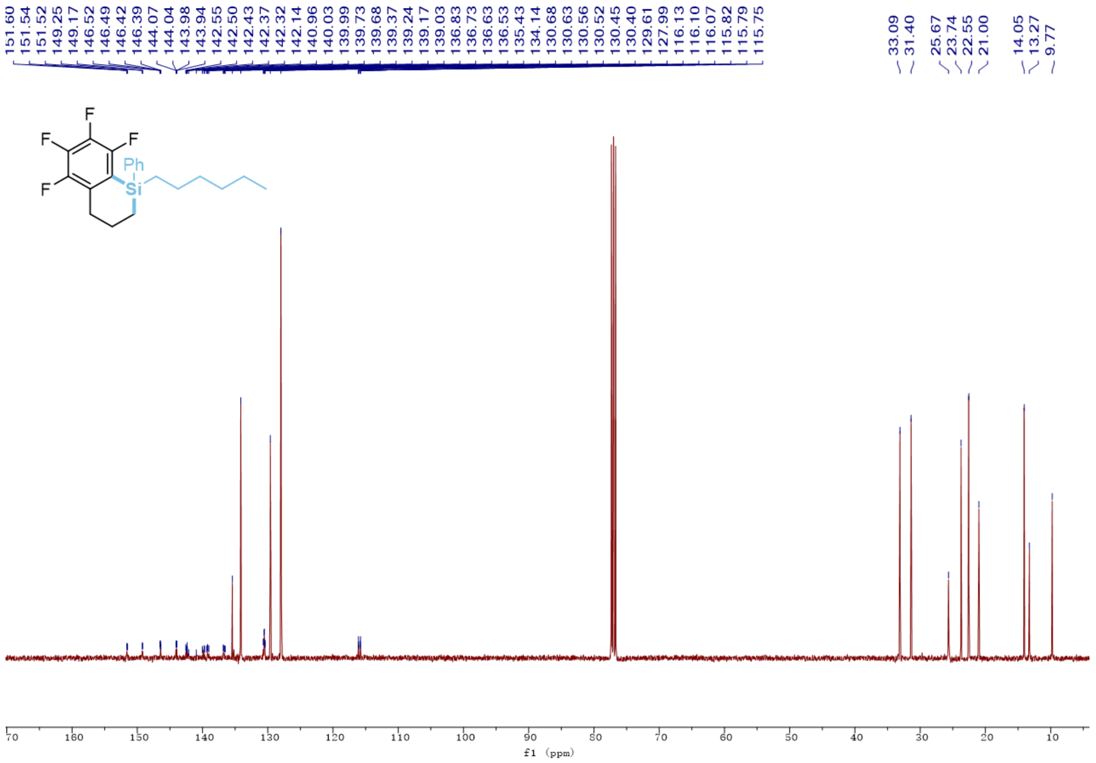

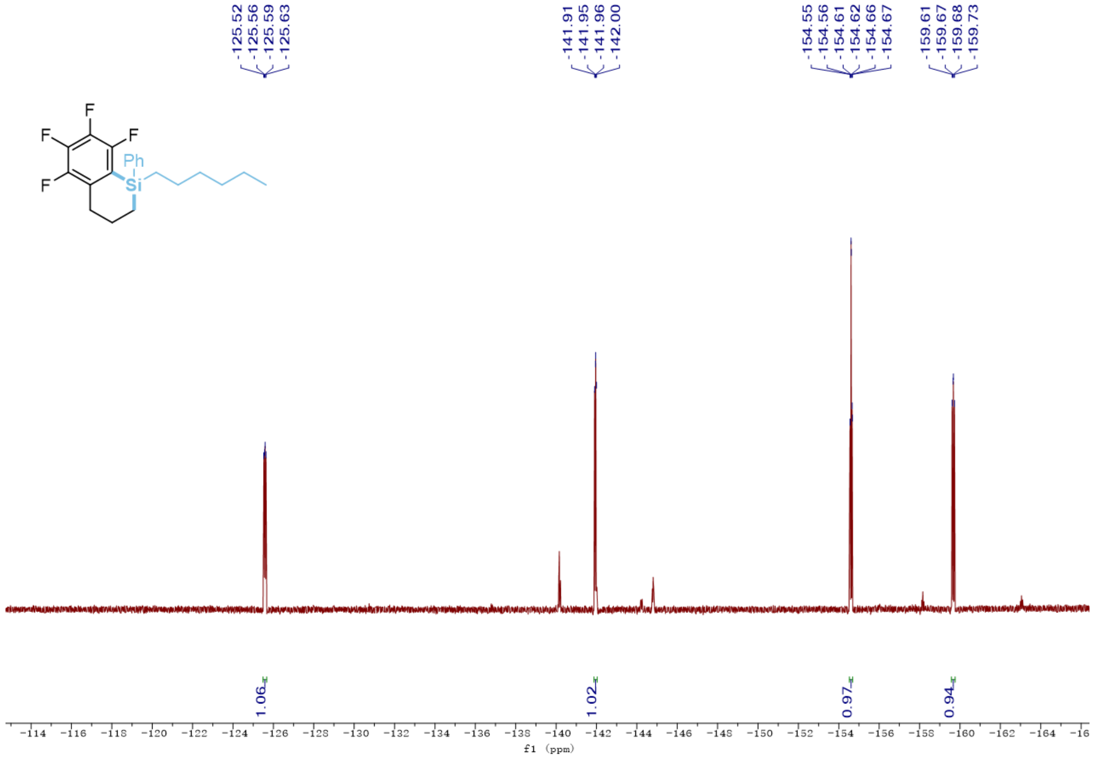

SUPPORTING INFORMATION

5,6,7,8-tetrafluoro-1-isobutyl-1-phenyl-1,2,3,4-tetrahydrobenzo[*b*]siline (**3o**)

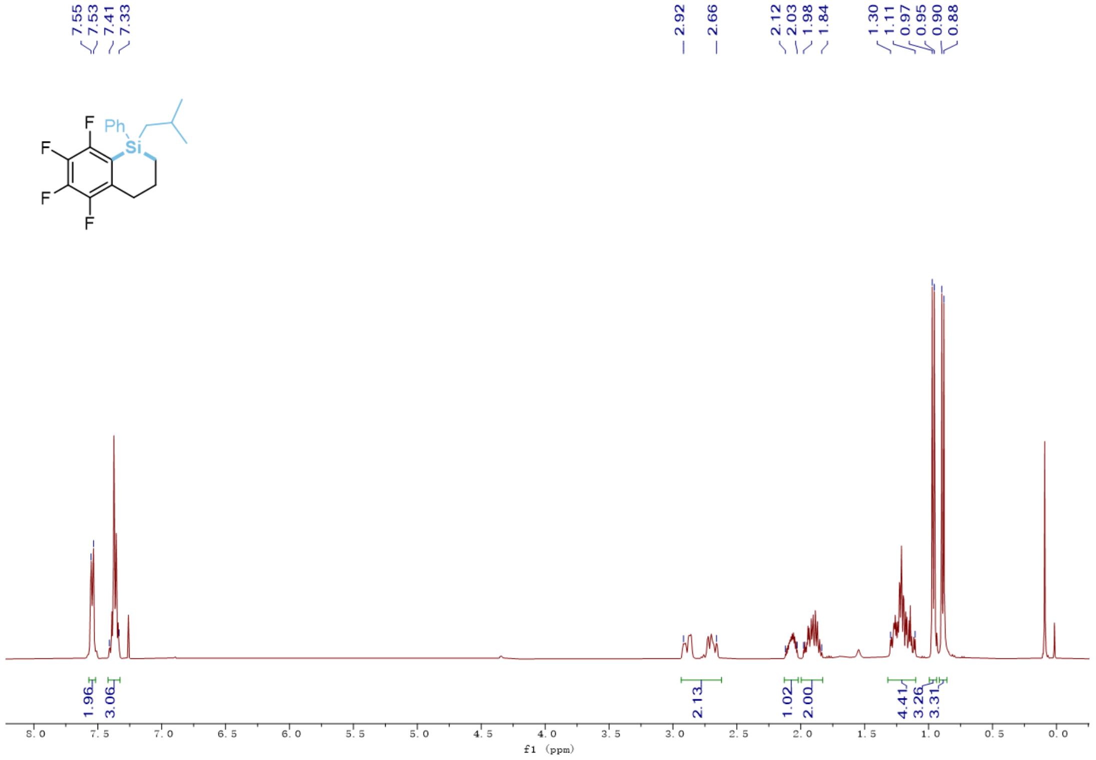

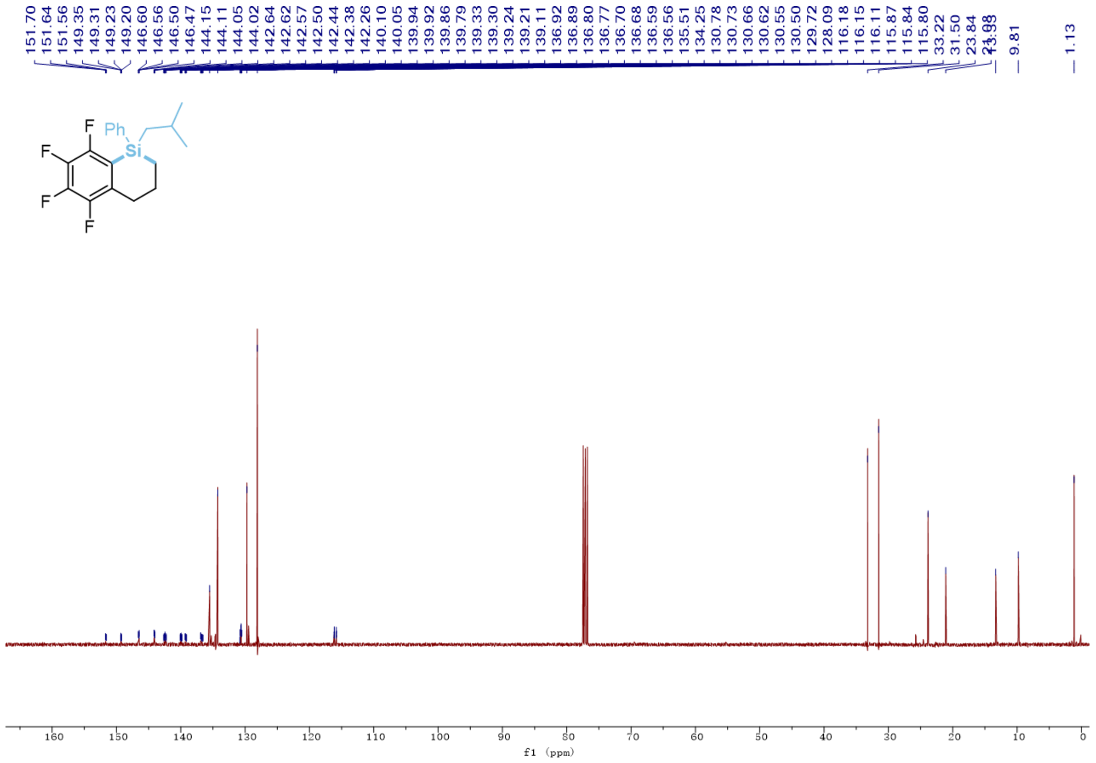

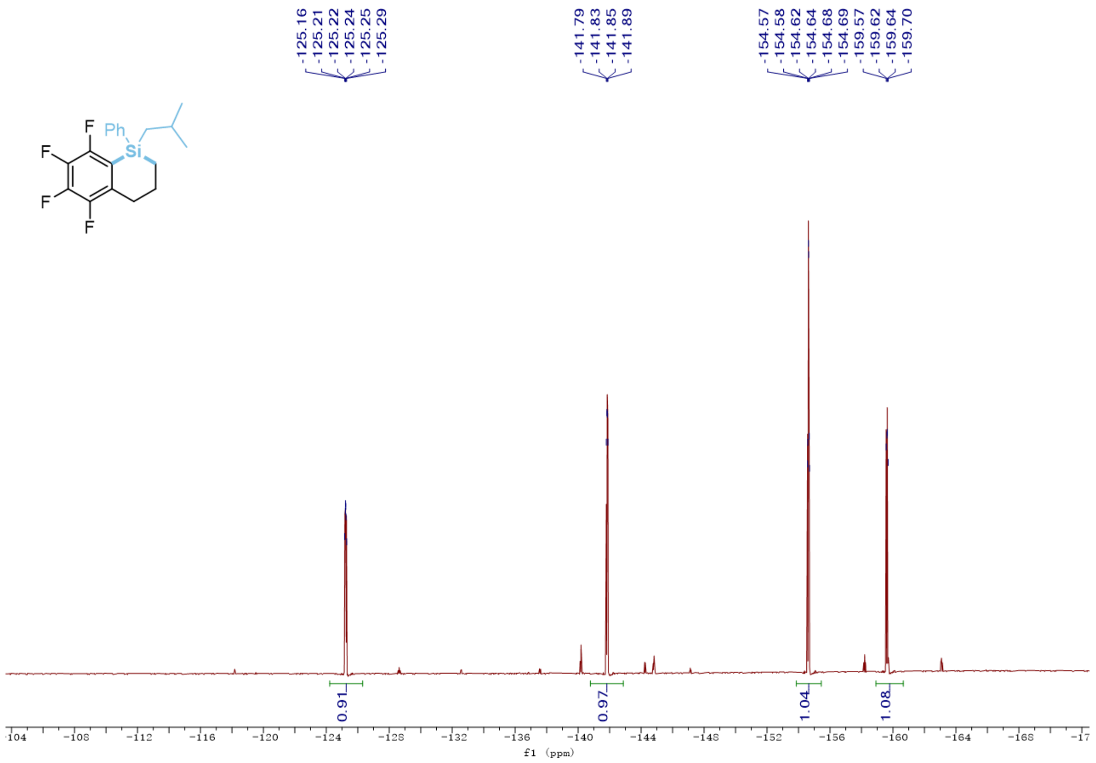

1-cyclopentyl-5,6,7,8-tetrafluoro-1-phenyl-1,2,3,4-tetrahydrobenzo[*b*]siline (**3p**)

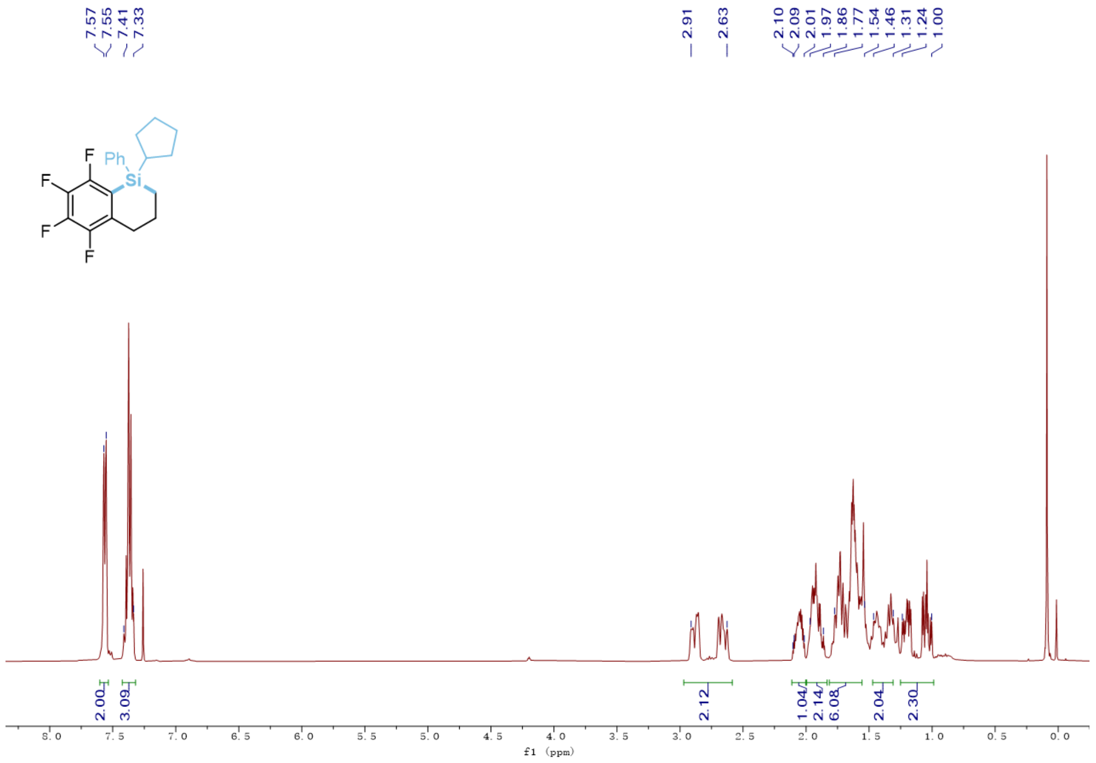

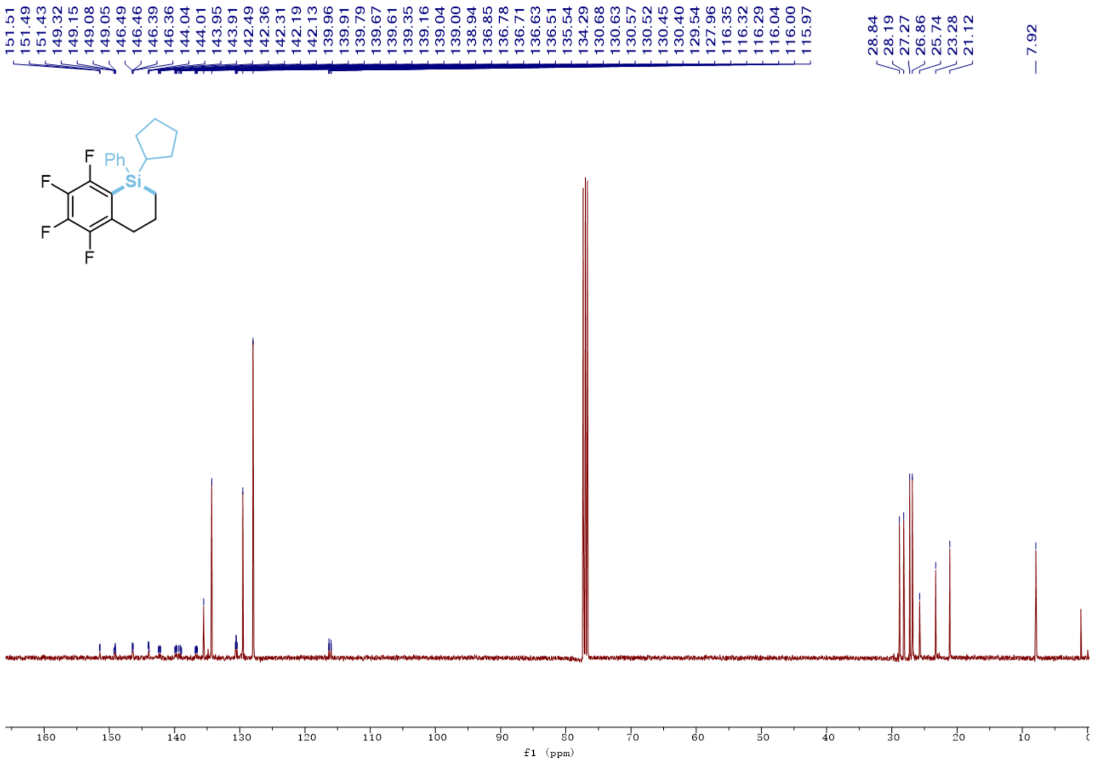

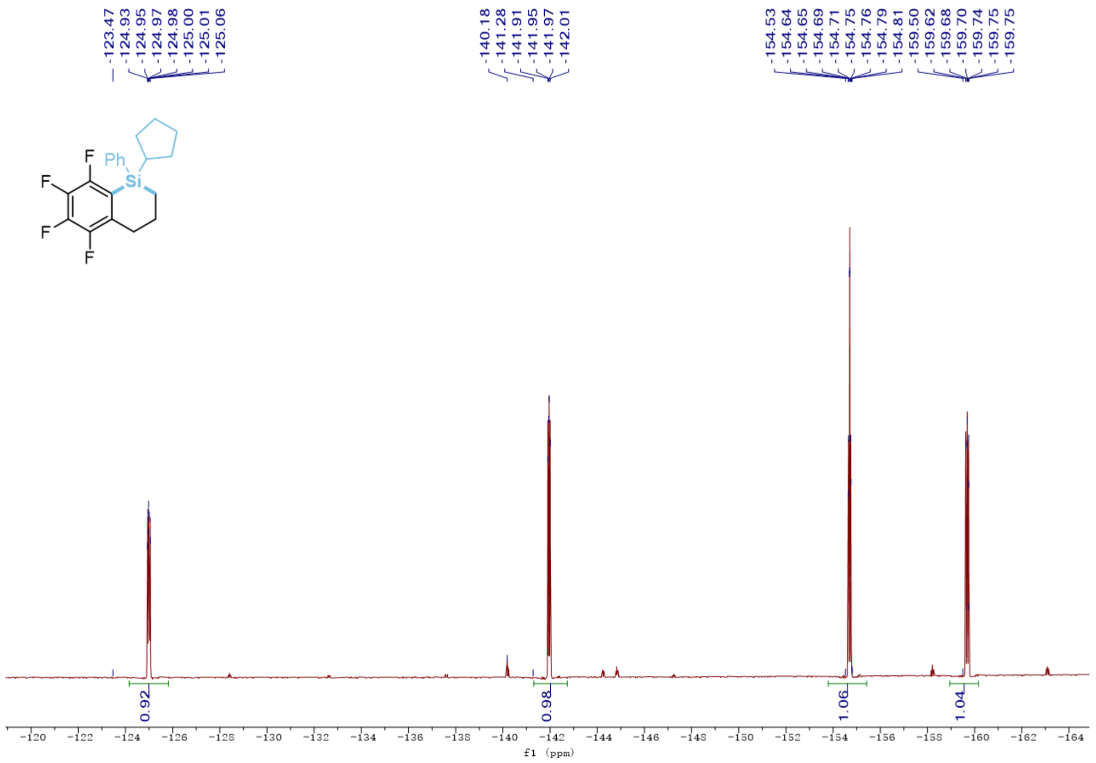

SUPPORTING INFORMATION

1-cyclohexyl-5,6,7,8-tetrafluoro-1-phenyl-1,2,3,4-tetrahydrobenzo[*b*]siline (**3q**)

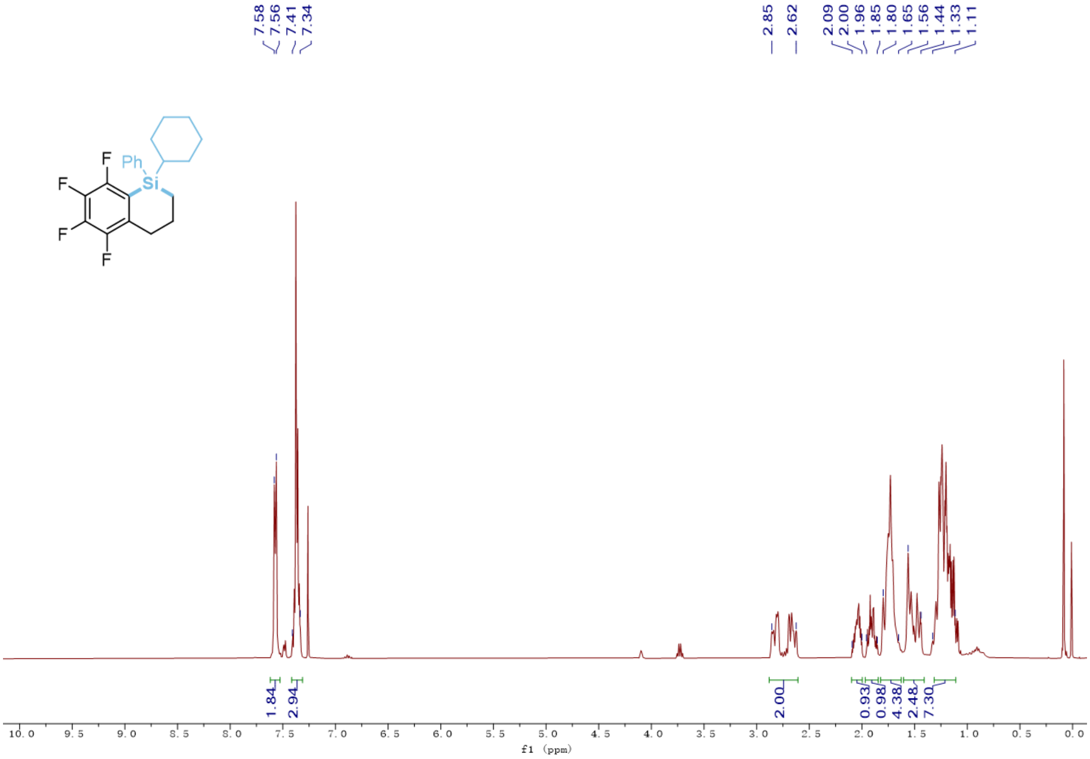

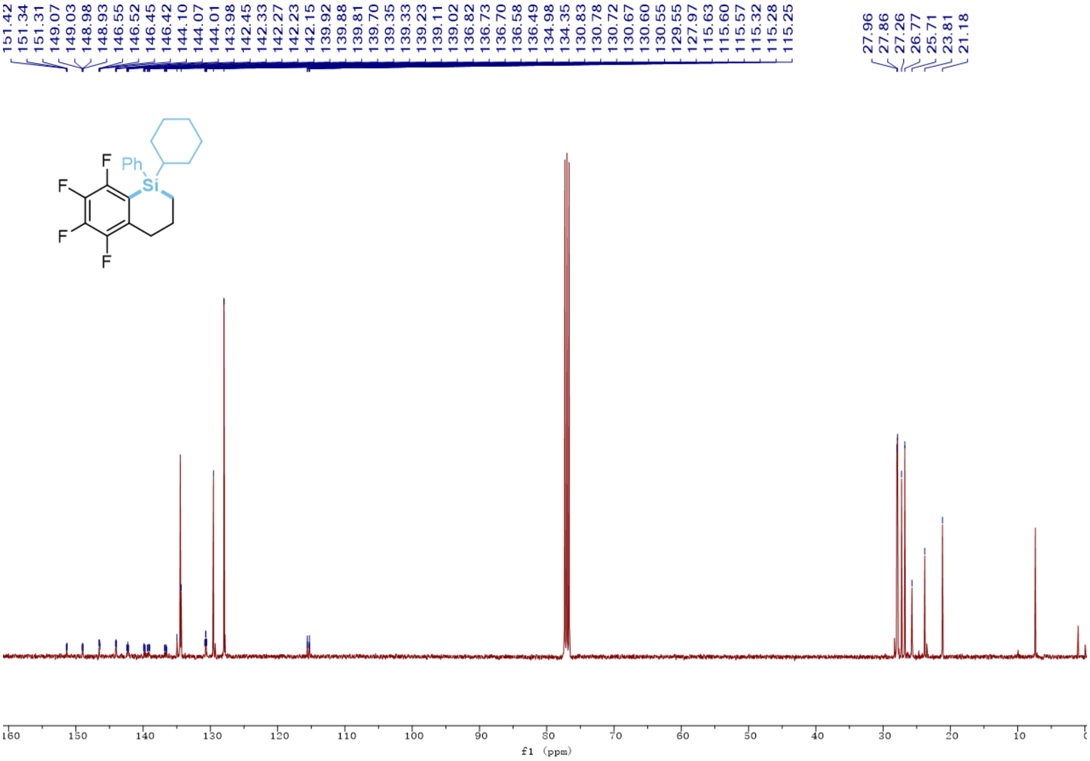

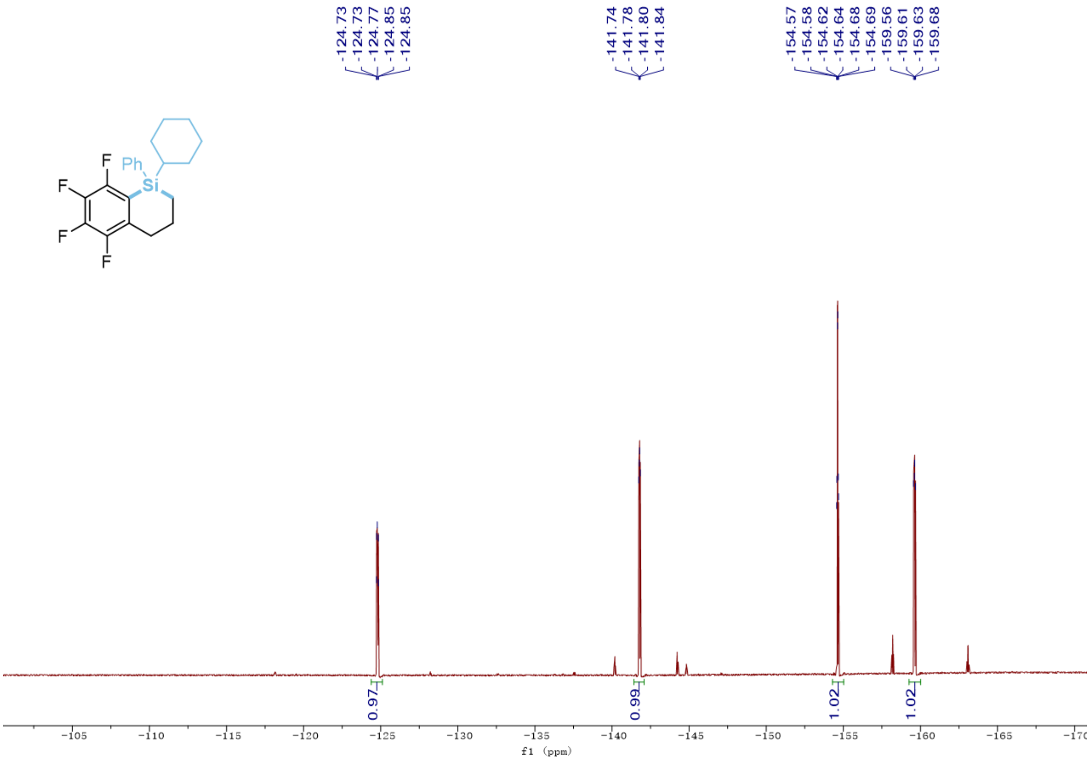

1,1-diethyl-5,6,7,8-tetrafluoro-1,2,3,4-tetrahydrobenzo[*b*]siline (**3r**)

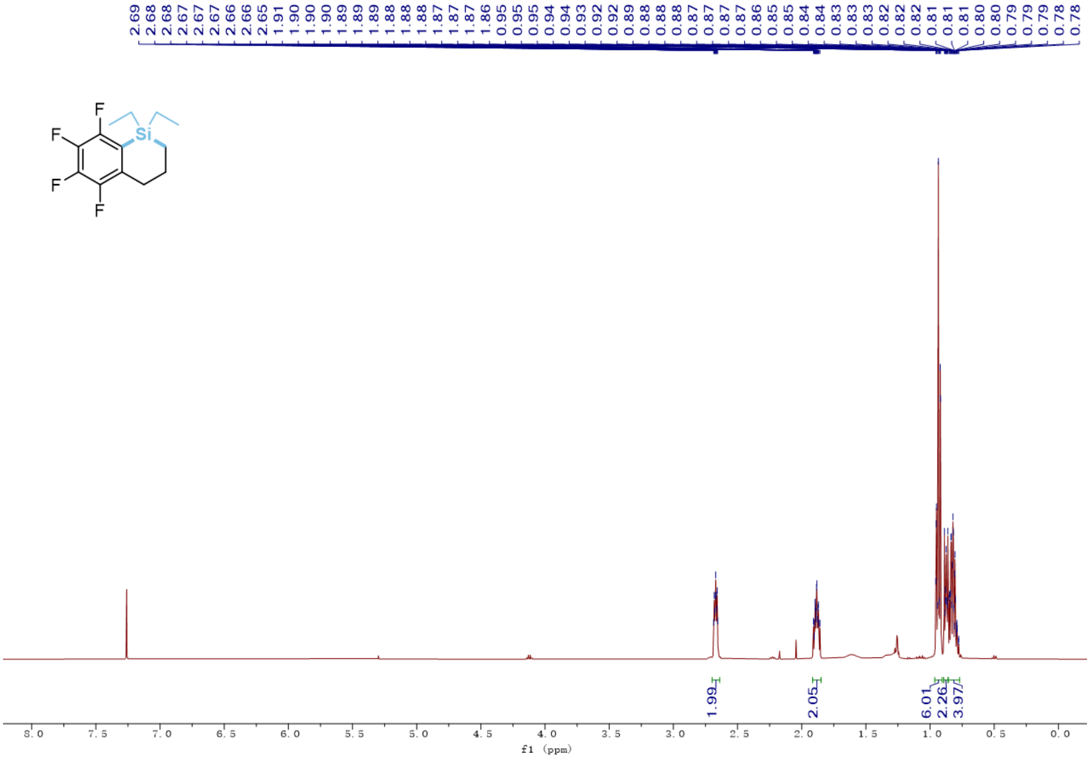

SUPPORTING INFORMATION

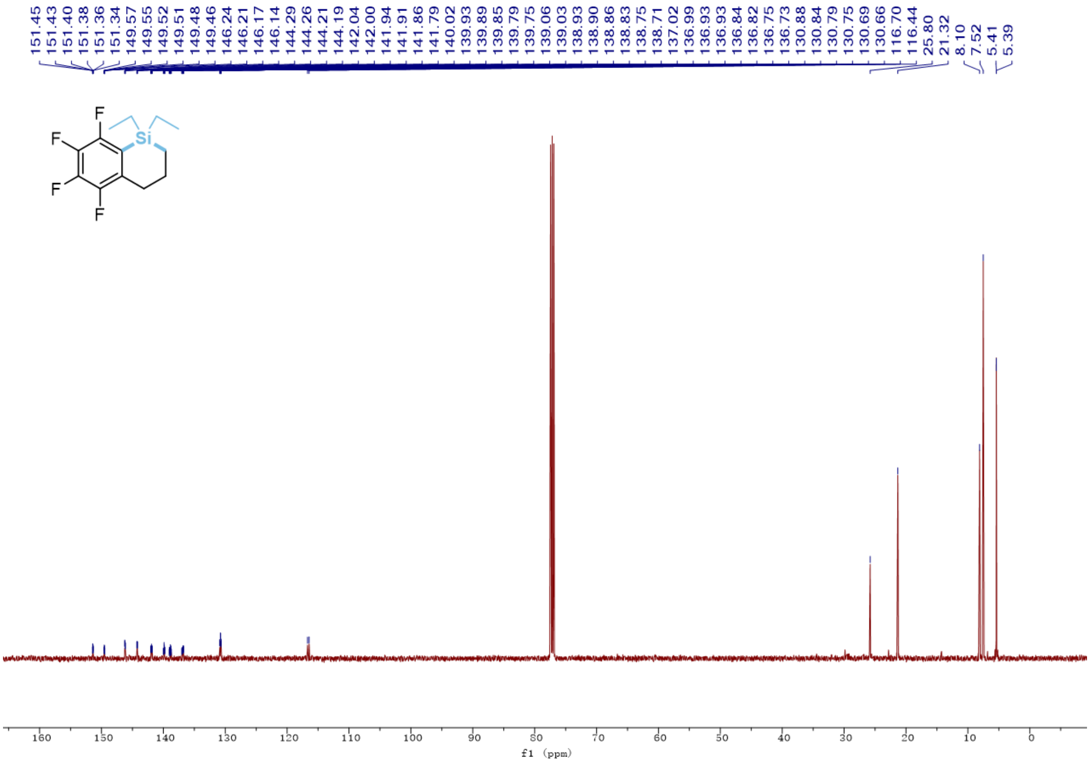

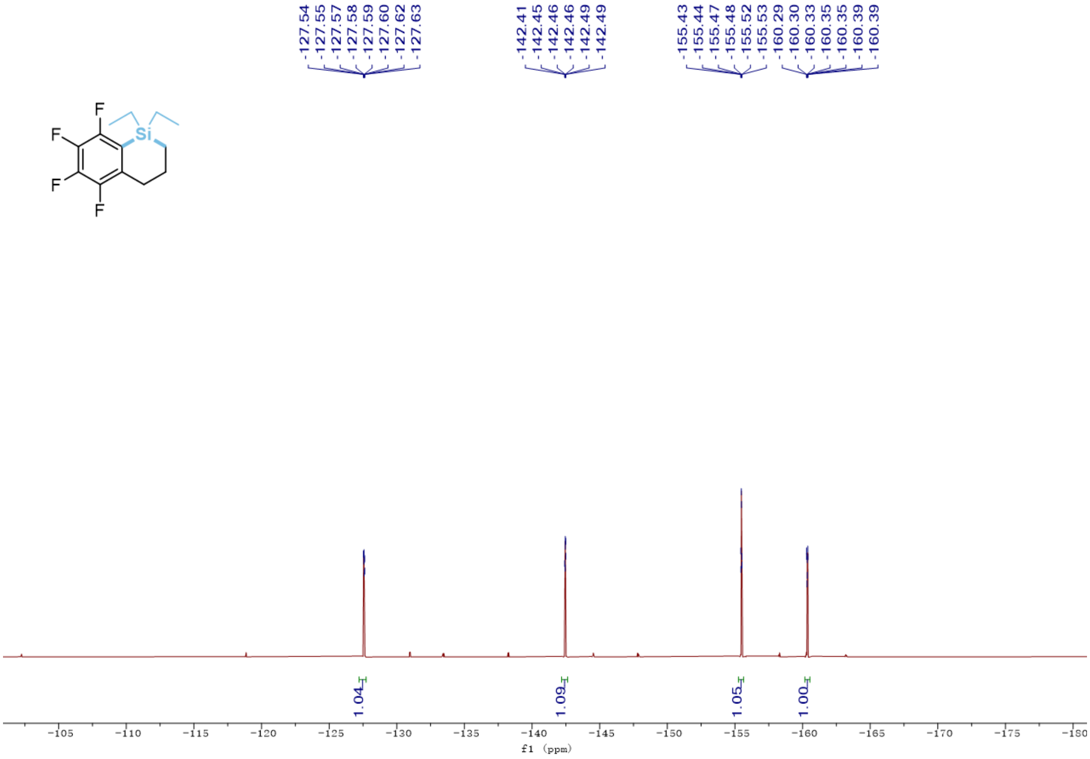

SUPPORTING INFORMATION

5,6,7,8-tetrafluoro-1,1-diphenyl-1,2,3,4-tetrahydrobenzo[*b*]germine (**3s**)

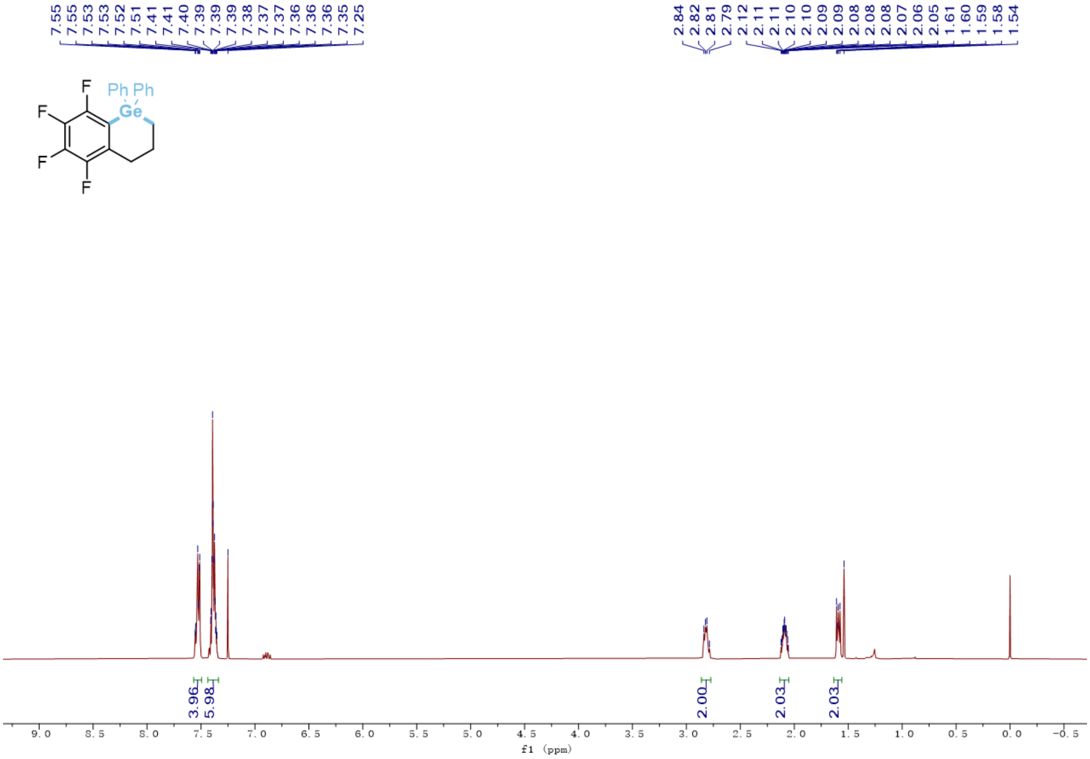

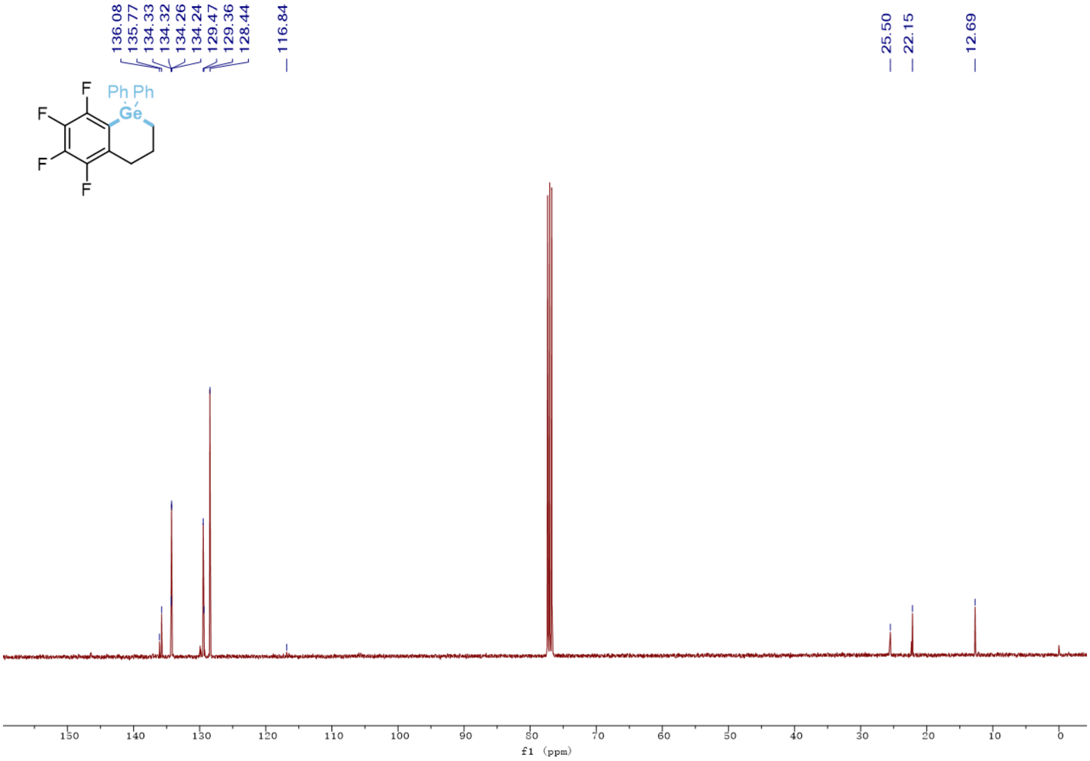

SUPPORTING INFORMATION

5,6,8-trifluoro-1,1-diphenyl-1,2,3,4-tetrahydrobenzo[*b*]siline (**4b**)

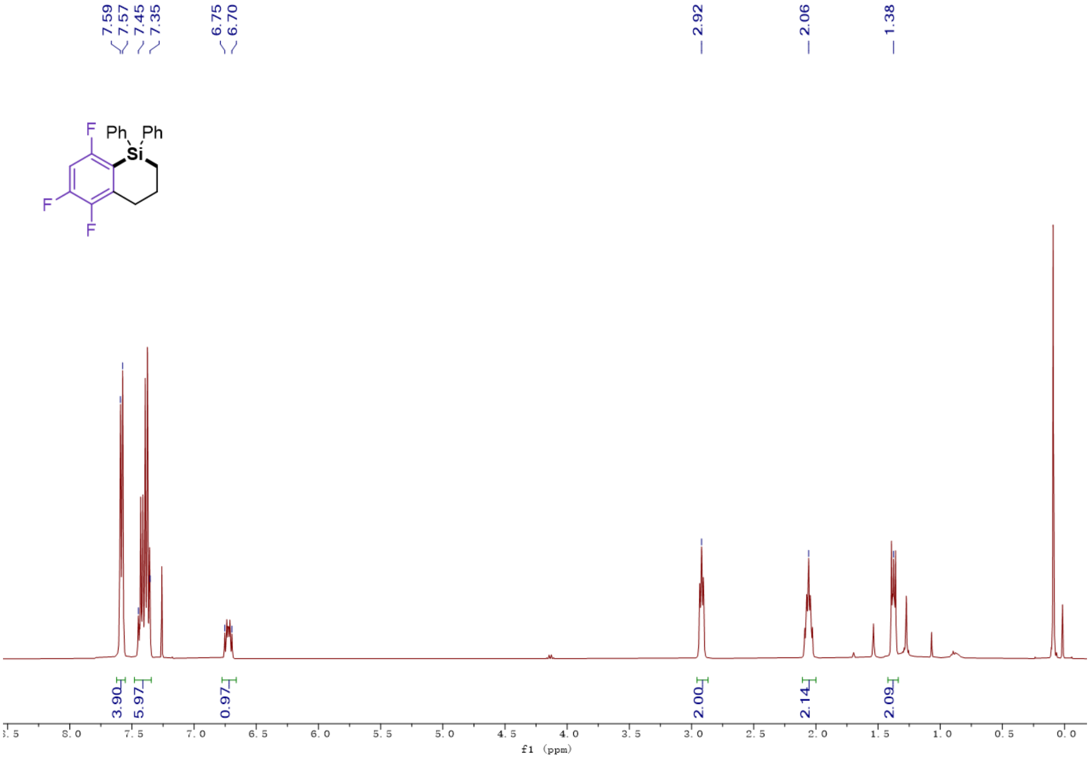

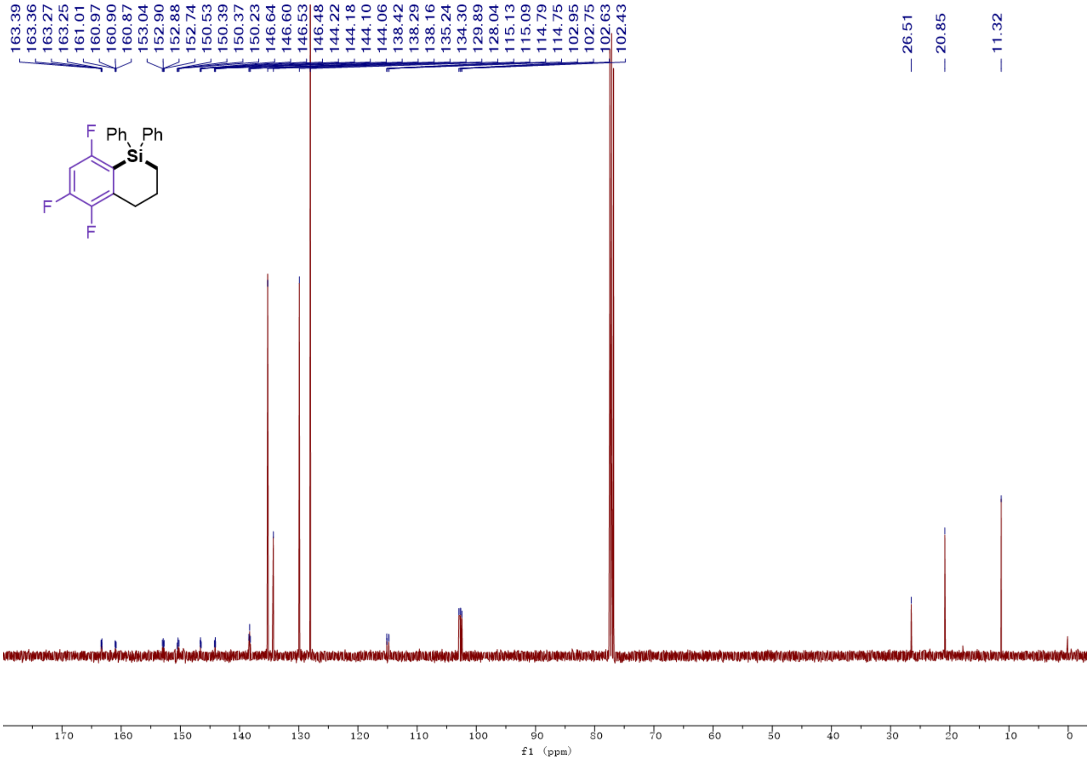

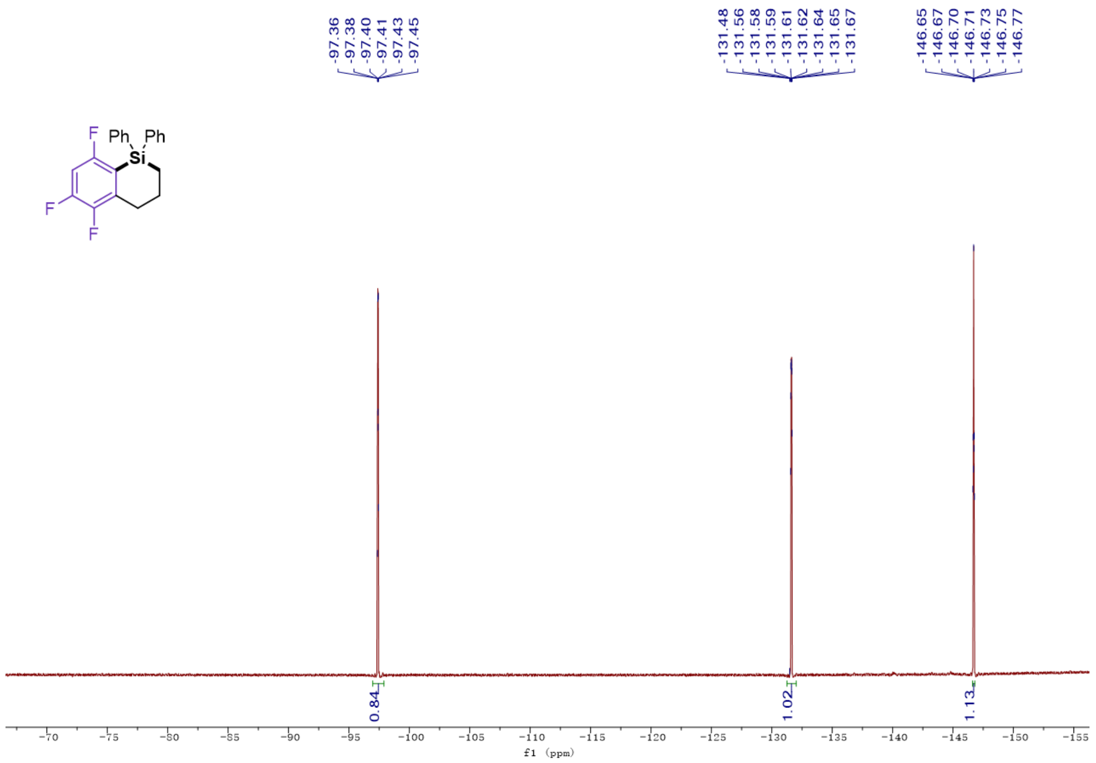

5,6,8-trifluoro-1,1-diphenyl-1,2,3,4-tetrahydrobenzo[*b*]siline (**4c**)

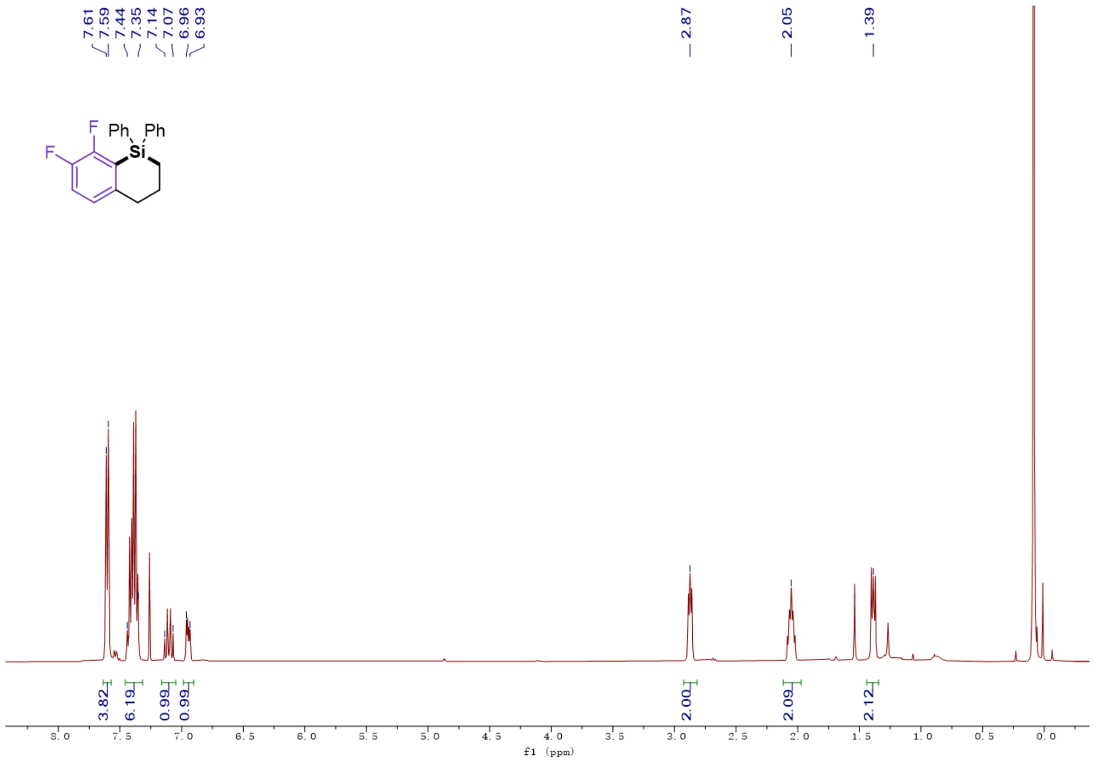

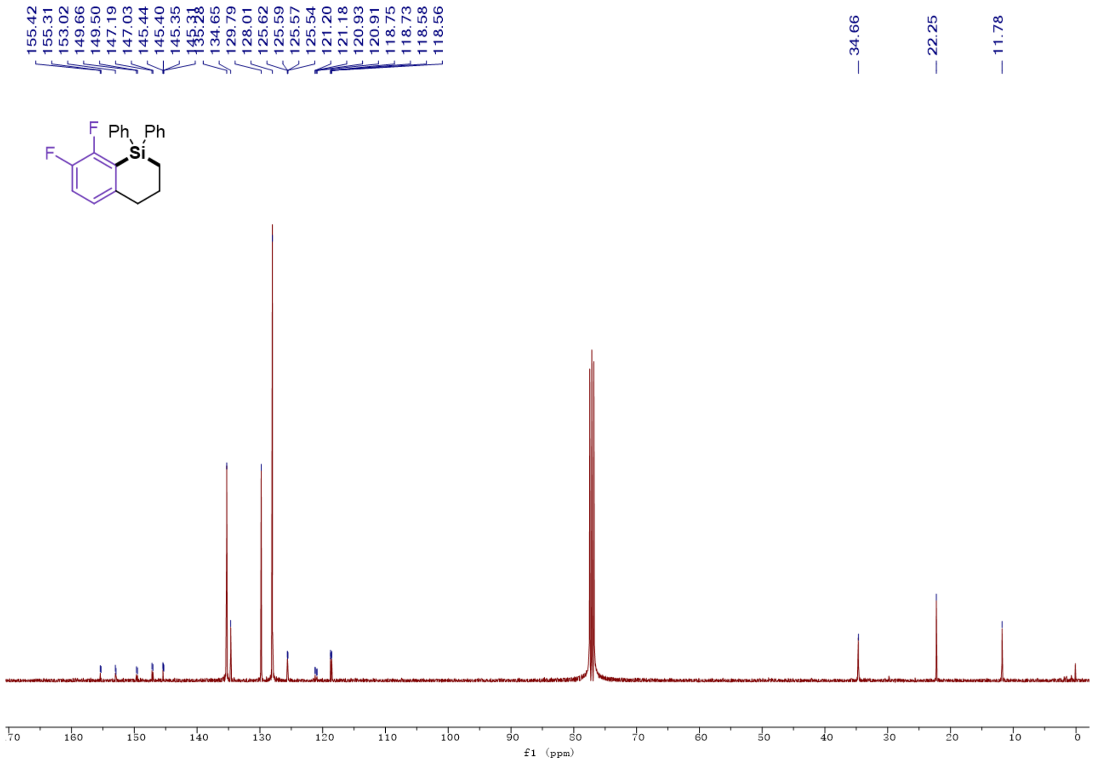

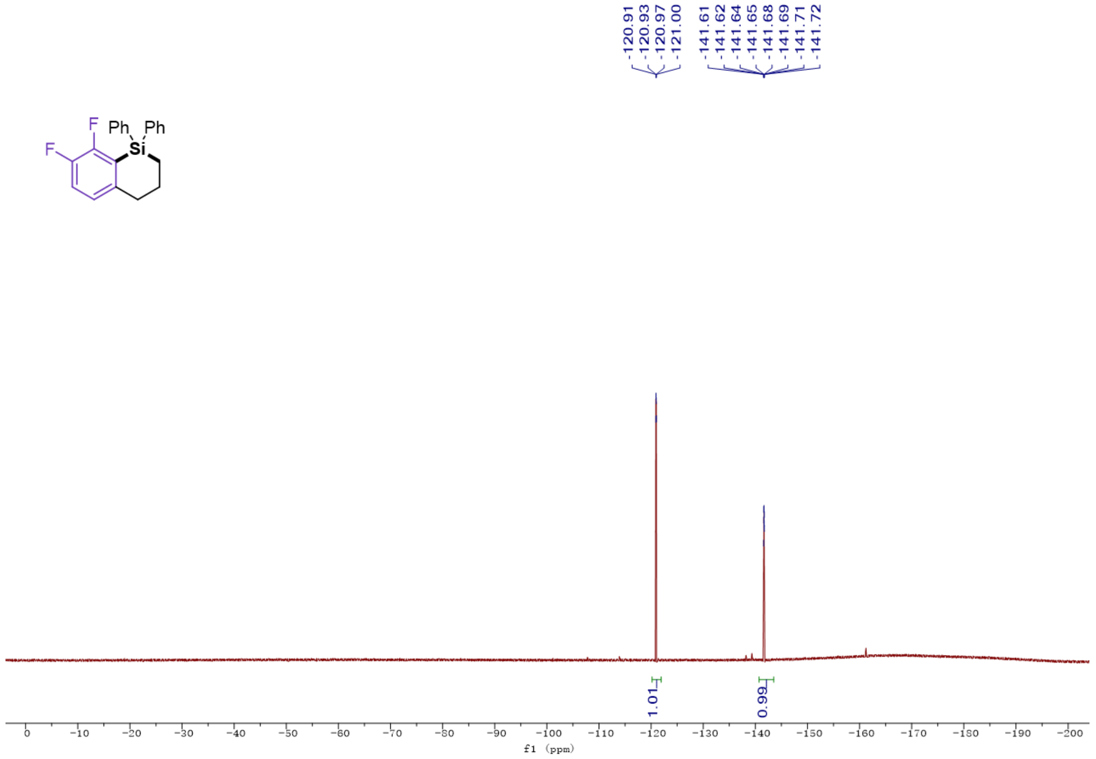

SUPPORTING INFORMATION

5,8-difluoro-1,1-diphenyl-1,2,3,4-tetrahydrobenzo[*b*]siline (**4d**)

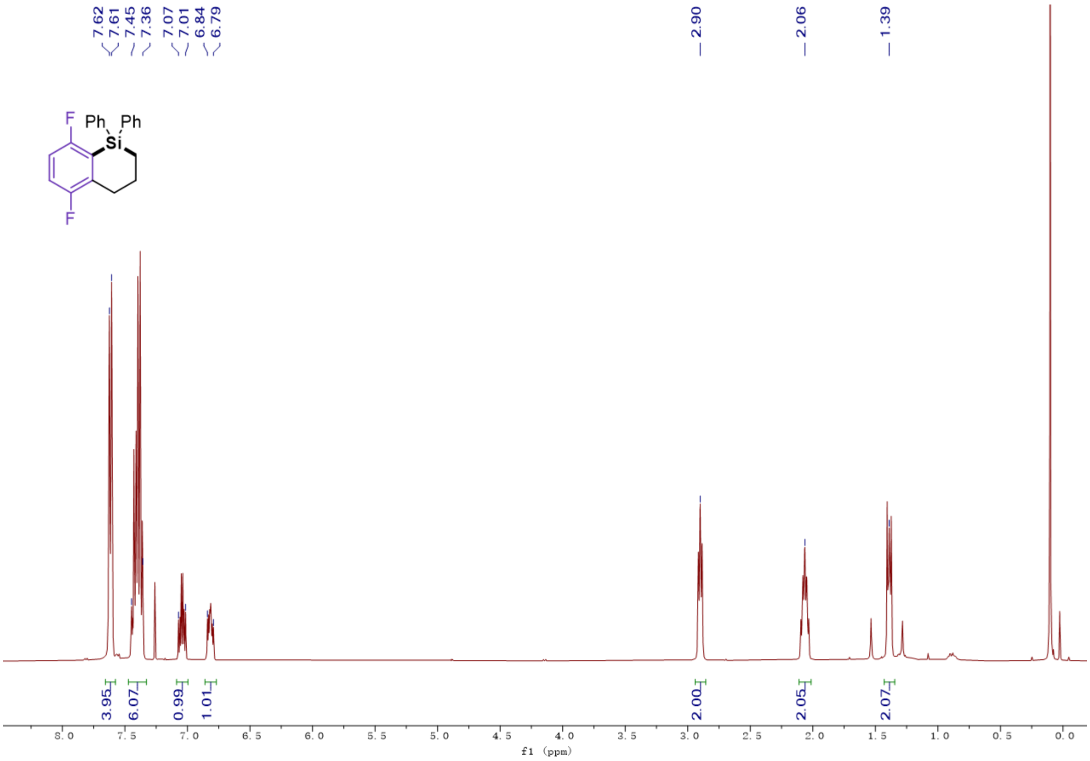

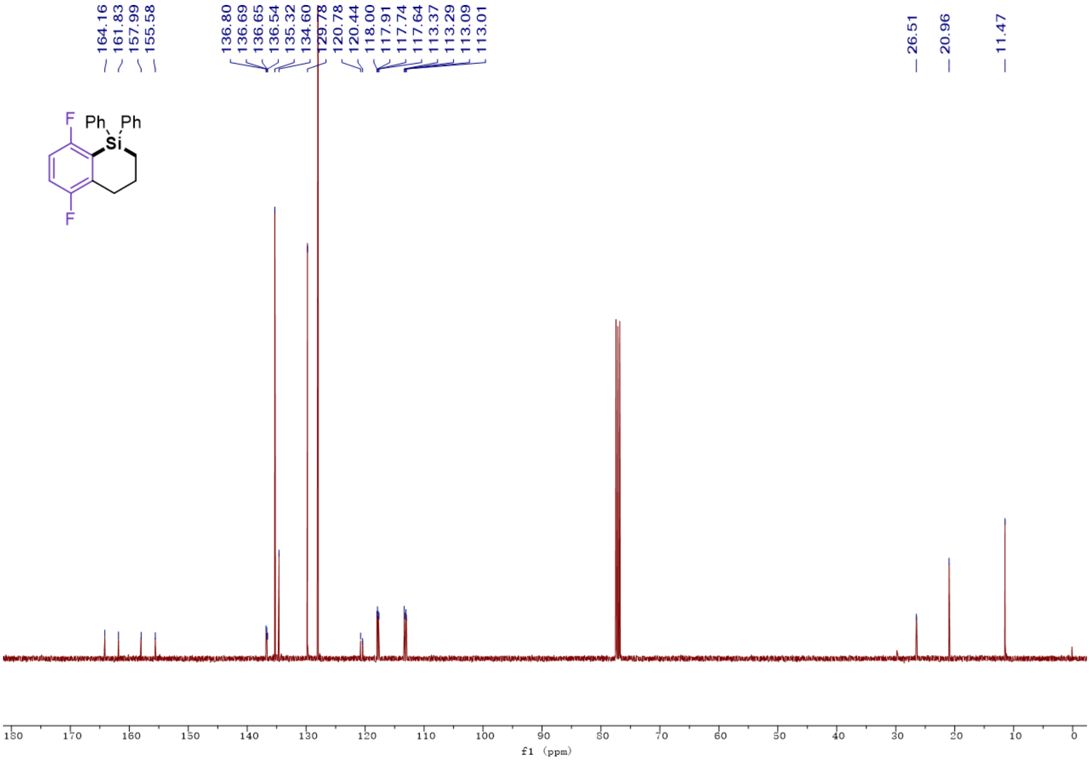

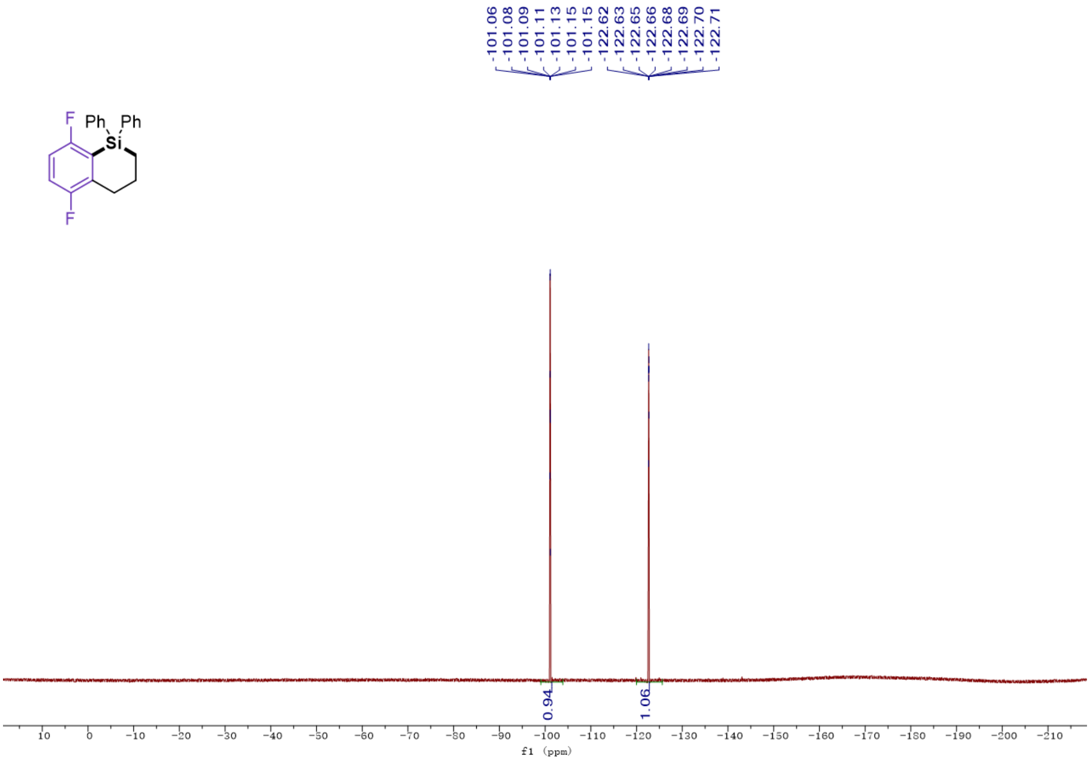

5,7-difluoro-1-methyl-1-phenyl-1,2,3,4-tetrahydrobenzo[*b*]siline (**4e**)

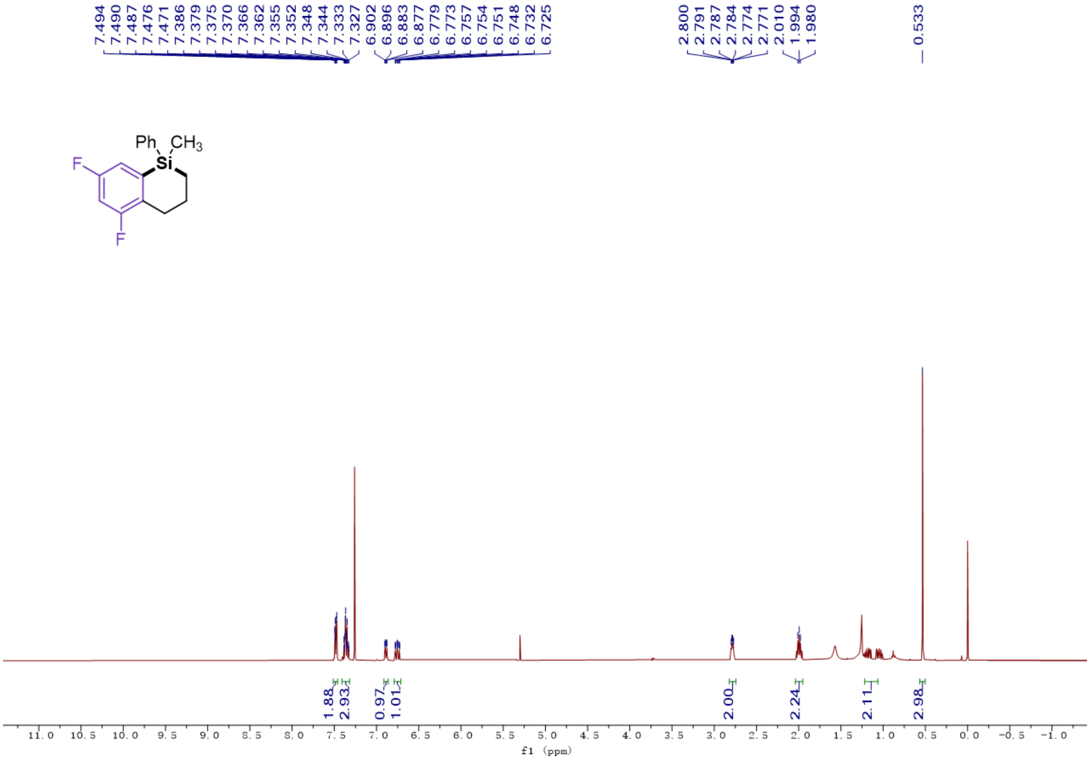

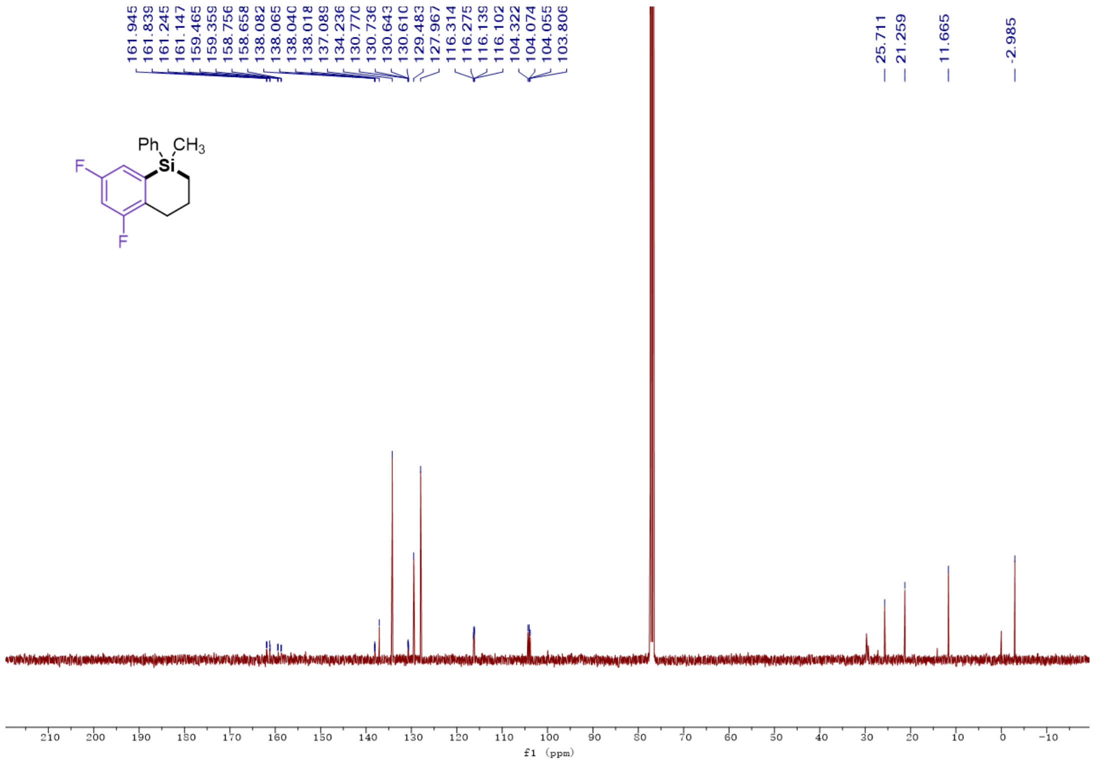
161.945
161.839
161.245
161.147
159.465
159.359
158.756
158.658
138.082
138.065
138.040
138.018
137.089
134.236
130.770
130.736
130.643
130.610
129.483
127.967
116.314
116.275
116.139
116.102
104.322
104.074
104.055
103.806
25.711
21.259
11.665
-2.985
Ph
CH3
Si
F
F
f1 (ppm)

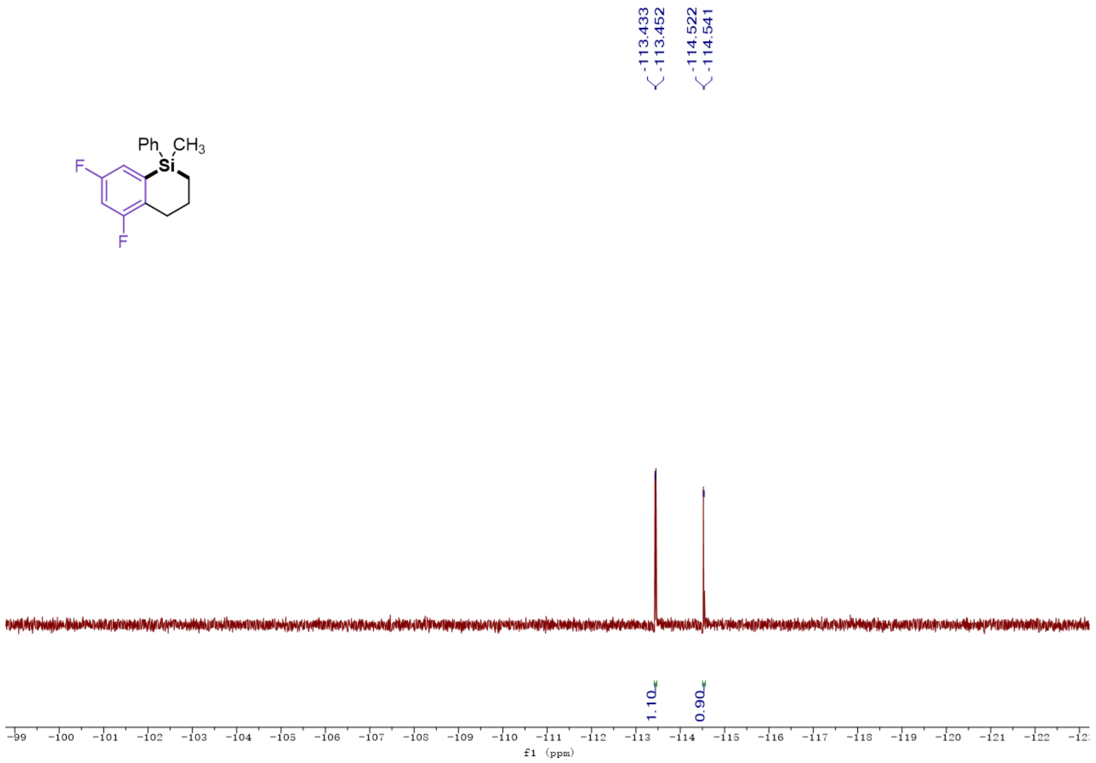
-113.433
-113.452
-114.522
-114.541
Ph
CH3
Si
F
F
1.10
0.90
f1 (ppm)

SUPPORTING INFORMATION

8-fluoro-1,1-diphenyl-1,2,3,4-tetrahydrobenzo[*b*]siline (**4f**)

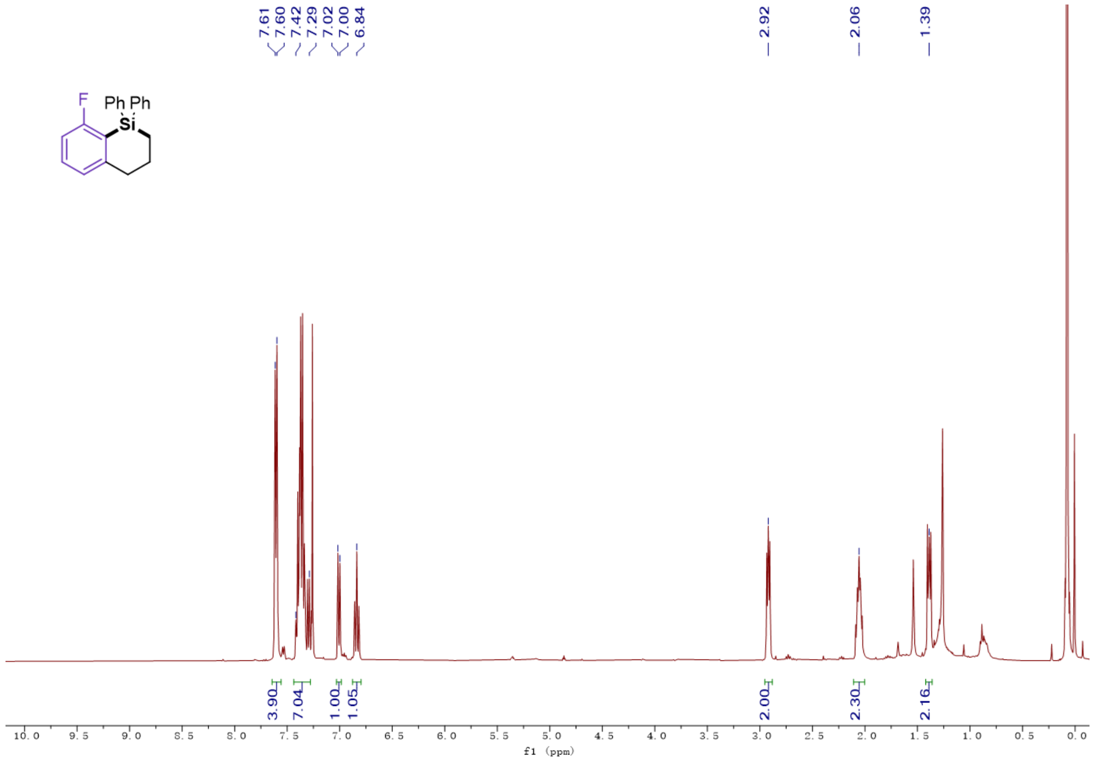

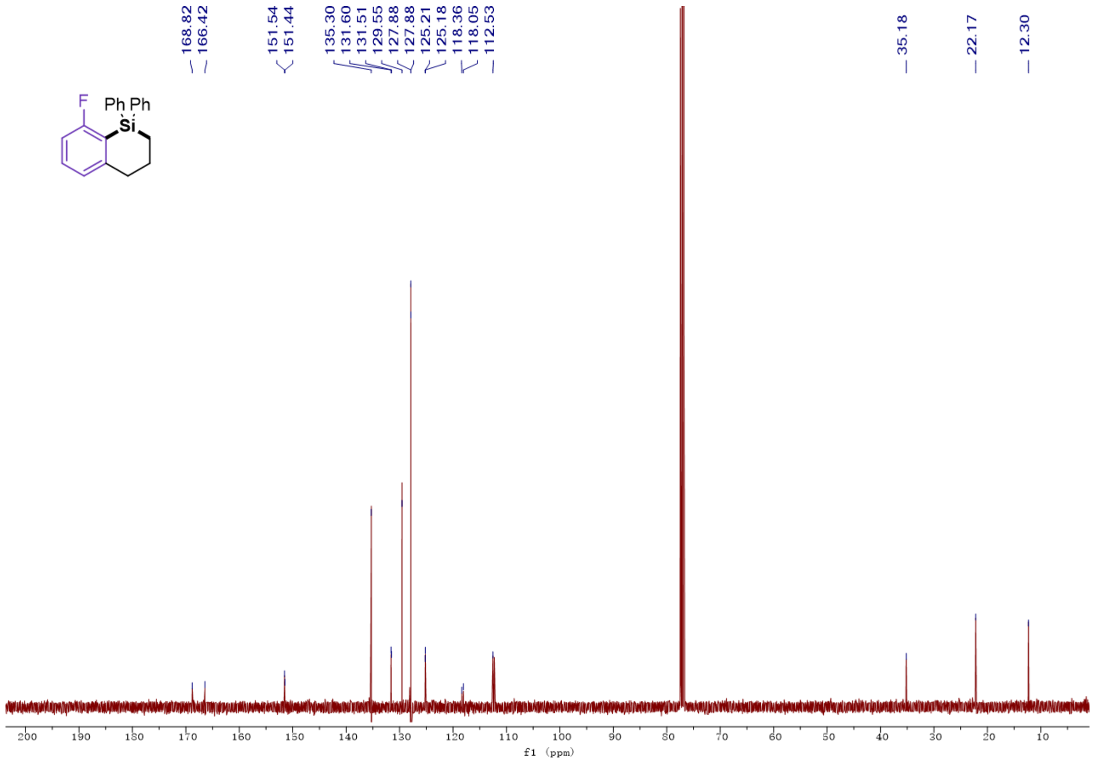

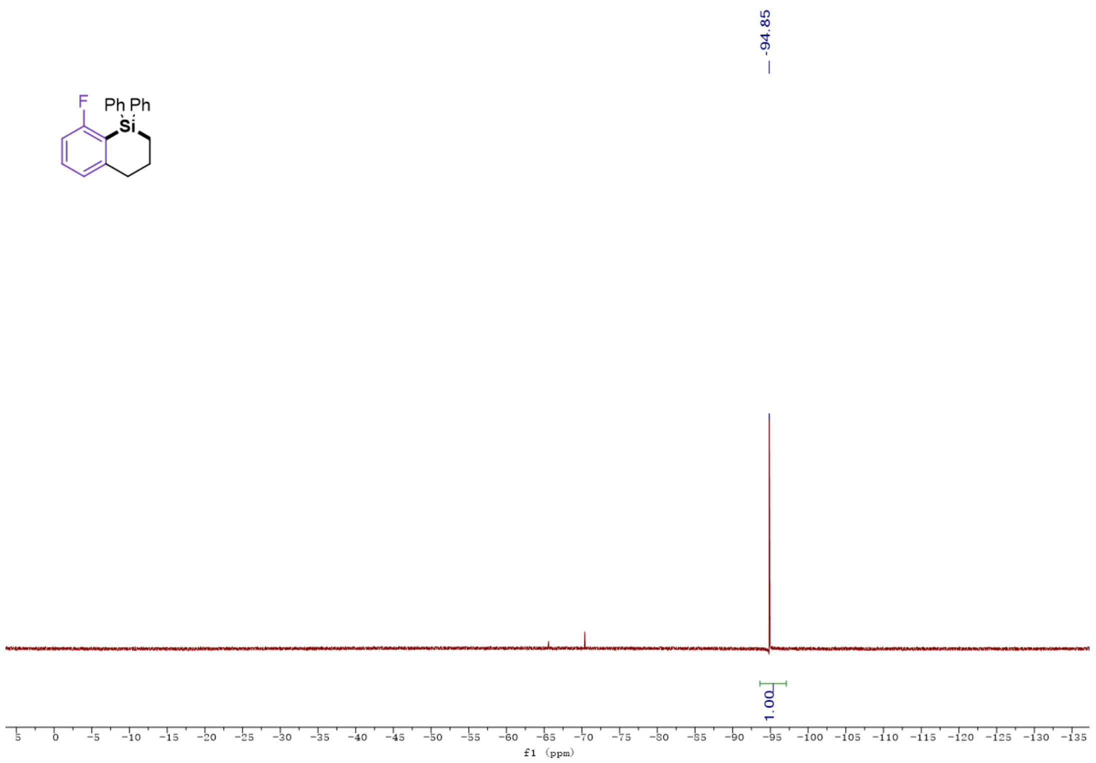

8-chloro-5-fluoro-1,1-diphenyl-1,2,3,4-tetrahydrobenzo[*b*]siline (**4g**)

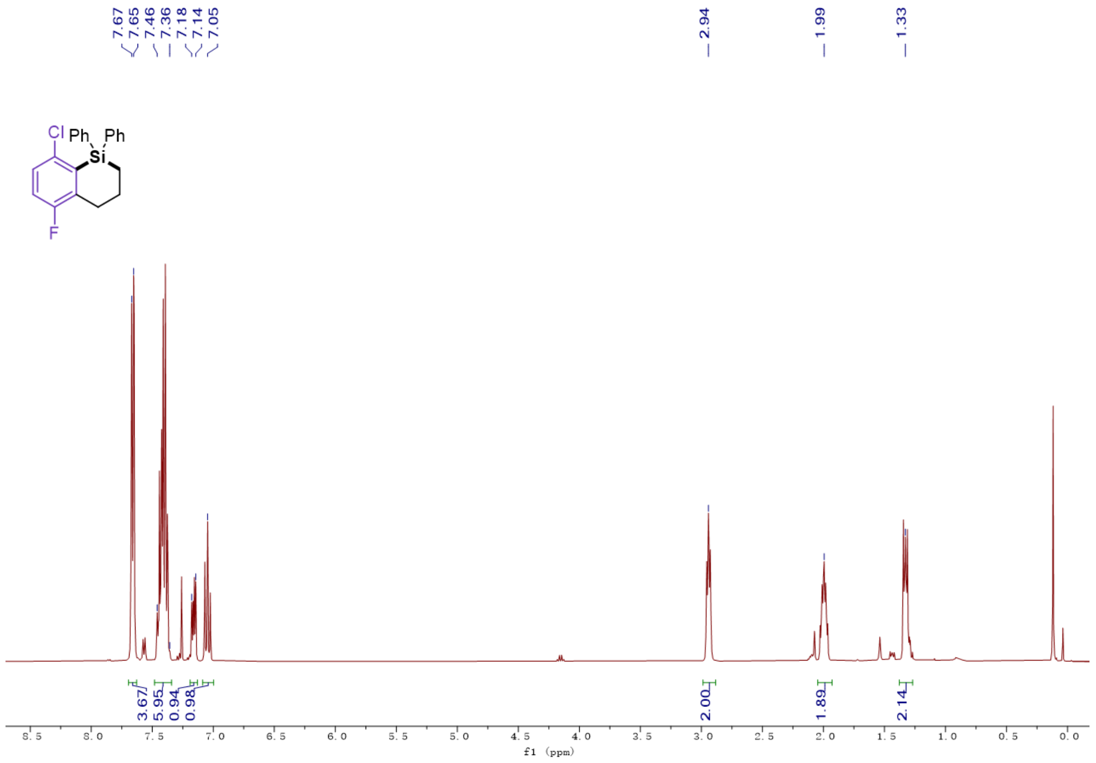

SUPPORTING INFORMATION

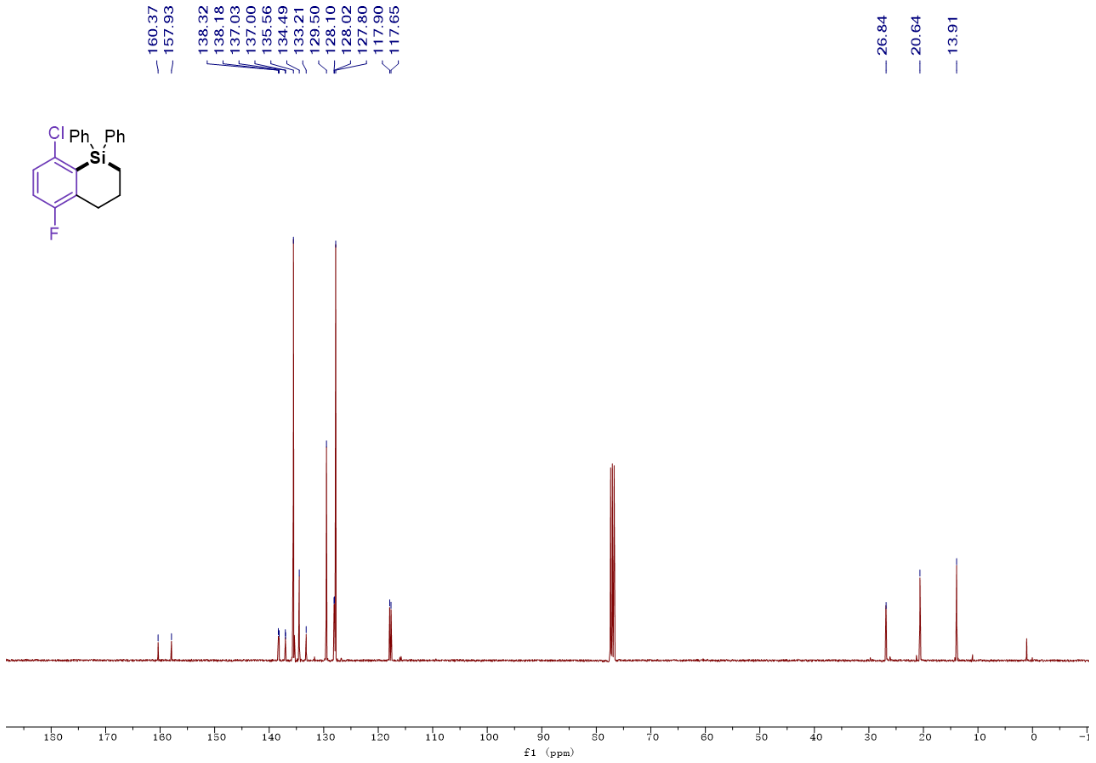

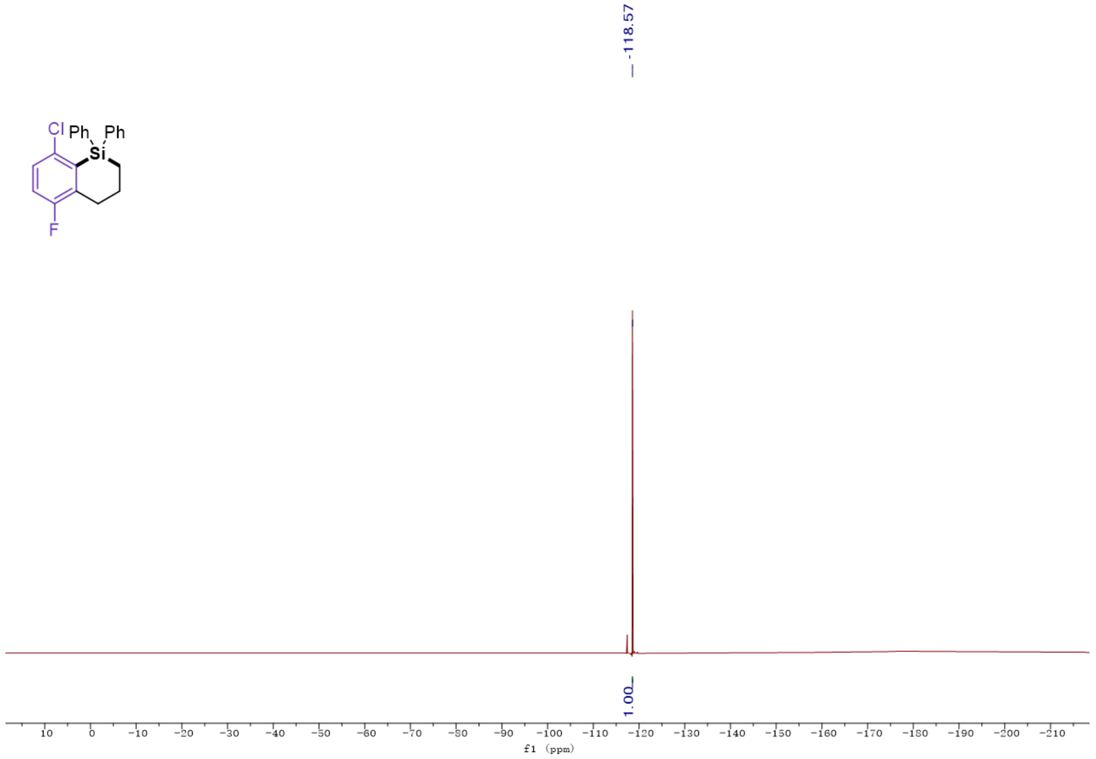

SUPPORTING INFORMATION

8-chloro-1,1-diphenyl-1,2,3,4-tetrahydrobenzo[*b*]siline (**4h**)

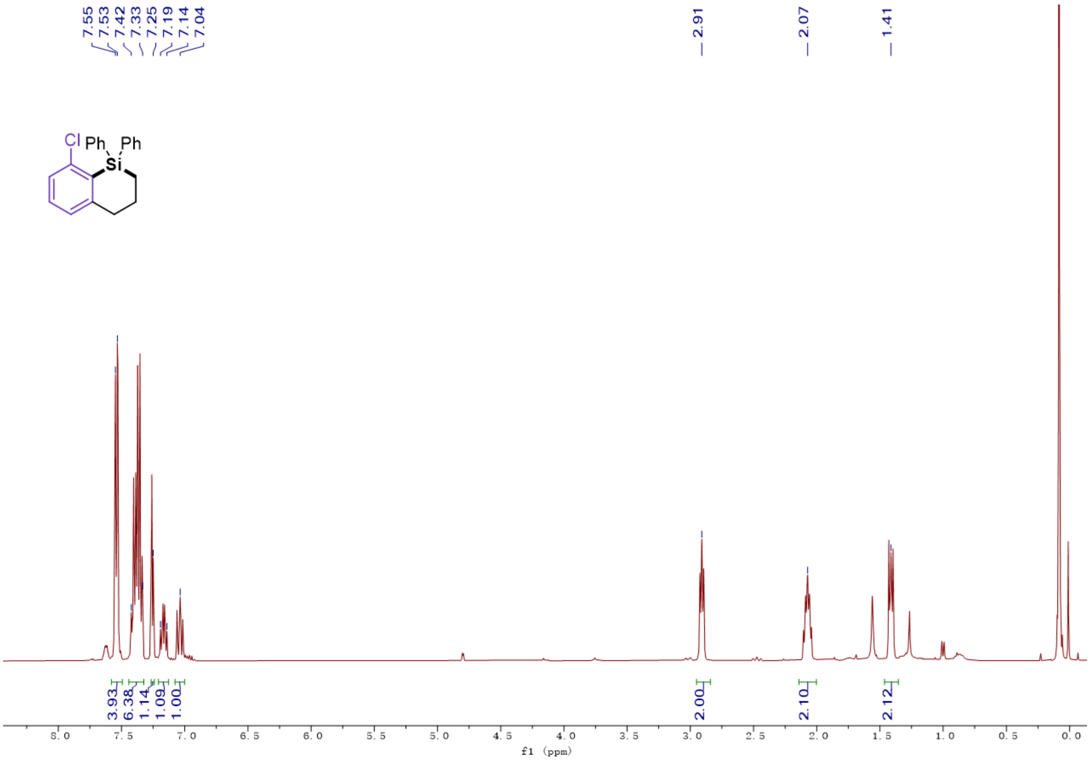

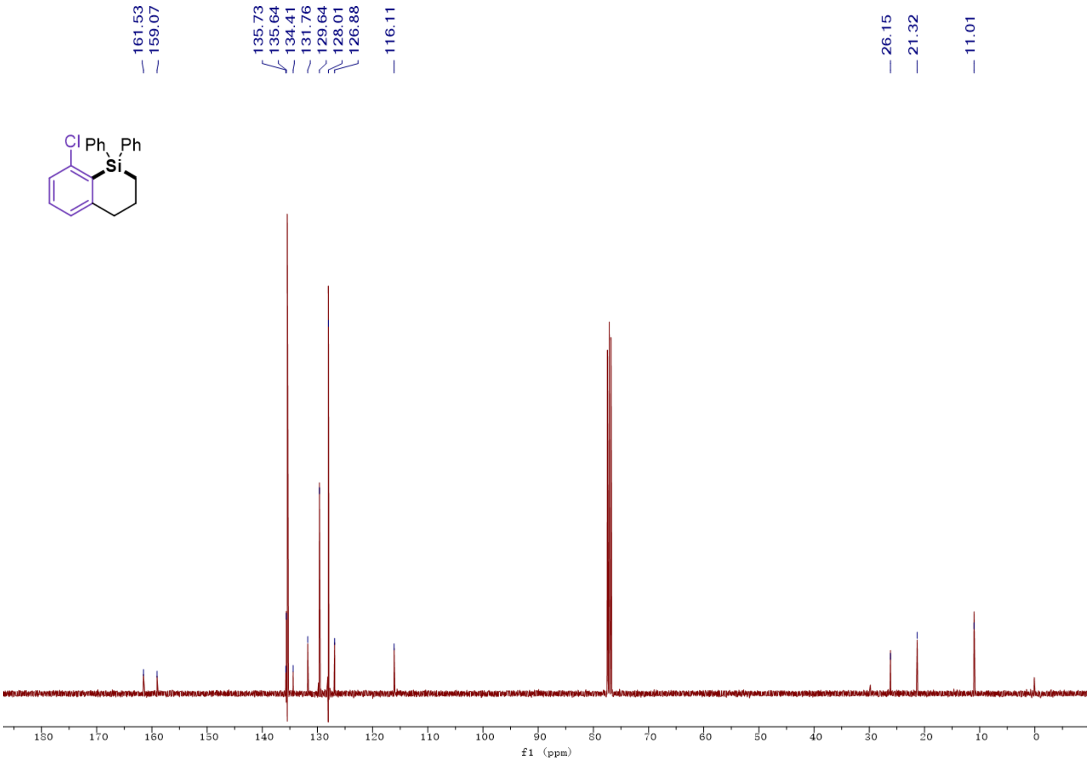

SUPPORTING INFORMATION

4,5,6,7-tetrafluoro-1,1-diphenyl-2,3-dihydro-1H-benzo[*b*]silole (**4i**)

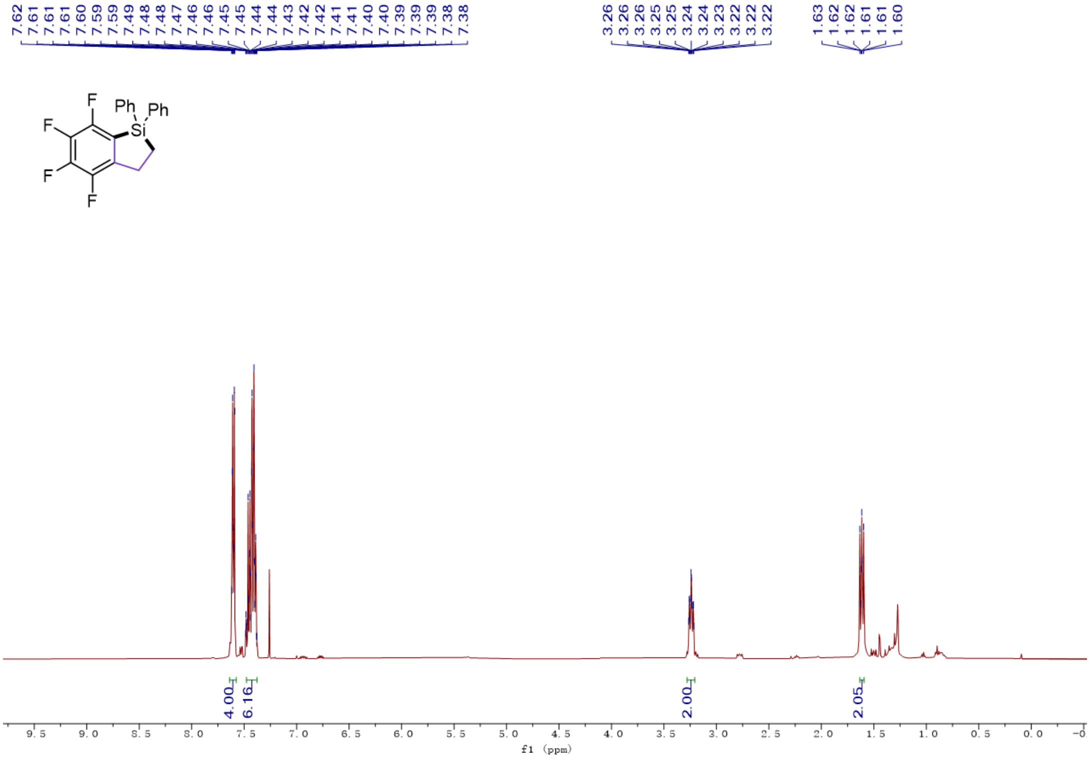

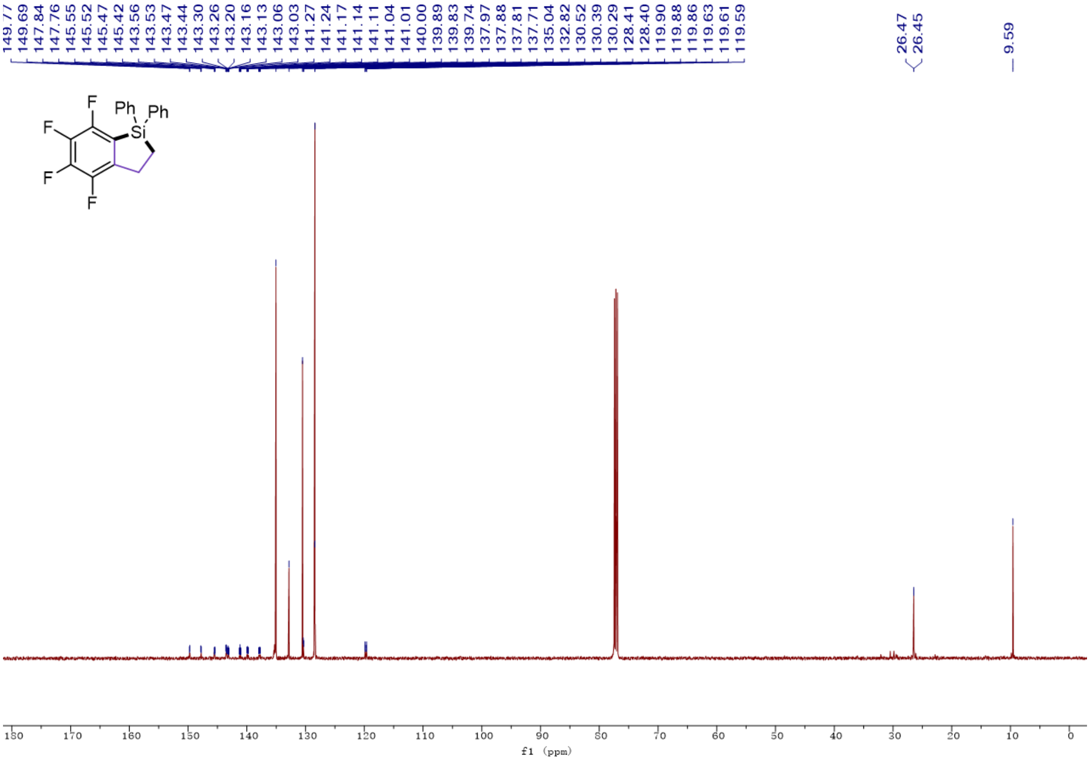

SUPPORTING INFORMATION

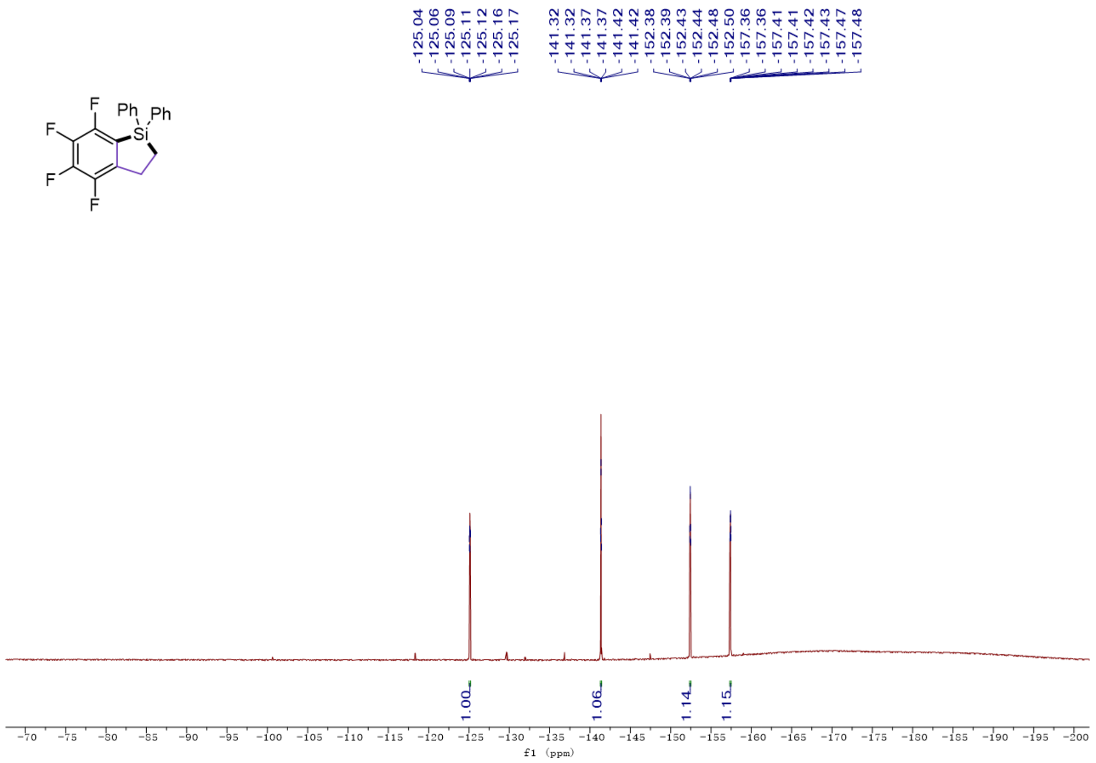

6,7,8,9-tetrafluoro-1,1-diphenyl-2,3,4,5-tetrahydro-1H-benzo[*b*]silepine (**4j**)

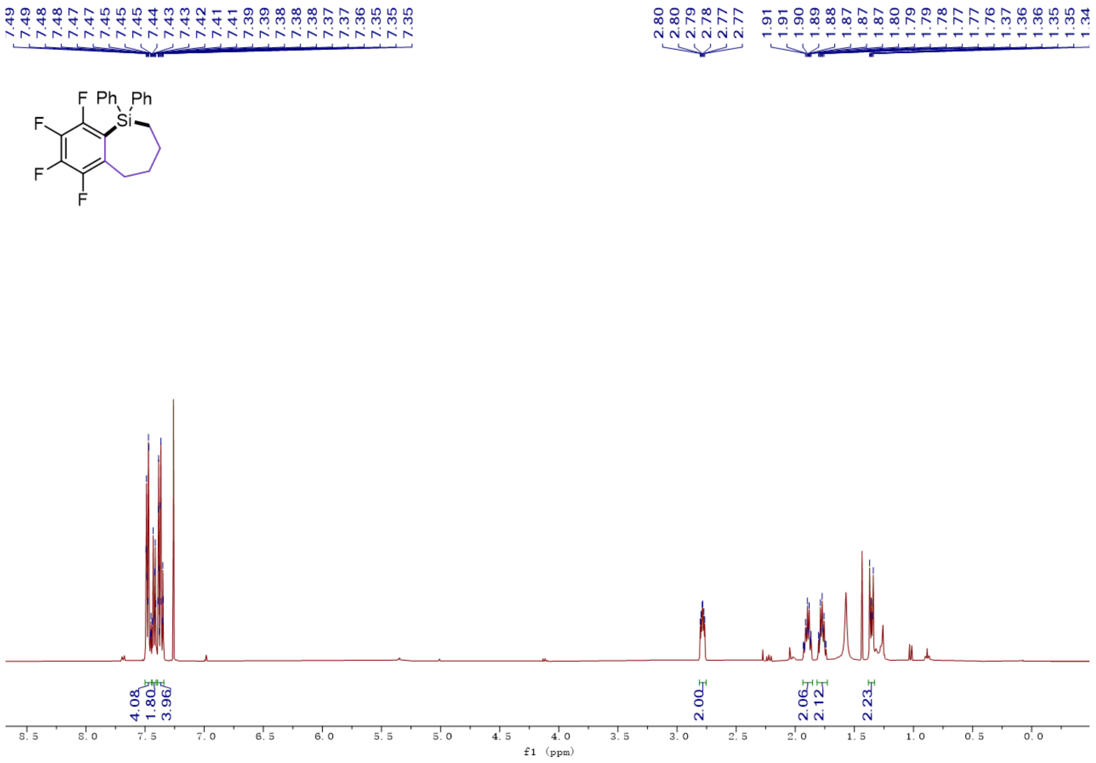

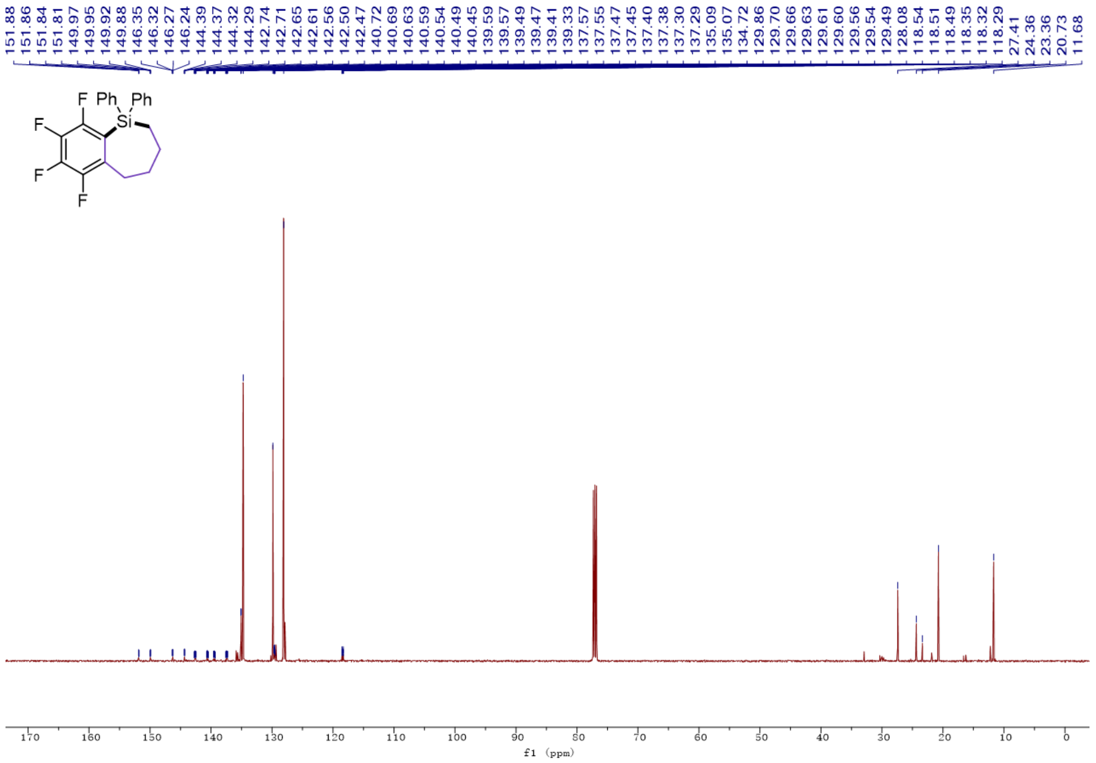
F
Ph
Ph
Si
F
F
F
f1 (ppm)

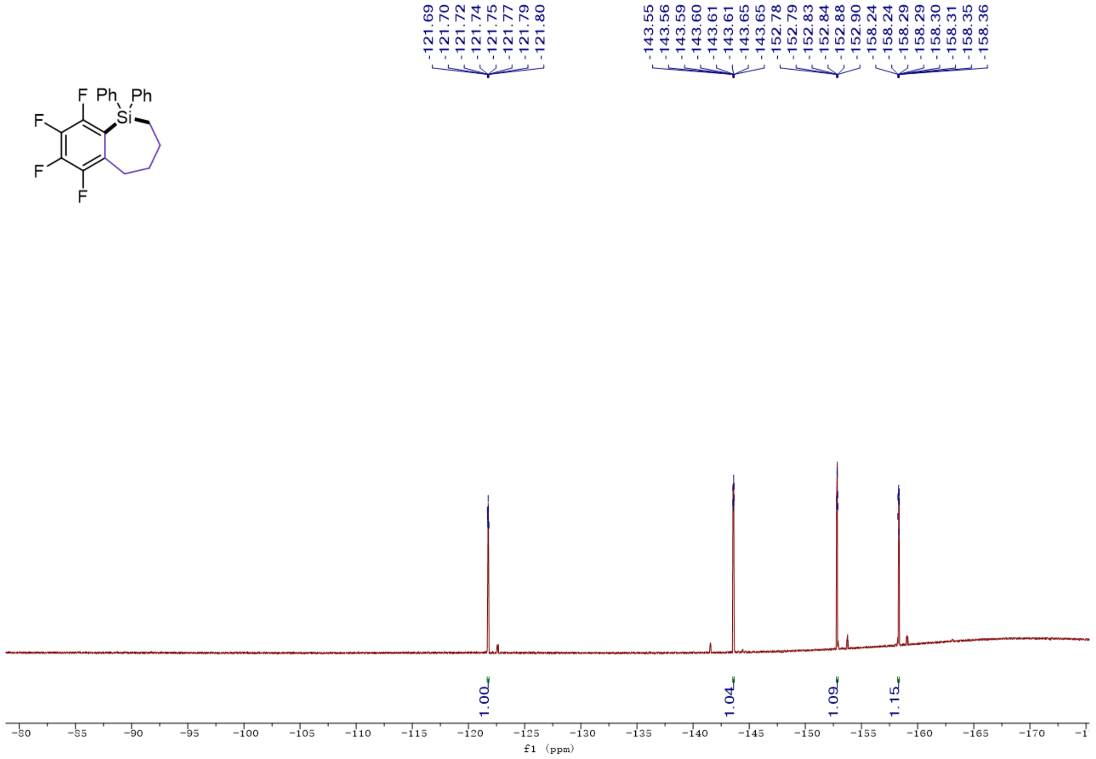
F
Ph
Ph
Si
F
F
F
1.00
1.04
1.09
1.15
f1 (ppm)

SUPPORTING INFORMATION

1,1-diphenyl-1,2,3,4-tetrahydrobenzo[*b*]siline (**6a**)

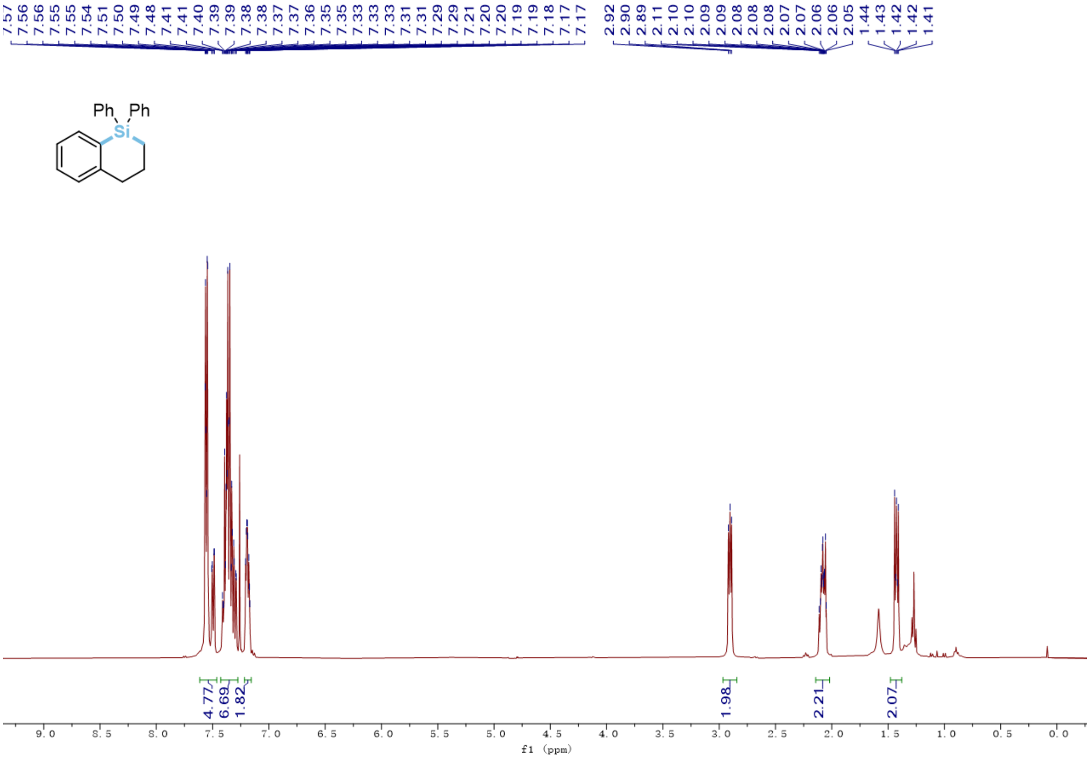

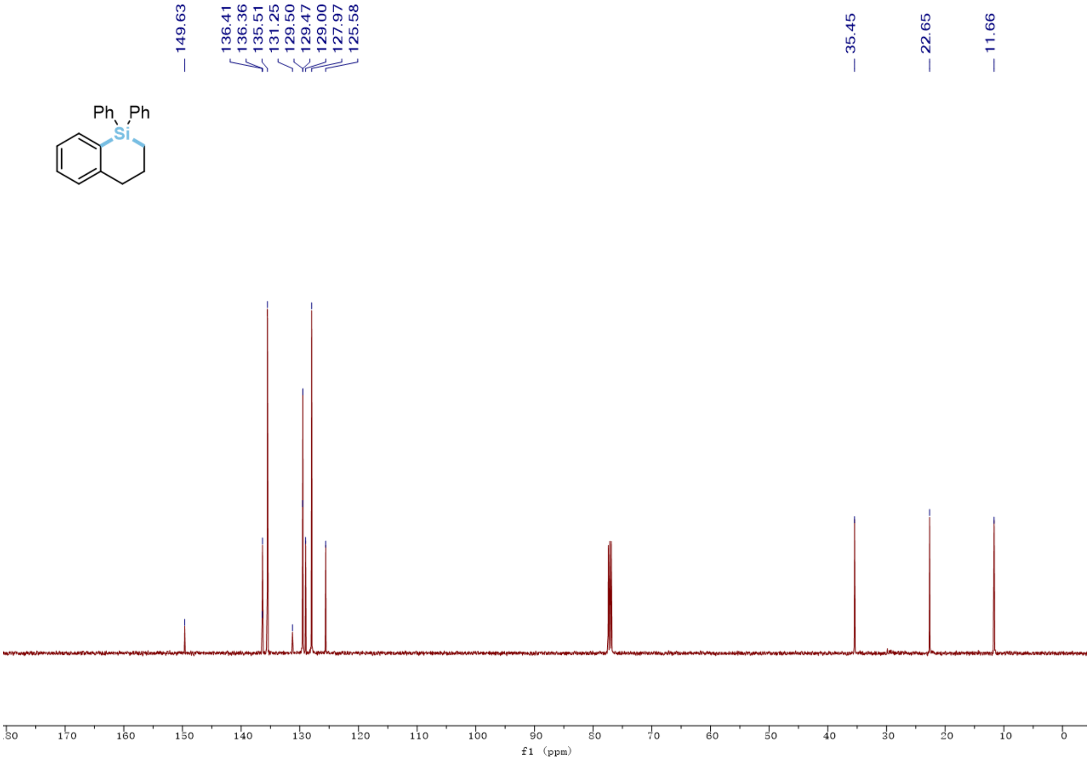

8-methyl-1,1-diphenyl-1,2,3,4-tetrahydrobenzo[*b*]siline (**6b**)

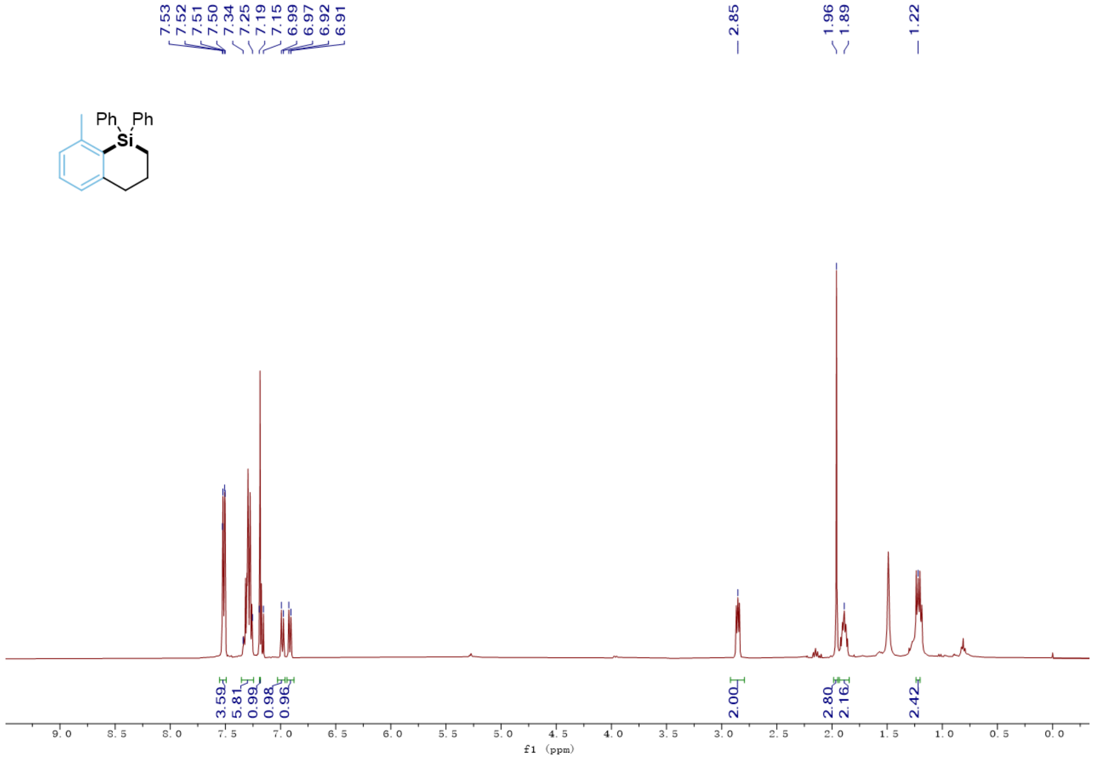

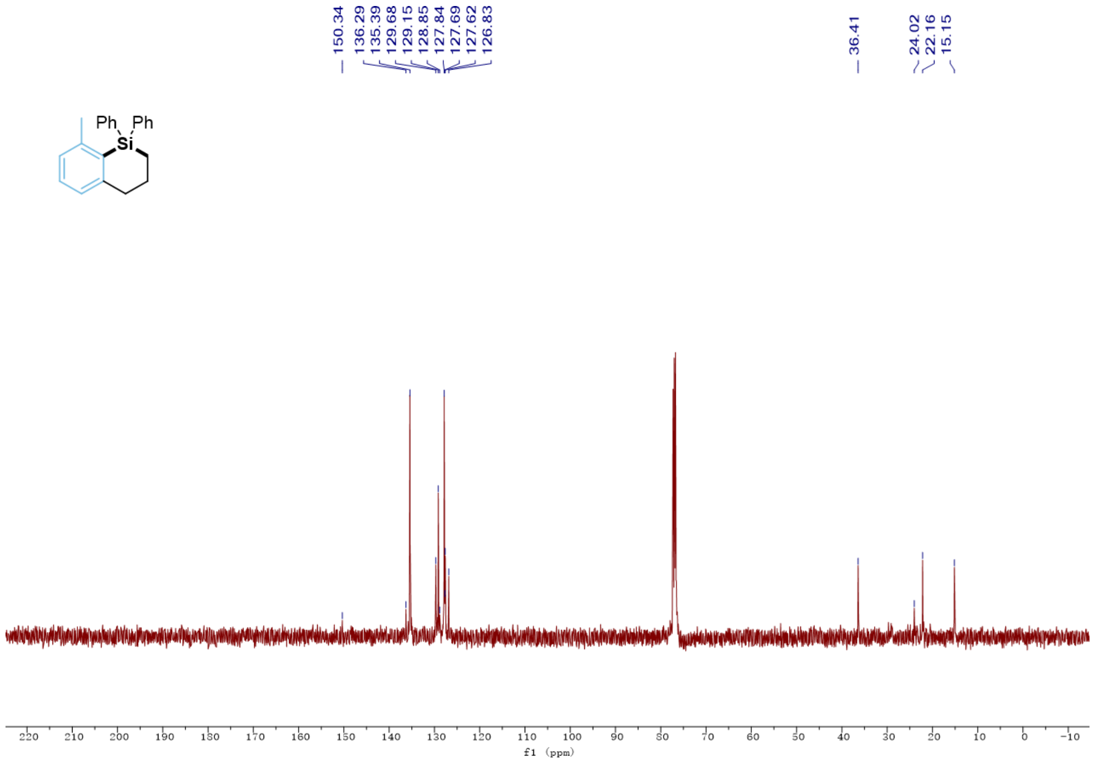

SUPPORTING INFORMATION

6-methyl-1,1-diphenyl-1,2,3,4-tetrahydrobenzo[*b*]siline (**6b′**)

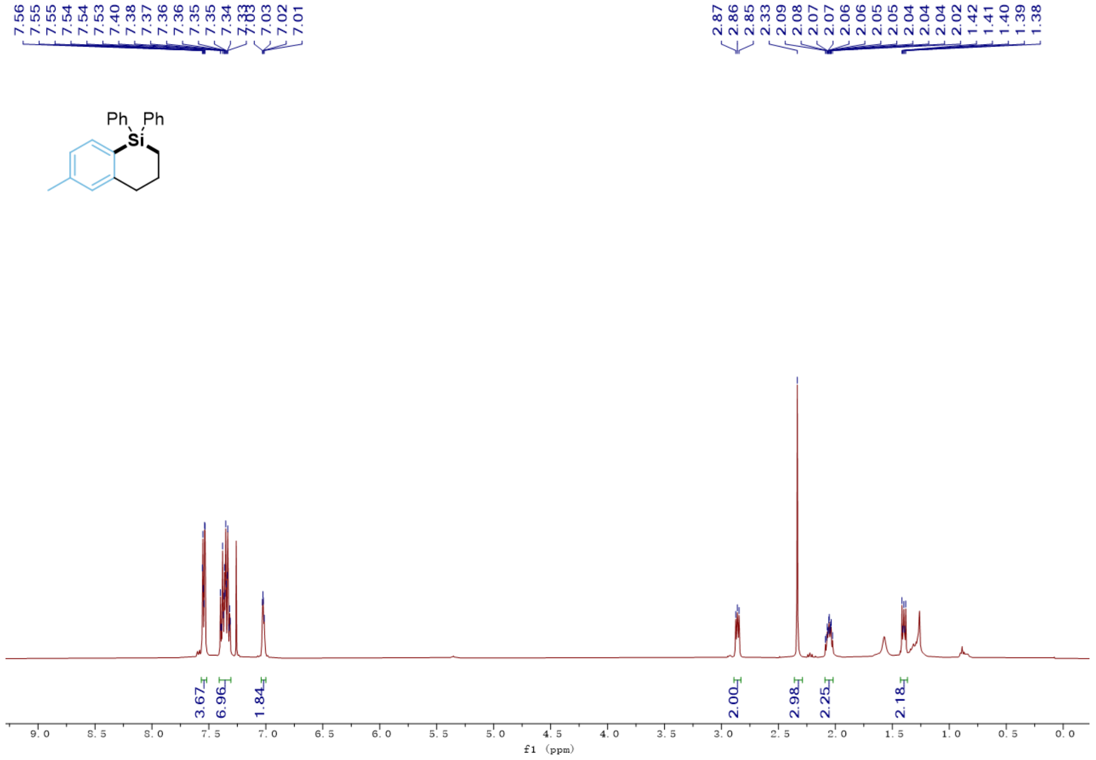

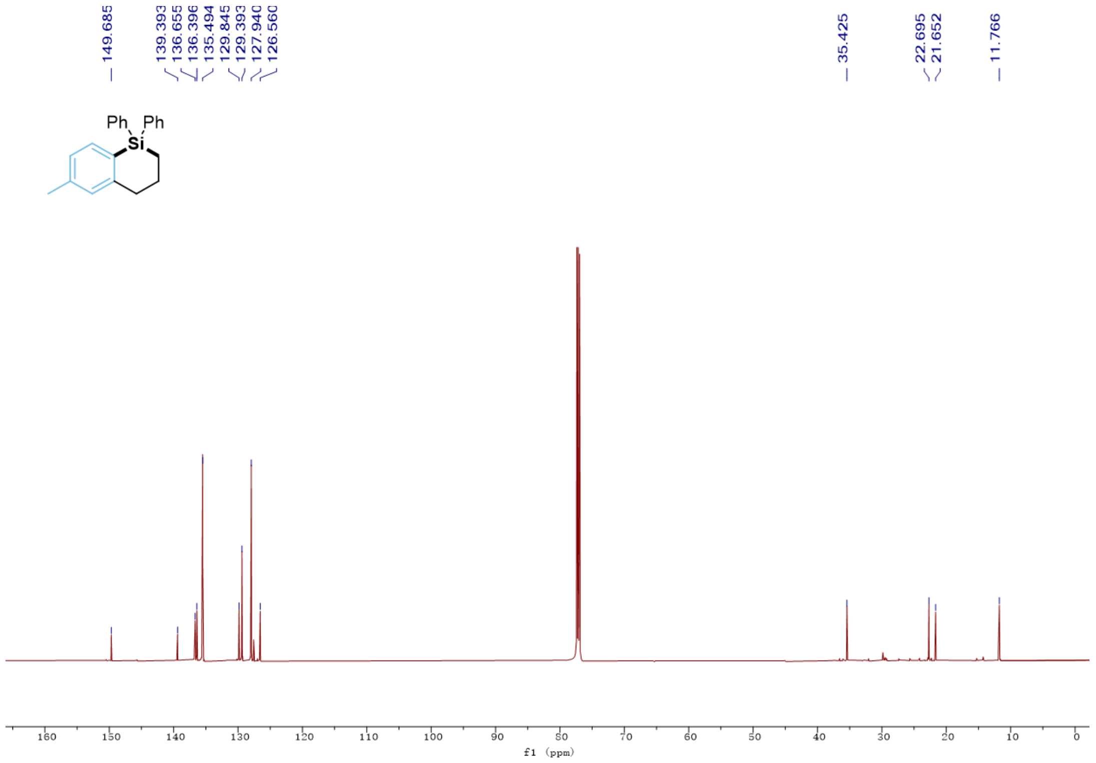

SUPPORTING INFORMATION

5-methyl-1,1-diphenyl-1,2,3,4-tetrahydrobenzo[*b*]siline (**6c**)

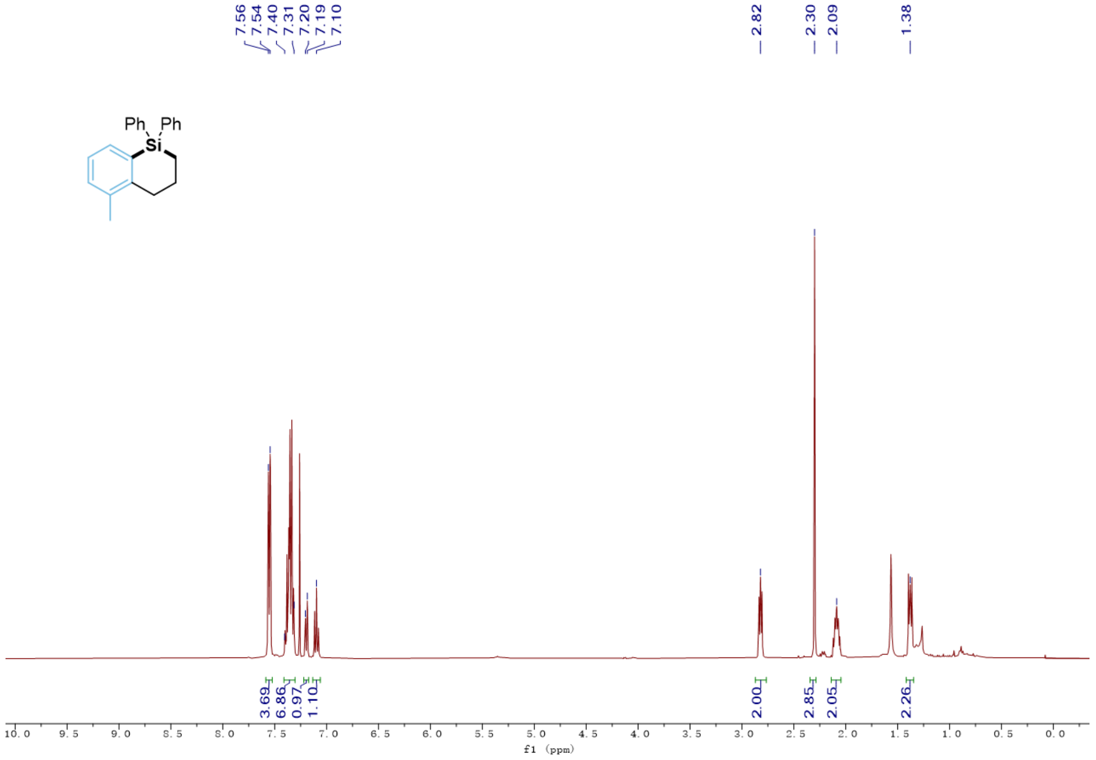

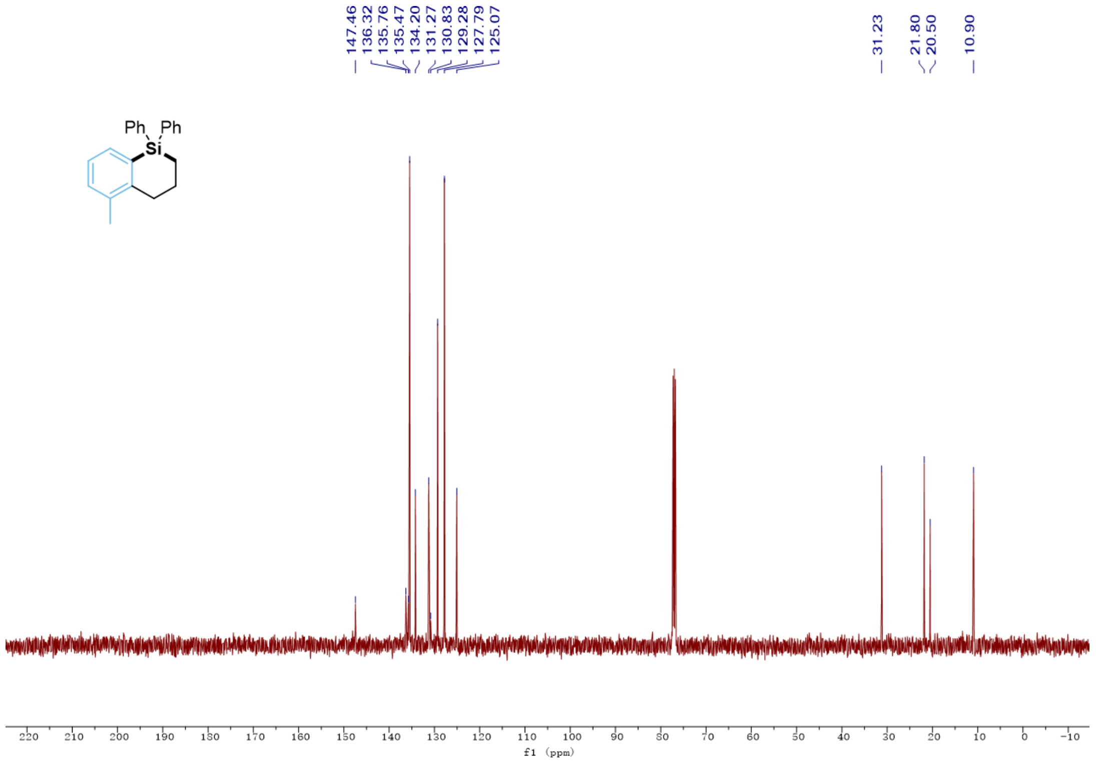

SUPPORTING INFORMATION

7-methyl-1,1-diphenyl-1,2,3,4-tetrahydrobenzo[*b*]siline (**6d**)

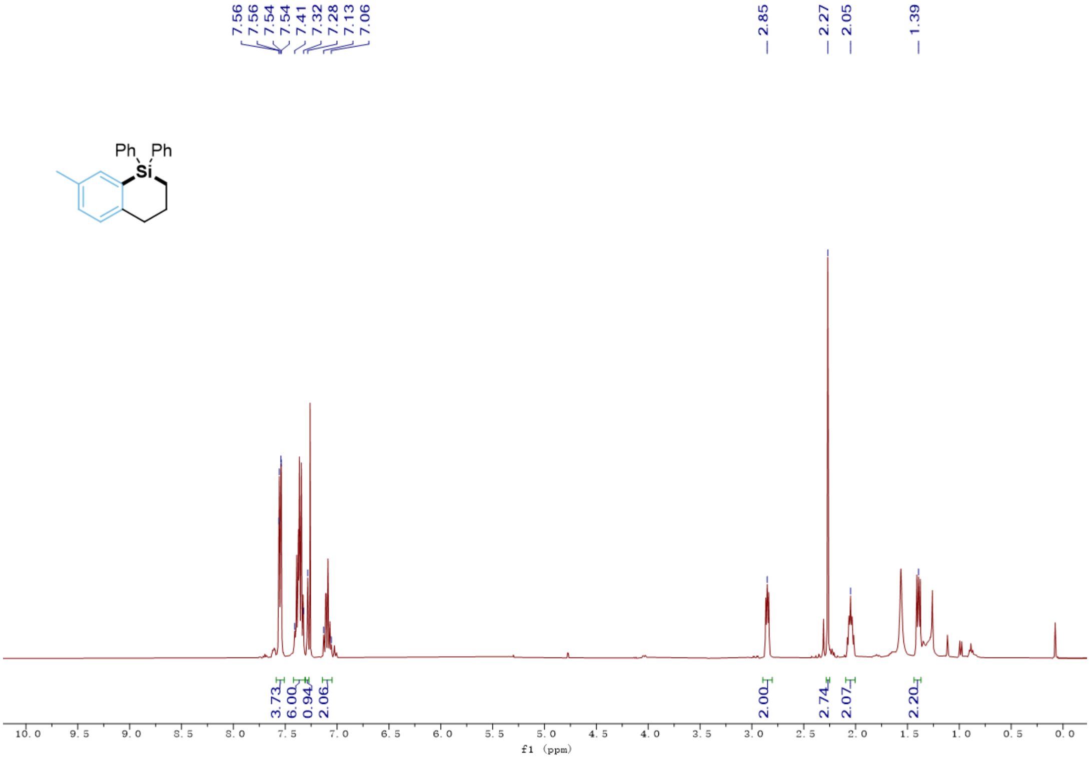

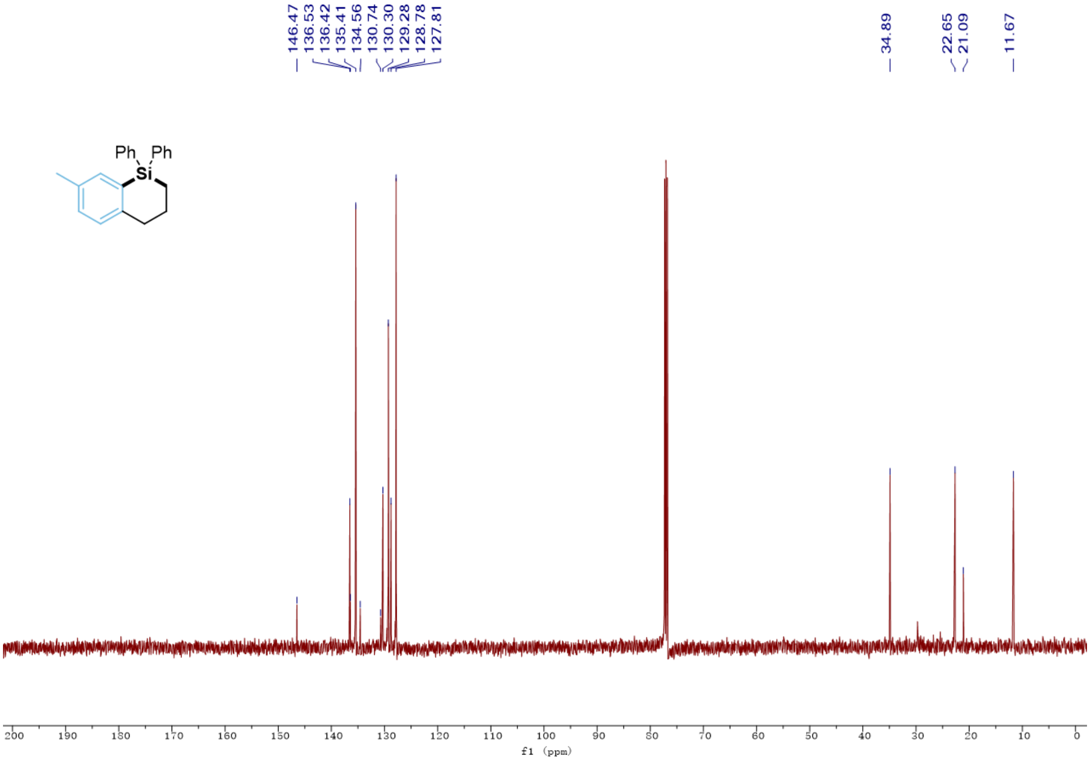

SUPPORTING INFORMATION

1,1,7-triphenyl-1,2,3,4-tetrahydrobenzo[*b*]siline (**6e**)

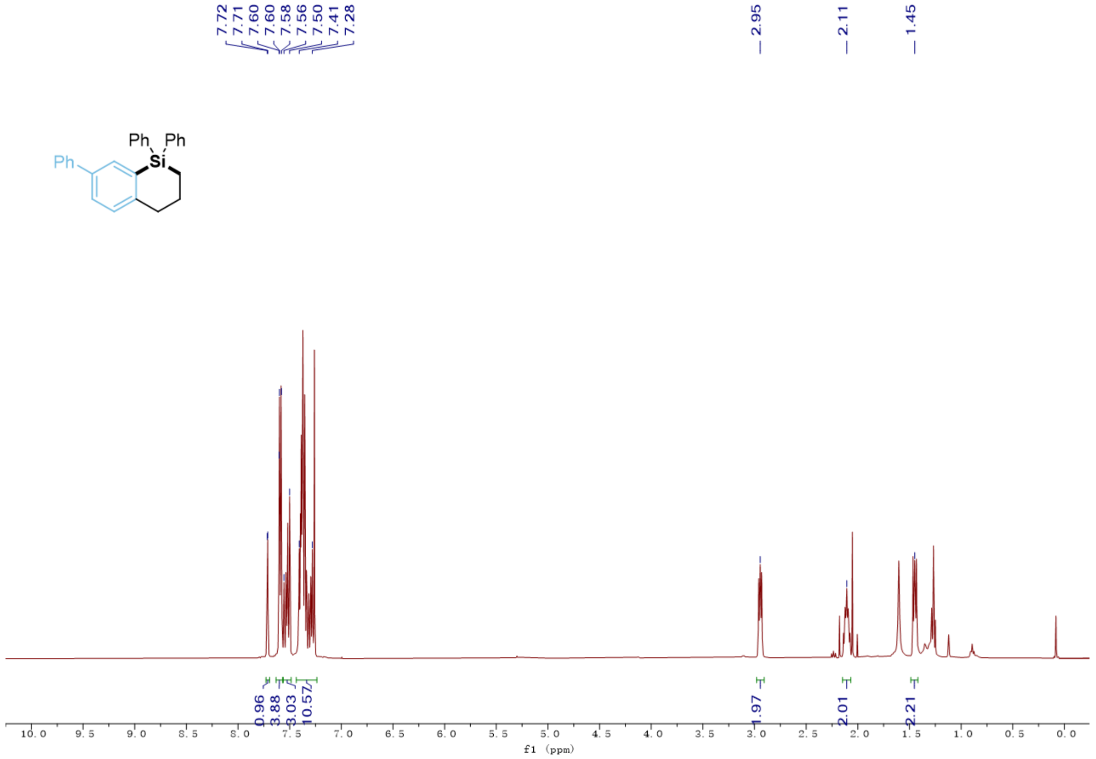

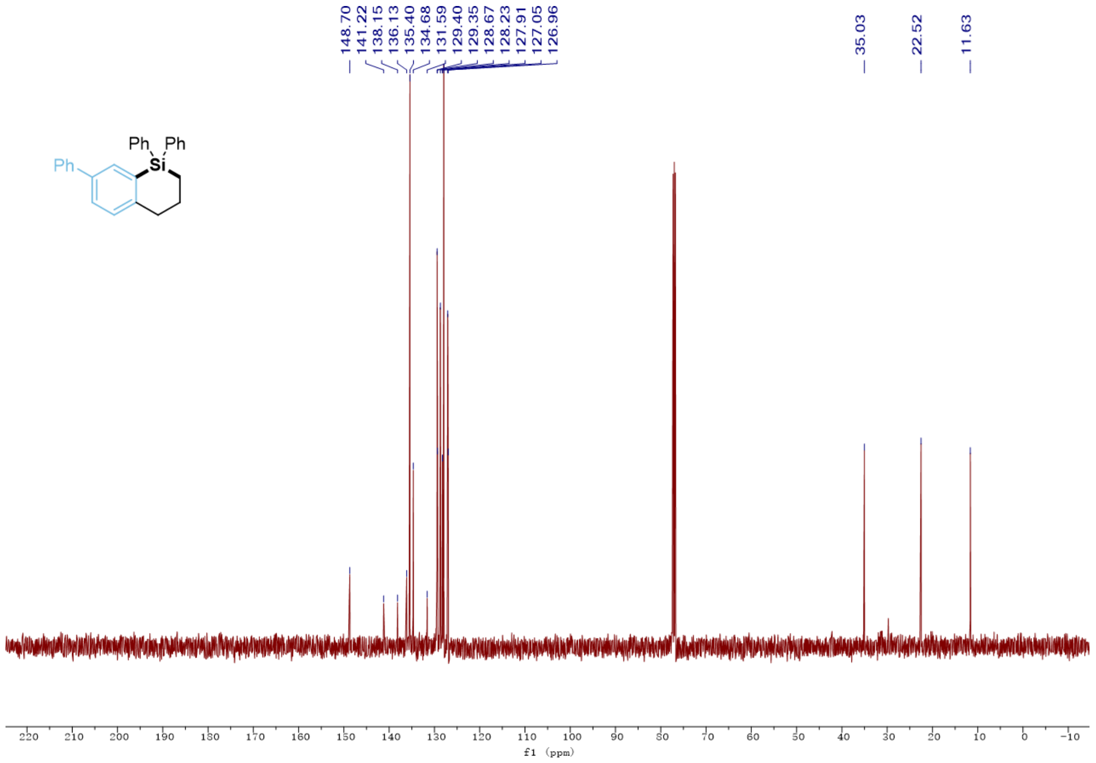

SUPPORTING INFORMATION

7-chloro-1,1-diphenyl-1,2,3,4-tetrahydrobenzo[*b*]siline (**6f**)

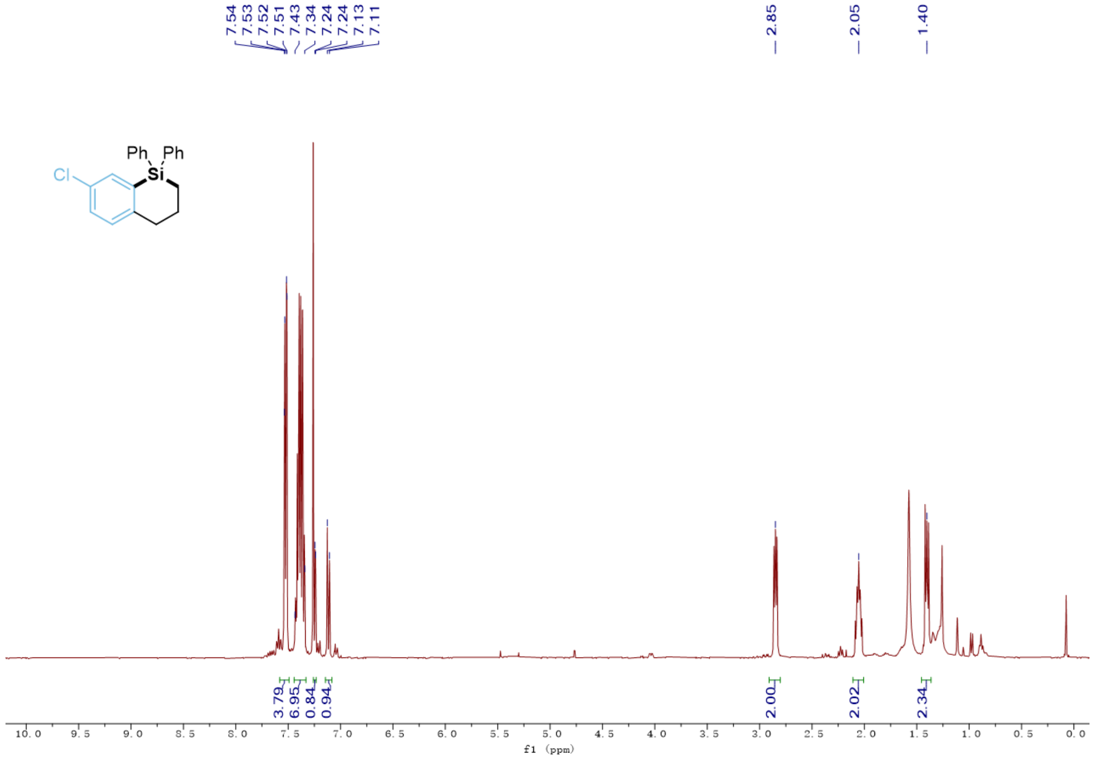

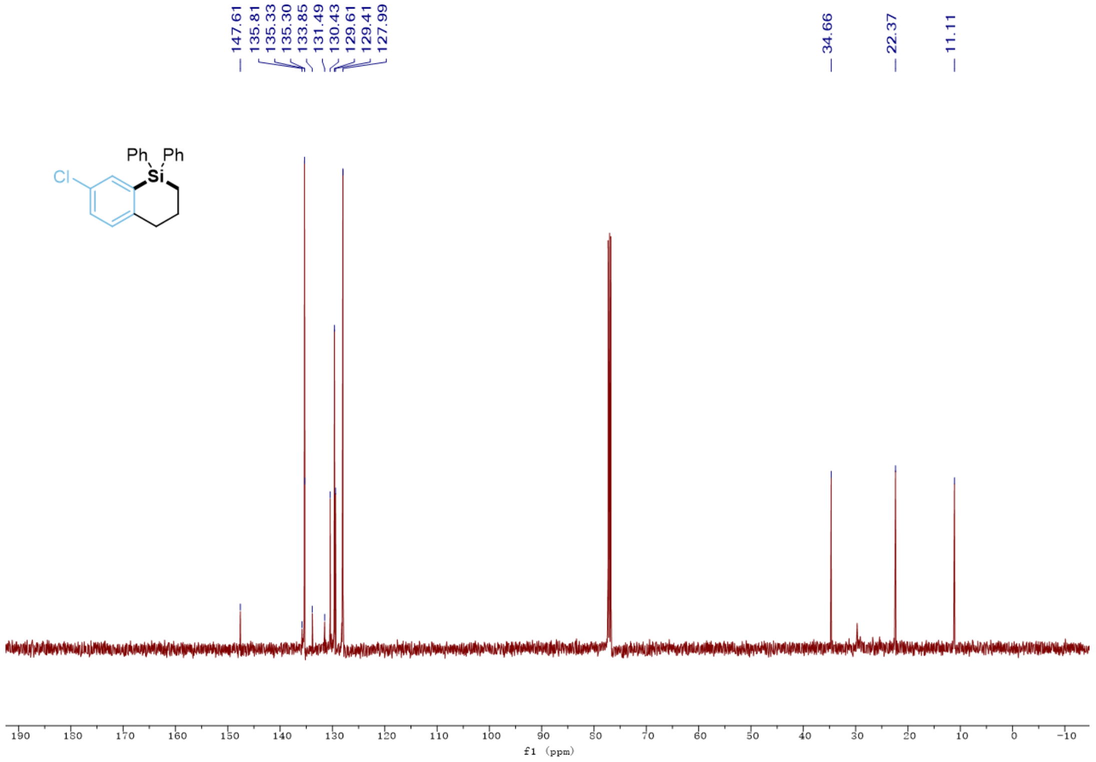

SUPPORTING INFORMATION

7-fluoro-1,1-diphenyl-1,2,3,4-tetrahydrobenzo[*b*]siline (**6g**)

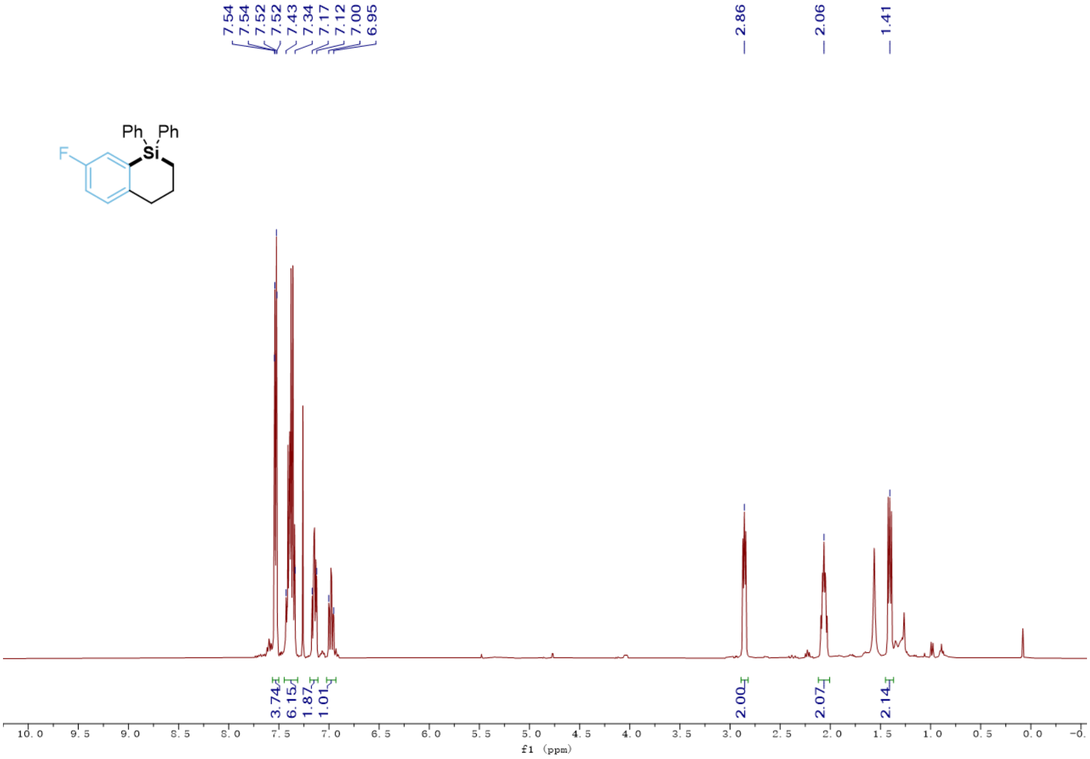

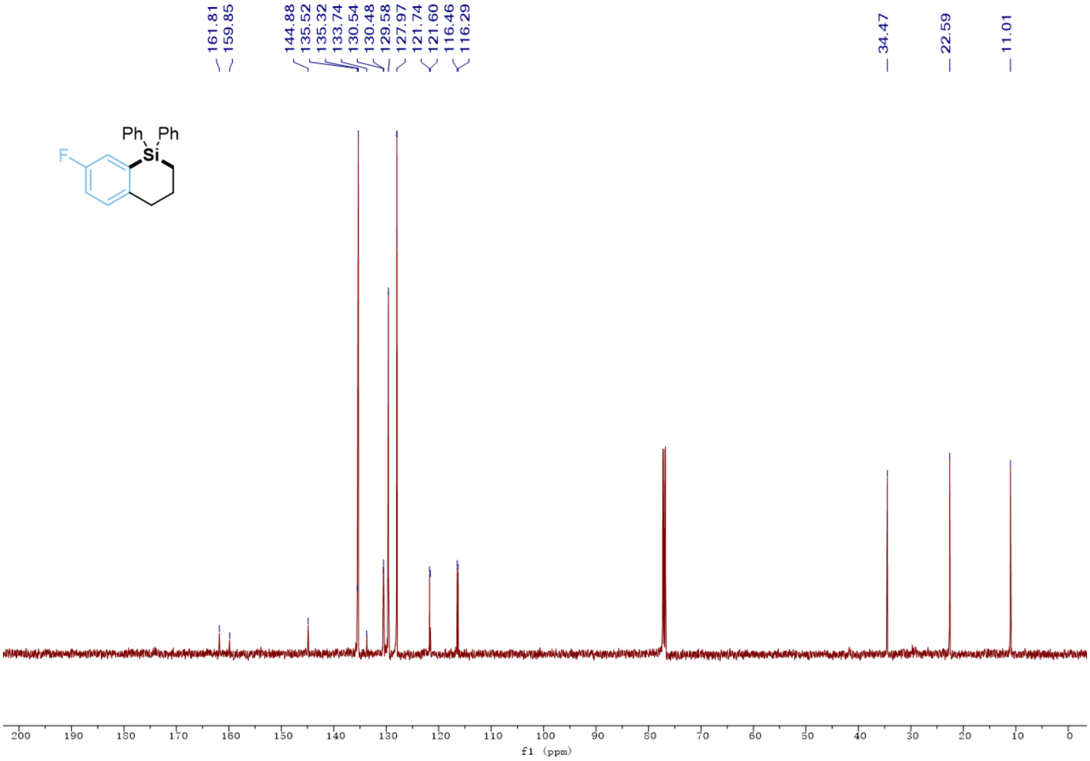

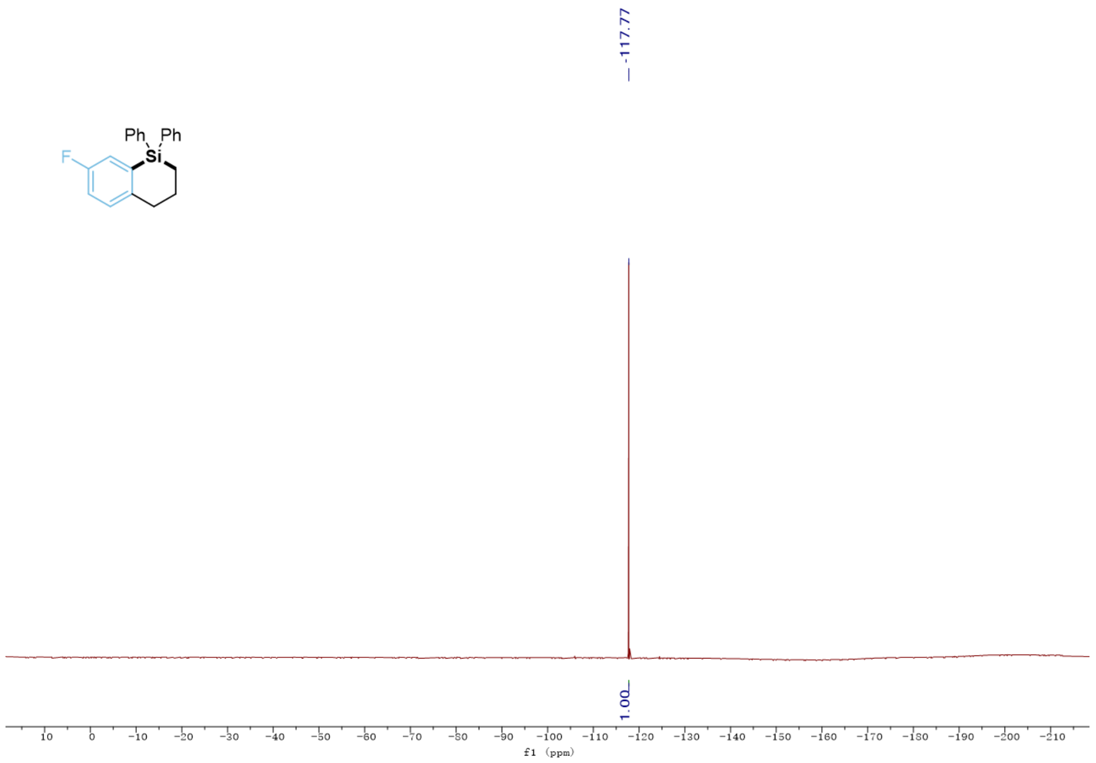

1,1-diphenyl-7-(trifluoromethyl)-1,2,3,4-tetrahydrobenzo[*b*]siline (**6h**)

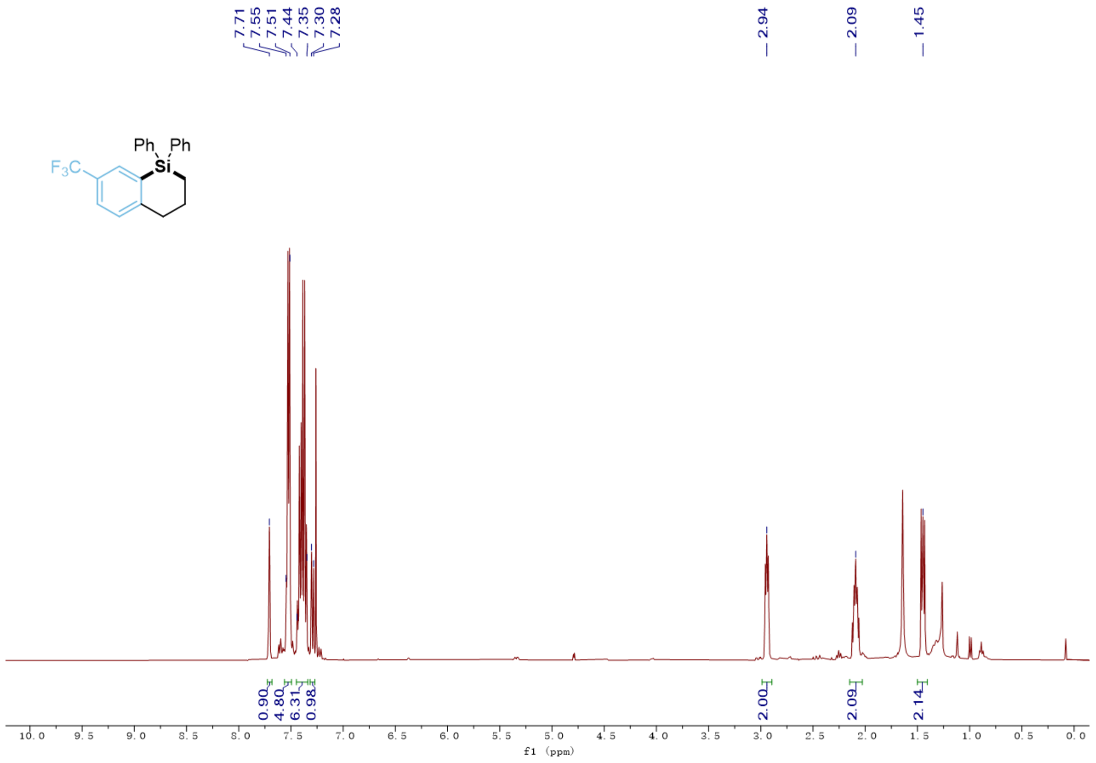

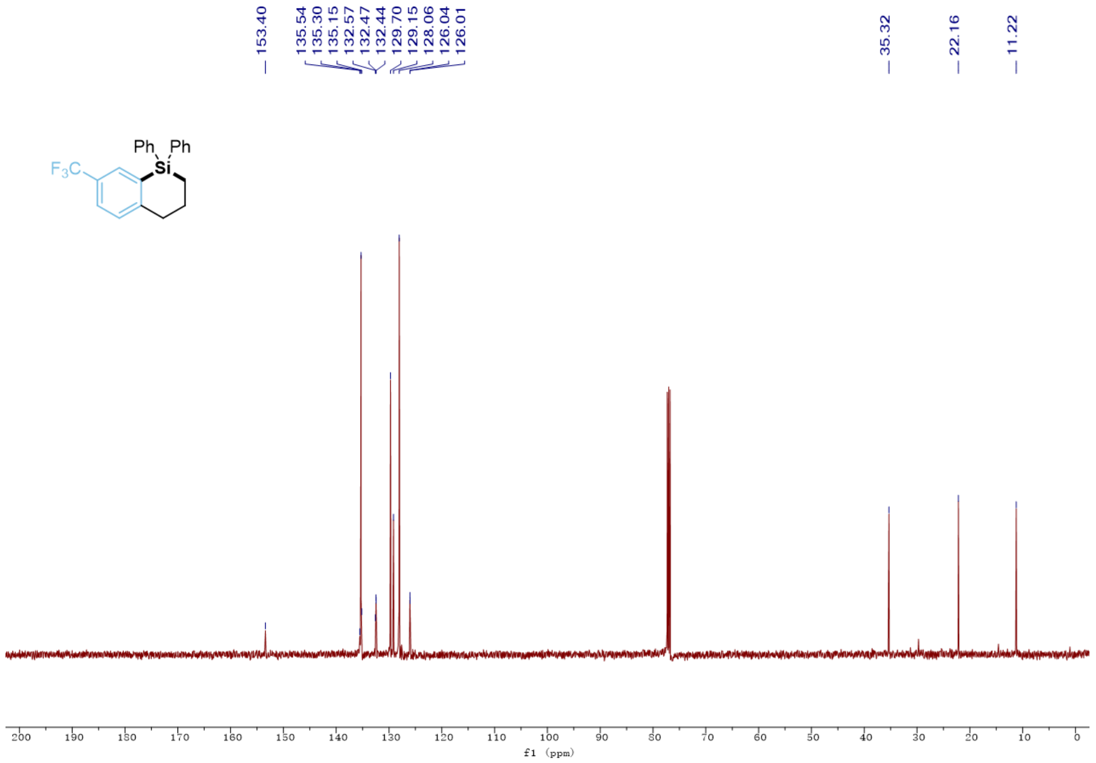
153.40
135.54
135.30
135.15
132.57
132.47
132.44
129.70
129.15
128.06
126.04
126.01
35.32
22.16
11.22
Ph
Ph
Si
F3C
f1 (ppm)

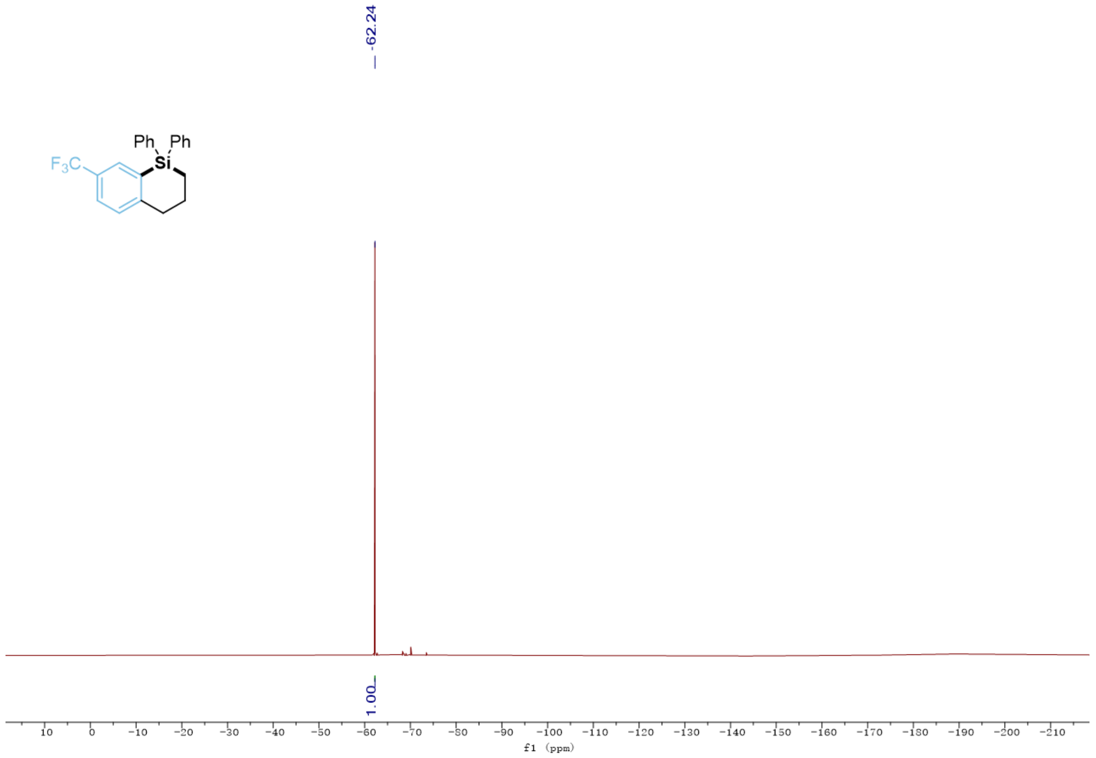
-62.24
Ph
Ph
Si
F3C
1.00
f1 (ppm)

1,1-diphenyl-1,2,3,4-tetrahydrobenzo[*b*]siline-7-carbonitrile (**6i**)

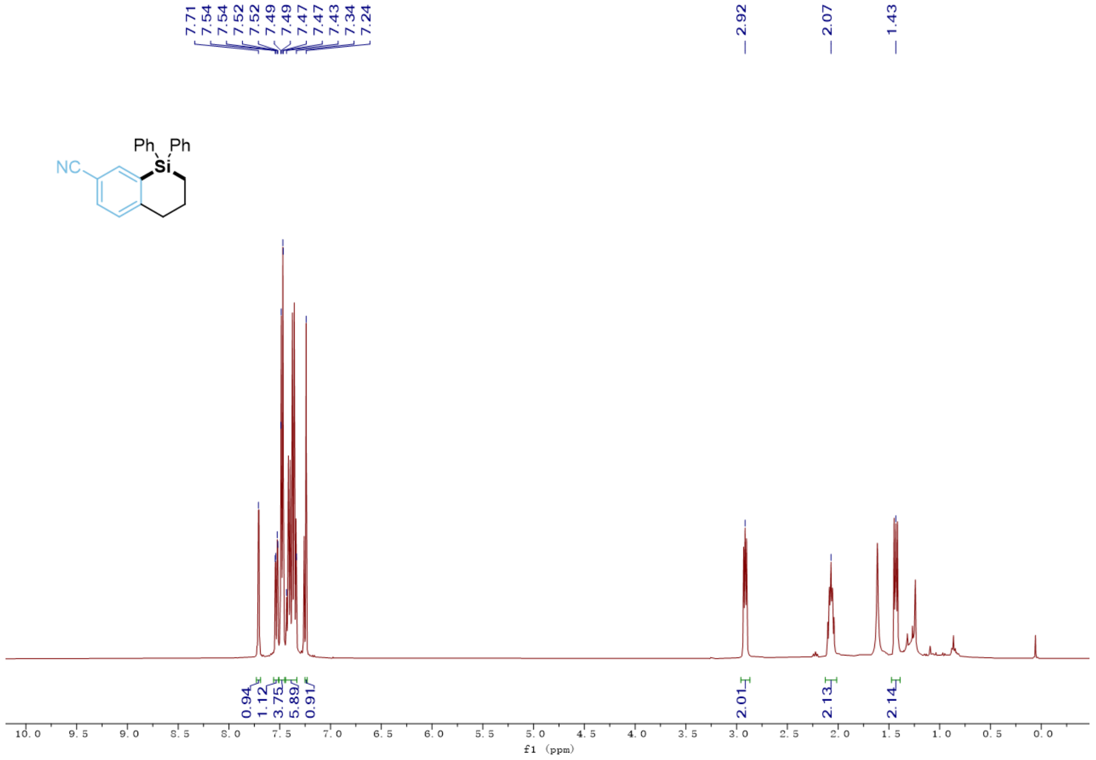

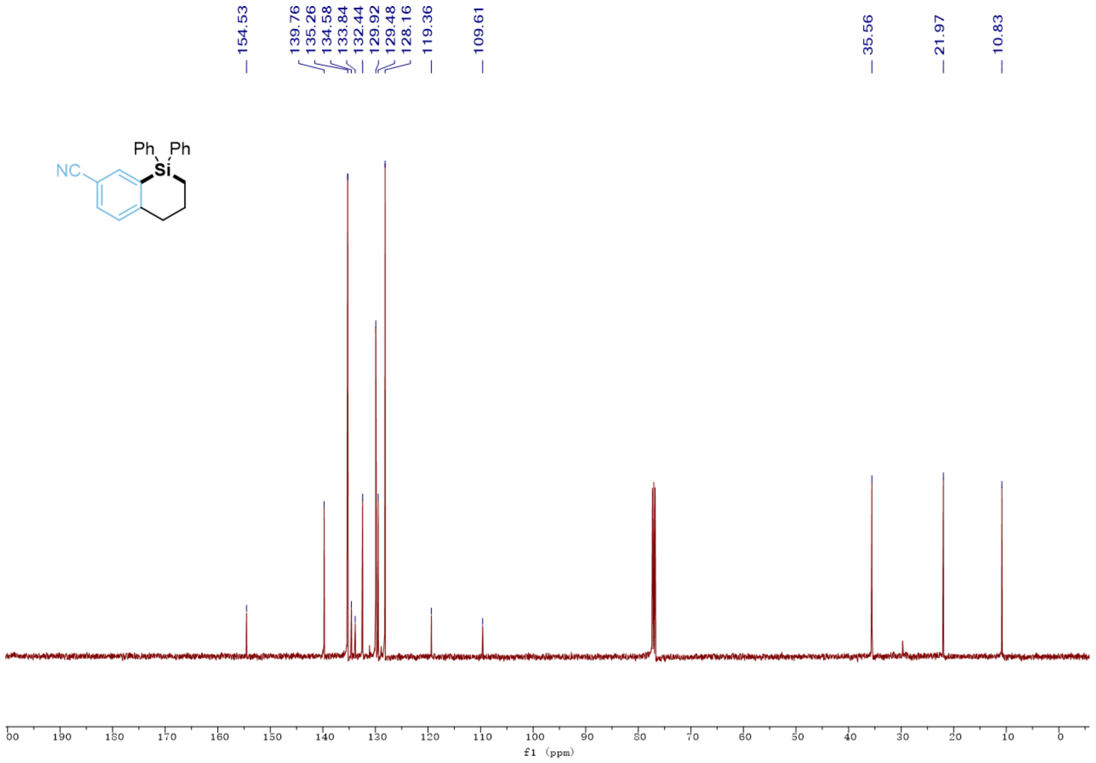

SUPPORTING INFORMATION

Methyl 1,1-diphenyl-1,2,3,4-tetrahydrobenzo[*b*]siline-7-carboxylate (**6j**)

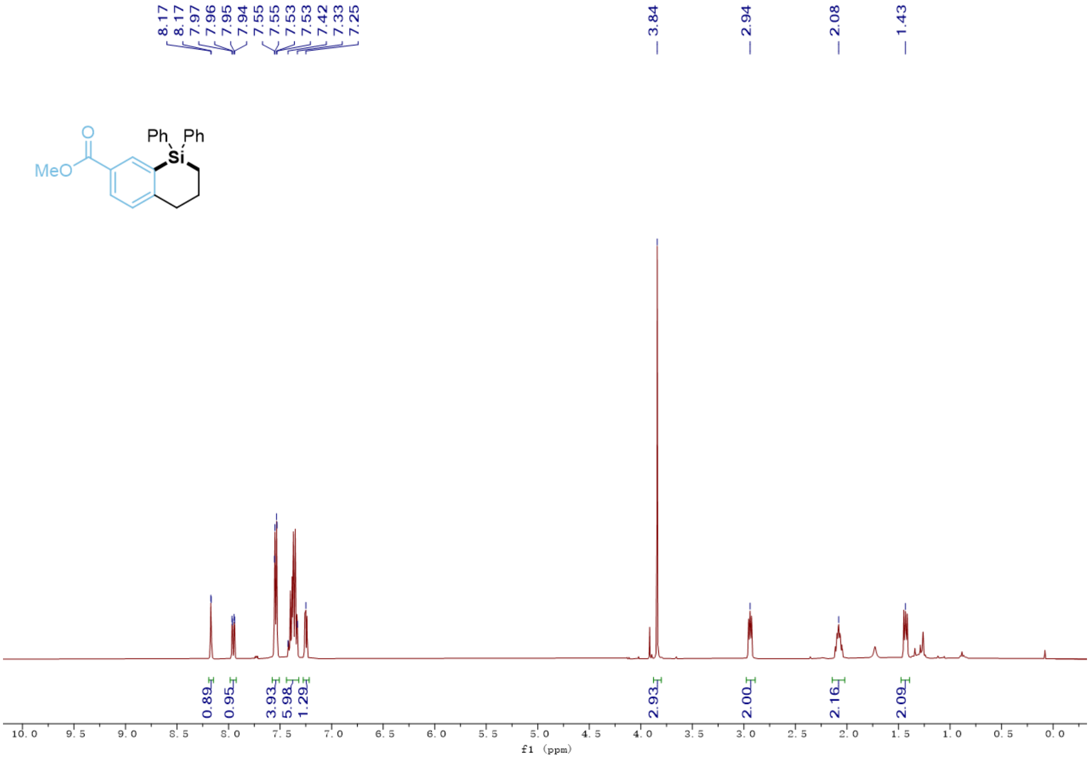

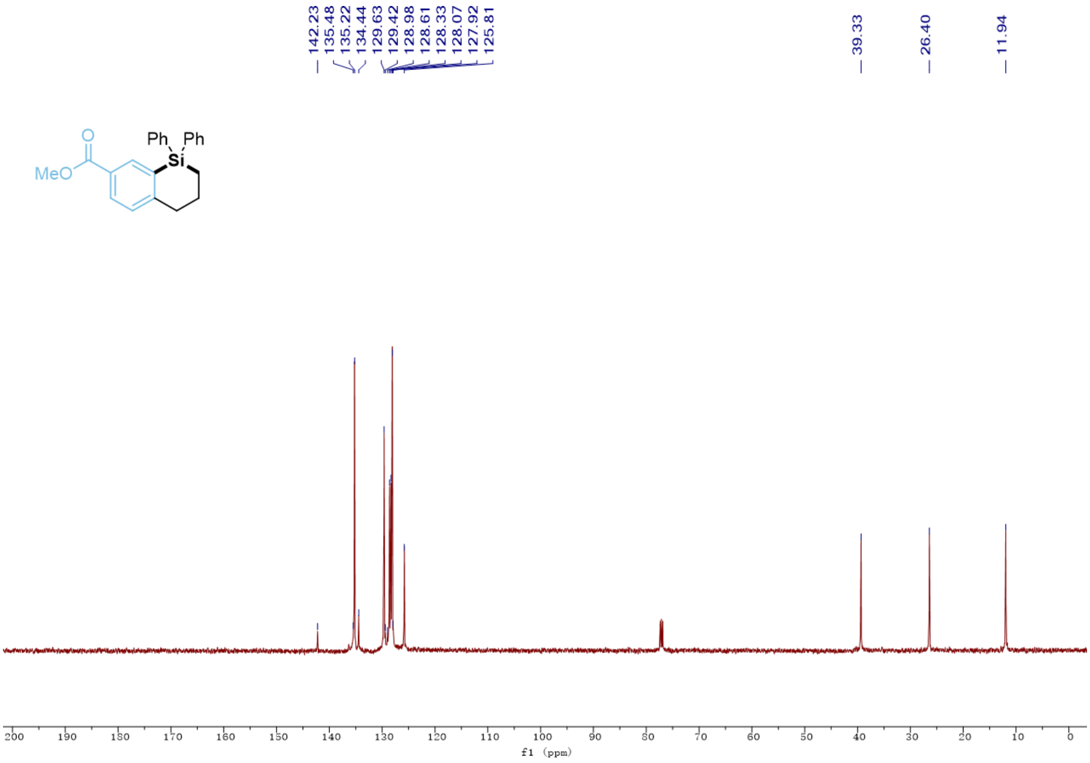

SUPPORTING INFORMATION

tert-Butyl 1,1-diphenyl-1,2,3,4-tetrahydrobenzo[*b*]siline-7-carboxylate (**6k**)

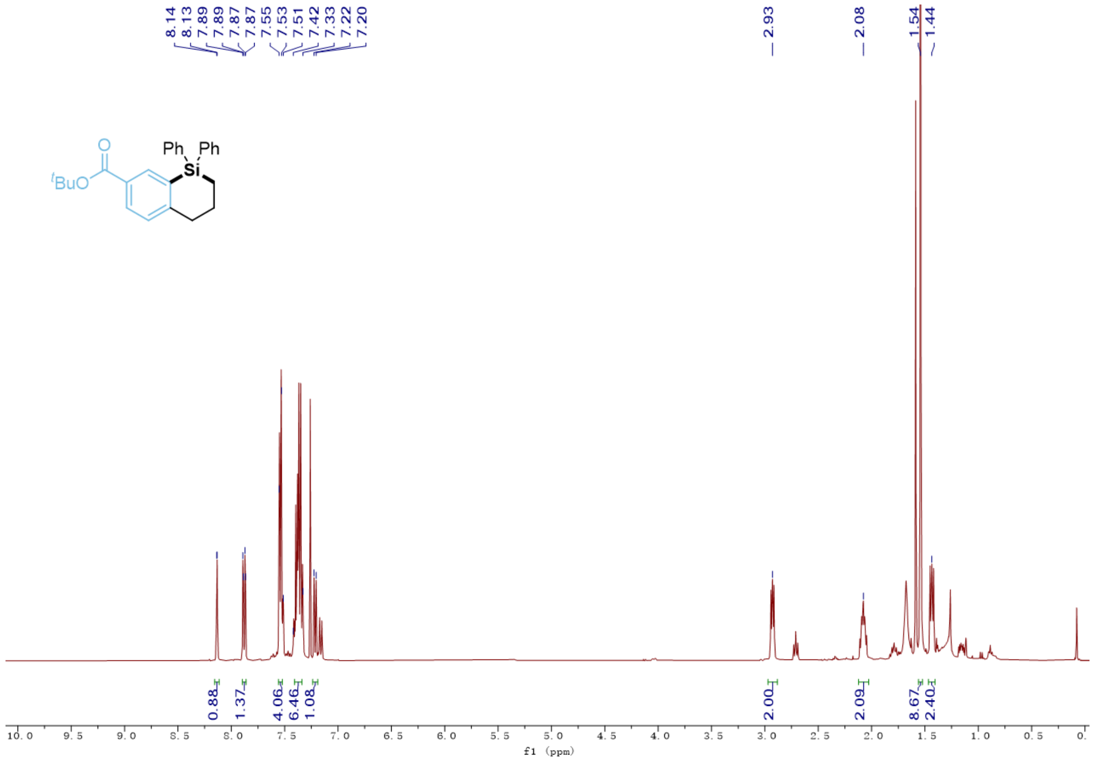

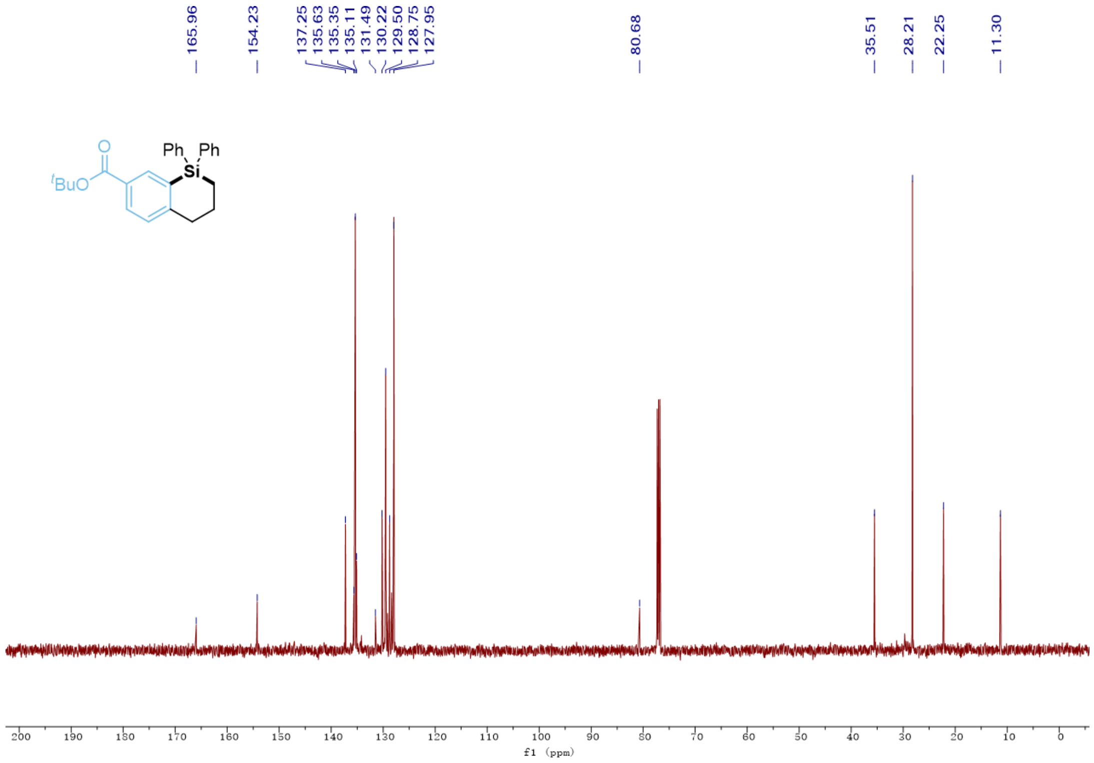

SUPPORTING INFORMATION

6,8-dimethyl-1,1-diphenyl-1,2,3,4-tetrahydrobenzo[*b*]siline (**6l**)

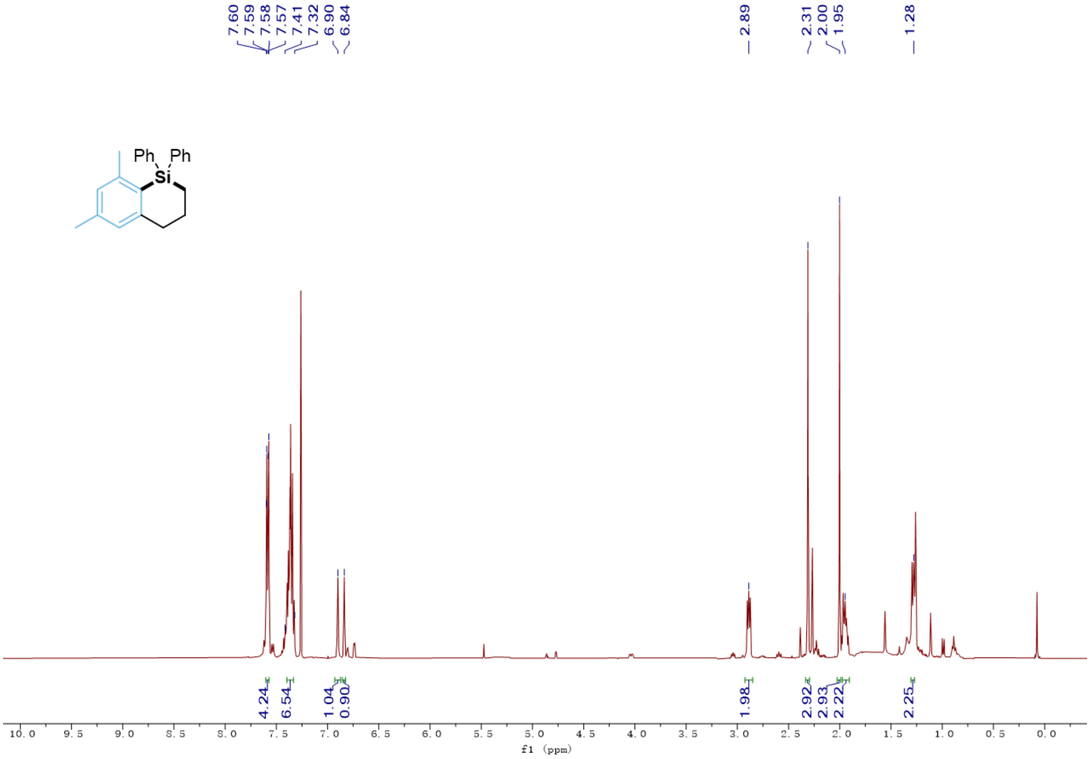

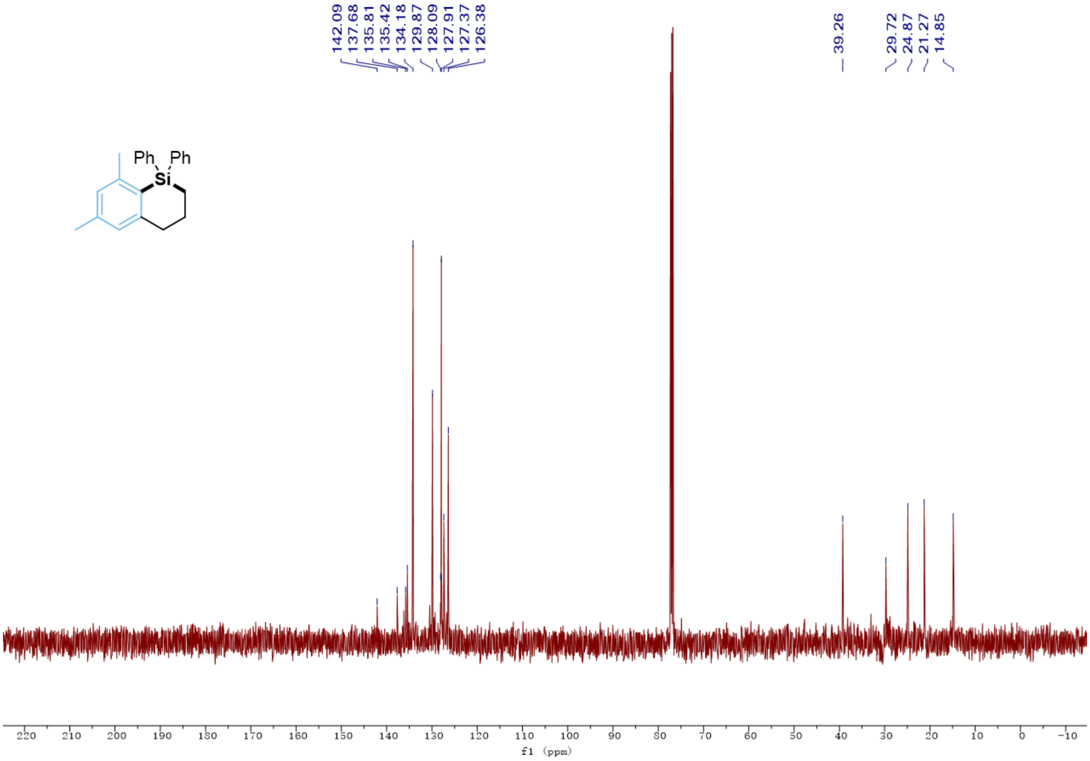

SUPPORTING INFORMATION

6,8-di-tert-butyl-1,1-diphenyl-1,2,3,4-tetrahydrobenzo[*b*]siline (**6m**)

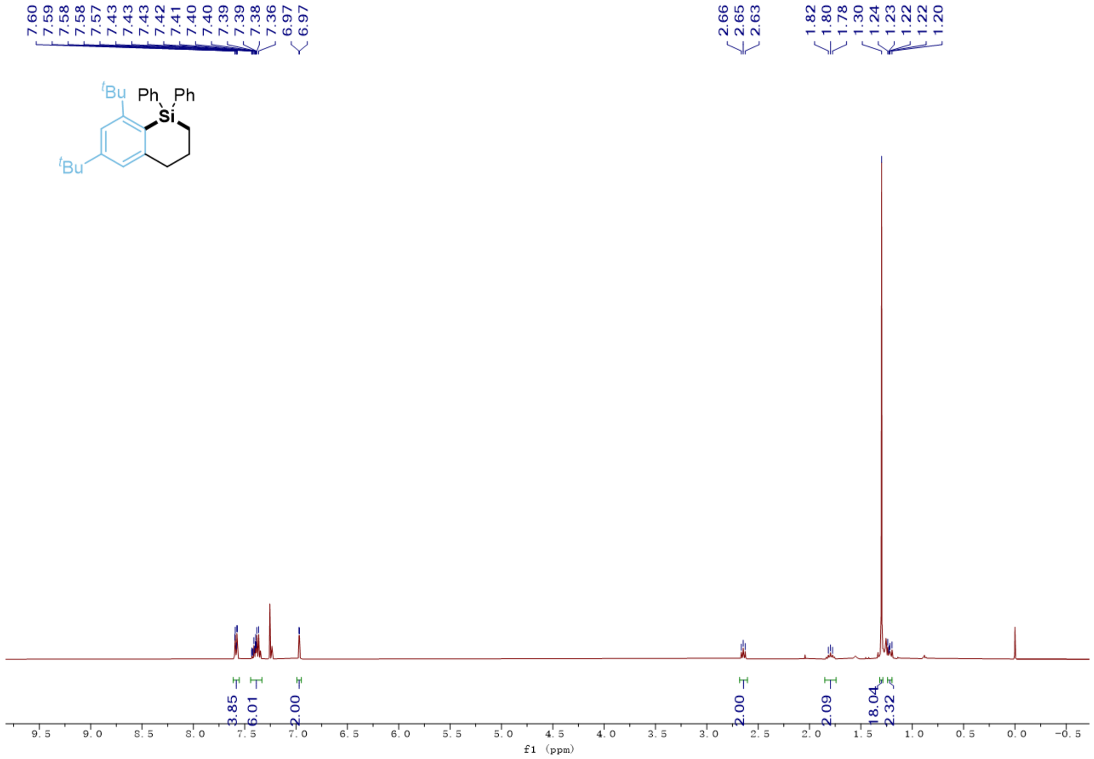

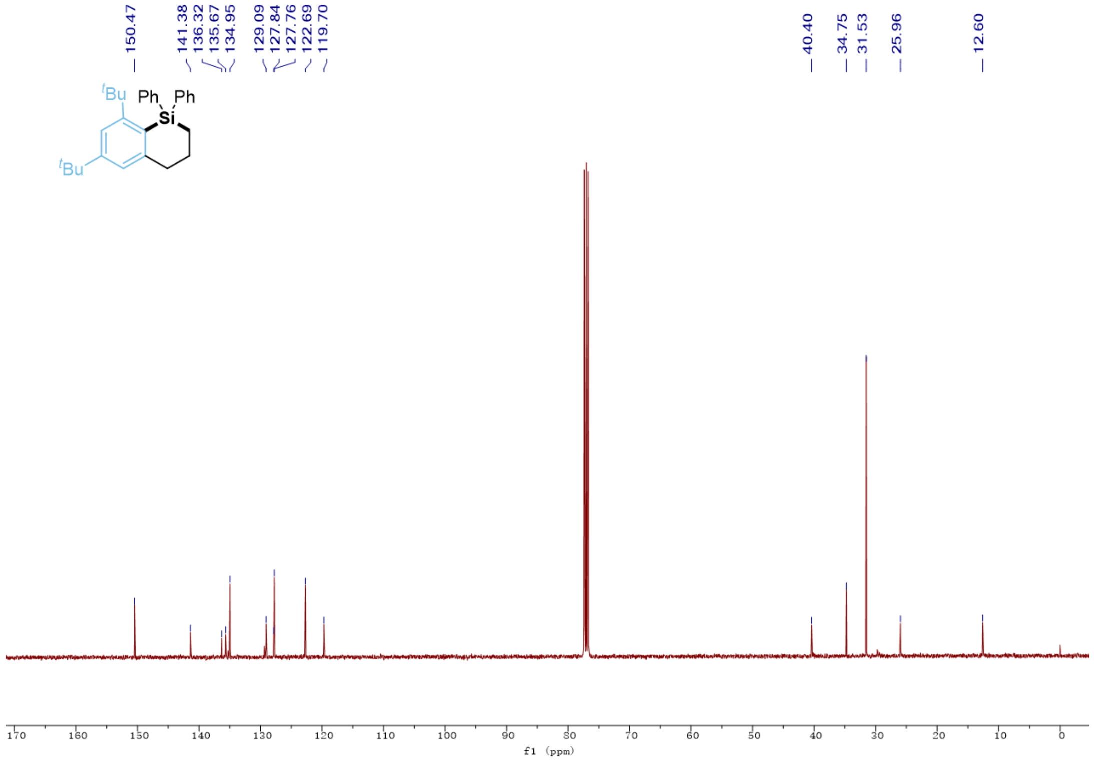

SUPPORTING INFORMATION

6,7-dimethoxy-1,1-diphenyl-1,2,3,4-tetrahydrobenzo[*b*]siline (**6n**)

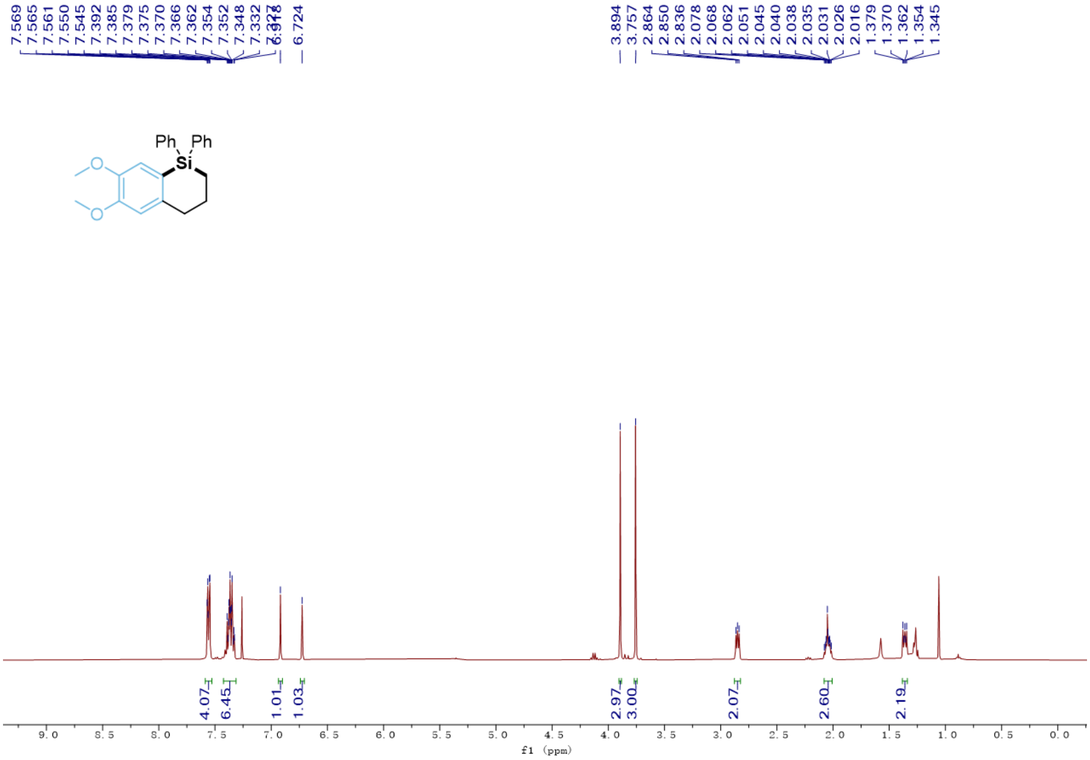

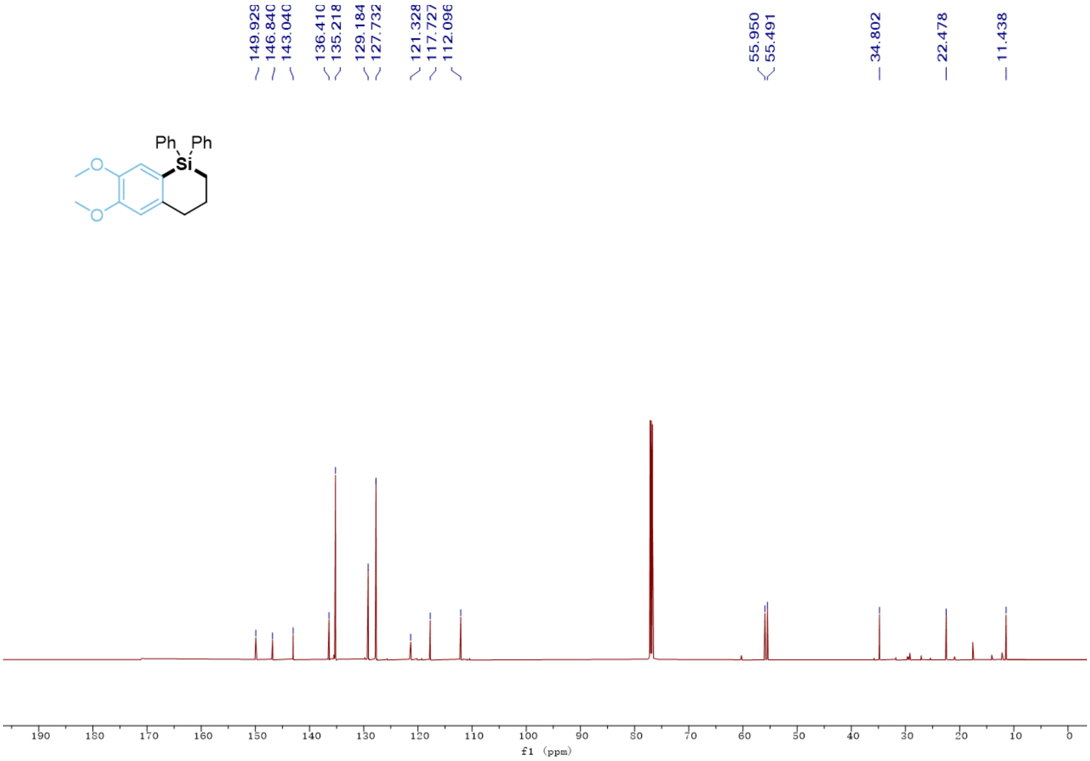

7,8-dimethoxy-1,1-diphenyl-1,2,3,4-tetrahydrobenzo[*b*]siline (**6n′**)

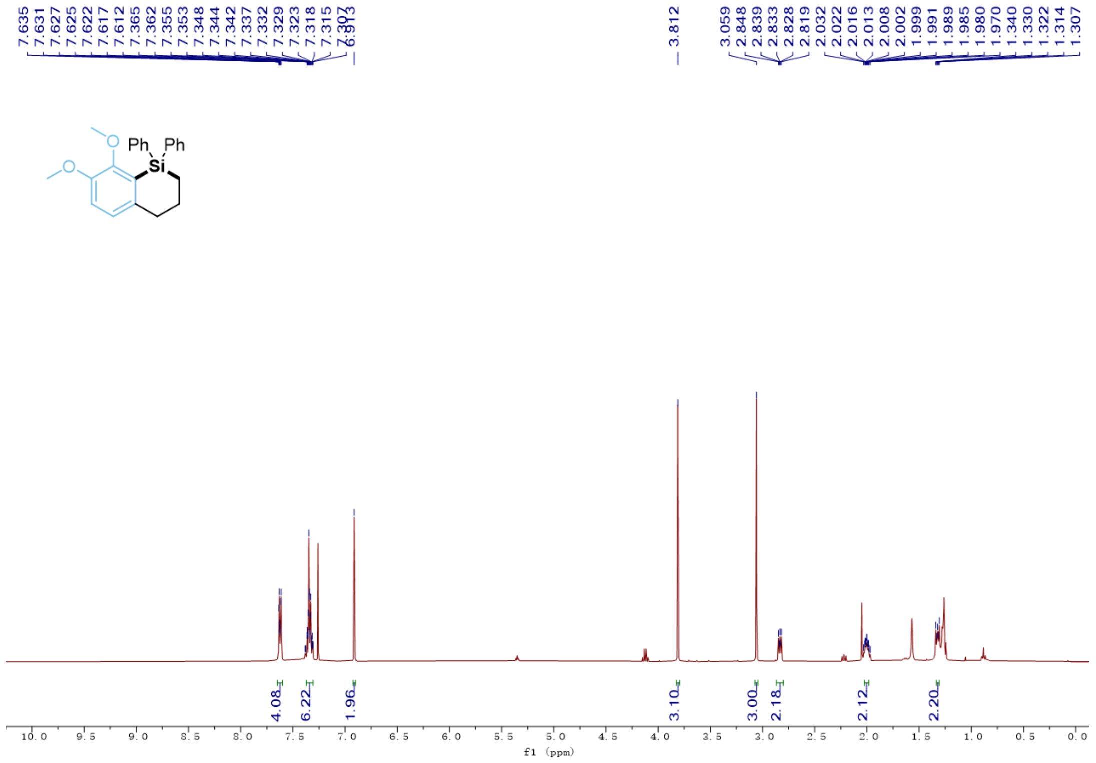

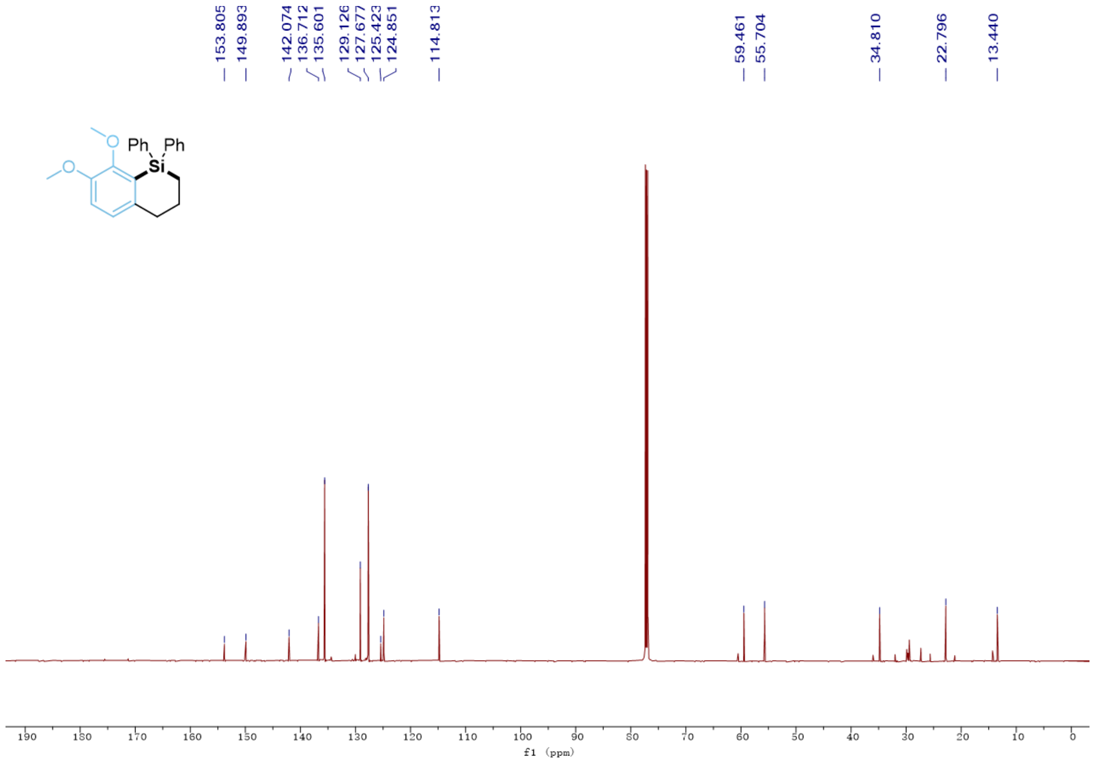

SUPPORTING INFORMATION

6,7-dichloro-1,1-diphenyl-1,2,3,4-tetrahydrobenzo[*b*]siline (**6o**)

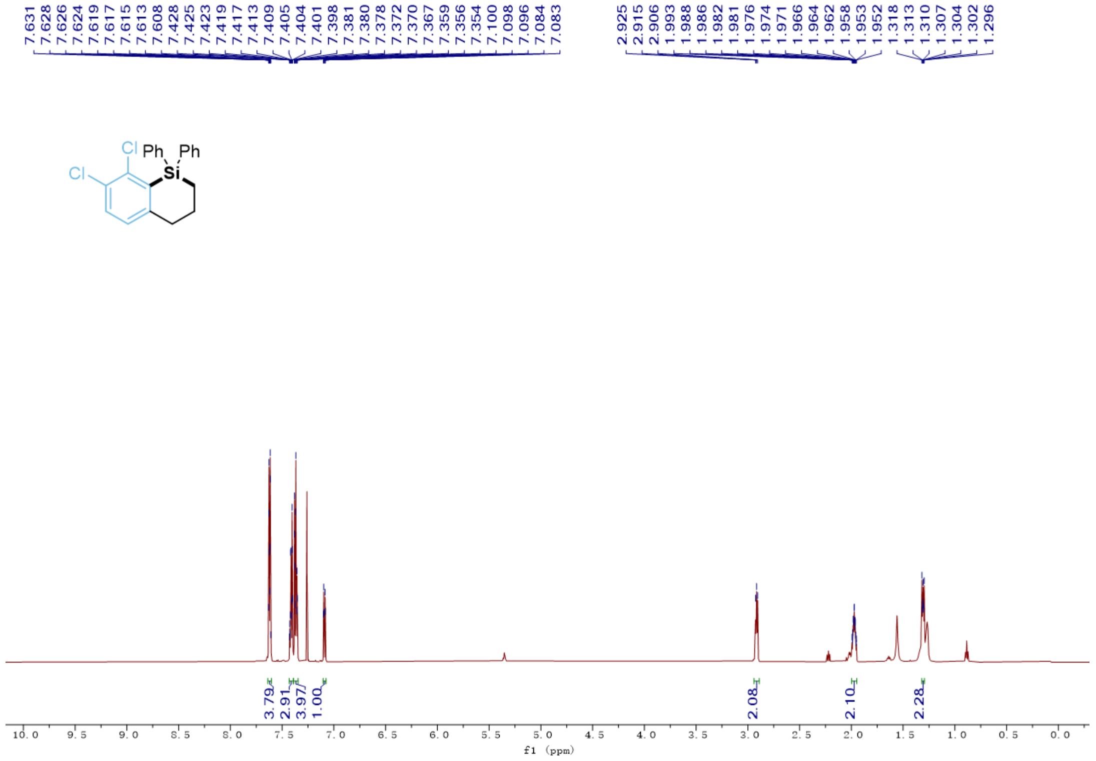

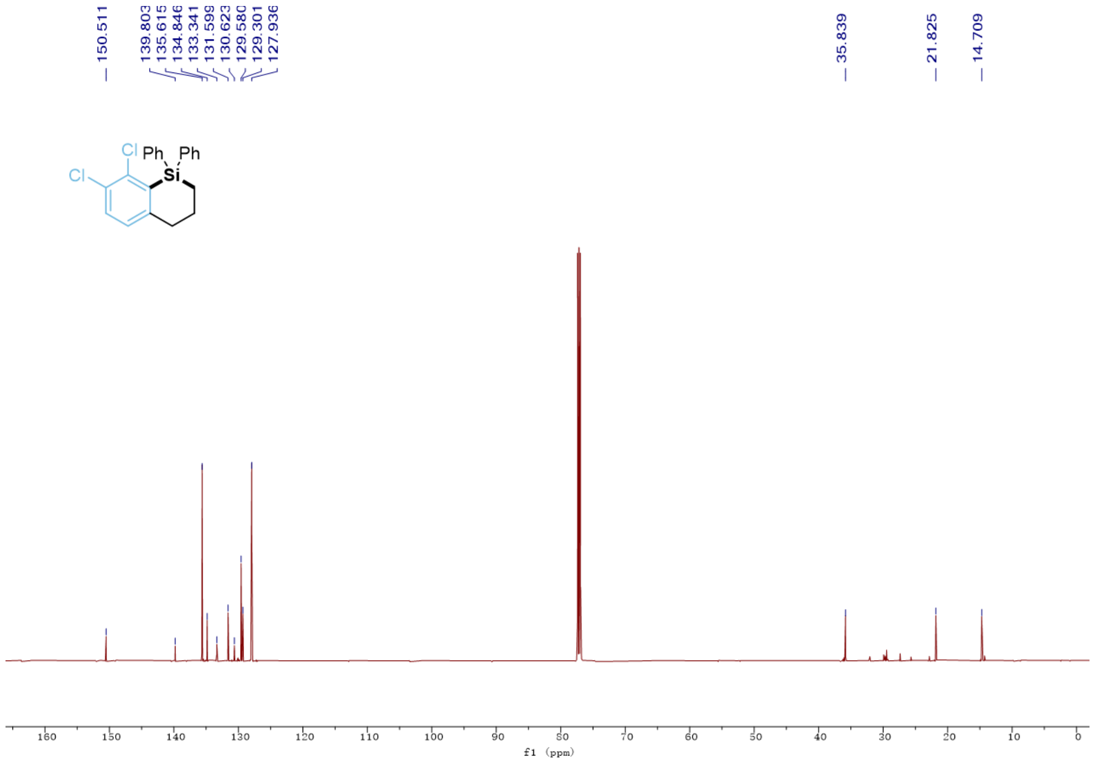

SUPPORTING INFORMATION

1,1-diphenyl-6,7-bis(trifluoromethyl)-1,2,3,4-tetrahydrobenzo[*b*]siline (**6p**)

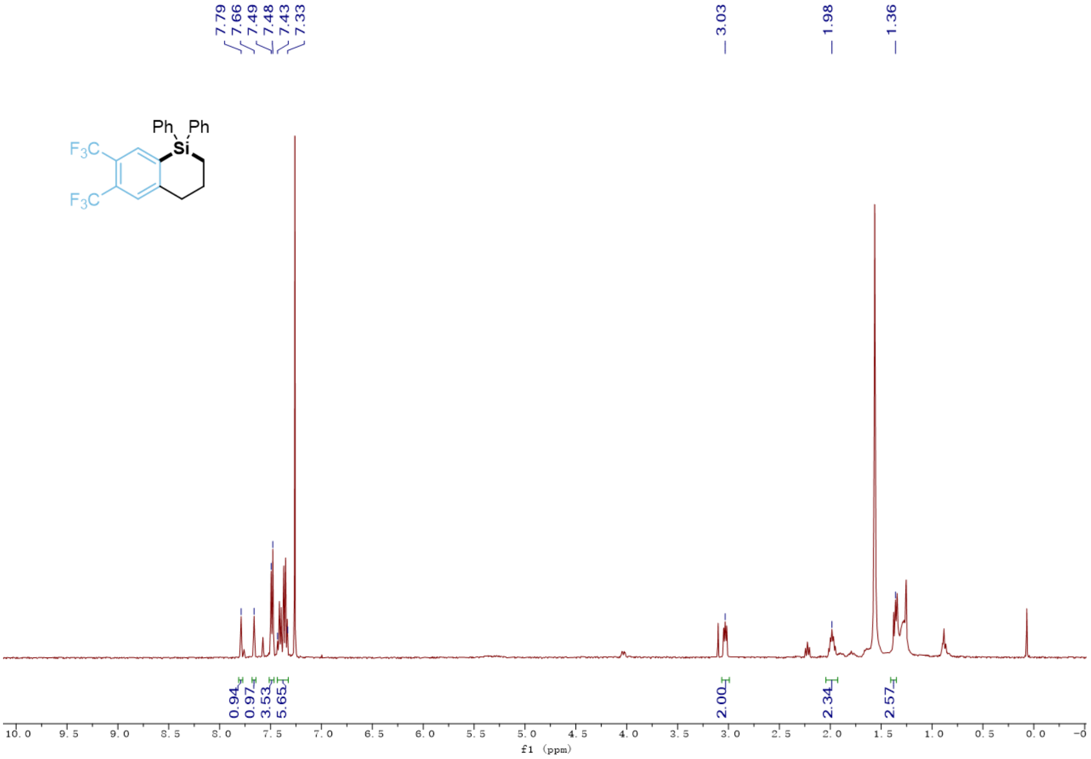

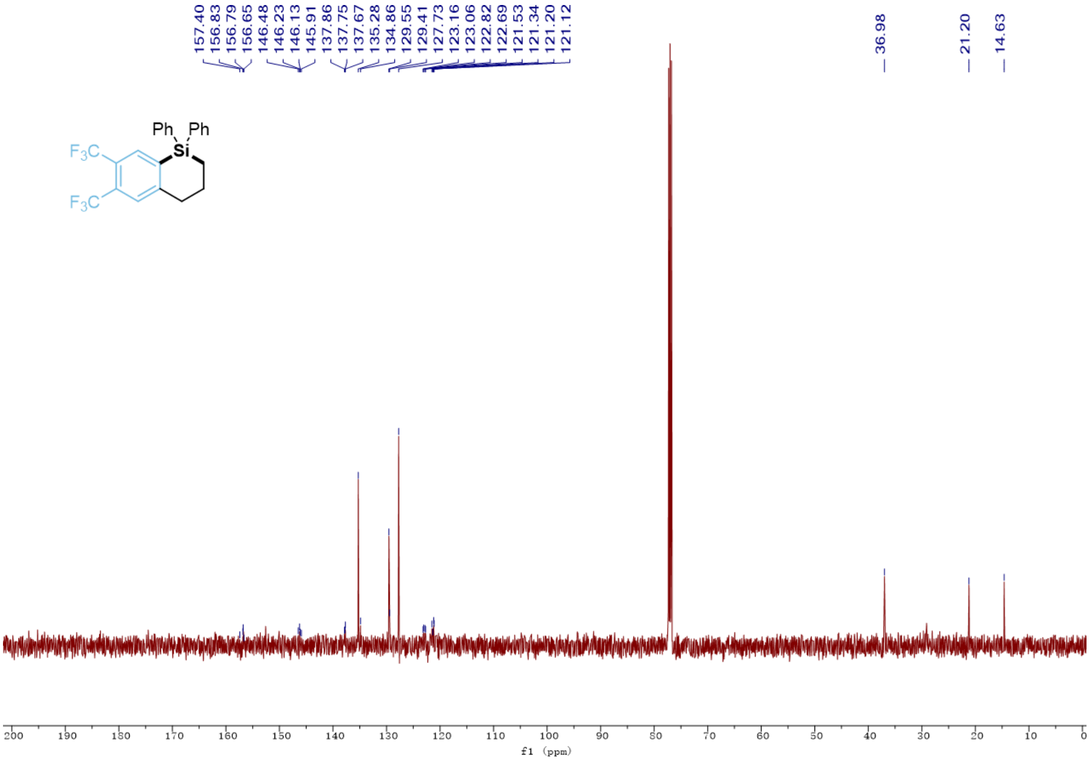

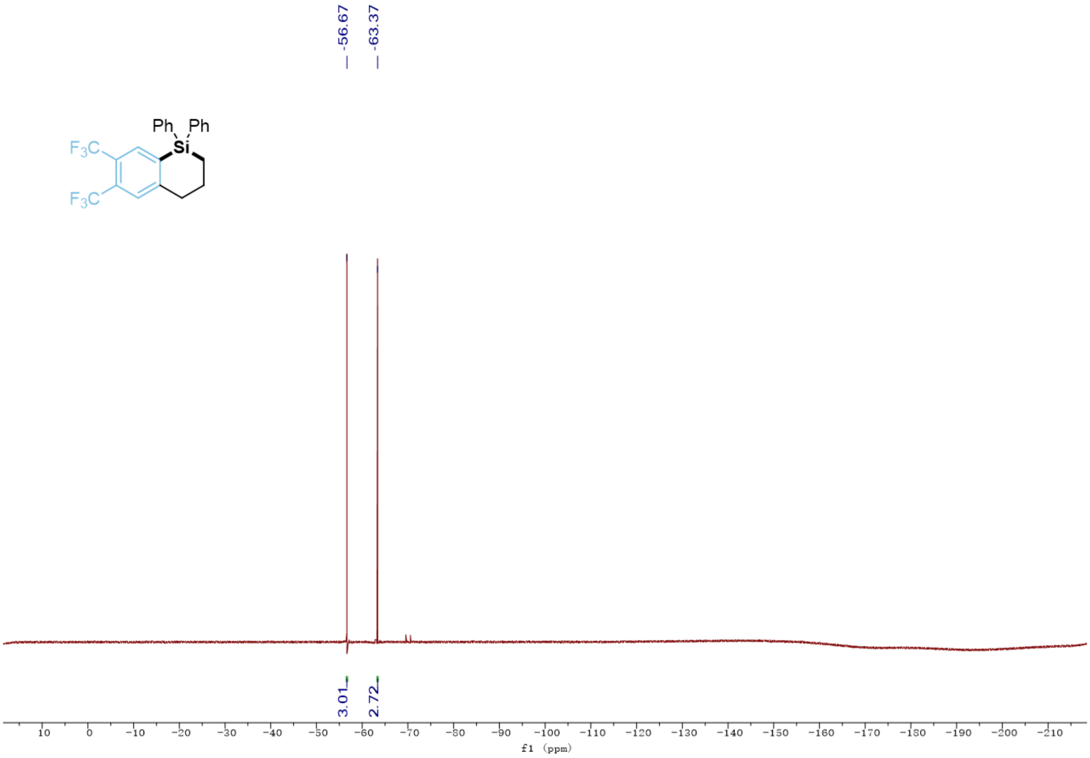

4,4-diphenyl-1,2,3,4-tetrahydronaphtho[2,1-*b*]siline (**6q**)

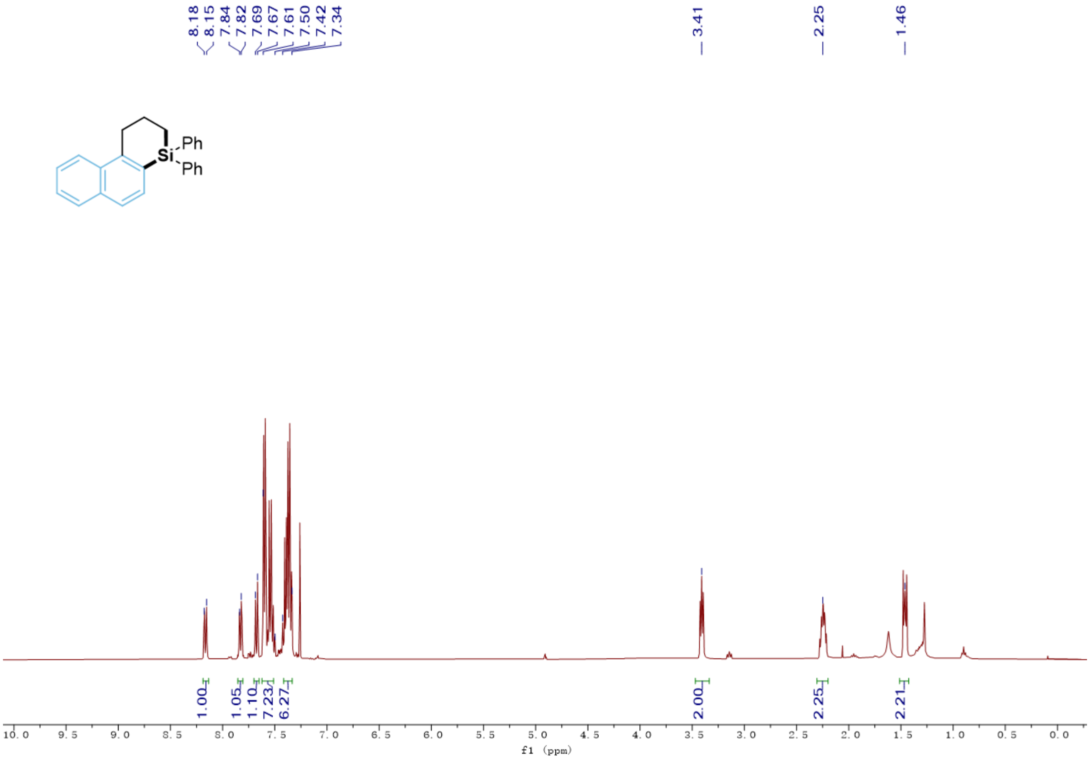

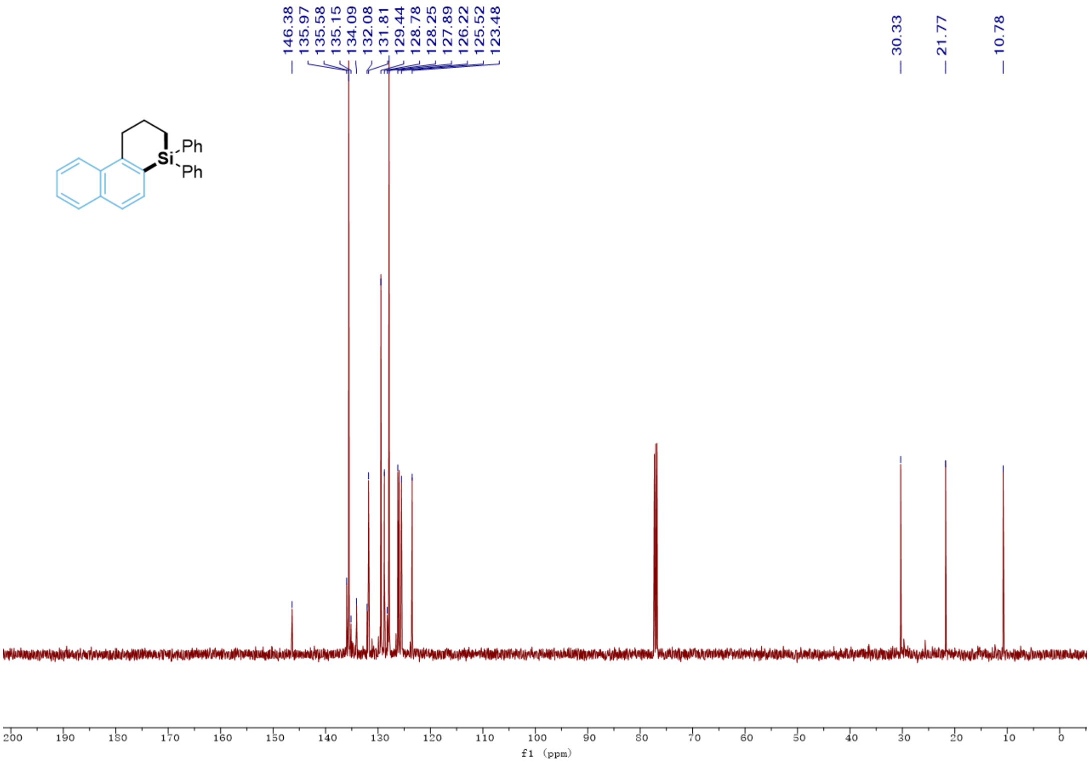

1,1-diphenyl-1,2,3,4-tetrahydronaphtho[2,3-*b*]siline (**6r**)

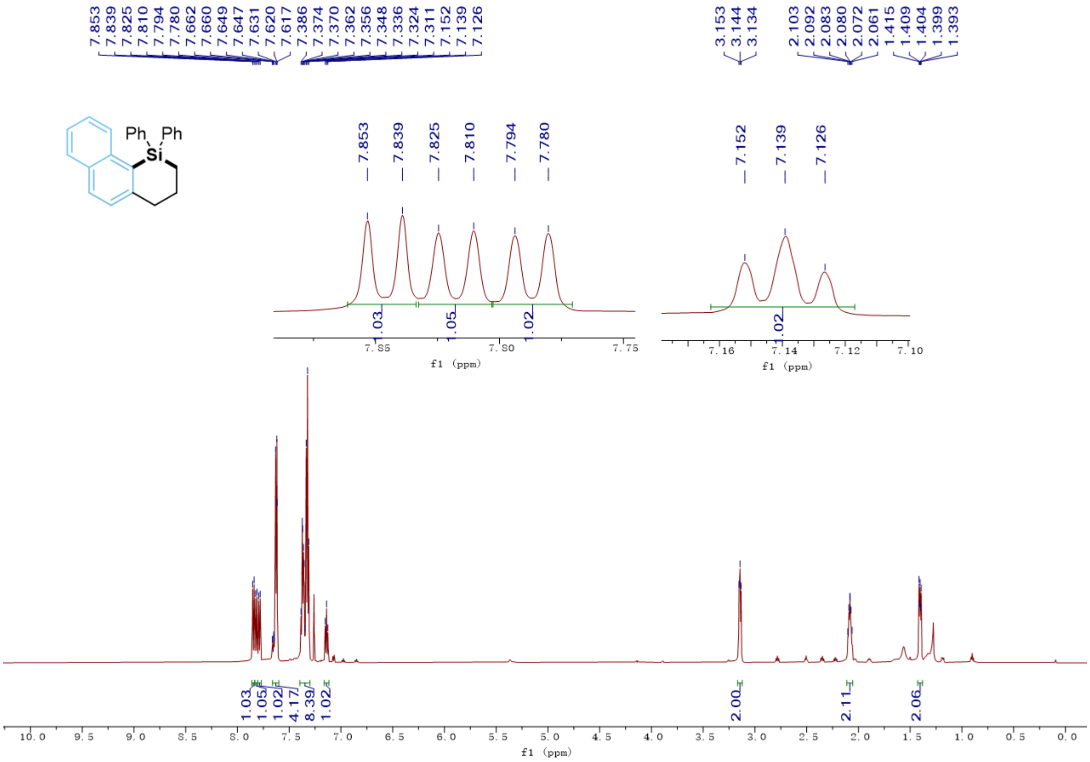

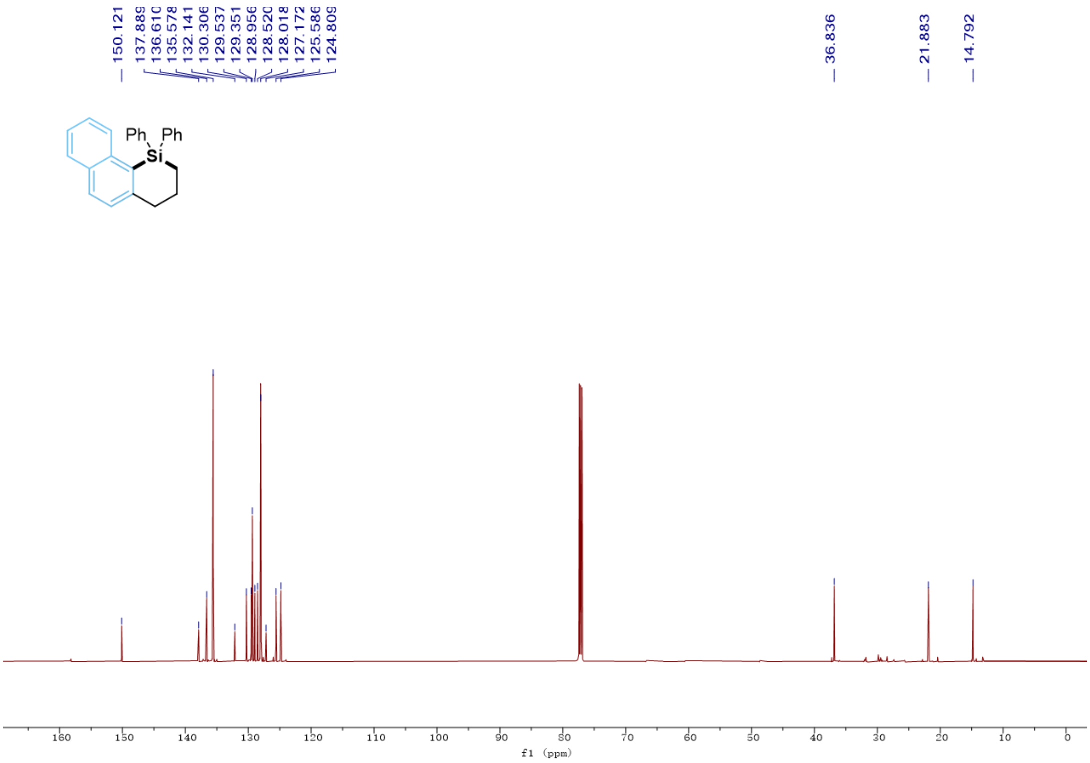

1,1,1',1'-tetraphenyl-1,1',2,2',3,3',4,4'-octahydro-7,7'-bibenzo[*b*]siline (**6s**)

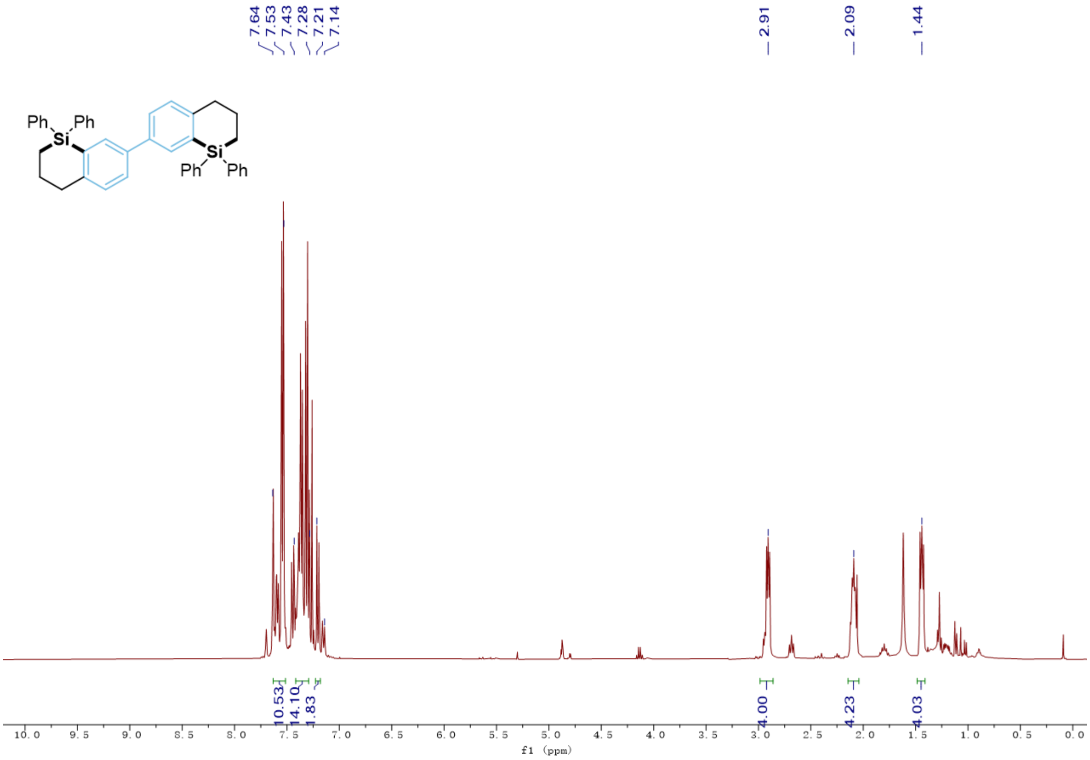

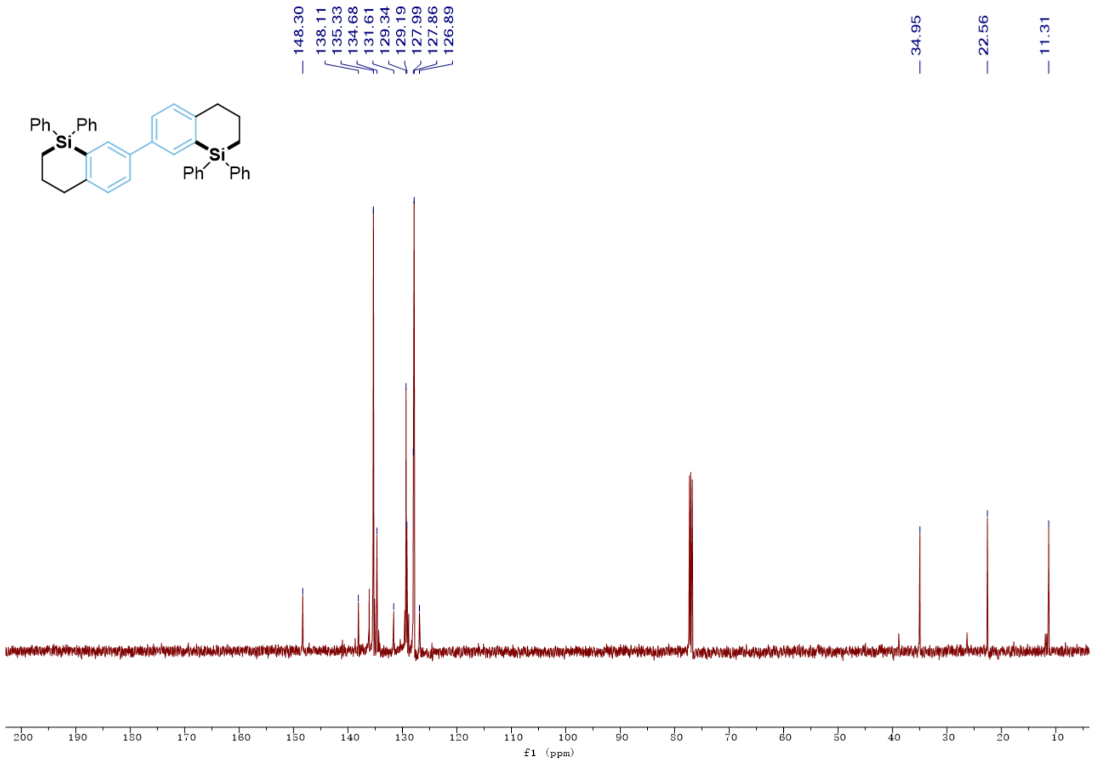

5,6,7-trifluoro-1,1-diphenyl-1,2,3,4-tetrahydrobenzo[*b*]siline (**6t**)

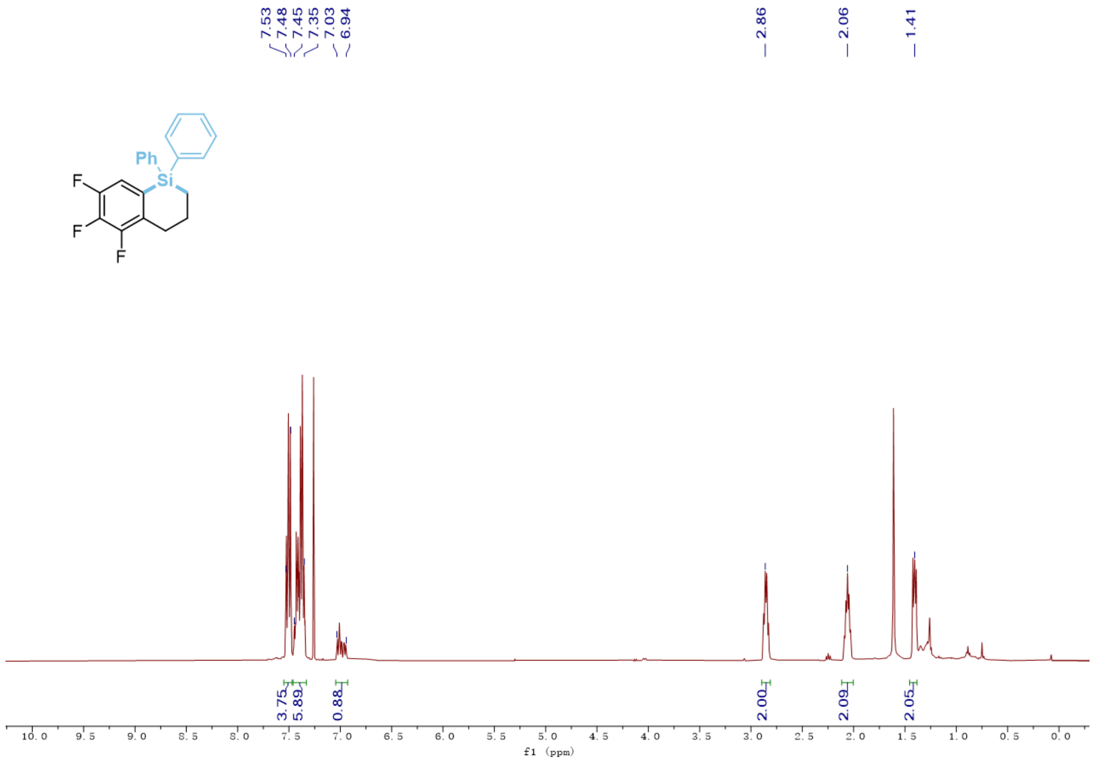

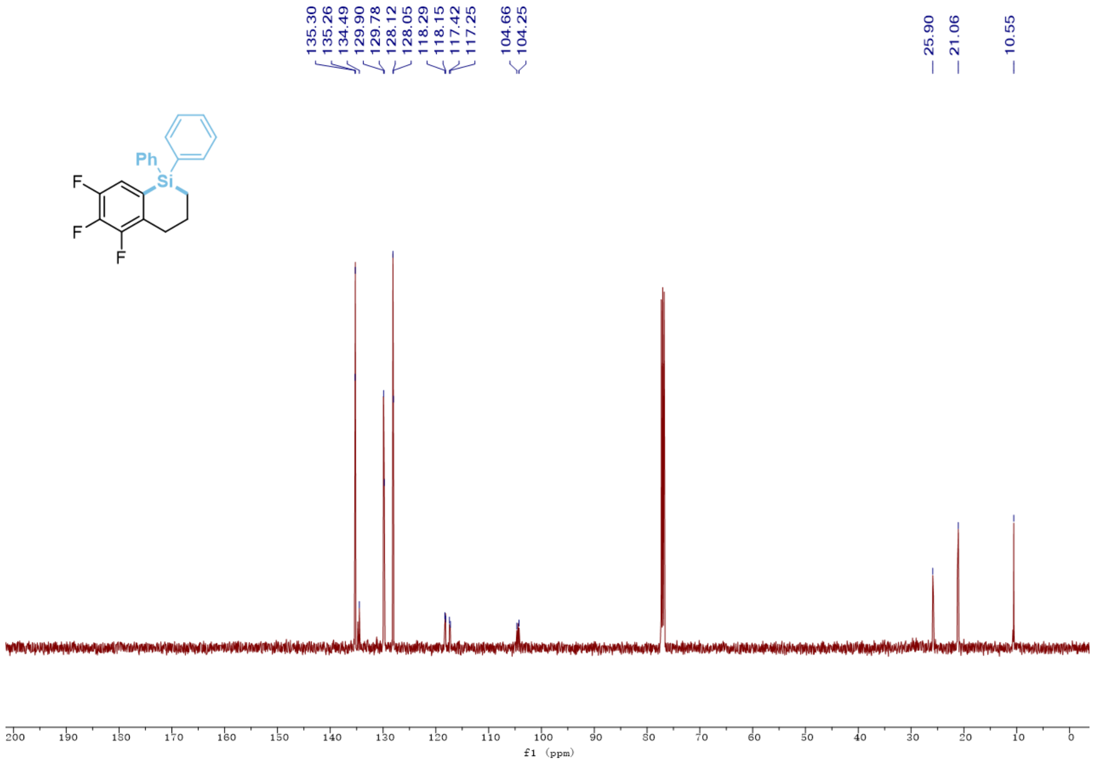
135.30
135.26
134.49
129.90
129.78
128.12
128.05
118.29
118.15
117.42
117.25
104.66
104.25
25.90
21.06
10.55
Ph
Si
F
F
F
f1 (ppm)

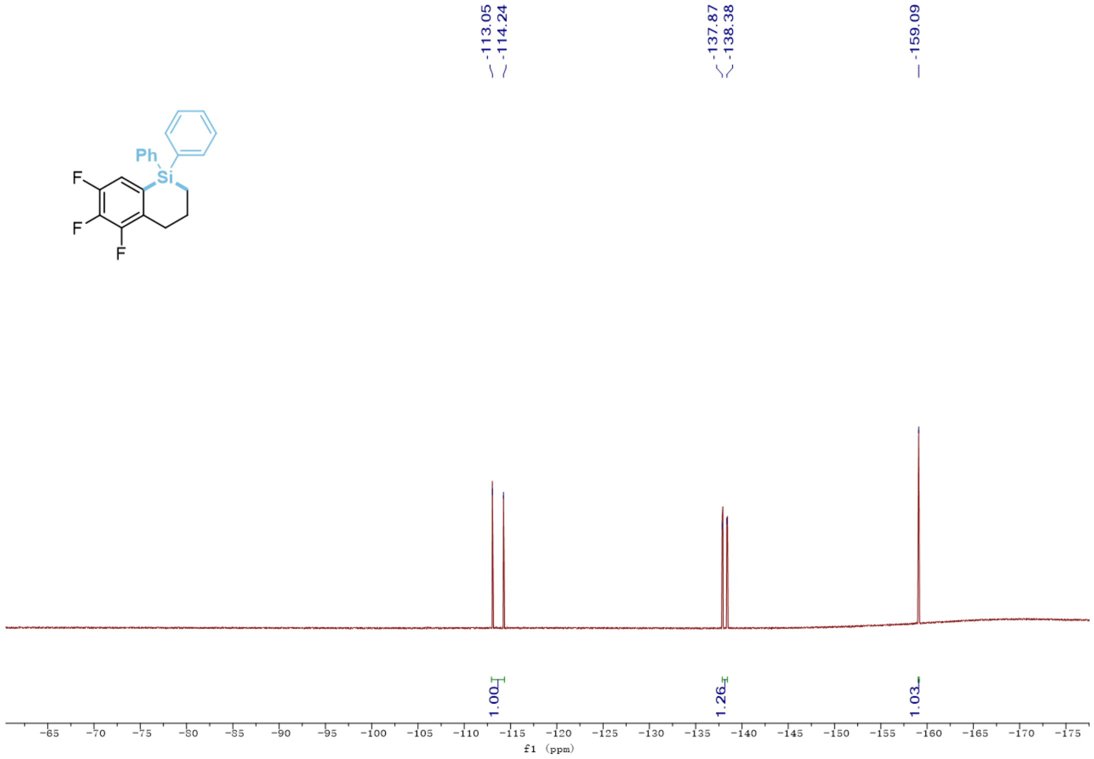
-113.05
-114.24
-137.87
-138.38
-159.09
Ph
Si
F
F
F
1.00
1.26
1.03
f1 (ppm)

SUPPORTING INFORMATION

1-phenyl-1-(p-tolyl)-1,2,3,4-tetrahydrobenzo[*b*]siline (**7a**)

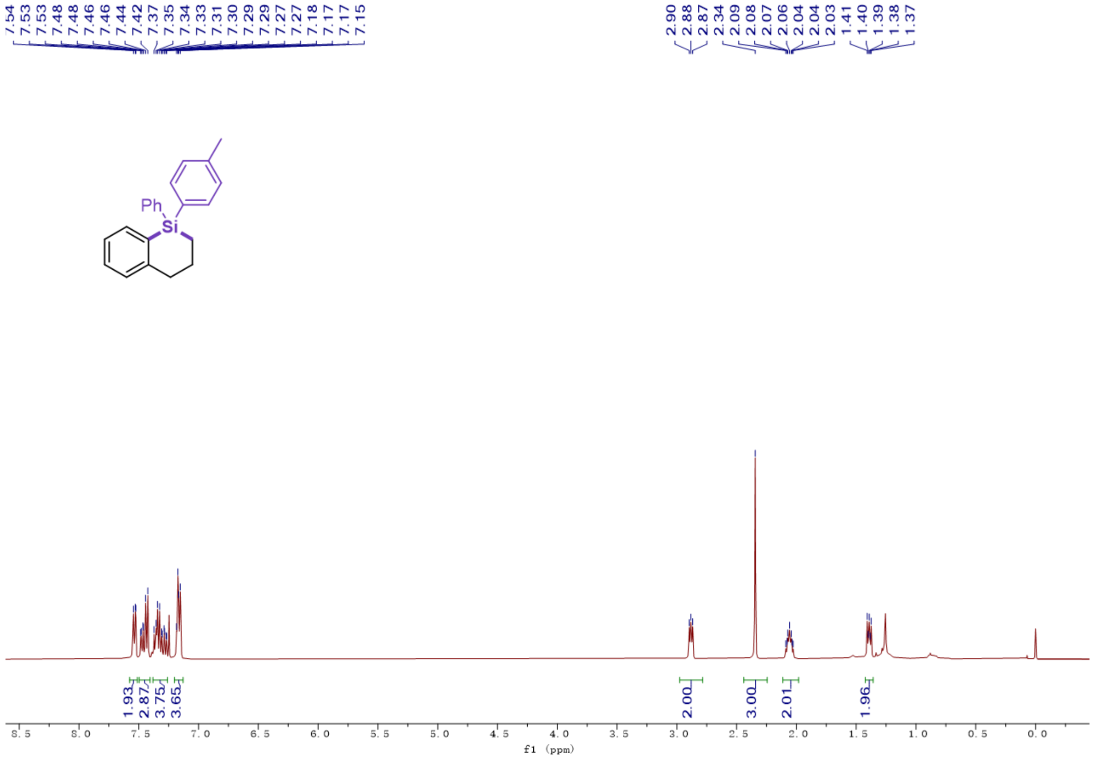

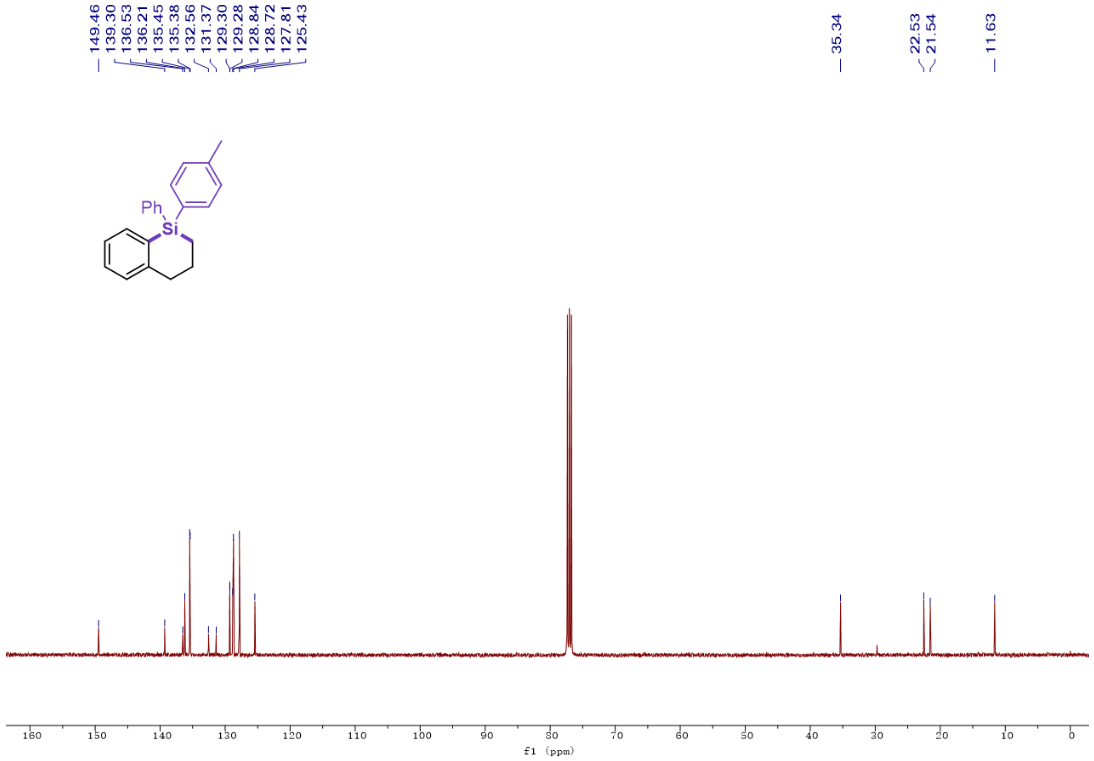

1-(4-fluorophenyl)-1-phenyl-1,2,3,4-tetrahydrobenzo[*b*]siline (**7b**)

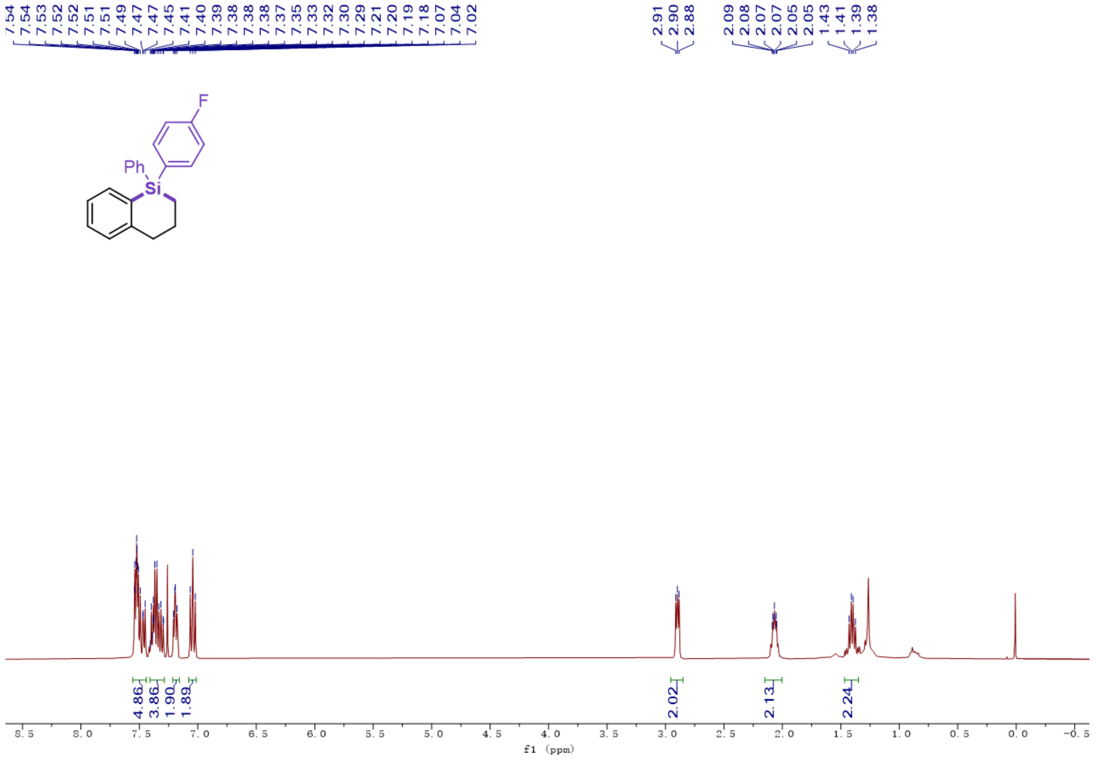

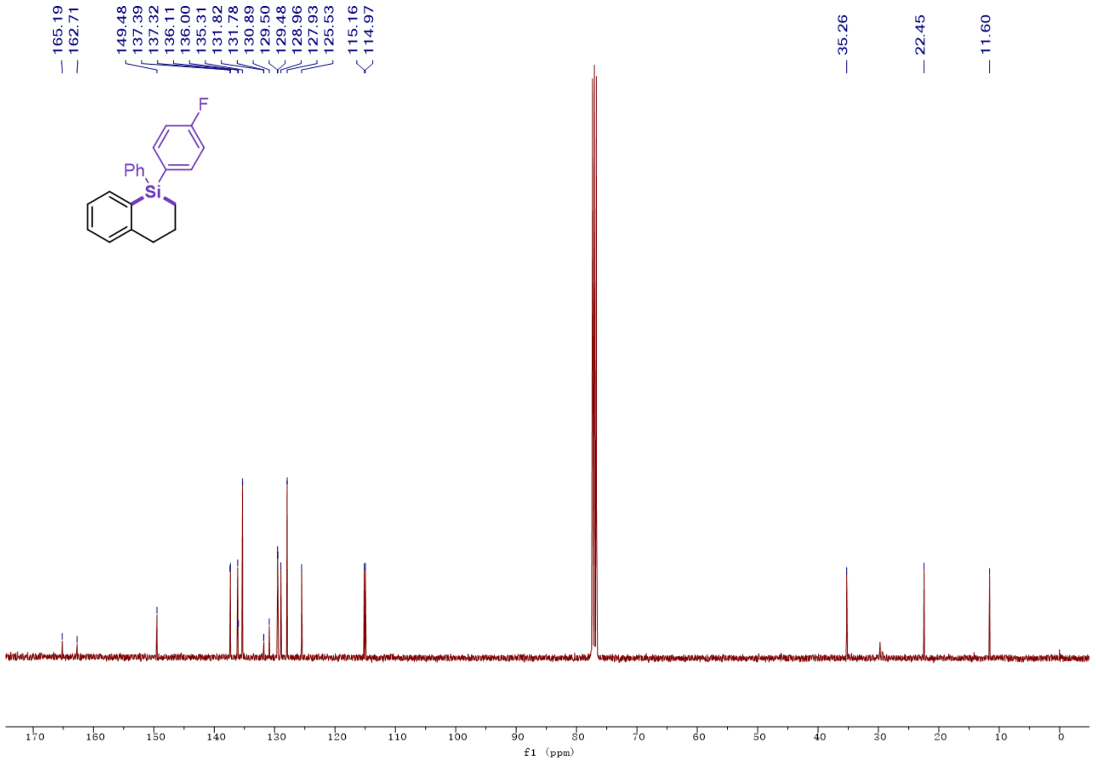

SUPPORTING INFORMATION

1-(4-methoxyphenyl)-1-phenyl-1,2,3,4-tetrahydrobenzo[*b*]siline (**7c**)

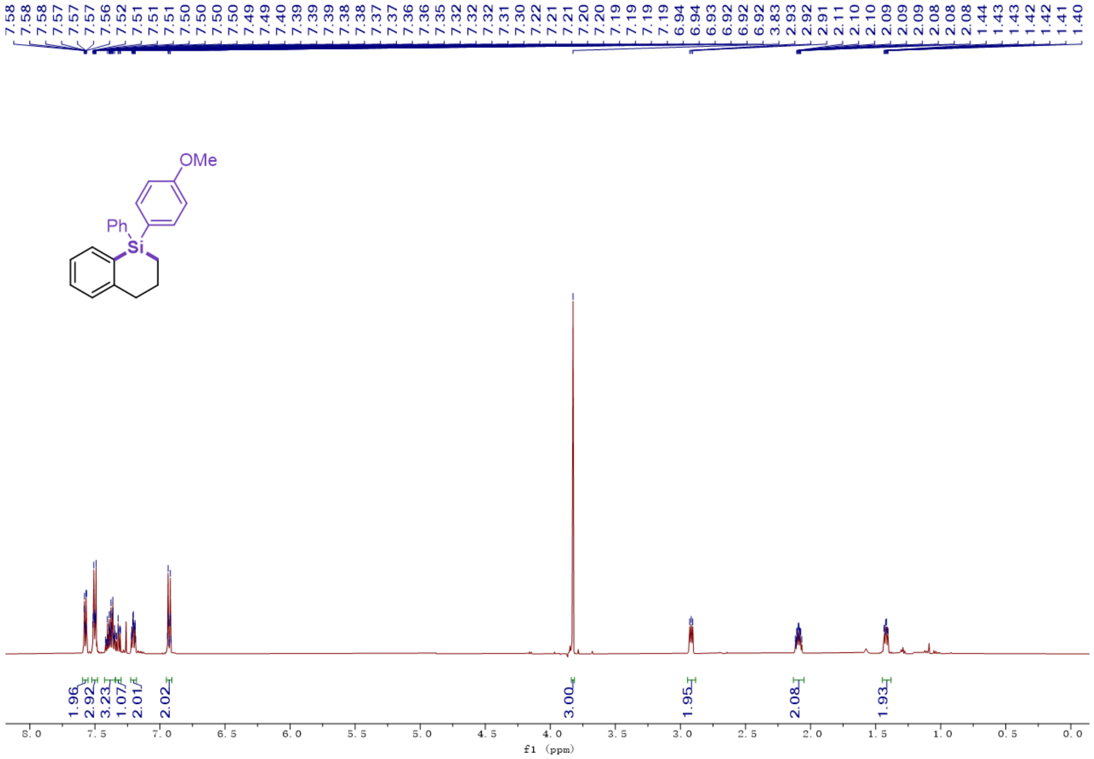

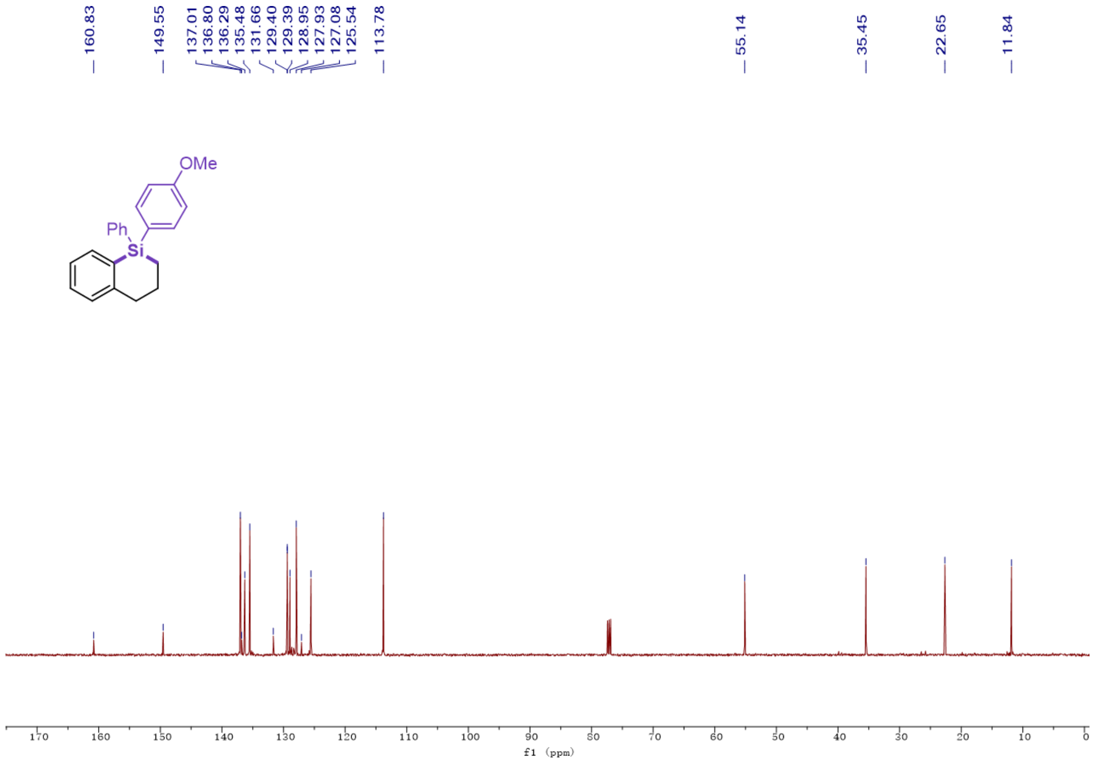

SUPPORTING INFORMATION

1-methyl-1-phenyl-1,2,3,4-tetrahydrobenzo[*b*]siline (**7d**)

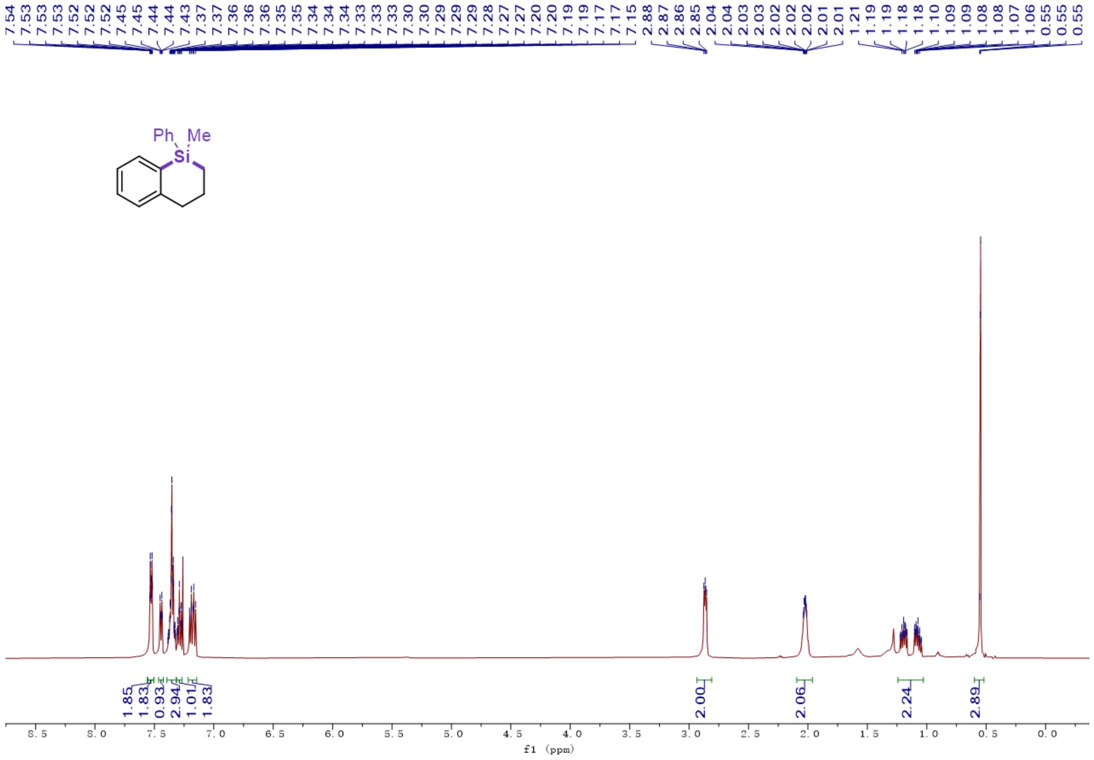

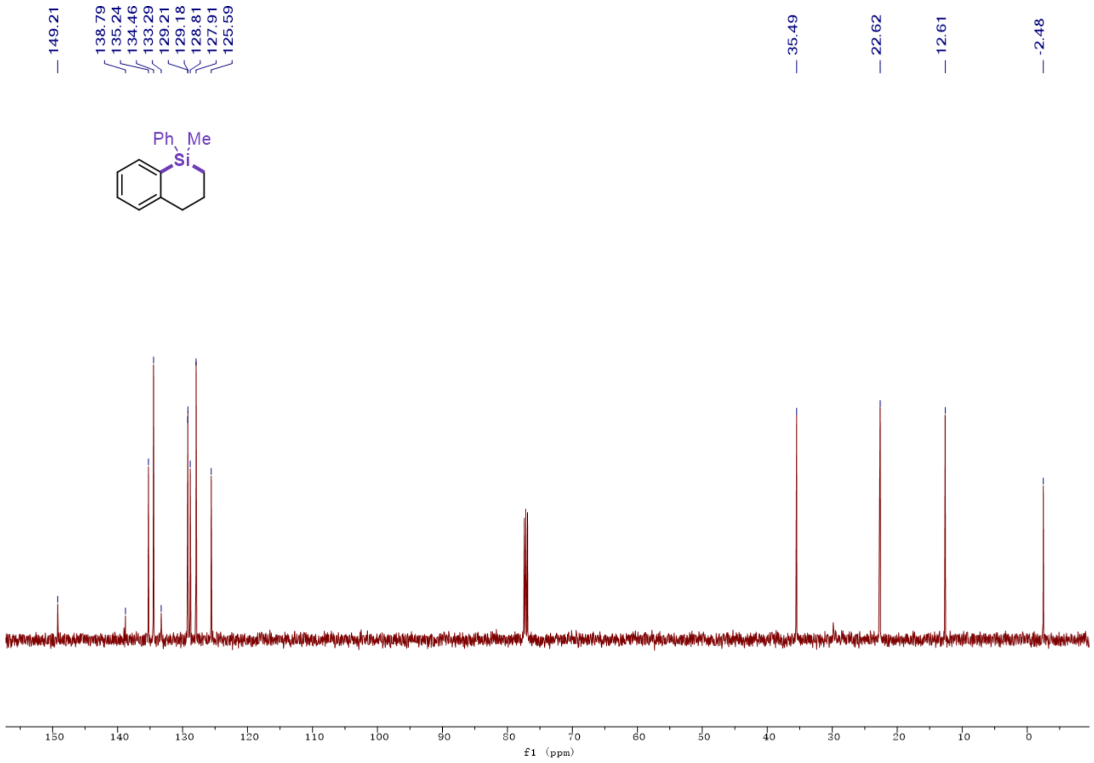

1,1-diethyl-7-methoxy-1,2,3,4-tetrahydrobenzo[*b*]siline (**7e**)

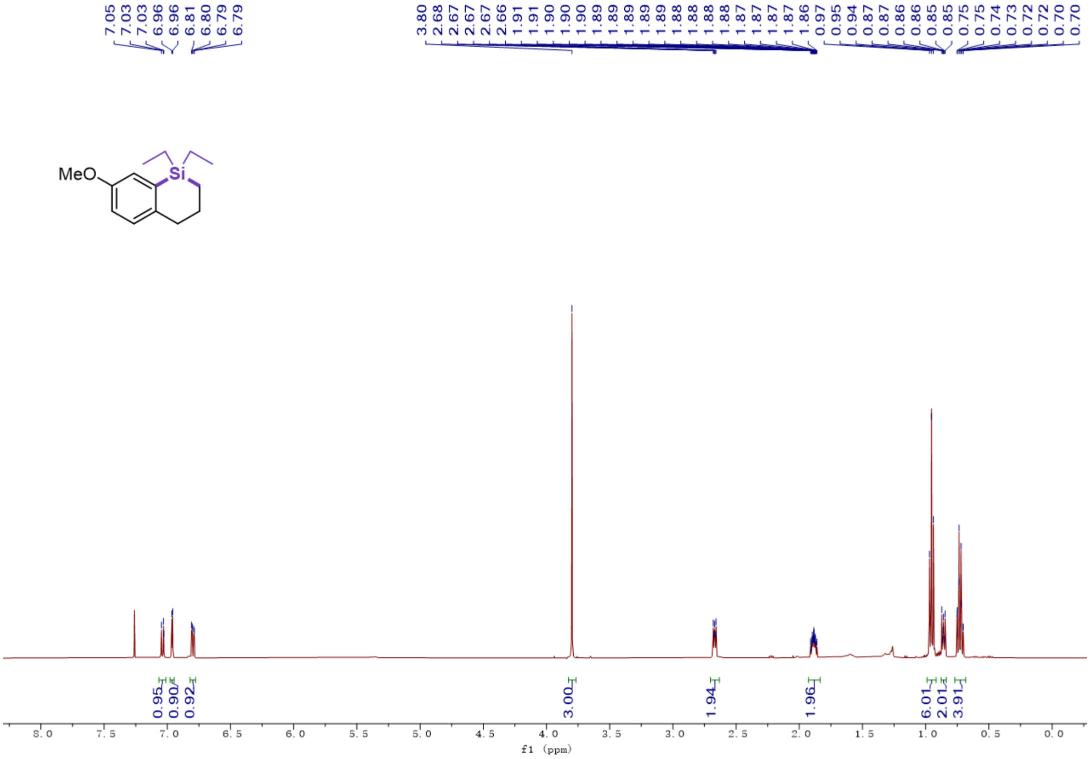

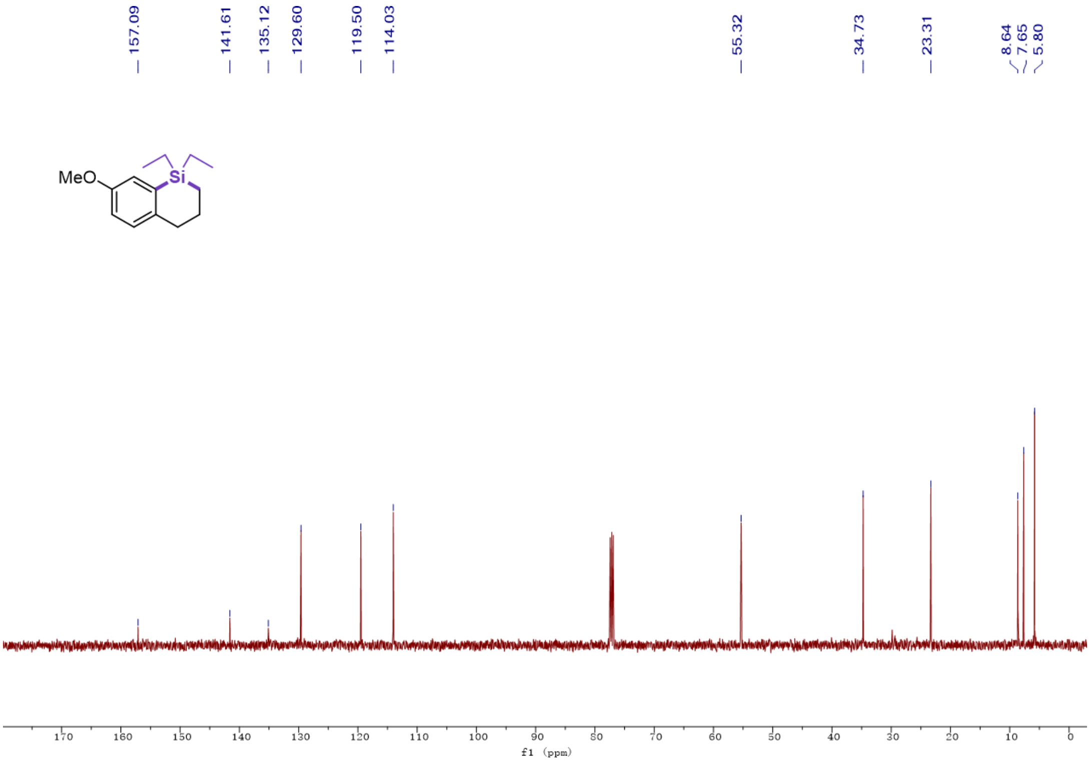

SUPPORTING INFORMATION

methyl(3-(perfluorophenyl)propyl)(phenyl)silane (**I-1**)

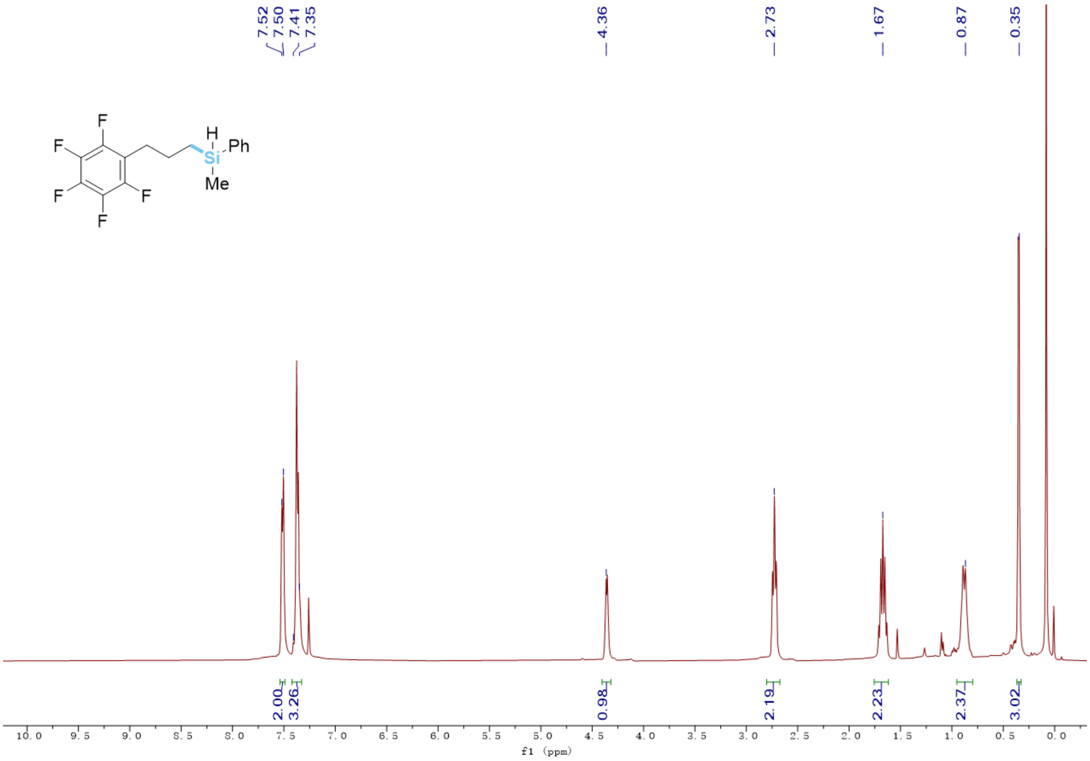

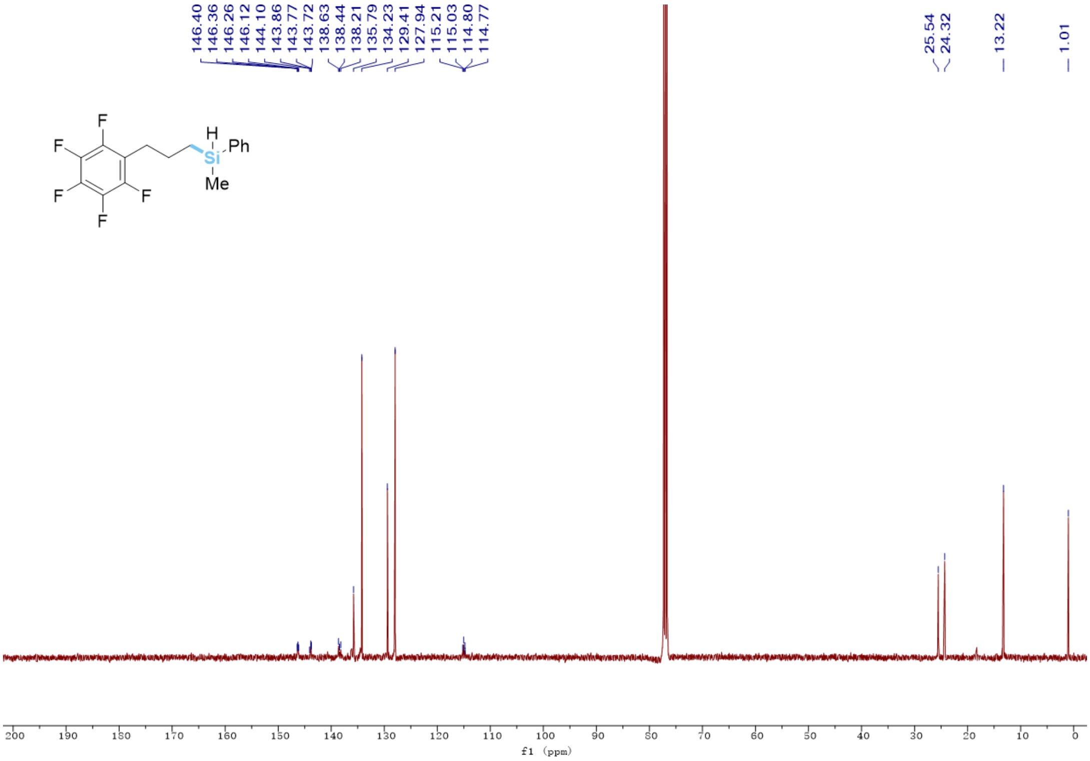

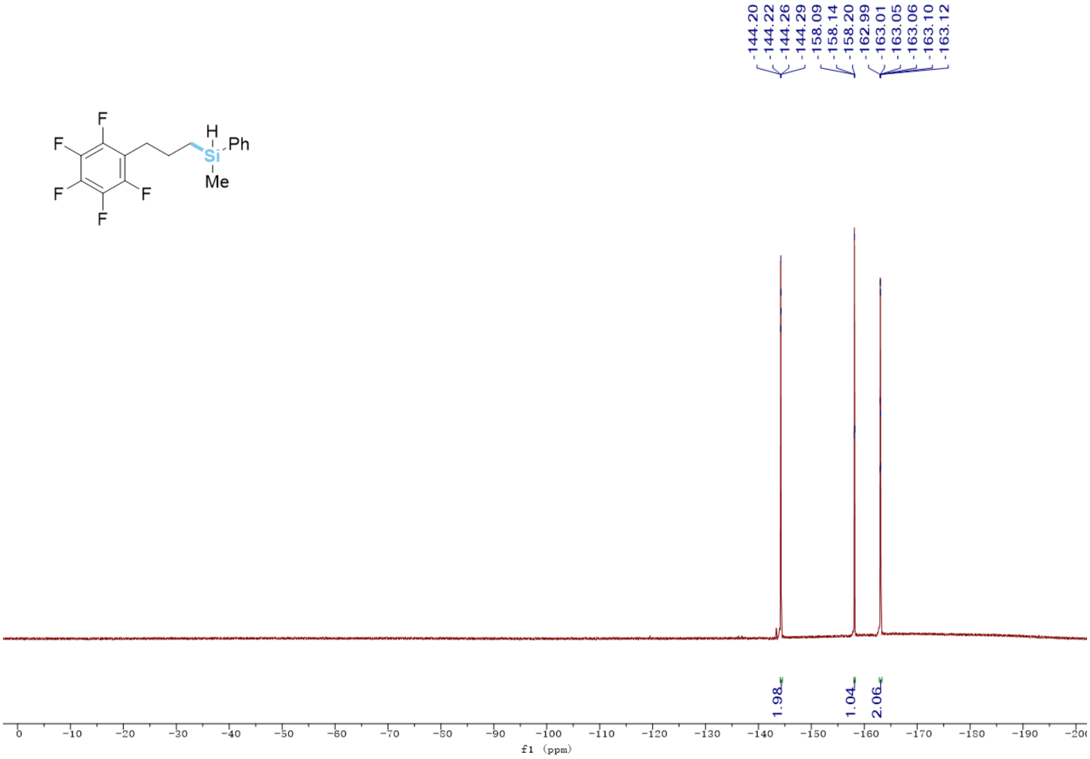

diphenyl(3-phenylpropyl)silane (**II-1**)

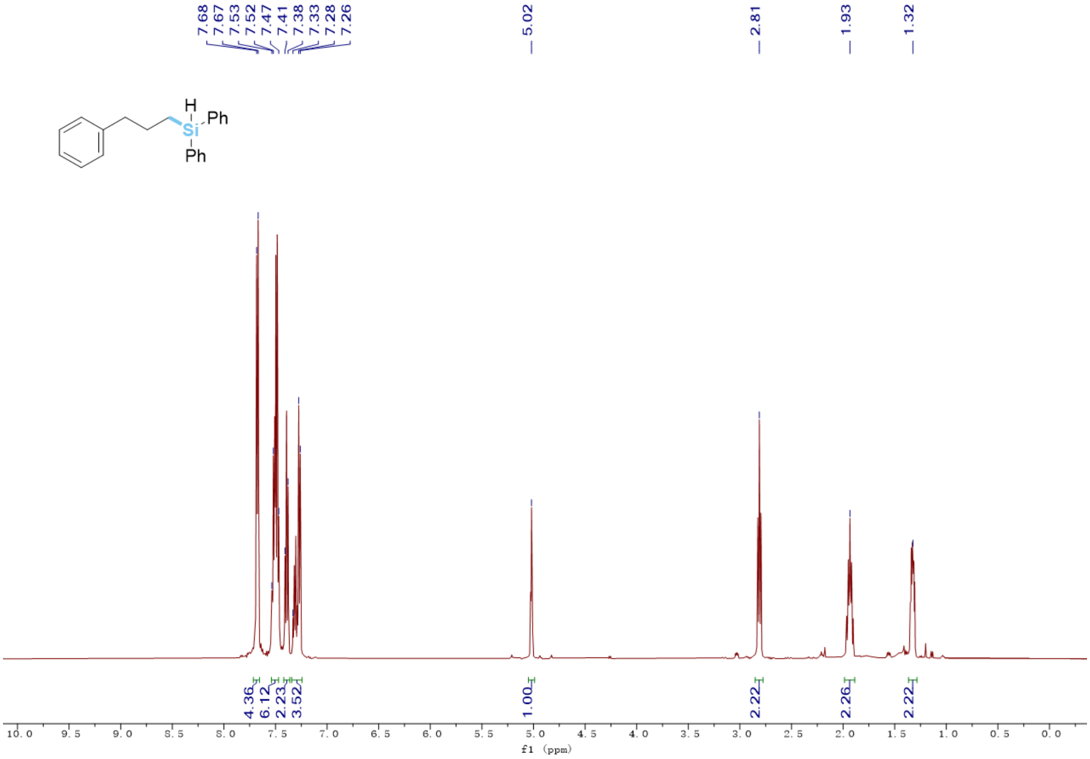

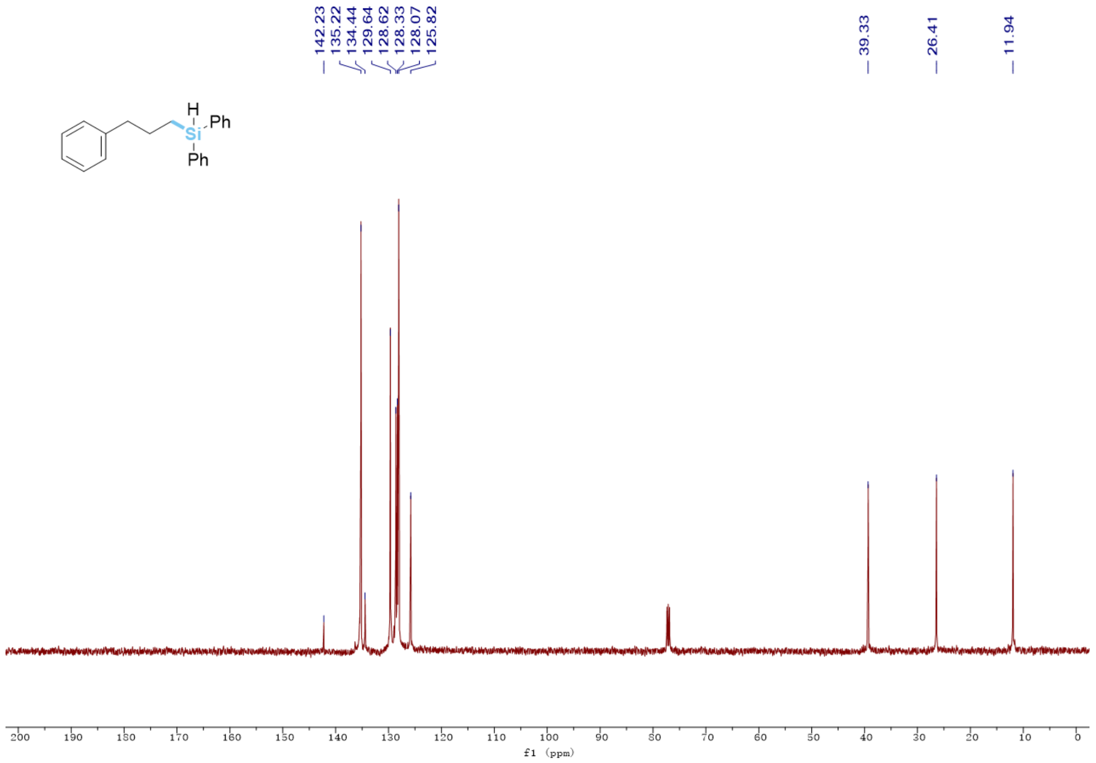

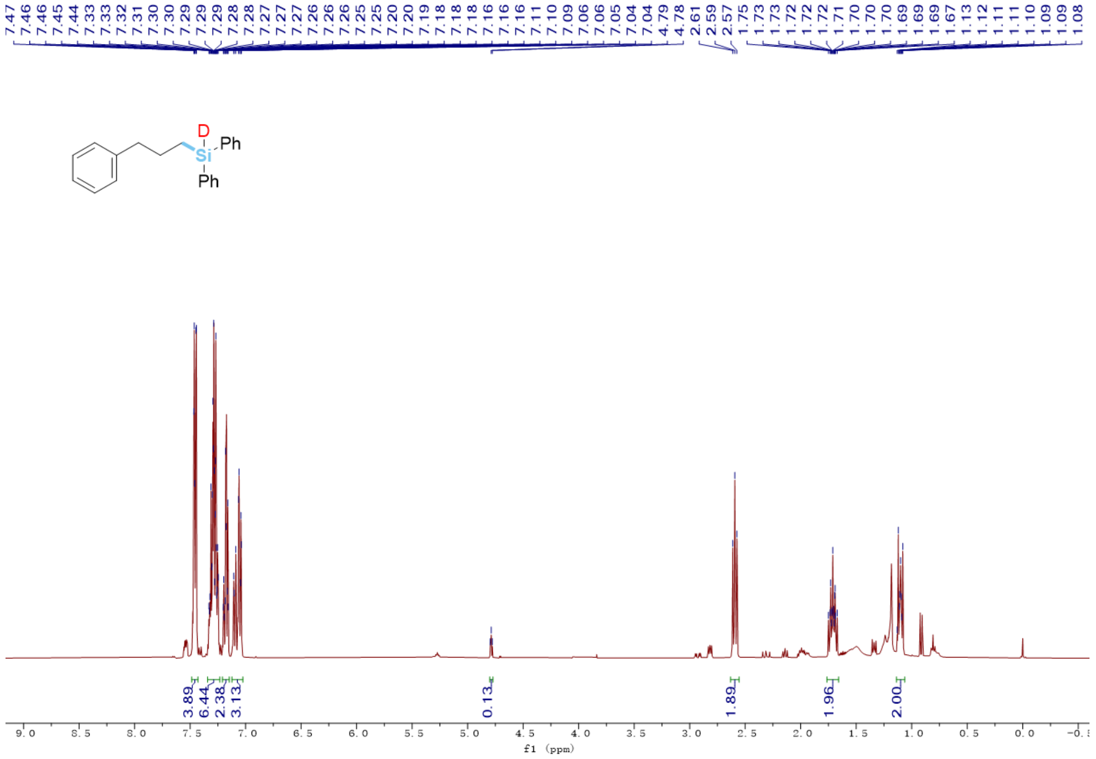

Supplement: Supplementary file 1 — Supporting Information [file ANIE-64-e202512420-s001.pdf]
